# Quantifying age-related disparities in outpatient psychotherapy utilization: a representation quotient analysis of routine data from 29 university clinics in Germany

**DOI:** 10.1186/s12913-025-13714-5

**Published:** 2025-11-25

**Authors:** Nicolas Wrede, Marlena L. Itz, Anne Katrin Risch, Hanna Christiansen, Tina In-Albon, Jürgen Hoyer, Tania M. Lincoln, Wolfgang Lutz, Jürgen Margraf, Julian Rubel, Rudolf Stark, Julia Velten, Katja Werheid, Ulrike Willutzki, Georg W. Alpers, Stephan Bartholdy, Elisa-Maria Berger, Eva-Lotta Brakemeier, Monika Equit, Lydia Fehm, Thomas Forkmann, Jessica Fritz, Julia Glombiewski, Jens Heider, Sylvia Helbig-Lang, Andrea Hermann, Christiane Hermann, Anke Kirsch, Tim Klucken, Ulrike Lueken, Patrizia Odyniec, Anya Pedersen, Andre Pittig, Babette Renneberg, Anja Riesel, Almut Rudolph, Brian Schwartz, Tobias Teismann, Antonia Vehlen, Michael Witthöft, Marcella L. Woud, Gabriele Wilz

**Affiliations:** 1 Department of Counseling and Clinical Intervention, Friedrich Schiller University Jena, Institute of Psychology, Humboldtstraße 11, 07743 Jena, Germany; 2 Institute of Psychosocial Medicine, Psychotherapy and Psychooncology, University Hospital Jena, Jena, Germany; 3 Clinical Child and Adolescent Psychology, Philipps University Marburg, Marburg, Germany; 4 German Center for Mental Health (DZPG), Partner Site, Bochum/Marburg, Germany; 5 Clinical Child and Adolescent Psychology and Psychotherapy, University of Mannheim, Mannheim, Germany; 6 Clinical Psychology and Psychotherapy, Technische Universität Dresden, Dresden, Germany; 7 Clinical Psychology and Psychotherapy, Universität Hamburg, Hamburg, Germany; 8 Clinical Psychology and Psychotherapy, Department of Psychology, Trier University, Trier, Germany; 9 Mental Health Research and Treatment Center, Faculty of Psychology, Ruhr University Bochum, Bochum, Germany; 10 Psychotherapy Research & Clinical Psychology, Department of Psychology, Osnabrück University, Osnabrück, Germany; 11 Psychotherapy and Systems Neuroscience, Justus Liebig University Giessen, Giessen, Germany; 12 Clinical Neuropsychology & Psychotherapy, Bielefeld University, Bielefeld, Germany; 13 Clinical Psychology and Psychotherapy, Department of Psychology and Psychotherapy, University Witten/Herdecke, Witten/Herdecke, Germany; 14 Department of Psychology, School of Social Sciences and Otto Selz Institute, University of Mannheim, Mannheim, Germany; 15 Department of Psychology, Clinical Psychology and Psychotherapy, University Greifswald, Greifswald, Germany; 16 Outpatient Department of Psychotherapy, Johannes Gutenberg-University, Mainz, Germany; 17 Department of Clinical Psychology and Psychotherapy, Saarland University, Saarbrücken, Germany; 18 Department of Psychology, Humboldt-Universität zu Berlin, Berlin, Germany; 19 Department of Clinical Psychology and Psychotherapy, University of Duisburg-Essen, Duisburg/Essen, Germany; 20 Clinical Psychology and Psychotherapy, University of Kaiserslautern-Landau, Kaiserslautern-Landau, Germany; 21 Psychotherapie Ausbildung Hamburg, Universität Hamburg, Hamburg, Germany; 22 Clinical Psychology and Psychotherapy, Justus Liebig University Giessen, Giessen, Germany; 23 Training Institute for Psychotherapy, Saarbrücken, Germany; 24 Department of Clinical Psychology and Psychotherapy, University of Siegen, Institute of Psychology, Siegen, Germany; 25 German Center for Mental Health (DZPG), Partner Site Berlin-Potsdam, Berlin/Potsdam, Germany; 26 Department of Clinical Psychology and Psychotherapy, Cristian-Albrechts-University Kiel, Kiel, Germany; 27 Translational Psychotherapy, Institute of Psychology, University of Göttingen, Göttingen, Germany; 28 Clinical Psychology and Psychotherapy, Freie Universität Berlin, Berlin, Germany; 29 Clinical Psychology and Neuroscience, Universität Hamburg, Hamburg, Germany; 30 Clinical Psychology and Psychotherapy, Department of Psychology, University of Leipzig, Leipzig, Germany; 31 Department of Clinical Psychology and Psychotherapy, Ruhr University Bochum, Bochum, Germany; 32 Clinical Psychology and Experimental Psychopathology, University of Göttingen, Göttingen, Germany

**Keywords:** Older adults, Psychotherapy utilization, Representation quotient, Mental health services, Age disparities, Access barriers

## Abstract

**Background:**

Although mental disorders are highly prevalent among older adults, evidence suggests that they underutilize psychotherapy. However, formal estimates of their actual representation in routine clinical settings are scarce. This study applied a representation quotient approach to identify and quantify age-related disparities in outpatient psychotherapy utilization in Germany.

**Methods:**

We analyzed data from 13,635 adult patients who initiated cognitive-behavioral therapy between 2018 and 2023 at 29 university outpatient clinics. Using a representation quotient approach, we compared the observed proportions of young-old adults (65–74 years) and old-old adults (≥75 years) with prevalence-stratified population age distributions. The robustness of the results was investigated by extensive sensitivity analyses, including alternative prevalence assumptions and adjustments for long-term care needs.

**Results:**

Even under conservative assumptions, young-old adults only accounted for about 25% and old-old adults for about 13% of their expected proportion. In contrast, young working-age adults (18–34 years) were particularly overrepresented in the sample. Underrepresentation of older adults was most pronounced among patients with post-traumatic stress disorder, eating disorders, and dysthymia, less pronounced among patients with generalized anxiety disorder, and not significant among patients with pain disorders.

**Conclusion:**

This study demonstrates the utility of representation quotients for systematically identifying and quantifying age-related disparities in psychotherapy utilization based on routine care data. Our analyses of large-scale data from university outpatient clinics in Germany revealed a marked underrepresentation of older adults in this setting. These findings highlight the need to improve access to, and utilization of, psychotherapeutic services for older adults.

**Supplementary Information:**

The online version contains supplementary material available at 10.1186/s12913-025-13714-5.

## Background

There is a global demographic shift towards population aging due to increasing life expectancy and declining birth rates [1]. This shift presents significant challenges for healthcare systems, which must adapt to the growing demand for both medical and psychosocial care for an increasing number of older adults [2]. Mental disorders rank among the leading causes of disease burden worldwide [3, 4], are associated with high economic costs [5, 6], and remain highly prevalent in later life [7, 8]. According to a population-based study in Germany, the setting of the present study, the 12-month prevalence of any mental disorder is at 20.3% among older adults (65–79 years), compared to approximately 30% among working-age adults (18–64 years) [9]. Further, a recent study using age-sensitive diagnostic measures reported a 12-month prevalence of any mental disorder of 35.2% among people aged 65 years or older across multiple European countries [8]. Given these findings, ensuring adequate mental healthcare for older adults remains an urgent priority for healthcare systems worldwide [10].

Psychotherapy is an effective treatment for common mental disorders [11–13], and evidence suggests that older adults benefit from psychotherapy to a similar extent as working-age adults. This is supported by both meta-analyses of randomized controlled trials on efficacy [14–17] and recent large-scale studies on effectiveness under routine care conditions [18–21]. Despite these findings, research on health service utilization consistently finds lower psychotherapy utilization rates among older compared to working-age adults [19, 22–29]. Instead of receiving psychotherapy, older adults are more frequently prescribed psychotropic medication [30–32], although they face an elevated risk of drug interactions and adverse side effects [33, 34]. Hence, the international evidence consistently suggests an age gap in psychotherapy utilization.

Germany represents a particularly distinctive context for examining age-related disparities in psychotherapy utilization: even though the German statutory health insurance provides universal coverage for psychotherapeutic treatment, regardless of a person’s age, previous studies have documented significantly lower utilization among older adults. A recent nationwide survey of more than 12,000 adults found that older age was a significant predictor of non-utilization of psychotherapy [35]. Fittingly, studies on routine outpatient psychotherapy in Germany indicate that older adults account for only a small proportion of treated patients. Several therapist surveys have examined the share of older adults among psychotherapy patients in private practice. For instance, a survey among therapists in a region of Westphalia conducted in 2008 found that only 2.4% of patients were aged 65 years or older [36]. Additionally, two surveys among psychotherapists in Berlin conducted in 2012 and 2022 reported that approximately 9 and 12% of patients, respectively, were aged 60 years or older [37, 38]. Further, a recent analysis of a large statutory health insurance dataset comprising more than 11 million adults aged 18–79 who received psychotherapy between 2015 and 2019 found that only about 13% of patients were 60 years or older [39; own calculations based on supplementary material].

### Quantifying underrepresentation of older adults in outpatient psychotherapy

Taken together, previous studies indicate that older adults constitute only a relatively small share of patients in routine outpatient psychotherapy in Germany. However, such descriptive figures alone are insufficient to determine whether this reflects genuine underrepresentation or to quantify its extent. Instead, the concept of underrepresentation presupposes a comparison between two groups: (1) an observed sample (i.e., patients who actually receive a given treatment) and (2) a reference population (i.e., individuals in the general population who would reasonably be eligible for this treatment). In the context of outpatient psychotherapy, the reference population would encompass all individuals with a mental disorder who are eligible to receive psychotherapy. Underrepresentation occurs when the proportion of older adults in the observed sample is substantially lower than their proportion in the reference population, indicating that, within the reference population, the probability of receiving treatment depends on age.

Based on this conceptual foundation, a methodological approach that formalizes the quantification of underrepresentation is the use of representation quotients, which are rooted in research on gender and ethnic representation [40, 41]. The representation quotient is defined as the ratio between the proportion of a subgroup in the observed sample and its proportion in the reference population. Hence, a representation quotient (RQ) of 1 indicates perfect representation, while RQ < 1 indicates underrepresentation, and RQ > 1 overrepresentation of a certain age group in the observed sample. For example, if older adults constitute 30% of the reference population of individuals with a mental disorder who would be reasonably eligible for psychotherapy, but account for only 15% of those actually receiving psychotherapy, their representation quotient would be 0.15 / 0.30 = 0.50. This would indicate that they are represented at only half of the expected level. Importantly, the reference population is typically non-observed and must therefore be constructed based on assumptions. These may include the age distribution of the general population, the age-specific prevalence of mental disorders, and other age-related factors that may affect eligibility for outpatient psychotherapy (e.g., long-term care needs).

This approach has two key advantages. First, it moves beyond mere descriptive figures of sample composition and instead provides specific estimates of representation on an interpretable quantitative scale, while accounting for potential alternative explanations of low shares of older adults in observed psychotherapy samples. It also allows statistical testing of whether observed proportions differ significantly from expected values. Second, and perhaps most importantly, it delivers such estimates under transparent and formally specified assumptions about the reference population. By making these assumptions explicit, the approach ensures transparency regarding the presuppositions underlying any claim of underrepresentation, requires researchers to justify their assumptions, and permits sensitivity analyses to assess their impact on the resulting estimates.

To date, no study on outpatient psychotherapy in Germany has systematically applied this methodological rationale to formally identify and quantify the underrepresentation of older adults. As outlined above, most research has been limited to reporting descriptive figures of the proportion of older adults in psychotherapy samples, often supplemented by narrative comparisons to population shares [37–39]. Only in rare cases have studies conducted a formal comparison with a reference population. For instance, Imai et al. [36] contrasted the observed proportion of older adults in their sample with their share in the regional population but did not account for age-specific prevalence rates of mental disorders. Internationally, Chaplin et al. [19] analyzed a large routine care dataset from the UK and compared the observed frequency of older adults with age-adjusted prevalence estimates. They found that adults aged 65 years and older were represented at only about 50% of the expected value. While conceptually close to a representation quotient approach, their analyses did not fully exploit the potential of this framework, as they neither tested the robustness of their estimates under varying assumptions (e.g., alternative prevalence estimates) nor considered additional age-related factors that might justify exclusion from the reference population (e.g., long-term care needs).

### University outpatient clinics in Germany

Beyond psychotherapists in private practice, university outpatient psychotherapy clinics play a pivotal role in the outpatient mental healthcare system in Germany. They serve as postgraduate training centers, research hubs, and clinical providers. Moreover, these clinics accommodate patient volumes equivalent to approximately 5 to 15 private practices [42–45], and frequently provide care for individuals with complex mental disorders who might otherwise face significant barriers to receiving treatment. It is important to note that while these clinics are embedded within universities for the purposes of research and postgraduate training, they do not specifically target students as patients. Rather, they are officially licensed providers within the statutory health insurance system serving the general population. In light of their influence through educating the next generation of psychotherapists and their model character for innovative care, university clinics have the potential to be a crucial setting for addressing barriers to psychotherapy utilization in older age. Despite the relevance of these clinics in the German mental healthcare system, no study has systematically investigated age-related utilization differences in this setting.

### Present study

The aim of this study was to identify and quantify age-related disparities in outpatient psychotherapy utilization in Germany using a representation quotient approach. Drawing on routine data from 29 university outpatient clinics, we systematically compared the age distribution in the observed patient sample with the expected distribution in a reference population of adults with a mental disorder who would be eligible for psychotherapy. Our goal was to derive interpretable estimates of representation based on transparent assumptions about the reference population and to assess their robustness under varying assumptions.

## Methods

### Participants

We analyzed data from university outpatient psychotherapy clinics in Germany of the KODAP research network (acronym for “Coordination of Data Acquisition at Research Clinics for Psychotherapy”). The network addresses research and clinical care questions by harmonizing and merging routine assessments [43–48]. The study procedures have been registered in the German Clinical Trials Register (DRKS00015883; registered on 16 July 2021: https://drks.de/search/en/trial/DRKS00015883) and approved by the Ethics Committee of the Faculty of Psychology at Ruhr University Bochum (committee number 228). All patients provided informed consent prior to participation. We created a retrospective dataset including 13,635 patients aged 18 years or older who initiated psychotherapeutic treatment between 2018 and 2023 in one of 29 KODAP clinics.

### Treatments

All investigated patients received cognitive-behavioral therapy (CBT). In Germany, CBT is one of four guideline-based psychotherapeutic modalities reimbursed under statutory health insurance (alongside psychodynamic therapy, psychoanalysis, and systemic therapy). Importantly, CBT represents the largest share of reimbursed outpatient psychotherapies with approximately half of all adult treatments [49–51], and is the predominant modality offered at university outpatient clinics [42, 52]. We included patients regardless of whether their treatment was ongoing, regularly finished, prematurely terminated, or currently suspended. While age group differences in treatment duration, attrition, and effectiveness in the KODAP sample have previously been reported elsewhere [20], the current study focuses on providing estimates of representation of older adults among the sample.

### Instruments and measures

#### Demographic data

Demographic data were documented by standardized assessments. These included age, gender, marital status, relationship status, and education.

#### Clinical diagnoses

A maximum of five mental disorder diagnoses were recorded for each patient prior to the start of treatment. In order to ensure high data quality, we only included patients whose diagnoses were assessed by structured clinical interviews such as the Structured Clinical Interview for DSM-IV (SCID; [53]) or DSM-5 [54] and the Diagnostic Interview for Mental Disorders (DIPS; [55]), or diagnostic checklists [56].

### Data analysis

All data were analyzed using R version 4.4.1 [57]. All statistical input and output code is given in the Supplementary Material. Age was treated as a categorical variable, divided into three groups: working-age adults (18–64 years), young-old adults (65–74 years), and old-old adults (≥75 years). This classification aligns with recent calls for a granular examination of aging populations and current recommendations for psychotherapy research in later life [14, 58, 59].

To quantify the representation of older adults among the sample, we followed a representation quotient approach. The representation quotient was defined as the ratio between the proportion of a certain age group in the observed sample and the proportion of this respective age group in the reference population of people with mental disorders. To approximate these proportions of young-old and old-old adults in the reference population, we integrated demographic census data from the German Federal Statistical Office [60] with age-specific prevalence estimates of mental disorders from the most recent population-based epidemiological study conducted in Germany [9, 61]. The complete prevalence assumptions (Supplement B, Table S1), as well as the detailed approximation of the age distribution in the reference population including example step-by-step calculations (Supplement A1) are provided in the Supplementary Material.

Representation quotients were calculated by dividing the proportion of a specific age group in the observed sample by their corresponding proportion in the reference population. 95% confidence intervals of representation quotients were obtained by dividing the 95% confidence interval around the sample proportion of a specific age group by their proportion in the reference population. Additionally, we conducted exact binomial tests with Bonferroni-corrected p-values to assess deviations of observed sample proportions of specific age groups from their proportions in the reference population.

In addition to global estimates of representation in the complete sample, we estimated representation among specific disorder groups, using disorder-specific prevalence estimates [9, 61]. Patients were included in disorder-specific analyses when the respective disorder was one of the five possible diagnoses assigned to each patient.

To account for potential differences in representation within the working-age group, we further subdivided it into three subcategories, following Jacobi et al. [9, 61]: early working-age adults (18–34 years), middle working-age adults (35–49 years), late working-age adults (50–64 years). We then recalculated the expected age distributions using only the respective working-age subgroup and compared them to subsamples that included only this subgroup, along with young-old and old-old adults.

Finally, we conducted all analyses separately for male and female patients. Therefore, we used gender-specific prevalences of mental disorders in Germany [9, 61] which are given in the Supplementary Material (Supplement B, Table S2).

#### Sensitivity analyses

Within the representation quotient approach of our study, several assumed input variables for calculating the age group distribution within the reference population were subject to uncertainty. To examine the impact of these assumptions, we conducted several sensitivity analyses, both for the main analyses and for the disorder-specific analyses.

**Prevalence assumptions.** First, we accounted for potential uncertainties and biases in the assumed age-specific prevalence estimates. More specifically, we considered two plausible sources of bias, both originating from the population-based study that served as the primary basis for our prevalence assumptions [9, 61]. The first issue concerns that study’s report of a substantially lower prevalence of mental disorders in older adults compared to working-age adults. More recent research suggests that when age-sensitive assessment methods are applied, prevalence rates in older adults are comparable to those observed in younger age groups [8]. Therefore, it is plausible that Jacobi et al. [9, 61] may have underestimated the true prevalence of mental disorders in later life. To examine the potential consequences of this underestimation, we repeated all analyses under the extreme assumption of equal prevalence rates across age groups. The second issue relates to the fact that Jacobi et al. [9, 61] only reported a single prevalence estimate for all individuals aged 65 and older, requiring us to use this value as a proxy for both young-old and old-old adults. However, recent prevalence studies have consistently shown a decline in prevalence rates within the older age range [8, 62, 63]. To evaluate the impact of this potential misassumption, we repeated all analyses assuming a pronounced age-related decrease in prevalence by 50% from the young-old to the old-old group.

**Influence of long-term care needs.** Second, our main analyses assumed that all older adults with a mental disorder constitute a suitable patient group for outpatient psychotherapy. However, outpatient psychotherapy may be fundamentally inaccessible for certain subgroups, particularly for older adults who require long-term home-based or inpatient care [64]. Including these individuals in the reference population for outpatient psychotherapy may therefore lead to inflated estimates of underrepresentation. To assess the potential size of this bias, we recalculated the age distribution of the reference population, excluding individuals with any long-term care needs as defined by the German statutory nursing care insurance. Age-specific rates of long-term care dependency were obtained from the German Federal Statistical Office [65]. A detailed description of the recalculation of the reference population excluding individuals in need of long-term care is provided in the Supplementary Material (Supplement A2).

## Results

The dataset included *N* = 13,635 adults aged 18–96 years, of whom 13,218 (96.9%) were working-age adults (18–64 years), 324 (2.4%) young-old adults (65–74 years), and 93 (0.7%) old-old adults (≥75 years). A fine-graded description of the complete age distribution in the sample is given in the Supplementary Material (Supplement B, Table S3). Sample characteristics are presented in Table 1. Compared to working-age adults, older adults comprised more females, and were more often married or widowed. Further, older adults reported lower school education compared to working-age adults, while working-age adults were more frequently working towards a tertiary education degree. Older adults had fewer primary diagnoses than working-age adults. Moreover, older adults were less often diagnosed with major depressive disorder, social phobia, post-traumatic stress disorder (PTSD), and eating disorders, while they were more often diagnosed with pain disorders than working-age adults.

**Table 1:** Sample characteristics in the subsamples of working-age adults (18–64 years), young-old adults (65–74 years), and old-old adults (≥75 years)

	Working-age adults (*N* = 13,218)	Young-old adults (*N* = 324)	Old-old adults (*N* = 93)	F/χ2
Age, *M (SD)*	35.25 (12.44)	68.24 (2.6)	78.82 (4.08)	3236.7***
Female, *N* (%)	8,447 (64.0)	224 (69.1)	67 (72.0)	6.24*
Marital status, N (%)				
* Single*	6,859 (51.9)	25 (7.7)	2 (2.2)	1395.60***
* Married*	2,680 (20.3)	145 (44.8)	32 (34.4)	
* Divorced*	1,034 (7.8)	52 (16.0)	8 (8.6)	
* Widowed*	105 (0.8)	36 (11.1)	29 (31.2)	
* Other*	1,327 (10.0)	20 (6.2)	1 (1.1)	
* No data*	1,213 (9.2)	46 (14.2)	21 (22.6)	
In a partnership, N (%)				
* Yes*	5,821 (44.0)	153 (47.2)	30 (32.3)	7.52*
* No*	4,868 (36.8)	91 (28.1)	32 (34.4)	
* No data*	2,529 (19.1)	80 (24.7)	31 (33.3)	
School education, N (%)				
* Still in school*	189 (1.4)	0 (0.0)	0 (0.0)	108.10***
* No degree*	126 (1.0)	1 (0.3)	0 (0.0)	
* Basic school certificate (German “Hauptschulabschluss”)*	1,134 (8.6)	66 (20.4)	16 (17.2)	
* Intermediate school certificate (German “Realschulabschluss”)*	2,607 (19.7)	75 (23.1)	21 (22.6)	
* High school degree (German “Abitur”)*	6,541 (49.5)	94 (29.0)	26 (28.0)	
* Other*	212 (1.6)	2 (0.6)	1 (1.1)	
* No data*	2,409 (18.2)	86 (26.5)	29 (31.2)	
Education, N (%)				
* Working towards degree*	1,725 (13.1)	0 (0.0)	0 (0.0)	75.83***
* No degree*	838 (6.3)	9 (2.8)	1 (1.1)	
* Vocational training*	2,987 (22.6)	88 (27.2)	24 (25.8)	
* College or university degree*	2,523 (19.1)	57 (17.6)	19 (20.4)	
* Other*	2,352 (17.8)	70 (21.6)	20 (21.5)	
* No data*	2,793 (21.1)	100 (30.9)	29 (31.2)	
Number of clinical diagnoses, *M (SD)*	1.67 (0.85)	1.52 (0.81)	1.28 (0.52)	28.53***
Clinical diagnoses, N (%)				
* Any mood disorder*	8,027 (60.7)	179 (55.2)	38 (40.9)	19.03***
* Major Depressive Disorder*	7,261 (54.9)	163 (50.3)	36 (38.7)	12.41**
* Dysthymia*	935 (7.1)	22 (6.8)	1 (1.1)	5.12
* Any anxiety disorder*	4,318 (32.7)	106 (32.7)	29 (31.2)	0.09
* Panic disorder/Agoraphobia*	1,400 (10.6)	47 (14.5)	9 (9.7)	5.18
* Social phobia*	1,830 (13.8)	16 (4.9)	4 (4.3)	28.25***
* Specific phobias*	678 (5.1)	22 (6.8)	3 (3.2)	2.50
* GAD*	526 (4.0)	20 (6.2)	6 (6.5)	5.31
* OCD*	778 (5.9)	11 (3.4)	2 (2.2)	5.87
* PTSD*	1,052 (8.0)	13 (4.0)	2 (2.2)	11.01**
* Any somatoform disorder*	1,009 (7.6)	48 (14.8)	17 (18.3)	36.44***
* Somatization disorder*	203 (1.5)	7 (2.2)	1 (1.1)	0.95
* Pain disorder*	465 (3.5)	30 (9.3)	13 (14.0)	56.51***
* Eating disorders*	849 (6.4)	8 (2.5)	0 (0.0)	14.67***
* Substance use disorders*	748 (5.7)	15 (4.6)	1 (1.1)	4.26
* Psychotic disorders*	331 (2.5)	4 (1.2)	1 (1.1)	2.87

Note. GAD = Generalized Anxiety Disorder; OCD = Obsessive Compulsive Disorder; PTSD = Post-Traumatic Stress Disorder; F = F-statistics from one-way ANOVA to test differences between age groups on continuous outcomes, χ^2^ = test statistics from χ^2^-test to test differences between age groups on categorial outcomes, *: *p* < .05; **: *p* < .01, ***: *p* < .001

### Representation quotient analysis

The detailed calculation of the age distribution of the reference population is provided in the Supplementary Material (Supplement A1). Young-old adults were estimated to account for 9.3% of the reference population but represented only 2.4% of the observed sample (95%-CI=[2.1%; 2.6%]). Hence, the representation quotient was calculated as follows: RQ = 0.024 / 0.093 = 0.26 (95%-CI = [0.23; 0.29]). This indicates that only one quarter of the expected number of young-old adults were observed. Old-old adults made up 9.7% of the reference population but only 0.7% of the observed sample (95%-CI = [0.6%; 0.8%]), resulting in a representation quotient of 0.007 / 0.097 = 0.07 (95%-CI = [0.06; 0.09]). Thus, only about 7% of the expected old-old adults were represented in the dataset. The complete results from the representation quotient analysis are presented in Table 2.

**Table 2:** Global and disorder-specific results from representation quotient analysis

Disorder	Reference population proportions (%)			Observed sample N (%)			Representation quotient [95%-CI]	
	Working-Age	Young-old	Old-old	Working-Age	Young-old	Old-old	Young-old	Old-old
Any mental disorder	81.0	9.3	9.7	13,218 (96.9)	324 (2.4)	93 (0.7)	0.26 [0.23; 0.29]***	0.07 [0.06; 0.09]***
Any mood disorder	83.4	8.1	8.5	8,027 (97.4)	179 (2.2)	38 (0.5)	0.27 [0.23; 0.31]***	0.05 [0.04; 0.08]***
* Major Depressive Disorder*	82.4	8.6	9.0	7,261 (97.3)	163 (2.2)	36 (0.5)	0.25 [0.22; 0.30]***	0.05 [0.04; 0.07]***
* Dysthymia*	74.6	12.4	13.0	935 (97.6)	22 (2.3)	1 (0.1)	0.18 [0.12; 0.28]***	0.01 [0.00; 0.05]***
Any anxiety disorder	80.6	9.5	9.9	4,318 (97.0)	106 (2.4)	29 (0.7)	0.25 [0.21; 0.30]***	0.07 [0.04; 0.10]***
* Panic disorder/Agoraphobia*	76.7	11.4	11.9	1,400 (96.2)	47 (3.2)	9 (0.6)	0.28 [0.21; 0.38]***	0.05 [0.03; 0.10]***
* Social phobia*	92.8	3.5	3.7	1,830 (98.9)	16 (0.9)	4 (0.2)	0.25 [0.15; 0.41]***	0.06 [0.02; 0.16]***
* Specific phobias*	78.4	10.6	11.0	678 (96.4)	22 (3.1)	3 (0.4)	0.30 [0.19; 0.45]***	0.04 [0.01; 0.12]***
* GAD*	84.5	7.6	7.9	526 (95.3)	20 (3.6)	6 (1.1)	0.48 [0.30; 0.74]***	0.14 [0.06; 0.31]***
OCD	91.5	4.1	4.3	778 (98.4)	11 (1.4)	2 (0.3)	0.34 [0.18; 0.62]***	0.06 [0.01; 0.23]***
PTSD	78.3	10.6	11.1	1,052 (98.6)	13 (1.2)	2 (0.2)	0.12 [0.06; 0.20]***	0.02 [0.00; 0.07]***
Any somatoform disorder	83.7	8.0	8.3	1,009 (93.9)	48 (4.5)	17 (1.6)	0.56 [0.42; 0.74]***	0.19 [0.11; 0.31]***
* Somatization disorder*	73.8	12.8	13.4	203 (96.2)	7 (3.3)	1 (0.5)	0.26 [0.11; 0.55]***	0.04 [0.00; 0.23]***
* Pain disorder*	86.2	6.8	7.1	465 (91.5)	30 (5.9)	13 (2.6)	0.87 [0.60; 1.24]	0.36 [0.20; 0.63]***
Eating disorders	89.0	5.4	5.6	849 (99.1)	8 (0.9)	0 (0.0)	0.17 [0.08; 0.35]***	0.00 [0.00; 0.10]***
Substance use disorders	88.0	5.9	6.1	748 (97.9)	15 (2.0)	1 (0.1)	0.33 [0.19; 0.56]***	0.02 [0.00; 0.14]***
Psychotic disorders	86.4	6.6	6.9	331 (98.5)	4 (1.2)	1 (0.3)	0.18 [0.06; 0.49]***	0.04 [0.00; 0.28]***

Note. GAD = Generalized Anxiety Disorder; OCD = Obsessive Compulsive Disorder; PTSD = Post-Traumatic Stress Disorder; *: *p* < 0.05, **: *p* < 0.01, ***: *p* < 0.001, p-values are from Bonferroni-corrected post-hoc binomial tests for age group specific deviations of observed proportions from reference population proportions. Representation quotients indicate the ratio between the proportion of an age group in the observed sample and their expected proportion in the reference population of German adults with a mental disorder

Disorder-specific results indicated underrepresentation of older adults across mental disorders. With the exception of pain disorders, young-old adults were significantly underrepresented in all disorder subgroups, with estimates of underrepresentation ranging from RQ = 0.12 to RQ = 0.87. Particularly strong underrepresentation of young-old adults was found for PTSD (RQ = 0.12, 95%-CI = [0.06; 0.20]), eating disorders (RQ = 0.17, 95%-CI = [0.08; 0.35]), dysthymia (RQ = 0.18, 95%-CI = [0.12; 0.28]), and psychotic disorders (RQ = 0.18, 95%-CI = [0.06; 0.49]), while they were less underrepresented among patients with generalized anxiety disorder (GAD; RQ = 0.48, 95%-CI = [0.30; 0.74]) and not significantly underrepresented among patients with pain disorders (RQ = 0.87, 95%-CI = [0.60; 1.24]).

Old-old adults were significantly underrepresented in all disorder subgroups with estimates ranging from RQ = 0.00 to RQ = 0.36. Strongest underrepresentation was estimated among patients with eating disorders (RQ = 0.00, 95%-CI = [0.00; 0.10]), dysthymia (RQ = 0.01, 95%-CI = [0.00; 0.05]), PTSD (RQ = 0.02, 95%-CI = [0.00; 0.07]), and substance use disorders (RQ = 0.02, 95%-CI = [0.00; 0.14]). Old-old adults were less underrepresented for pain disorders (RQ = 0.36, 95%-CI = [0.20; 0.63]) and GAD (RQ = 0.14, 95%-CI = [0.06; 0.31]).

Underrepresentation of older adults was more pronounced when estimated relative to early working-age adults (18–34 years) rather than to older subgroups within the working-age category. Despite these variations, both young-old and old-old adults remained significantly underrepresented compared to any subgroup of working-age adults. However, when examining individual mental disorders, young-old adults were not significantly underrepresented when compared to late working-age adults within patients with social phobia, specific phobias, GAD, obsessive compulsive disorder, somatization disorder, substance use disorders, and psychotic disorders. Noteworthy, the opposite pattern was observed among patients with pain disorders: here, underrepresentation of older adults was strongest when compared to later working-age subgroups than when compared to early working-age adults. The complete results are given in the Supplementary Material (Supplement B, Table S5).

Gender-specific analyses revealed comparable levels of underrepresentation among male and female patients. Underrepresentation was similar for male (RQ = 0.25, 95%-CI = [0.20; 0.30]) and female (RQ = 0.26, 95%-CI = [0.22; 0.29]) young-old adults, as well as for male (RQ = 0.07, 95%-CI = [0.05; 0.11]) and female (RQ = 0.07, 95%-CI = [0.05; 0.08]) old-old adults. Detailed results for individual mental disorders are given in the Supplementary Material (Supplement B, Table S4).

### Sensitivity analyses 

#### Prevalence assumptions

When repeating the analyses assuming equal prevalences across age groups, the estimated underrepresentation aggravated both for young-old (RQ = 0.18, 95%-CI = [0.16; 0.20]) and old-old adults (RQ = 0.05, 95%-CI = [0.04; 0.06]). When repeating the analyses while assuming considerably lower prevalences of mental disorders in old-old compared to young-old adults (prevalence ratio = 0.5), the differences in estimates of underrepresentation between young-old and old-old adults were reduced. However, the global results still indicated more severe underrepresentation of old-old adults (RQ = 0.13, 95%-CI = [0.11; 0.16]) compared to young-old adults (RQ = 0.24, 95%-CI = [0.22; 0.27]). The complete results of sensitivity analyses for varying prevalence assumptions for individual disorders are presented in the Supplementary Material (Supplement B, Table S6 and S7).

#### Long-term care needs

Removing people with long-term care needs from the reference population had minimal impact on the results for young-old adults, who remained significantly underrepresented (RQ = 0.26, 95%-CI = [0.23; 0.29]). For old-old adults, however, removing individuals with long-term care needs slightly reduced the extent of underrepresentation but still indicated substantial discrepancies (RQ = 0.10, 95%-CI = [0.08; 0.12]). The results for individual disorders are given in the Supplementary Material (Supplement B, Table S8).

## Discussion

This study provides the first large-scale quantification of age-related disparities in psychotherapy utilization within university outpatient clinics in Germany. Whereas previous research on the representation of older adults among psychotherapy patients in Germany has largely been limited to descriptive figures [36–39, 51], the present study is the first to systematically apply a representation quotient approach to formally identify and quantify underrepresentation of older adults. By comparing the observed age distribution of psychotherapy patients with a reference population, we obtained interpretable estimates of representation. In doing so, our study demonstrates the practical utility of representation quotients for formally identifying and quantifying underrepresentation in routine care data. Moreover, the study provides substantive estimates of underrepresentation of older adults in university outpatient clinics. Although these clinics are not representative of the entire psychotherapeutic landscape, they constitute a key component of the outpatient mental healthcare system in Germany [42, 43]. Consequently, the results offer valuable, quantitative insights into age-related disparities in psychotherapy utilization within this specific care setting.

Despite universal coverage for psychotherapeutic care under the German statutory health insurance system, we found a marked underrepresentation of older adults. Under all prevalence assumptions, young-old adults accounted only for about 25% of their expected proportion (all RQs ≤ 0.26), while old-old adults accounted for no more than 15% of their anticipated number (all RQs ≤ 0.13). These findings highlight a substantial gap between the mental healthcare needs of older adults and their actual utilization of psychotherapeutic services, even in settings that are publicly funded, academically affiliated, and structurally integrated into the healthcare system. The results indicate that to achieve equity in service provision, the proportion of treated younger-old adults would need to be increased by at least a factor of four, and for older-old adults increased by at least a factor of seven.

Representation quotients in all individual mental disorder subgroups indicated strong underrepresentation, with a notable exception for pain disorders. Older adults were most strongly underrepresented among patients with PTSD, eating disorders, and dysthymia. In contrast, representation was comparatively higher among older patients with GAD. This pattern is particularly noteworthy as PTSD, eating disorders, and dysthymia have been previously identified as underrecognized and inadequately treated in older adults [66–68]. By contrast, GAD is not only considered especially prevalent in later life but is also the most extensively studied anxiety disorder in older populations [14, 69]. These findings suggest that disorders commonly, yet incorrectly, assumed to be of limited clinical relevance in older age, are also those for which older adults are most underrepresented in psychotherapy. This highlights the need to correct such misconceptions and ensure that research and practice adequately address overlooked conditions.

The relatively high representation of older adults with pain disorders may point to a key pathway through which older individuals access the mental healthcare system. Prior research has shown that somatic comorbidities are significant predictors of mental health service utilization among older adults with depression, potentially due to more frequent interactions with general practitioners [70]. Accordingly, older adults presenting with primarily somatic symptoms, such as those associated with pain disorders, may have an increased likelihood of being referred to mental healthcare compared to those with predominantly psychological complaints [71]. These findings underscore the importance of understanding how older adults typically access mental healthcare and of paying particular attention to the role of other healthcare providers. Such insights can in turn inform outreach strategies that align with healthcare-seeking behaviors of older adults.

We found no indication of gender differences in underrepresentation of older adults. This finding warrants contextualization as it may seem to contradict evidence that male gender is a risk factor for non-utilization of mental health services in general [35, 72], and also among older adults [73, 74]. However, those studies indicate a main effect of male gender on non-utilization, while significant gender differences in our results would imply an interaction effect of old age and male gender, which was not found here. Thus, both male and female older adults appear to be similarly underrepresented compared with male and female working-age adults. Nevertheless, old age and male gender represent two distinct risk factors for non-utilization, making older men an important target group for initiatives aimed at increasing access to psychotherapeutic care.

Compared to previous studies on the representation of older adults in outpatient psychotherapy in Germany, the proportion of older adults in our sample was even lower, although direct comparisons are complicated by heterogenous age group definitions [37–39]. For example, while about 13% of patients in a large study based on health insurance data were 60 years or older [39], this group accounted for only 7.3% in our sample. This difference likely reflects the specific setting of our study in university outpatient clinics. These clinics, typically located in academic and urban environments, may particularly attract younger and more mobile populations. Fittingly, when comparing older adults to subgroups of working-age adults, we found that representation gradually decreased with increasing age. A gradual decline in psychotherapy utilization with age is a well-established finding [22, 35], and our results are largely consistent with this pattern. However, the age group distribution in our sample was particularly skewed: Compared to previous studies on routine outpatient psychotherapy in Germany, young working-age adults (18–34 years) were markedly overrepresented in our sample [75–77]. Specifically, young working-age adults accounted for 55.9% of our sample, while they made up 24.3% of the sample of a large quality assurance project on outpatient psychotherapy in Germany (*N* = 22,294) [75, 76]. Hence, the lower share of older adults observed in our data likely results in part from this disproportionate representation of young working-age patients. Importantly, however, older adults remained significantly underrepresented even when compared only to middle and late working-age adults. Therefore, the observed underrepresentation relative to the overall working-age group cannot be attributed solely to the overrepresentation of younger adults but also reflects a distinct pattern of underrepresentation of older adults.

### Clinical and research implications

Overall, our results demonstrate a substantial underrepresentation of older adults among outpatient psychotherapy patients in Germany, highlighting the need for targeted initiatives to increase help-seeking and treatment utilization within this group. Previous research has identified several key barriers to psychotherapy utilization among older adults. At the patient level, difficulties in recognizing the need for treatment and the tendency to normalize psychopathological symptoms as part of aging have been noted as major obstacles [78–81]. Additionally, internalized stigma rooted in historically grounded values of self-reliance, as well as concerns about social judgment, can further hinder help-seeking behavior [82–84]. In this context, initiatives aimed at improving mental health literacy and promoting help-seeking behavior among older adults appear particularly promising [85, 86].

In addition to patient-related factors, specific barriers within the healthcare system also contribute to the underrepresentation of older adults in psychotherapy. As noted above, referral pathways play a crucial role in this context. Compared to younger patients, older adults rely more heavily on general practitioners (GPs) for access to mental health services. However, GPs have been found to refer older adults with mental disorders to psychotherapeutic services less frequently than younger patients [87–89]. Limited time resources in primary care settings further constrain opportunities for adequate referral practices [84, 89]. Strengthening the education of GPs regarding mental health in older adults and non-pharmacological treatments, as well as fostering closer collaboration between GPs and psychotherapists, could therefore be important strategies to improve utilization. Moreover, barriers may also exist at the level of psychotherapists themselves, who frequently report age-related stereotypes and low perceived competence in treating older patients [90, 91]. Promoting critical reflection on age-related biases and providing training in age-augmented psychotherapy thus appears essential [92, 93].

Finally, practical and structural barriers may also impede psychotherapy utilization among older adults. While common barriers in international contexts related to cost and insurance [82, 83, 94] are largely irrelevant in the German statutory healthcare system, issues such as transportation and accessibility may still pose significant challenges, particularly for older adults living in rural areas with limited mobility [78, 83, 94]. In this regard, expanding transportation services tailored to the needs of older adults could facilitate access. Moreover, greater emphasis on outreach and telehealth interventions may offer additional solutions [64, 95]. Specific living arrangements and responsibilities, such as providing informal care for a spouse, may further restrict the ability to attend regular psychotherapy. For example, teleinterventions for family caregivers, which have been shown to be both feasible and effective for older adults, represent a promising approach and should be integrated into routine care [96–98].

Initiatives aimed at improving psychotherapy utilization among older adults in Germany should address barriers at the patient, provider, and structural levels as outlined above. University outpatient clinics should be closely involved in these efforts given their high patient volumes, their role in training future psychotherapists, and their potential to serve as models for innovative care. We believe that our results are a representative snapshot of older adults seeking psychotherapy in university outpatient clinics in Germany, between 2018 and 2023, and can thus be used as benchmarks to evaluate changes in representation in the future.

## Limitations

The findings of our study should be interpreted in light of several limitations. First, our representation quotient analyses were designed to quantify the relative underutilization of psychotherapy among older compared to working-age adults. However, these coefficients reflect relative discrepancies and should not be taken to imply that mental healthcare for working-age adults in Germany is without significant shortcomings. Substantial gaps and barriers to utilization also exist within younger populations [99], and our findings do not suggest that their care needs are fully met.

Second, the representation estimates rely heavily on the underlying assumptions regarding prevalence rates of mental disorders. As the population-based prevalence study that served as our primary reference did not differentiate between young-old and old-old adults, we had to either assume equal prevalence rates across these subgroups or apply age-specific prevalence ratios that are not yet well-established empirically. Consequently, the precise estimates of representation should be interpreted with these uncertainties in mind. Future epidemiological studies should provide more granular, age-differentiated prevalence data for older populations.

Third, alternative explanations for the observed underrepresentation of older adults may exist that are not related to utilization barriers or insufficient care provision. Older adults might rely more heavily on other services, such as specialized geriatric inpatient care, or may have completed one or more courses of psychotherapy earlier in life, reducing the likelihood of seeking further treatment. Differences in representation between disorders could also partly reflect these factors. For instance, eating disorders, typically characterized by early onset, may be more extensively treated during younger years, resulting in fewer older adults requiring treatment later in life. However, given the substantial underrepresentation observed in our study, it seems unlikely that such factors alone account for the discrepancy.

Fourth, our sample exclusively included CBT patients. Thus, the generalizability of our findings to other psychotherapeutic modalities is unclear, as it is possible that representation of older adults differs across treatment modalities. For example, available evidence indicates that older adults may be even less represented in psychodynamic and psychoanalytic treatments in Germany [37]. Therefore, our findings should be interpreted as reflecting the context of CBT, with some caution required when generalizing to other forms of psychotherapy.

Fifth, our study does not identify specific barriers contributing to the underrepresentation of older adults. This limitation is inherent in the nature of the dataset, which included only individuals who have already accessed psychotherapy. As a result, the analysis of specific utilization barriers remains beyond the scope of this study.

## Conclusion

This study is the first to quantify age-related disparities in psychotherapy utilization in Germany using a representation quotient approach. By applying this methodology to a large routine dataset from 29 university outpatient clinics, we demonstrate its utility for deriving interpretable estimates of underrepresentation compared to a reference population constructed under transparent and explicitly defined assumptions. The results indicate an underrepresentation of young-old and old-old adults among the investigated sample, which is concerning given the growing mental healthcare needs of an aging population. Due to their patient volumes, their impact through educating the next generation of psychotherapists, and their potential to serve as models for innovative care, university clinics could play a crucial role in the process of enhancing psychotherapy uptake among older adults. Concrete steps may include targeted outreach to older adults, strengthening cooperations with general practitioners, training psychotherapists in age-augmented therapy, and expanding age-adapted service models.

## Electronic supplementary material

Below is the link to the electronic supplementary material.

**Supplementary Material 1:** Statistical Code File

**Supplementary Material 2:** Supplement A

**Supplementary Material 3:** Supplement B

## Data Availability

All statistical input and output code is given in the Supplementary Material. The dataset analyzed during the current study is available from the authors on reasonable request.
