# Supplementary material for: Quantifying age-related disparities in outpatient psychotherapy utilization: a representation quotient analysis of routine data from 29 university clinics in Germany
Source: BMC Health Serv Res. 2025 Nov 25;25:1558. doi: 10.1186/s12913-025-13714-5 (PMC12670761; doi:10.1186/s12913-025-13714-5)
Supplement: Supplementary file 1 — Supplementary Material 1: Statistical Code File [file 12913_2025_13714_MOESM1_ESM.html]

Quantifying age-related disparities in outpatient psychotherapy utilization: A representation quotient analysis of routine data from 29 university clinics in Germany


# Quantifying age-related disparities in outpatient psychotherapy utilization: A representation quotient analysis of routine data from 29 university clinics in Germany

# Representation quotient analysis

## Data preparation

### Preparation of dataset

```
library(haven)
library(mice)
library(miceadds)
library(lmtest)
library(sandwich)
library(psych)
library(foreign)
library(car)
library(mice)
library(miceadds)
library(dplyr)
library(labeling)
library(MatchThem)
library(MatchIt)
library(cobalt)
library(robustbase)
library(broom)
library(estimatr)
library(reshape)
library(reshape2)
library(ggplot2)
library(readr)
library(mitools)
library(mice) 
library(miceadds)
library(cli)
library(haven)
library(MASS)
library(robustbase)
library(ggmice)
library(nnet)
library(readxl)

#Load dataset
KODAP_data_complete <- read_sav("2025-02-17_KODAP_PP_2018-2023_Wrede_Jena.sav")

length(KODAP_data_complete$Patient_ID)
```

```
## [1] 22381
```

```
#Exclude duplicate patients
KODAP_data_complete <- KODAP_data_complete[!duplicated(KODAP_data_complete$Patient_ID) & !duplicated(KODAP_data_complete$Patient_ID, fromLast = TRUE), ]
#Exclude patients < 18 year
KODAP_data_complete <- subset(KODAP_data_complete, Pat_Alter >= 18)
#Only include CBT treatments
KODAP_data_complete <- subset(KODAP_data_complete, Ther_Verfahren == 1)

#Add NAs to diagnosis variables
KODAP_data_complete$ICD1_pre_clean <- ifelse(KODAP_data_complete$ICD1_pre_clean == "" | KODAP_data_complete$ICD1_pre_clean == "-99",NA,KODAP_data_complete$ICD1_pre_clean)
KODAP_data_complete$ICD2_pre_clean <- ifelse(KODAP_data_complete$ICD2_pre_clean == "" | KODAP_data_complete$ICD2_pre_clean == "-99",NA,KODAP_data_complete$ICD2_pre_clean)
KODAP_data_complete$ICD3_pre_clean <- ifelse(KODAP_data_complete$ICD3_pre_clean == "" | KODAP_data_complete$ICD3_pre_clean == "-99",NA,KODAP_data_complete$ICD3_pre_clean)
KODAP_data_complete$ICD4_pre_clean <- ifelse(KODAP_data_complete$ICD4_pre_clean == "" | KODAP_data_complete$ICD4_pre_clean == "-99",NA,KODAP_data_complete$ICD4_pre_clean)
KODAP_data_complete$ICD5_pre_clean <- ifelse(KODAP_data_complete$ICD5_pre_clean == "" | KODAP_data_complete$ICD5_pre_clean == "-99",NA,KODAP_data_complete$ICD5_pre_clean)

#Exclude treatments that were not reimbursed by health insurances
table(KODAP_data_complete$Abschluss)
```

```
## 
##     0     1     2     3     4 
##  6453 10155  2345    41    67
```

```
KODAP_data_complete <- subset(KODAP_data_complete, Abschluss %in% c(0,1,2,4))
length(KODAP_data_complete$Patient_ID)
```

```
## [1] 19020
```

```
#Only include treatments between 2018 and 2023
pss2date <- function(x) as.Date(x/86400, origin = "1582-10-14")
KODAP_data_complete$Therapie_pre <- pss2date(KODAP_data_complete$Therapie_pre)
KODAP_data_complete$Beginn_Therapie <- substr(KODAP_data_complete$Therapie_pre, 1, 4)
table(KODAP_data_complete$Beginn_Therapie)
```

```
## 
## 2013 2014 2015 2016 2017 2018 2019 2020 2021 2022 2023 2024 
##    7   10   21   79  478 2231 2938 3056 2990 2977 2886  461
```

```
KODAP_data_complete <- subset(KODAP_data_complete, Beginn_Therapie %in% c("2018", "2019", "2020", "2021", "2022", "2023"))
length(KODAP_data_complete$Patient_ID)
```

```
## [1] 17078
```

```
length(unique(KODAP_data_complete$Ambulanz_ID))
```

```
## [1] 30
```

```
table(KODAP_data_complete$Beginn_Therapie)
```

```
## 
## 2018 2019 2020 2021 2022 2023 
## 2231 2938 3056 2990 2977 2886
```

```
table(KODAP_data_complete$Beginn_Therapie)/length(KODAP_data_complete$Patient_ID)*100
```

```
## 
##     2018     2019     2020     2021     2022     2023 
## 13.06359 17.20342 17.89437 17.50790 17.43178 16.89893
```

```
KODAP_data_complete$Age_Stepped_bin <- ifelse(KODAP_data_complete$Pat_Alter %in% c(18:64), 0,
                                              ifelse(KODAP_data_complete$Pat_Alter %in% c(65:100), 1,NA))

KODAP_data_complete$Age_Stepped <- ifelse(KODAP_data_complete$Pat_Alter %in% c(18:64), 0,
                                          ifelse(KODAP_data_complete$Pat_Alter %in% c(65:74), 1,
                                                 ifelse(KODAP_data_complete$Pat_Alter %in% c(75:100), 2,NA)))


KODAP_data_complete$Age_Stepped_2 <- ifelse(KODAP_data_complete$Pat_Alter %in% c(18:34), 0,
                                            ifelse(KODAP_data_complete$Pat_Alter %in% c(35:49), 1,
                                                   ifelse(KODAP_data_complete$Pat_Alter %in% c(50:64), 2,
                                                          ifelse(KODAP_data_complete$Pat_Alter %in% c(65:74), 3,
                                                                 ifelse(KODAP_data_complete$Pat_Alter %in% c(75:100), 4,NA)))))


table(KODAP_data_complete$Age_Stepped)
```

```
## 
##     0     1     2 
## 16558   405   115
```

```
KODAP_data_complete <- subset(KODAP_data_complete, Art_Diagnoseerhebung_pre %in% c(1,2,3))
length(KODAP_data_complete$Patient_ID)
```

```
## [1] 15915
```

```
KODAP_data_complete$Missing_diagnosis <- ifelse(is.na(KODAP_data_complete$ICD1_pre_clean) == TRUE & 
                                                  is.na(KODAP_data_complete$ICD2_pre_clean) == TRUE & 
                                                  is.na(KODAP_data_complete$ICD3_pre_clean) == TRUE & 
                                                  is.na(KODAP_data_complete$ICD4_pre_clean) == TRUE & 
                                                  is.na(KODAP_data_complete$ICD5_pre_clean) == TRUE, 1,0)

KODAP_data_complete <- subset(KODAP_data_complete, Missing_diagnosis == 0)
length(KODAP_data_complete$Patient_ID)
```

```
## [1] 13635
```

### Preparation of census data

```
#Age Distibution Germany
Census_data <- read_delim("15_bevoelkerungsvorausberechnung_daten.csv", 
                          delim = ";", escape_double = FALSE, trim_ws = TRUE)
View(Census_data)

census_amount_1864_2018 <- sum(Census_data[Census_data$Simulationsjahr == 2018, c(23:69)])/sum(Census_data[Census_data$Simulationsjahr == 2018, c(23:104)])
census_amount_1864_2019 <- sum(Census_data[Census_data$Simulationsjahr == 2019, c(23:69)])/sum(Census_data[Census_data$Simulationsjahr == 2019, c(23:104)])
census_amount_1864_2020 <- sum(Census_data[Census_data$Simulationsjahr == 2020, c(23:69)])/sum(Census_data[Census_data$Simulationsjahr == 2020, c(23:104)])
census_amount_1864_2021 <- sum(Census_data[Census_data$Simulationsjahr == 2021, c(23:69)])/sum(Census_data[Census_data$Simulationsjahr == 2021, c(23:104)])
census_amount_1864_2022 <- sum(Census_data[Census_data$Simulationsjahr == 2022 & Census_data$Variante == 1, c(23:69)])/sum(Census_data[Census_data$Simulationsjahr == 2022 & Census_data$Variante == 1, c(23:104)])
census_amount_1864_2023 <- sum(Census_data[Census_data$Simulationsjahr == 2023 & Census_data$Variante == 1, c(23:69)])/sum(Census_data[Census_data$Simulationsjahr == 2023 & Census_data$Variante == 1, c(23:104)])

census_amount_1834_2018 <- sum(Census_data[Census_data$Simulationsjahr == 2018, c(23:39)])/sum(Census_data[Census_data$Simulationsjahr == 2018, c(23:104)])
census_amount_1834_2019 <- sum(Census_data[Census_data$Simulationsjahr == 2019, c(23:39)])/sum(Census_data[Census_data$Simulationsjahr == 2019, c(23:104)])
census_amount_1834_2020 <- sum(Census_data[Census_data$Simulationsjahr == 2020, c(23:39)])/sum(Census_data[Census_data$Simulationsjahr == 2020, c(23:104)])
census_amount_1834_2021 <- sum(Census_data[Census_data$Simulationsjahr == 2021, c(23:39)])/sum(Census_data[Census_data$Simulationsjahr == 2021, c(23:104)])
census_amount_1834_2022 <- sum(Census_data[Census_data$Simulationsjahr == 2022 & Census_data$Variante == 1, c(23:39)])/sum(Census_data[Census_data$Simulationsjahr == 2022 & Census_data$Variante == 1, c(23:104)])
census_amount_1834_2023 <- sum(Census_data[Census_data$Simulationsjahr == 2023 & Census_data$Variante == 1, c(23:39)])/sum(Census_data[Census_data$Simulationsjahr == 2023 & Census_data$Variante == 1, c(23:104)])

census_amount_3549_2018 <- sum(Census_data[Census_data$Simulationsjahr == 2018, c(40:54)])/sum(Census_data[Census_data$Simulationsjahr == 2018, c(23:104)])
census_amount_3549_2019 <- sum(Census_data[Census_data$Simulationsjahr == 2019, c(40:54)])/sum(Census_data[Census_data$Simulationsjahr == 2019, c(23:104)])
census_amount_3549_2020 <- sum(Census_data[Census_data$Simulationsjahr == 2020, c(40:54)])/sum(Census_data[Census_data$Simulationsjahr == 2020, c(23:104)])
census_amount_3549_2021 <- sum(Census_data[Census_data$Simulationsjahr == 2021, c(40:54)])/sum(Census_data[Census_data$Simulationsjahr == 2021, c(23:104)])
census_amount_3549_2022 <- sum(Census_data[Census_data$Simulationsjahr == 2022 & Census_data$Variante == 1, c(40:54)])/sum(Census_data[Census_data$Simulationsjahr == 2022 & Census_data$Variante == 1, c(23:104)])
census_amount_3549_2023 <- sum(Census_data[Census_data$Simulationsjahr == 2023 & Census_data$Variante == 1, c(40:54)])/sum(Census_data[Census_data$Simulationsjahr == 2023 & Census_data$Variante == 1, c(23:104)])

census_amount_5064_2018 <- sum(Census_data[Census_data$Simulationsjahr == 2018, c(55:69)])/sum(Census_data[Census_data$Simulationsjahr == 2018, c(23:104)])
census_amount_5064_2019 <- sum(Census_data[Census_data$Simulationsjahr == 2019, c(55:69)])/sum(Census_data[Census_data$Simulationsjahr == 2019, c(23:104)])
census_amount_5064_2020 <- sum(Census_data[Census_data$Simulationsjahr == 2020, c(55:69)])/sum(Census_data[Census_data$Simulationsjahr == 2020, c(23:104)])
census_amount_5064_2021 <- sum(Census_data[Census_data$Simulationsjahr == 2021, c(55:69)])/sum(Census_data[Census_data$Simulationsjahr == 2021, c(23:104)])
census_amount_5064_2022 <- sum(Census_data[Census_data$Simulationsjahr == 2022 & Census_data$Variante == 1, c(55:69)])/sum(Census_data[Census_data$Simulationsjahr == 2022 & Census_data$Variante == 1, c(23:104)])
census_amount_5064_2023 <- sum(Census_data[Census_data$Simulationsjahr == 2023 & Census_data$Variante == 1, c(55:69)])/sum(Census_data[Census_data$Simulationsjahr == 2023 & Census_data$Variante == 1, c(23:104)])

census_amount_6574_2018 <- sum(Census_data[Census_data$Simulationsjahr == 2018, c(70:79)])/sum(Census_data[Census_data$Simulationsjahr == 2018, c(23:104)])
census_amount_6574_2019 <- sum(Census_data[Census_data$Simulationsjahr == 2019, c(70:79)])/sum(Census_data[Census_data$Simulationsjahr == 2019, c(23:104)])
census_amount_6574_2020 <- sum(Census_data[Census_data$Simulationsjahr == 2020, c(70:79)])/sum(Census_data[Census_data$Simulationsjahr == 2020, c(23:104)])
census_amount_6574_2021 <- sum(Census_data[Census_data$Simulationsjahr == 2021, c(70:79)])/sum(Census_data[Census_data$Simulationsjahr == 2021, c(23:104)])
census_amount_6574_2022 <- sum(Census_data[Census_data$Simulationsjahr == 2022 & Census_data$Variante == 1, c(70:79)])/sum(Census_data[Census_data$Simulationsjahr == 2022 & Census_data$Variante == 1, c(23:104)])
census_amount_6574_2023 <- sum(Census_data[Census_data$Simulationsjahr == 2023 & Census_data$Variante == 1, c(70:79)])/sum(Census_data[Census_data$Simulationsjahr == 2023 & Census_data$Variante == 1, c(23:104)])

census_amount_75plus_2018 <- sum(Census_data[Census_data$Simulationsjahr == 2018, c(80:104)])/sum(Census_data[Census_data$Simulationsjahr == 2018, c(23:104)])
census_amount_75plus_2019 <- sum(Census_data[Census_data$Simulationsjahr == 2019, c(80:104)])/sum(Census_data[Census_data$Simulationsjahr == 2019, c(23:104)])
census_amount_75plus_2020 <- sum(Census_data[Census_data$Simulationsjahr == 2020, c(80:104)])/sum(Census_data[Census_data$Simulationsjahr == 2020, c(23:104)])
census_amount_75plus_2021 <- sum(Census_data[Census_data$Simulationsjahr == 2021, c(80:104)])/sum(Census_data[Census_data$Simulationsjahr == 2021, c(23:104)])
census_amount_75plus_2022 <- sum(Census_data[Census_data$Simulationsjahr == 2022 & Census_data$Variante == 1, c(80:104)])/sum(Census_data[Census_data$Simulationsjahr == 2022 & Census_data$Variante == 1, c(23:104)])
census_amount_75plus_2023 <- sum(Census_data[Census_data$Simulationsjahr == 2023 & Census_data$Variante == 1, c(80:104)])/sum(Census_data[Census_data$Simulationsjahr == 2023 & Census_data$Variante == 1, c(23:104)])

census_amount_65plus_2018 <- sum(Census_data[Census_data$Simulationsjahr == 2018, c(70:104)])/sum(Census_data[Census_data$Simulationsjahr == 2018, c(23:104)])
census_amount_65plus_2019 <- sum(Census_data[Census_data$Simulationsjahr == 2019, c(70:104)])/sum(Census_data[Census_data$Simulationsjahr == 2019, c(23:104)])
census_amount_65plus_2020 <- sum(Census_data[Census_data$Simulationsjahr == 2020, c(70:104)])/sum(Census_data[Census_data$Simulationsjahr == 2020, c(23:104)])
census_amount_65plus_2021 <- sum(Census_data[Census_data$Simulationsjahr == 2021, c(70:104)])/sum(Census_data[Census_data$Simulationsjahr == 2021, c(23:104)])
census_amount_65plus_2022 <- sum(Census_data[Census_data$Simulationsjahr == 2022 & Census_data$Variante == 1, c(70:104)])/sum(Census_data[Census_data$Simulationsjahr == 2022 & Census_data$Variante == 1, c(23:104)])
census_amount_65plus_2023 <- sum(Census_data[Census_data$Simulationsjahr == 2023 & Census_data$Variante == 1, c(70:104)])/sum(Census_data[Census_data$Simulationsjahr == 2023 & Census_data$Variante == 1, c(23:104)])

census_amount_6569_2018 <- sum(Census_data[Census_data$Simulationsjahr == 2018, c(70:74)])/sum(Census_data[Census_data$Simulationsjahr == 2018, c(23:104)])
census_amount_6569_2019 <- sum(Census_data[Census_data$Simulationsjahr == 2019, c(70:74)])/sum(Census_data[Census_data$Simulationsjahr == 2019, c(23:104)])
census_amount_6569_2020 <- sum(Census_data[Census_data$Simulationsjahr == 2020, c(70:74)])/sum(Census_data[Census_data$Simulationsjahr == 2020, c(23:104)])
census_amount_6569_2021 <- sum(Census_data[Census_data$Simulationsjahr == 2021, c(70:74)])/sum(Census_data[Census_data$Simulationsjahr == 2021, c(23:104)])
census_amount_6569_2022 <- sum(Census_data[Census_data$Simulationsjahr == 2022 & Census_data$Variante == 1, c(70:74)])/sum(Census_data[Census_data$Simulationsjahr == 2022 & Census_data$Variante == 1, c(23:104)])
census_amount_6569_2023 <- sum(Census_data[Census_data$Simulationsjahr == 2023 & Census_data$Variante == 1, c(70:74)])/sum(Census_data[Census_data$Simulationsjahr == 2023 & Census_data$Variante == 1, c(23:104)])

census_amount_7074_2018 <- sum(Census_data[Census_data$Simulationsjahr == 2018, c(75:79)])/sum(Census_data[Census_data$Simulationsjahr == 2018, c(23:104)])
census_amount_7074_2019 <- sum(Census_data[Census_data$Simulationsjahr == 2019, c(75:79)])/sum(Census_data[Census_data$Simulationsjahr == 2019, c(23:104)])
census_amount_7074_2020 <- sum(Census_data[Census_data$Simulationsjahr == 2020, c(75:79)])/sum(Census_data[Census_data$Simulationsjahr == 2020, c(23:104)])
census_amount_7074_2021 <- sum(Census_data[Census_data$Simulationsjahr == 2021, c(75:79)])/sum(Census_data[Census_data$Simulationsjahr == 2021, c(23:104)])
census_amount_7074_2022 <- sum(Census_data[Census_data$Simulationsjahr == 2022 & Census_data$Variante == 1, c(75:79)])/sum(Census_data[Census_data$Simulationsjahr == 2022 & Census_data$Variante == 1, c(23:104)])
census_amount_7074_2023 <- sum(Census_data[Census_data$Simulationsjahr == 2023 & Census_data$Variante == 1, c(75:79)])/sum(Census_data[Census_data$Simulationsjahr == 2023 & Census_data$Variante == 1, c(23:104)])

census_amount_7579_2018 <- sum(Census_data[Census_data$Simulationsjahr == 2018, c(80:84)])/sum(Census_data[Census_data$Simulationsjahr == 2018, c(23:104)])
census_amount_7579_2019 <- sum(Census_data[Census_data$Simulationsjahr == 2019, c(80:84)])/sum(Census_data[Census_data$Simulationsjahr == 2019, c(23:104)])
census_amount_7579_2020 <- sum(Census_data[Census_data$Simulationsjahr == 2020, c(80:84)])/sum(Census_data[Census_data$Simulationsjahr == 2020, c(23:104)])
census_amount_7579_2021 <- sum(Census_data[Census_data$Simulationsjahr == 2021, c(80:84)])/sum(Census_data[Census_data$Simulationsjahr == 2021, c(23:104)])
census_amount_7579_2022 <- sum(Census_data[Census_data$Simulationsjahr == 2022 & Census_data$Variante == 1, c(80:84)])/sum(Census_data[Census_data$Simulationsjahr == 2022 & Census_data$Variante == 1, c(23:104)])
census_amount_7579_2023 <- sum(Census_data[Census_data$Simulationsjahr == 2023 & Census_data$Variante == 1, c(80:84)])/sum(Census_data[Census_data$Simulationsjahr == 2023 & Census_data$Variante == 1, c(23:104)])

census_amount_80plus_2018 <- sum(Census_data[Census_data$Simulationsjahr == 2018, c(85:104)])/sum(Census_data[Census_data$Simulationsjahr == 2018, c(23:104)])
census_amount_80plus_2019 <- sum(Census_data[Census_data$Simulationsjahr == 2019, c(85:104)])/sum(Census_data[Census_data$Simulationsjahr == 2019, c(23:104)])
census_amount_80plus_2020 <- sum(Census_data[Census_data$Simulationsjahr == 2020, c(85:104)])/sum(Census_data[Census_data$Simulationsjahr == 2020, c(23:104)])
census_amount_80plus_2021 <- sum(Census_data[Census_data$Simulationsjahr == 2021, c(85:104)])/sum(Census_data[Census_data$Simulationsjahr == 2021, c(23:104)])
census_amount_80plus_2022 <- sum(Census_data[Census_data$Simulationsjahr == 2022 & Census_data$Variante == 1, c(85:104)])/sum(Census_data[Census_data$Simulationsjahr == 2022 & Census_data$Variante == 1, c(23:104)])
census_amount_80plus_2023 <- sum(Census_data[Census_data$Simulationsjahr == 2023 & Census_data$Variante == 1, c(85:104)])/sum(Census_data[Census_data$Simulationsjahr == 2023 & Census_data$Variante == 1, c(23:104)])

year_distribution_data <- as.data.frame(table(KODAP_data_complete$Beginn_Therapie))
year_distribution_data
```

```
##   Var1 Freq
## 1 2018 2095
## 2 2019 2625
## 3 2020 2432
## 4 2021 2410
## 5 2022 2255
## 6 2023 1818
```

```
#Computing dataset specific census amounts: 
census_amount_1864 <- (2095/13635)*census_amount_1864_2018+(2625/13635)*census_amount_1864_2019+(2432/13635)*census_amount_1864_2020+(2410/13635)*census_amount_1864_2021+(2255/13635)*census_amount_1864_2022+(1818/13635)*census_amount_1864_2023

census_amount_1834 <- (2095/13635)*census_amount_1834_2018+(2625/13635)*census_amount_1834_2019+(2432/13635)*census_amount_1834_2020+(2410/13635)*census_amount_1834_2021+(2255/13635)*census_amount_1834_2022+(1818/13635)*census_amount_1834_2023

census_amount_3549 <- (2095/13635)*census_amount_3549_2018+(2625/13635)*census_amount_3549_2019+(2432/13635)*census_amount_3549_2020+(2410/13635)*census_amount_3549_2021+(2255/13635)*census_amount_3549_2022+(1818/13635)*census_amount_3549_2023

census_amount_5064 <- (2095/13635)*census_amount_5064_2018+(2625/13635)*census_amount_5064_2019+(2432/13635)*census_amount_5064_2020+(2410/13635)*census_amount_5064_2021+(2255/13635)*census_amount_5064_2022+(1818/13635)*census_amount_5064_2023

census_amount_6574 <- (2095/13635)*census_amount_6574_2018+(2625/13635)*census_amount_6574_2019+(2432/13635)*census_amount_6574_2020+(2410/13635)*census_amount_6574_2021+(2255/13635)*census_amount_6574_2022+(1818/13635)*census_amount_6574_2023

census_amount_75plus <- (2095/13635)*census_amount_75plus_2018+(2625/13635)*census_amount_75plus_2019+(2432/13635)*census_amount_75plus_2020+(2410/13635)*census_amount_75plus_2021+(2255/13635)*census_amount_75plus_2022+(1818/13635)*census_amount_75plus_2023

census_amount_65plus <- (2095/13635)*census_amount_65plus_2018+(2625/13635)*census_amount_65plus_2019+(2432/13635)*census_amount_65plus_2020+(2410/13635)*census_amount_65plus_2021+(2255/13635)*census_amount_65plus_2022+(1818/13635)*census_amount_65plus_2023

census_amount_6569 <- (2095/13635)*census_amount_6569_2018+(2625/13635)*census_amount_6569_2019+(2432/13635)*census_amount_6569_2020+(2410/13635)*census_amount_6569_2021+(2255/13635)*census_amount_6569_2022+(1818/13635)*census_amount_6569_2023
census_amount_7074 <- (2095/13635)*census_amount_7074_2018+(2625/13635)*census_amount_7074_2019+(2432/13635)*census_amount_7074_2020+(2410/13635)*census_amount_7074_2021+(2255/13635)*census_amount_7074_2022+(1818/13635)*census_amount_7074_2023
census_amount_7579 <- (2095/13635)*census_amount_7579_2018+(2625/13635)*census_amount_7579_2019+(2432/13635)*census_amount_7579_2020+(2410/13635)*census_amount_7579_2021+(2255/13635)*census_amount_7579_2022+(1818/13635)*census_amount_7579_2023
census_amount_80plus <- (2095/13635)*census_amount_80plus_2018+(2625/13635)*census_amount_80plus_2019+(2432/13635)*census_amount_80plus_2020+(2410/13635)*census_amount_80plus_2021+(2255/13635)*census_amount_80plus_2022+(1818/13635)*census_amount_80plus_2023


#Female only
female_amount_1864_2018 <- sum(Census_data[Census_data$Simulationsjahr == 2018 & Census_data$mw == "w", c(23:69)])/sum(Census_data[Census_data$Simulationsjahr == 2018, c(23:104)])
female_amount_1864_2019 <- sum(Census_data[Census_data$Simulationsjahr == 2019 & Census_data$mw == "w", c(23:69)])/sum(Census_data[Census_data$Simulationsjahr == 2019, c(23:104)])
female_amount_1864_2020 <- sum(Census_data[Census_data$Simulationsjahr == 2020 & Census_data$mw == "w", c(23:69)])/sum(Census_data[Census_data$Simulationsjahr == 2020, c(23:104)])
female_amount_1864_2021 <- sum(Census_data[Census_data$Simulationsjahr == 2021 & Census_data$mw == "w", c(23:69)])/sum(Census_data[Census_data$Simulationsjahr == 2021, c(23:104)])
female_amount_1864_2022 <- sum(Census_data[Census_data$Simulationsjahr == 2022 & Census_data$mw == "w" & Census_data$Variante == 1, c(23:69)])/sum(Census_data[Census_data$Simulationsjahr == 2022 & Census_data$Variante == 1, c(23:104)])
female_amount_1864_2023 <- sum(Census_data[Census_data$Simulationsjahr == 2023 & Census_data$mw == "w" & Census_data$Variante == 1, c(23:69)])/sum(Census_data[Census_data$Simulationsjahr == 2023 & Census_data$Variante == 1, c(23:104)])

female_amount_1834_2018 <- sum(Census_data[Census_data$Simulationsjahr == 2018 & Census_data$mw == "w", c(23:39)])/sum(Census_data[Census_data$Simulationsjahr == 2018, c(23:104)])
female_amount_1834_2019 <- sum(Census_data[Census_data$Simulationsjahr == 2019 & Census_data$mw == "w", c(23:39)])/sum(Census_data[Census_data$Simulationsjahr == 2019, c(23:104)])
female_amount_1834_2020 <- sum(Census_data[Census_data$Simulationsjahr == 2020 & Census_data$mw == "w", c(23:39)])/sum(Census_data[Census_data$Simulationsjahr == 2020, c(23:104)])
female_amount_1834_2021 <- sum(Census_data[Census_data$Simulationsjahr == 2021 & Census_data$mw == "w", c(23:39)])/sum(Census_data[Census_data$Simulationsjahr == 2021, c(23:104)])
female_amount_1834_2022 <- sum(Census_data[Census_data$Simulationsjahr == 2022 & Census_data$mw == "w" & Census_data$Variante == 1, c(23:39)])/sum(Census_data[Census_data$Simulationsjahr == 2022 & Census_data$Variante == 1, c(23:104)])
female_amount_1834_2023 <- sum(Census_data[Census_data$Simulationsjahr == 2023 & Census_data$mw == "w" & Census_data$Variante == 1, c(23:39)])/sum(Census_data[Census_data$Simulationsjahr == 2023 & Census_data$Variante == 1, c(23:104)])

female_amount_3549_2018 <- sum(Census_data[Census_data$Simulationsjahr == 2018 & Census_data$mw == "w", c(40:54)])/sum(Census_data[Census_data$Simulationsjahr == 2018, c(23:104)])
female_amount_3549_2019 <- sum(Census_data[Census_data$Simulationsjahr == 2019 & Census_data$mw == "w", c(40:54)])/sum(Census_data[Census_data$Simulationsjahr == 2019, c(23:104)])
female_amount_3549_2020 <- sum(Census_data[Census_data$Simulationsjahr == 2020 & Census_data$mw == "w", c(40:54)])/sum(Census_data[Census_data$Simulationsjahr == 2020, c(23:104)])
female_amount_3549_2021 <- sum(Census_data[Census_data$Simulationsjahr == 2021 & Census_data$mw == "w", c(40:54)])/sum(Census_data[Census_data$Simulationsjahr == 2021, c(23:104)])
female_amount_3549_2022 <- sum(Census_data[Census_data$Simulationsjahr == 2022 & Census_data$mw == "w" & Census_data$Variante == 1, c(40:54)])/sum(Census_data[Census_data$Simulationsjahr == 2022 & Census_data$Variante == 1, c(23:104)])
female_amount_3549_2023 <- sum(Census_data[Census_data$Simulationsjahr == 2023 & Census_data$mw == "w" & Census_data$Variante == 1, c(40:54)])/sum(Census_data[Census_data$Simulationsjahr == 2023 & Census_data$Variante == 1, c(23:104)])

female_amount_5064_2018 <- sum(Census_data[Census_data$Simulationsjahr == 2018 & Census_data$mw == "w", c(55:69)])/sum(Census_data[Census_data$Simulationsjahr == 2018, c(23:104)])
female_amount_5064_2019 <- sum(Census_data[Census_data$Simulationsjahr == 2019 & Census_data$mw == "w", c(55:69)])/sum(Census_data[Census_data$Simulationsjahr == 2019, c(23:104)])
female_amount_5064_2020 <- sum(Census_data[Census_data$Simulationsjahr == 2020 & Census_data$mw == "w", c(55:69)])/sum(Census_data[Census_data$Simulationsjahr == 2020, c(23:104)])
female_amount_5064_2021 <- sum(Census_data[Census_data$Simulationsjahr == 2021 & Census_data$mw == "w", c(55:69)])/sum(Census_data[Census_data$Simulationsjahr == 2021, c(23:104)])
female_amount_5064_2022 <- sum(Census_data[Census_data$Simulationsjahr == 2022 & Census_data$mw == "w" & Census_data$Variante == 1, c(55:69)])/sum(Census_data[Census_data$Simulationsjahr == 2022 & Census_data$Variante == 1, c(23:104)])
female_amount_5064_2023 <- sum(Census_data[Census_data$Simulationsjahr == 2023 & Census_data$mw == "w" & Census_data$Variante == 1, c(55:69)])/sum(Census_data[Census_data$Simulationsjahr == 2023 & Census_data$Variante == 1, c(23:104)])

female_amount_6574_2018 <- sum(Census_data[Census_data$Simulationsjahr == 2018 & Census_data$mw == "w", c(70:79)])/sum(Census_data[Census_data$Simulationsjahr == 2018, c(23:104)])
female_amount_6574_2019 <- sum(Census_data[Census_data$Simulationsjahr == 2019 & Census_data$mw == "w", c(70:79)])/sum(Census_data[Census_data$Simulationsjahr == 2019, c(23:104)])
female_amount_6574_2020 <- sum(Census_data[Census_data$Simulationsjahr == 2020 & Census_data$mw == "w", c(70:79)])/sum(Census_data[Census_data$Simulationsjahr == 2020, c(23:104)])
female_amount_6574_2021 <- sum(Census_data[Census_data$Simulationsjahr == 2021 & Census_data$mw == "w", c(70:79)])/sum(Census_data[Census_data$Simulationsjahr == 2021, c(23:104)])
female_amount_6574_2022 <- sum(Census_data[Census_data$Simulationsjahr == 2022 & Census_data$mw == "w" & Census_data$Variante == 1, c(70:79)])/sum(Census_data[Census_data$Simulationsjahr == 2022 & Census_data$Variante == 1, c(23:104)])
female_amount_6574_2023 <- sum(Census_data[Census_data$Simulationsjahr == 2023 & Census_data$mw == "w" & Census_data$Variante == 1, c(70:79)])/sum(Census_data[Census_data$Simulationsjahr == 2023 & Census_data$Variante == 1, c(23:104)])

female_amount_75plus_2018 <- sum(Census_data[Census_data$Simulationsjahr == 2018 & Census_data$mw == "w", c(80:104)])/sum(Census_data[Census_data$Simulationsjahr == 2018, c(23:104)])
female_amount_75plus_2019 <- sum(Census_data[Census_data$Simulationsjahr == 2019 & Census_data$mw == "w", c(80:104)])/sum(Census_data[Census_data$Simulationsjahr == 2019, c(23:104)])
female_amount_75plus_2020 <- sum(Census_data[Census_data$Simulationsjahr == 2020 & Census_data$mw == "w", c(80:104)])/sum(Census_data[Census_data$Simulationsjahr == 2020, c(23:104)])
female_amount_75plus_2021 <- sum(Census_data[Census_data$Simulationsjahr == 2021 & Census_data$mw == "w", c(80:104)])/sum(Census_data[Census_data$Simulationsjahr == 2021, c(23:104)])
female_amount_75plus_2022 <- sum(Census_data[Census_data$Simulationsjahr == 2022 & Census_data$mw == "w" & Census_data$Variante == 1, c(80:104)])/sum(Census_data[Census_data$Simulationsjahr == 2022 & Census_data$Variante == 1, c(23:104)])
female_amount_75plus_2023 <- sum(Census_data[Census_data$Simulationsjahr == 2023 & Census_data$mw == "w" & Census_data$Variante == 1, c(80:104)])/sum(Census_data[Census_data$Simulationsjahr == 2023 & Census_data$Variante == 1, c(23:104)])

female_amount_65plus_2018 <- sum(Census_data[Census_data$Simulationsjahr == 2018 & Census_data$mw == "w", c(70:104)])/sum(Census_data[Census_data$Simulationsjahr == 2018, c(23:104)])
female_amount_65plus_2019 <- sum(Census_data[Census_data$Simulationsjahr == 2019 & Census_data$mw == "w", c(70:104)])/sum(Census_data[Census_data$Simulationsjahr == 2019, c(23:104)])
female_amount_65plus_2020 <- sum(Census_data[Census_data$Simulationsjahr == 2020 & Census_data$mw == "w", c(70:104)])/sum(Census_data[Census_data$Simulationsjahr == 2020, c(23:104)])
female_amount_65plus_2021 <- sum(Census_data[Census_data$Simulationsjahr == 2021 & Census_data$mw == "w", c(70:104)])/sum(Census_data[Census_data$Simulationsjahr == 2021, c(23:104)])
female_amount_65plus_2022 <- sum(Census_data[Census_data$Simulationsjahr == 2022 & Census_data$mw == "w" & Census_data$Variante == 1, c(70:104)])/sum(Census_data[Census_data$Simulationsjahr == 2022 & Census_data$Variante == 1, c(23:104)])
female_amount_65plus_2023 <- sum(Census_data[Census_data$Simulationsjahr == 2023 & Census_data$mw == "w" & Census_data$Variante == 1, c(70:104)])/sum(Census_data[Census_data$Simulationsjahr == 2023 & Census_data$Variante == 1, c(23:104)])

female_amount_1864 <- (2095/13635)*female_amount_1864_2018+(2625/13635)*female_amount_1864_2019+(2432/13635)*female_amount_1864_2020+(2410/13635)*female_amount_1864_2021+(2255/13635)*female_amount_1864_2022+(1818/13635)*female_amount_1864_2023

female_amount_1834 <- (2095/13635)*female_amount_1834_2018+(2625/13635)*female_amount_1834_2019+(2432/13635)*female_amount_1834_2020+(2410/13635)*female_amount_1834_2021+(2255/13635)*female_amount_1834_2022+(1818/13635)*female_amount_1834_2023

female_amount_3549 <- (2095/13635)*female_amount_3549_2018+(2625/13635)*female_amount_3549_2019+(2432/13635)*female_amount_3549_2020+(2410/13635)*female_amount_3549_2021+(2255/13635)*female_amount_3549_2022+(1818/13635)*female_amount_3549_2023

female_amount_5064 <- (2095/13635)*female_amount_5064_2018+(2625/13635)*female_amount_5064_2019+(2432/13635)*female_amount_5064_2020+(2410/13635)*female_amount_5064_2021+(2255/13635)*female_amount_5064_2022+(1818/13635)*female_amount_5064_2023

female_amount_6574 <- (2095/13635)*female_amount_6574_2018+(2625/13635)*female_amount_6574_2019+(2432/13635)*female_amount_6574_2020+(2410/13635)*female_amount_6574_2021+(2255/13635)*female_amount_6574_2022+(1818/13635)*female_amount_6574_2023

female_amount_75plus <- (2095/13635)*female_amount_75plus_2018+(2625/13635)*female_amount_75plus_2019+(2432/13635)*female_amount_75plus_2020+(2410/13635)*female_amount_75plus_2021+(2255/13635)*female_amount_75plus_2022+(1818/13635)*female_amount_75plus_2023

female_amount_65plus <- (2095/13635)*female_amount_65plus_2018+(2625/13635)*female_amount_65plus_2019+(2432/13635)*female_amount_65plus_2020+(2410/13635)*female_amount_65plus_2021+(2255/13635)*female_amount_65plus_2022+(1818/13635)*female_amount_65plus_2023


#Male only
male_amount_1864_2018 <- sum(Census_data[Census_data$Simulationsjahr == 2018 & Census_data$mw == "m", c(23:69)])/sum(Census_data[Census_data$Simulationsjahr == 2018, c(23:104)])
male_amount_1864_2019 <- sum(Census_data[Census_data$Simulationsjahr == 2019 & Census_data$mw == "m", c(23:69)])/sum(Census_data[Census_data$Simulationsjahr == 2019, c(23:104)])
male_amount_1864_2020 <- sum(Census_data[Census_data$Simulationsjahr == 2020 & Census_data$mw == "m", c(23:69)])/sum(Census_data[Census_data$Simulationsjahr == 2020, c(23:104)])
male_amount_1864_2021 <- sum(Census_data[Census_data$Simulationsjahr == 2021 & Census_data$mw == "m", c(23:69)])/sum(Census_data[Census_data$Simulationsjahr == 2021, c(23:104)])
male_amount_1864_2022 <- sum(Census_data[Census_data$Simulationsjahr == 2022 & Census_data$mw == "m" & Census_data$Variante == 1, c(23:69)])/sum(Census_data[Census_data$Simulationsjahr == 2022 & Census_data$Variante == 1, c(23:104)])
male_amount_1864_2023 <- sum(Census_data[Census_data$Simulationsjahr == 2023 & Census_data$mw == "m" & Census_data$Variante == 1, c(23:69)])/sum(Census_data[Census_data$Simulationsjahr == 2023 & Census_data$Variante == 1, c(23:104)])

male_amount_1834_2018 <- sum(Census_data[Census_data$Simulationsjahr == 2018 & Census_data$mw == "m", c(23:39)])/sum(Census_data[Census_data$Simulationsjahr == 2018, c(23:104)])
male_amount_1834_2019 <- sum(Census_data[Census_data$Simulationsjahr == 2019 & Census_data$mw == "m", c(23:39)])/sum(Census_data[Census_data$Simulationsjahr == 2019, c(23:104)])
male_amount_1834_2020 <- sum(Census_data[Census_data$Simulationsjahr == 2020 & Census_data$mw == "m", c(23:39)])/sum(Census_data[Census_data$Simulationsjahr == 2020, c(23:104)])
male_amount_1834_2021 <- sum(Census_data[Census_data$Simulationsjahr == 2021 & Census_data$mw == "m", c(23:39)])/sum(Census_data[Census_data$Simulationsjahr == 2021, c(23:104)])
male_amount_1834_2022 <- sum(Census_data[Census_data$Simulationsjahr == 2022 & Census_data$mw == "m" & Census_data$Variante == 1, c(23:39)])/sum(Census_data[Census_data$Simulationsjahr == 2022 & Census_data$Variante == 1, c(23:104)])
male_amount_1834_2023 <- sum(Census_data[Census_data$Simulationsjahr == 2023 & Census_data$mw == "m" & Census_data$Variante == 1, c(23:39)])/sum(Census_data[Census_data$Simulationsjahr == 2023 & Census_data$Variante == 1, c(23:104)])

male_amount_3549_2018 <- sum(Census_data[Census_data$Simulationsjahr == 2018 & Census_data$mw == "m", c(40:54)])/sum(Census_data[Census_data$Simulationsjahr == 2018, c(23:104)])
male_amount_3549_2019 <- sum(Census_data[Census_data$Simulationsjahr == 2019 & Census_data$mw == "m", c(40:54)])/sum(Census_data[Census_data$Simulationsjahr == 2019, c(23:104)])
male_amount_3549_2020 <- sum(Census_data[Census_data$Simulationsjahr == 2020 & Census_data$mw == "m", c(40:54)])/sum(Census_data[Census_data$Simulationsjahr == 2020, c(23:104)])
male_amount_3549_2021 <- sum(Census_data[Census_data$Simulationsjahr == 2021 & Census_data$mw == "m", c(40:54)])/sum(Census_data[Census_data$Simulationsjahr == 2021, c(23:104)])
male_amount_3549_2022 <- sum(Census_data[Census_data$Simulationsjahr == 2022 & Census_data$mw == "m" & Census_data$Variante == 1, c(40:54)])/sum(Census_data[Census_data$Simulationsjahr == 2022 & Census_data$Variante == 1, c(23:104)])
male_amount_3549_2023 <- sum(Census_data[Census_data$Simulationsjahr == 2023 & Census_data$mw == "m" & Census_data$Variante == 1, c(40:54)])/sum(Census_data[Census_data$Simulationsjahr == 2023 & Census_data$Variante == 1, c(23:104)])

male_amount_5064_2018 <- sum(Census_data[Census_data$Simulationsjahr == 2018 & Census_data$mw == "m", c(55:69)])/sum(Census_data[Census_data$Simulationsjahr == 2018, c(23:104)])
male_amount_5064_2019 <- sum(Census_data[Census_data$Simulationsjahr == 2019 & Census_data$mw == "m", c(55:69)])/sum(Census_data[Census_data$Simulationsjahr == 2019, c(23:104)])
male_amount_5064_2020 <- sum(Census_data[Census_data$Simulationsjahr == 2020 & Census_data$mw == "m", c(55:69)])/sum(Census_data[Census_data$Simulationsjahr == 2020, c(23:104)])
male_amount_5064_2021 <- sum(Census_data[Census_data$Simulationsjahr == 2021 & Census_data$mw == "m", c(55:69)])/sum(Census_data[Census_data$Simulationsjahr == 2021, c(23:104)])
male_amount_5064_2022 <- sum(Census_data[Census_data$Simulationsjahr == 2022 & Census_data$mw == "m" & Census_data$Variante == 1, c(55:69)])/sum(Census_data[Census_data$Simulationsjahr == 2022 & Census_data$Variante == 1, c(23:104)])
male_amount_5064_2023 <- sum(Census_data[Census_data$Simulationsjahr == 2023 & Census_data$mw == "m" & Census_data$Variante == 1, c(55:69)])/sum(Census_data[Census_data$Simulationsjahr == 2023 & Census_data$Variante == 1, c(23:104)])

male_amount_6574_2018 <- sum(Census_data[Census_data$Simulationsjahr == 2018 & Census_data$mw == "m", c(70:79)])/sum(Census_data[Census_data$Simulationsjahr == 2018, c(23:104)])
male_amount_6574_2019 <- sum(Census_data[Census_data$Simulationsjahr == 2019 & Census_data$mw == "m", c(70:79)])/sum(Census_data[Census_data$Simulationsjahr == 2019, c(23:104)])
male_amount_6574_2020 <- sum(Census_data[Census_data$Simulationsjahr == 2020 & Census_data$mw == "m", c(70:79)])/sum(Census_data[Census_data$Simulationsjahr == 2020, c(23:104)])
male_amount_6574_2021 <- sum(Census_data[Census_data$Simulationsjahr == 2021 & Census_data$mw == "m", c(70:79)])/sum(Census_data[Census_data$Simulationsjahr == 2021, c(23:104)])
male_amount_6574_2022 <- sum(Census_data[Census_data$Simulationsjahr == 2022 & Census_data$mw == "m" & Census_data$Variante == 1, c(70:79)])/sum(Census_data[Census_data$Simulationsjahr == 2022 & Census_data$Variante == 1, c(23:104)])
male_amount_6574_2023 <- sum(Census_data[Census_data$Simulationsjahr == 2023 & Census_data$mw == "m" & Census_data$Variante == 1, c(70:79)])/sum(Census_data[Census_data$Simulationsjahr == 2023 & Census_data$Variante == 1, c(23:104)])

male_amount_75plus_2018 <- sum(Census_data[Census_data$Simulationsjahr == 2018 & Census_data$mw == "m", c(80:104)])/sum(Census_data[Census_data$Simulationsjahr == 2018, c(23:104)])
male_amount_75plus_2019 <- sum(Census_data[Census_data$Simulationsjahr == 2019 & Census_data$mw == "m", c(80:104)])/sum(Census_data[Census_data$Simulationsjahr == 2019, c(23:104)])
male_amount_75plus_2020 <- sum(Census_data[Census_data$Simulationsjahr == 2020 & Census_data$mw == "m", c(80:104)])/sum(Census_data[Census_data$Simulationsjahr == 2020, c(23:104)])
male_amount_75plus_2021 <- sum(Census_data[Census_data$Simulationsjahr == 2021 & Census_data$mw == "m", c(80:104)])/sum(Census_data[Census_data$Simulationsjahr == 2021, c(23:104)])
male_amount_75plus_2022 <- sum(Census_data[Census_data$Simulationsjahr == 2022 & Census_data$mw == "m" & Census_data$Variante == 1, c(80:104)])/sum(Census_data[Census_data$Simulationsjahr == 2022 & Census_data$Variante == 1, c(23:104)])
male_amount_75plus_2023 <- sum(Census_data[Census_data$Simulationsjahr == 2023 & Census_data$mw == "m" & Census_data$Variante == 1, c(80:104)])/sum(Census_data[Census_data$Simulationsjahr == 2023 & Census_data$Variante == 1, c(23:104)])

male_amount_65plus_2018 <- sum(Census_data[Census_data$Simulationsjahr == 2018 & Census_data$mw == "m", c(70:104)])/sum(Census_data[Census_data$Simulationsjahr == 2018, c(23:104)])
male_amount_65plus_2019 <- sum(Census_data[Census_data$Simulationsjahr == 2019 & Census_data$mw == "m", c(70:104)])/sum(Census_data[Census_data$Simulationsjahr == 2019, c(23:104)])
male_amount_65plus_2020 <- sum(Census_data[Census_data$Simulationsjahr == 2020 & Census_data$mw == "m", c(70:104)])/sum(Census_data[Census_data$Simulationsjahr == 2020, c(23:104)])
male_amount_65plus_2021 <- sum(Census_data[Census_data$Simulationsjahr == 2021 & Census_data$mw == "m", c(70:104)])/sum(Census_data[Census_data$Simulationsjahr == 2021, c(23:104)])
male_amount_65plus_2022 <- sum(Census_data[Census_data$Simulationsjahr == 2022 & Census_data$mw == "m" & Census_data$Variante == 1, c(70:104)])/sum(Census_data[Census_data$Simulationsjahr == 2022 & Census_data$Variante == 1, c(23:104)])
male_amount_65plus_2023 <- sum(Census_data[Census_data$Simulationsjahr == 2023 & Census_data$mw == "m" & Census_data$Variante == 1, c(70:104)])/sum(Census_data[Census_data$Simulationsjahr == 2023 & Census_data$Variante == 1, c(23:104)])


male_amount_1864 <- (2095/13635)*male_amount_1864_2018+(2625/13635)*male_amount_1864_2019+(2432/13635)*male_amount_1864_2020+(2410/13635)*male_amount_1864_2021+(2255/13635)*male_amount_1864_2022+(1818/13635)*male_amount_1864_2023
male_amount_1834 <- (2095/13635)*male_amount_1834_2018+(2625/13635)*male_amount_1834_2019+(2432/13635)*male_amount_1834_2020+(2410/13635)*male_amount_1834_2021+(2255/13635)*male_amount_1834_2022+(1818/13635)*male_amount_1834_2023
male_amount_3549 <- (2095/13635)*male_amount_3549_2018+(2625/13635)*male_amount_3549_2019+(2432/13635)*male_amount_3549_2020+(2410/13635)*male_amount_3549_2021+(2255/13635)*male_amount_3549_2022+(1818/13635)*male_amount_3549_2023
male_amount_5064 <- (2095/13635)*male_amount_5064_2018+(2625/13635)*male_amount_5064_2019+(2432/13635)*male_amount_5064_2020+(2410/13635)*male_amount_5064_2021+(2255/13635)*male_amount_5064_2022+(1818/13635)*male_amount_5064_2023
male_amount_6574 <- (2095/13635)*male_amount_6574_2018+(2625/13635)*male_amount_6574_2019+(2432/13635)*male_amount_6574_2020+(2410/13635)*male_amount_6574_2021+(2255/13635)*male_amount_6574_2022+(1818/13635)*male_amount_6574_2023
male_amount_75plus <- (2095/13635)*male_amount_75plus_2018+(2625/13635)*male_amount_75plus_2019+(2432/13635)*male_amount_75plus_2020+(2410/13635)*male_amount_75plus_2021+(2255/13635)*male_amount_75plus_2022+(1818/13635)*male_amount_75plus_2023
male_amount_65plus <- (2095/13635)*male_amount_65plus_2018+(2625/13635)*male_amount_65plus_2019+(2432/13635)*male_amount_65plus_2020+(2410/13635)*male_amount_65plus_2021+(2255/13635)*male_amount_65plus_2022+(1818/13635)*male_amount_65plus_2023

###Rates of long-term-care dependency
GENESIS <- read_csv2("Long-term care GENESIS.csv")
View(GENESIS)
colnames(GENESIS) <- c("Gender", "Age", "2011", "2013", "2015", "2017", "2019", "2021", "2023")

#2017
census_amount_1824_2017 <- sum(Census_data[Census_data$Simulationsjahr == 2017, c(23:29)])/sum(Census_data[Census_data$Simulationsjahr == 2017, "Bev"])
census_amount_2529_2017 <- sum(Census_data[Census_data$Simulationsjahr == 2017, c(30:34)])/sum(Census_data[Census_data$Simulationsjahr == 2017, "Bev"])
census_amount_3034_2017 <- sum(Census_data[Census_data$Simulationsjahr == 2017, c(35:39)])/sum(Census_data[Census_data$Simulationsjahr == 2017, "Bev"])
census_amount_3539_2017 <- sum(Census_data[Census_data$Simulationsjahr == 2017, c(40:44)])/sum(Census_data[Census_data$Simulationsjahr == 2017, "Bev"])
census_amount_4044_2017 <- sum(Census_data[Census_data$Simulationsjahr == 2017, c(45:49)])/sum(Census_data[Census_data$Simulationsjahr == 2017, "Bev"])
census_amount_4549_2017 <- sum(Census_data[Census_data$Simulationsjahr == 2017, c(50:54)])/sum(Census_data[Census_data$Simulationsjahr == 2017, "Bev"])
census_amount_5054_2017 <- sum(Census_data[Census_data$Simulationsjahr == 2017, c(55:59)])/sum(Census_data[Census_data$Simulationsjahr == 2017, "Bev"])
census_amount_5559_2017 <- sum(Census_data[Census_data$Simulationsjahr == 2017, c(60:64)])/sum(Census_data[Census_data$Simulationsjahr == 2017, "Bev"])
census_amount_6064_2017 <- sum(Census_data[Census_data$Simulationsjahr == 2017, c(65:69)])/sum(Census_data[Census_data$Simulationsjahr == 2017, "Bev"])
census_amount_6569_2017 <- sum(Census_data[Census_data$Simulationsjahr == 2017, c(70:74)])/sum(Census_data[Census_data$Simulationsjahr == 2017, "Bev"])
census_amount_7074_2017 <- sum(Census_data[Census_data$Simulationsjahr == 2017, c(75:79)])/sum(Census_data[Census_data$Simulationsjahr == 2017, "Bev"])
census_amount_7579_2017 <- sum(Census_data[Census_data$Simulationsjahr == 2017, c(80:84)])/sum(Census_data[Census_data$Simulationsjahr == 2017, "Bev"])
census_amount_8084_2017 <- sum(Census_data[Census_data$Simulationsjahr == 2017, c(85:89)])/sum(Census_data[Census_data$Simulationsjahr == 2017, "Bev"])
census_amount_8589_2017 <- sum(Census_data[Census_data$Simulationsjahr == 2017, c(90:94)])/sum(Census_data[Census_data$Simulationsjahr == 2017, "Bev"])
census_amount_9094_2017 <- sum(Census_data[Census_data$Simulationsjahr == 2017, c(95:99)])/sum(Census_data[Census_data$Simulationsjahr == 2017, "Bev"])
census_amount_95plus_2017 <- sum(Census_data[Census_data$Simulationsjahr == 2017, c(100:104)])/sum(Census_data[Census_data$Simulationsjahr == 2017, "Bev"])

sum_1864_2017 <- sum(census_amount_1824_2017, census_amount_2529_2017, census_amount_3034_2017, census_amount_3539_2017, census_amount_4044_2017,
                     census_amount_4549_2017, census_amount_5054_2017, census_amount_5559_2017, census_amount_6064_2017)

Long_term_care_rate_1864_2017 <- ((census_amount_1824_2017/sum_1864_2017)*as.numeric(GENESIS[GENESIS$Age == "20 to under 25 years" & GENESIS$Gender == "Total", "2017"])+
                                    (census_amount_2529_2017/sum_1864_2017)*as.numeric(GENESIS[GENESIS$Age == "25 to under 30 years" & GENESIS$Gender == "Total", "2017"])+
                                    (census_amount_3034_2017/sum_1864_2017)*as.numeric(GENESIS[GENESIS$Age == "30 to under 35 years" & GENESIS$Gender == "Total", "2017"])+
                                    (census_amount_3539_2017/sum_1864_2017)*as.numeric(GENESIS[GENESIS$Age == "35 to under 40 years" & GENESIS$Gender == "Total", "2017"])+
                                    (census_amount_4044_2017/sum_1864_2017)*as.numeric(GENESIS[GENESIS$Age == "40 to under 45 years" & GENESIS$Gender == "Total", "2017"])+
                                    (census_amount_4549_2017/sum_1864_2017)*as.numeric(GENESIS[GENESIS$Age == "45 to under 50 years" & GENESIS$Gender == "Total", "2017"])+
                                    (census_amount_5054_2017/sum_1864_2017)*as.numeric(GENESIS[GENESIS$Age == "50 to under 55 years" & GENESIS$Gender == "Total", "2017"])+
                                    (census_amount_5559_2017/sum_1864_2017)*as.numeric(GENESIS[GENESIS$Age == "55 to under 60 years" & GENESIS$Gender == "Total", "2017"])+
                                    (census_amount_6064_2017/sum_1864_2017)*as.numeric(GENESIS[GENESIS$Age == "60 to under 65 years" & GENESIS$Gender == "Total", "2017"]))/100

sum_6574_2017 <- sum(census_amount_6569_2017, census_amount_7074_2017)


Long_term_care_rate_6574_2017 <- ((census_amount_6569_2017/sum_6574_2017)*as.numeric(GENESIS[GENESIS$Age == "65 to under 70 years" & GENESIS$Gender == "Total", "2017"])+
                                    (census_amount_7074_2017/sum_6574_2017)*as.numeric(GENESIS[GENESIS$Age == "70 to under 75 years" & GENESIS$Gender == "Total", "2017"]))/100


sum_75plus_2017 <- sum(census_amount_7579_2017, census_amount_8084_2017, census_amount_8589_2017, census_amount_9094_2017, census_amount_95plus_2017)

Long_term_care_rate_75plus_2017 <-((census_amount_7579_2017/sum_75plus_2017)*as.numeric(GENESIS[GENESIS$Age == "75 to under 80 years" & GENESIS$Gender == "Total", "2017"])+
                                     (census_amount_8084_2017/sum_75plus_2017)*as.numeric(GENESIS[GENESIS$Age == "80 to under 85 years" & GENESIS$Gender == "Total", "2017"])+
                                     (census_amount_8589_2017/sum_75plus_2017)*as.numeric(GENESIS[GENESIS$Age == "85 to under 90 years" & GENESIS$Gender == "Total", "2017"])+
                                     (census_amount_9094_2017/sum_75plus_2017)*as.numeric(GENESIS[GENESIS$Age == "90 to under 95 years" & GENESIS$Gender == "Total", "2017"])+
                                     (census_amount_95plus_2017/sum_75plus_2017)*as.numeric(GENESIS[GENESIS$Age == "95 years and over" & GENESIS$Gender == "Total", "2017"]))/100

sum_65plus_2017 <- sum(census_amount_6569_2017, census_amount_7074_2017,census_amount_7579_2017, census_amount_8084_2017, census_amount_8589_2017, census_amount_9094_2017, census_amount_95plus_2017)

Long_term_care_rate_65plus_2017 <-((census_amount_6569_2017/sum_65plus_2017)*as.numeric(GENESIS[GENESIS$Age == "65 to under 70 years" & GENESIS$Gender == "Total", "2017"])+
                                     (census_amount_7074_2017/sum_65plus_2017)*as.numeric(GENESIS[GENESIS$Age == "70 to under 75 years" & GENESIS$Gender == "Total", "2017"])+
                                     (census_amount_7579_2017/sum_65plus_2017)*as.numeric(GENESIS[GENESIS$Age == "75 to under 80 years" & GENESIS$Gender == "Total", "2017"])+
                                     (census_amount_8084_2017/sum_65plus_2017)*as.numeric(GENESIS[GENESIS$Age == "80 to under 85 years" & GENESIS$Gender == "Total", "2017"])+
                                     (census_amount_8589_2017/sum_65plus_2017)*as.numeric(GENESIS[GENESIS$Age == "85 to under 90 years" & GENESIS$Gender == "Total", "2017"])+
                                     (census_amount_9094_2017/sum_65plus_2017)*as.numeric(GENESIS[GENESIS$Age == "90 to under 95 years" & GENESIS$Gender == "Total", "2017"])+
                                     (census_amount_95plus_2017/sum_65plus_2017)*as.numeric(GENESIS[GENESIS$Age == "95 years and over" & GENESIS$Gender == "Total", "2017"]))/100


#2019
census_amount_1824_2019 <- sum(Census_data[Census_data$Simulationsjahr == 2019, c(23:29)])/sum(Census_data[Census_data$Simulationsjahr == 2019, "Bev"])
census_amount_2529_2019 <- sum(Census_data[Census_data$Simulationsjahr == 2019, c(30:34)])/sum(Census_data[Census_data$Simulationsjahr == 2019, "Bev"])
census_amount_3034_2019 <- sum(Census_data[Census_data$Simulationsjahr == 2019, c(35:39)])/sum(Census_data[Census_data$Simulationsjahr == 2019, "Bev"])
census_amount_3539_2019 <- sum(Census_data[Census_data$Simulationsjahr == 2019, c(40:44)])/sum(Census_data[Census_data$Simulationsjahr == 2019, "Bev"])
census_amount_4044_2019 <- sum(Census_data[Census_data$Simulationsjahr == 2019, c(45:49)])/sum(Census_data[Census_data$Simulationsjahr == 2019, "Bev"])
census_amount_4549_2019 <- sum(Census_data[Census_data$Simulationsjahr == 2019, c(50:54)])/sum(Census_data[Census_data$Simulationsjahr == 2019, "Bev"])
census_amount_5054_2019 <- sum(Census_data[Census_data$Simulationsjahr == 2019, c(55:59)])/sum(Census_data[Census_data$Simulationsjahr == 2019, "Bev"])
census_amount_5559_2019 <- sum(Census_data[Census_data$Simulationsjahr == 2019, c(60:64)])/sum(Census_data[Census_data$Simulationsjahr == 2019, "Bev"])
census_amount_6064_2019 <- sum(Census_data[Census_data$Simulationsjahr == 2019, c(65:69)])/sum(Census_data[Census_data$Simulationsjahr == 2019, "Bev"])
census_amount_6569_2019 <- sum(Census_data[Census_data$Simulationsjahr == 2019, c(70:74)])/sum(Census_data[Census_data$Simulationsjahr == 2019, "Bev"])
census_amount_7074_2019 <- sum(Census_data[Census_data$Simulationsjahr == 2019, c(75:79)])/sum(Census_data[Census_data$Simulationsjahr == 2019, "Bev"])
census_amount_7579_2019 <- sum(Census_data[Census_data$Simulationsjahr == 2019, c(80:84)])/sum(Census_data[Census_data$Simulationsjahr == 2019, "Bev"])
census_amount_8084_2019 <- sum(Census_data[Census_data$Simulationsjahr == 2019, c(85:89)])/sum(Census_data[Census_data$Simulationsjahr == 2019, "Bev"])
census_amount_8589_2019 <- sum(Census_data[Census_data$Simulationsjahr == 2019, c(90:94)])/sum(Census_data[Census_data$Simulationsjahr == 2019, "Bev"])
census_amount_9094_2019 <- sum(Census_data[Census_data$Simulationsjahr == 2019, c(95:99)])/sum(Census_data[Census_data$Simulationsjahr == 2019, "Bev"])
census_amount_95plus_2019 <- sum(Census_data[Census_data$Simulationsjahr == 2019, c(100:104)])/sum(Census_data[Census_data$Simulationsjahr == 2019, "Bev"])


sum_1864_2019 <- sum(census_amount_1824_2019, census_amount_2529_2019, census_amount_3034_2019, census_amount_3539_2019, census_amount_4044_2019,
                     census_amount_4549_2019, census_amount_5054_2019, census_amount_5559_2019, census_amount_6064_2019)

Long_term_care_rate_1864_2019 <- ((census_amount_1824_2019/sum_1864_2019)*as.numeric(GENESIS[GENESIS$Age == "20 to under 25 years" & GENESIS$Gender == "Total", "2019"])+
                                    (census_amount_2529_2019/sum_1864_2019)*as.numeric(GENESIS[GENESIS$Age == "25 to under 30 years" & GENESIS$Gender == "Total", "2019"])+
                                    (census_amount_3034_2019/sum_1864_2019)*as.numeric(GENESIS[GENESIS$Age == "30 to under 35 years" & GENESIS$Gender == "Total", "2019"])+
                                    (census_amount_3539_2019/sum_1864_2019)*as.numeric(GENESIS[GENESIS$Age == "35 to under 40 years" & GENESIS$Gender == "Total", "2019"])+
                                    (census_amount_4044_2019/sum_1864_2019)*as.numeric(GENESIS[GENESIS$Age == "40 to under 45 years" & GENESIS$Gender == "Total", "2019"])+
                                    (census_amount_4549_2019/sum_1864_2019)*as.numeric(GENESIS[GENESIS$Age == "45 to under 50 years" & GENESIS$Gender == "Total", "2019"])+
                                    (census_amount_5054_2019/sum_1864_2019)*as.numeric(GENESIS[GENESIS$Age == "50 to under 55 years" & GENESIS$Gender == "Total", "2019"])+
                                    (census_amount_5559_2019/sum_1864_2019)*as.numeric(GENESIS[GENESIS$Age == "55 to under 60 years" & GENESIS$Gender == "Total", "2019"])+
                                    (census_amount_6064_2019/sum_1864_2019)*as.numeric(GENESIS[GENESIS$Age == "60 to under 65 years" & GENESIS$Gender == "Total", "2019"]))/100

sum_6574_2019 <- sum(census_amount_6569_2019, census_amount_7074_2019)

Long_term_care_rate_6574_2019 <- ((census_amount_6569_2019/sum_6574_2019)*as.numeric(GENESIS[GENESIS$Age == "65 to under 70 years" & GENESIS$Gender == "Total", "2019"])+
                                    (census_amount_7074_2019/sum_6574_2019)*as.numeric(GENESIS[GENESIS$Age == "70 to under 75 years" & GENESIS$Gender == "Total", "2019"]))/100


sum_75plus_2019 <- sum(census_amount_7579_2019, census_amount_8084_2019, census_amount_8589_2019, census_amount_9094_2019, census_amount_95plus_2019)

Long_term_care_rate_75plus_2019 <-((census_amount_7579_2019/sum_75plus_2019)*as.numeric(GENESIS[GENESIS$Age == "75 to under 80 years" & GENESIS$Gender == "Total", "2019"])+
                                     (census_amount_8084_2019/sum_75plus_2019)*as.numeric(GENESIS[GENESIS$Age == "80 to under 85 years" & GENESIS$Gender == "Total", "2019"])+
                                     (census_amount_8589_2019/sum_75plus_2019)*as.numeric(GENESIS[GENESIS$Age == "85 to under 90 years" & GENESIS$Gender == "Total", "2019"])+
                                     (census_amount_9094_2019/sum_75plus_2019)*as.numeric(GENESIS[GENESIS$Age == "90 to under 95 years" & GENESIS$Gender == "Total", "2019"])+
                                     (census_amount_95plus_2019/sum_75plus_2019)*as.numeric(GENESIS[GENESIS$Age == "95 years and over" & GENESIS$Gender == "Total", "2019"]))/100

sum_65plus_2019 <- sum(census_amount_6569_2019, census_amount_7074_2019,census_amount_7579_2019, census_amount_8084_2019, census_amount_8589_2019, census_amount_9094_2019, census_amount_95plus_2019)

Long_term_care_rate_65plus_2019 <-((census_amount_6569_2019/sum_65plus_2019)*as.numeric(GENESIS[GENESIS$Age == "65 to under 70 years" & GENESIS$Gender == "Total", "2019"])+
                                     (census_amount_7074_2019/sum_65plus_2019)*as.numeric(GENESIS[GENESIS$Age == "70 to under 75 years" & GENESIS$Gender == "Total", "2019"])+
                                     (census_amount_7579_2019/sum_65plus_2019)*as.numeric(GENESIS[GENESIS$Age == "75 to under 80 years" & GENESIS$Gender == "Total", "2019"])+
                                     (census_amount_8084_2019/sum_65plus_2019)*as.numeric(GENESIS[GENESIS$Age == "80 to under 85 years" & GENESIS$Gender == "Total", "2019"])+
                                     (census_amount_8589_2019/sum_65plus_2019)*as.numeric(GENESIS[GENESIS$Age == "85 to under 90 years" & GENESIS$Gender == "Total", "2019"])+
                                     (census_amount_9094_2019/sum_65plus_2019)*as.numeric(GENESIS[GENESIS$Age == "90 to under 95 years" & GENESIS$Gender == "Total", "2019"])+
                                     (census_amount_95plus_2019/sum_65plus_2019)*as.numeric(GENESIS[GENESIS$Age == "95 years and over" & GENESIS$Gender == "Total", "2019"]))/100


#2021
census_amount_1824_2021 <- sum(Census_data[Census_data$Simulationsjahr == 2021, c(23:29)])/sum(Census_data[Census_data$Simulationsjahr == 2021, "Bev"])
census_amount_2529_2021 <- sum(Census_data[Census_data$Simulationsjahr == 2021, c(30:34)])/sum(Census_data[Census_data$Simulationsjahr == 2021, "Bev"])
census_amount_3034_2021 <- sum(Census_data[Census_data$Simulationsjahr == 2021, c(35:39)])/sum(Census_data[Census_data$Simulationsjahr == 2021, "Bev"])
census_amount_3539_2021 <- sum(Census_data[Census_data$Simulationsjahr == 2021, c(40:44)])/sum(Census_data[Census_data$Simulationsjahr == 2021, "Bev"])
census_amount_4044_2021 <- sum(Census_data[Census_data$Simulationsjahr == 2021, c(45:49)])/sum(Census_data[Census_data$Simulationsjahr == 2021, "Bev"])
census_amount_4549_2021 <- sum(Census_data[Census_data$Simulationsjahr == 2021, c(50:54)])/sum(Census_data[Census_data$Simulationsjahr == 2021, "Bev"])
census_amount_5054_2021 <- sum(Census_data[Census_data$Simulationsjahr == 2021, c(55:59)])/sum(Census_data[Census_data$Simulationsjahr == 2021, "Bev"])
census_amount_5559_2021 <- sum(Census_data[Census_data$Simulationsjahr == 2021, c(60:64)])/sum(Census_data[Census_data$Simulationsjahr == 2021, "Bev"])
census_amount_6064_2021 <- sum(Census_data[Census_data$Simulationsjahr == 2021, c(65:69)])/sum(Census_data[Census_data$Simulationsjahr == 2021, "Bev"])
census_amount_6569_2021 <- sum(Census_data[Census_data$Simulationsjahr == 2021, c(70:74)])/sum(Census_data[Census_data$Simulationsjahr == 2021, "Bev"])
census_amount_7074_2021 <- sum(Census_data[Census_data$Simulationsjahr == 2021, c(75:79)])/sum(Census_data[Census_data$Simulationsjahr == 2021, "Bev"])
census_amount_7579_2021 <- sum(Census_data[Census_data$Simulationsjahr == 2021, c(80:84)])/sum(Census_data[Census_data$Simulationsjahr == 2021, "Bev"])
census_amount_8084_2021 <- sum(Census_data[Census_data$Simulationsjahr == 2021, c(85:89)])/sum(Census_data[Census_data$Simulationsjahr == 2021, "Bev"])
census_amount_8589_2021 <- sum(Census_data[Census_data$Simulationsjahr == 2021, c(90:94)])/sum(Census_data[Census_data$Simulationsjahr == 2021, "Bev"])
census_amount_9094_2021 <- sum(Census_data[Census_data$Simulationsjahr == 2021, c(95:99)])/sum(Census_data[Census_data$Simulationsjahr == 2021, "Bev"])
census_amount_95plus_2021 <- sum(Census_data[Census_data$Simulationsjahr == 2021, c(100:104)])/sum(Census_data[Census_data$Simulationsjahr == 2021, "Bev"])

sum_1864_2021 <- sum(census_amount_1824_2021, census_amount_2529_2021, census_amount_3034_2021, census_amount_3539_2021, census_amount_4044_2021,
                     census_amount_4549_2021, census_amount_5054_2021, census_amount_5559_2021, census_amount_6064_2021)

Long_term_care_rate_1864_2021 <- ((census_amount_1824_2021/sum_1864_2021)*as.numeric(GENESIS[GENESIS$Age == "20 to under 25 years" & GENESIS$Gender == "Total", "2021"])+
                                    (census_amount_2529_2021/sum_1864_2021)*as.numeric(GENESIS[GENESIS$Age == "25 to under 30 years" & GENESIS$Gender == "Total", "2021"])+
                                    (census_amount_3034_2021/sum_1864_2021)*as.numeric(GENESIS[GENESIS$Age == "30 to under 35 years" & GENESIS$Gender == "Total", "2021"])+
                                    (census_amount_3539_2021/sum_1864_2021)*as.numeric(GENESIS[GENESIS$Age == "35 to under 40 years" & GENESIS$Gender == "Total", "2021"])+
                                    (census_amount_4044_2021/sum_1864_2021)*as.numeric(GENESIS[GENESIS$Age == "40 to under 45 years" & GENESIS$Gender == "Total", "2021"])+
                                    (census_amount_4549_2021/sum_1864_2021)*as.numeric(GENESIS[GENESIS$Age == "45 to under 50 years" & GENESIS$Gender == "Total", "2021"])+
                                    (census_amount_5054_2021/sum_1864_2021)*as.numeric(GENESIS[GENESIS$Age == "50 to under 55 years" & GENESIS$Gender == "Total", "2021"])+
                                    (census_amount_5559_2021/sum_1864_2021)*as.numeric(GENESIS[GENESIS$Age == "55 to under 60 years" & GENESIS$Gender == "Total", "2021"])+
                                    (census_amount_6064_2021/sum_1864_2021)*as.numeric(GENESIS[GENESIS$Age == "60 to under 65 years" & GENESIS$Gender == "Total", "2021"]))/100

sum_6574_2021 <- sum(census_amount_6569_2021, census_amount_7074_2021)

Long_term_care_rate_6574_2021 <- ((census_amount_6569_2021/sum_6574_2021)*as.numeric(GENESIS[GENESIS$Age == "65 to under 70 years" & GENESIS$Gender == "Total", "2021"])+
                                    (census_amount_7074_2021/sum_6574_2021)*as.numeric(GENESIS[GENESIS$Age == "70 to under 75 years" & GENESIS$Gender == "Total", "2021"]))/100


sum_75plus_2021 <- sum(census_amount_7579_2021, census_amount_8084_2021, census_amount_8589_2021, census_amount_9094_2021, census_amount_95plus_2021)

Long_term_care_rate_75plus_2021 <-((census_amount_7579_2021/sum_75plus_2021)*as.numeric(GENESIS[GENESIS$Age == "75 to under 80 years" & GENESIS$Gender == "Total", "2021"])+
                                     (census_amount_8084_2021/sum_75plus_2021)*as.numeric(GENESIS[GENESIS$Age == "80 to under 85 years" & GENESIS$Gender == "Total", "2021"])+
                                     (census_amount_8589_2021/sum_75plus_2021)*as.numeric(GENESIS[GENESIS$Age == "85 to under 90 years" & GENESIS$Gender == "Total", "2021"])+
                                     (census_amount_9094_2021/sum_75plus_2021)*as.numeric(GENESIS[GENESIS$Age == "90 to under 95 years" & GENESIS$Gender == "Total", "2021"])+
                                     (census_amount_95plus_2021/sum_75plus_2021)*as.numeric(GENESIS[GENESIS$Age == "95 years and over" & GENESIS$Gender == "Total", "2021"]))/100


sum_65plus_2021 <- sum(census_amount_6569_2021, census_amount_7074_2021,census_amount_7579_2021, census_amount_8084_2021, census_amount_8589_2021, census_amount_9094_2021, census_amount_95plus_2021)

Long_term_care_rate_65plus_2021 <-((census_amount_6569_2021/sum_65plus_2021)*as.numeric(GENESIS[GENESIS$Age == "65 to under 70 years" & GENESIS$Gender == "Total", "2021"])+
                                     (census_amount_7074_2021/sum_65plus_2021)*as.numeric(GENESIS[GENESIS$Age == "70 to under 75 years" & GENESIS$Gender == "Total", "2021"])+
                                     (census_amount_7579_2021/sum_65plus_2021)*as.numeric(GENESIS[GENESIS$Age == "75 to under 80 years" & GENESIS$Gender == "Total", "2021"])+
                                     (census_amount_8084_2021/sum_65plus_2021)*as.numeric(GENESIS[GENESIS$Age == "80 to under 85 years" & GENESIS$Gender == "Total", "2021"])+
                                     (census_amount_8589_2021/sum_65plus_2021)*as.numeric(GENESIS[GENESIS$Age == "85 to under 90 years" & GENESIS$Gender == "Total", "2021"])+
                                     (census_amount_9094_2021/sum_65plus_2021)*as.numeric(GENESIS[GENESIS$Age == "90 to under 95 years" & GENESIS$Gender == "Total", "2021"])+
                                     (census_amount_95plus_2021/sum_65plus_2021)*as.numeric(GENESIS[GENESIS$Age == "95 years and over" & GENESIS$Gender == "Total", "2021"]))/100


#2023
census_amount_1824_2023 <- sum(Census_data[Census_data$Simulationsjahr == 2023 & Census_data$Variante == 1, c(23:29)])/sum(Census_data[Census_data$Simulationsjahr == 2023 & Census_data$Variante == 1, "Bev"])
census_amount_2529_2023 <- sum(Census_data[Census_data$Simulationsjahr == 2023 & Census_data$Variante == 1, c(30:34)])/sum(Census_data[Census_data$Simulationsjahr == 2023 & Census_data$Variante == 1, "Bev"])
census_amount_3034_2023 <- sum(Census_data[Census_data$Simulationsjahr == 2023 & Census_data$Variante == 1, c(35:39)])/sum(Census_data[Census_data$Simulationsjahr == 2023 & Census_data$Variante == 1, "Bev"])
census_amount_3539_2023 <- sum(Census_data[Census_data$Simulationsjahr == 2023 & Census_data$Variante == 1, c(40:44)])/sum(Census_data[Census_data$Simulationsjahr == 2023 & Census_data$Variante == 1, "Bev"])
census_amount_4044_2023 <- sum(Census_data[Census_data$Simulationsjahr == 2023 & Census_data$Variante == 1, c(45:49)])/sum(Census_data[Census_data$Simulationsjahr == 2023 & Census_data$Variante == 1, "Bev"])
census_amount_4549_2023 <- sum(Census_data[Census_data$Simulationsjahr == 2023 & Census_data$Variante == 1, c(50:54)])/sum(Census_data[Census_data$Simulationsjahr == 2023 & Census_data$Variante == 1, "Bev"])
census_amount_5054_2023 <- sum(Census_data[Census_data$Simulationsjahr == 2023 & Census_data$Variante == 1, c(55:59)])/sum(Census_data[Census_data$Simulationsjahr == 2023 & Census_data$Variante == 1, "Bev"])
census_amount_5559_2023 <- sum(Census_data[Census_data$Simulationsjahr == 2023 & Census_data$Variante == 1, c(60:64)])/sum(Census_data[Census_data$Simulationsjahr == 2023 & Census_data$Variante == 1, "Bev"])
census_amount_6064_2023 <- sum(Census_data[Census_data$Simulationsjahr == 2023 & Census_data$Variante == 1, c(65:69)])/sum(Census_data[Census_data$Simulationsjahr == 2023 & Census_data$Variante == 1, "Bev"])
census_amount_6569_2023 <- sum(Census_data[Census_data$Simulationsjahr == 2023 & Census_data$Variante == 1, c(70:74)])/sum(Census_data[Census_data$Simulationsjahr == 2023 & Census_data$Variante == 1, "Bev"])
census_amount_7074_2023 <- sum(Census_data[Census_data$Simulationsjahr == 2023 & Census_data$Variante == 1, c(75:79)])/sum(Census_data[Census_data$Simulationsjahr == 2023 & Census_data$Variante == 1, "Bev"])
census_amount_7579_2023 <- sum(Census_data[Census_data$Simulationsjahr == 2023 & Census_data$Variante == 1, c(80:84)])/sum(Census_data[Census_data$Simulationsjahr == 2023 & Census_data$Variante == 1, "Bev"])
census_amount_8084_2023 <- sum(Census_data[Census_data$Simulationsjahr == 2023 & Census_data$Variante == 1, c(85:89)])/sum(Census_data[Census_data$Simulationsjahr == 2023 & Census_data$Variante == 1, "Bev"])
census_amount_8589_2023 <- sum(Census_data[Census_data$Simulationsjahr == 2023 & Census_data$Variante == 1, c(90:94)])/sum(Census_data[Census_data$Simulationsjahr == 2023 & Census_data$Variante == 1, "Bev"])
census_amount_9094_2023 <- sum(Census_data[Census_data$Simulationsjahr == 2023 & Census_data$Variante == 1, c(95:99)])/sum(Census_data[Census_data$Simulationsjahr == 2023 & Census_data$Variante == 1, "Bev"])
census_amount_95plus_2023 <- sum(Census_data[Census_data$Simulationsjahr == 2023 & Census_data$Variante == 1, c(100:104)])/sum(Census_data[Census_data$Simulationsjahr == 2023 & Census_data$Variante == 1, "Bev"])

sum_1864_2023 <- sum(census_amount_1824_2023, census_amount_2529_2023, census_amount_3034_2023, census_amount_3539_2023, census_amount_4044_2023,
                     census_amount_4549_2023, census_amount_5054_2023, census_amount_5559_2023, census_amount_6064_2023)

Long_term_care_rate_1864_2023 <- ((census_amount_1824_2023/sum_1864_2023)*as.numeric(GENESIS[GENESIS$Age == "20 to under 25 years" & GENESIS$Gender == "Total", "2023"])+
                                    (census_amount_2529_2023/sum_1864_2023)*as.numeric(GENESIS[GENESIS$Age == "25 to under 30 years" & GENESIS$Gender == "Total", "2023"])+
                                    (census_amount_3034_2023/sum_1864_2023)*as.numeric(GENESIS[GENESIS$Age == "30 to under 35 years" & GENESIS$Gender == "Total", "2023"])+
                                    (census_amount_3539_2023/sum_1864_2023)*as.numeric(GENESIS[GENESIS$Age == "35 to under 40 years" & GENESIS$Gender == "Total", "2023"])+
                                    (census_amount_4044_2023/sum_1864_2023)*as.numeric(GENESIS[GENESIS$Age == "40 to under 45 years" & GENESIS$Gender == "Total", "2023"])+
                                    (census_amount_4549_2023/sum_1864_2023)*as.numeric(GENESIS[GENESIS$Age == "45 to under 50 years" & GENESIS$Gender == "Total", "2023"])+
                                    (census_amount_5054_2023/sum_1864_2023)*as.numeric(GENESIS[GENESIS$Age == "50 to under 55 years" & GENESIS$Gender == "Total", "2023"])+
                                    (census_amount_5559_2023/sum_1864_2023)*as.numeric(GENESIS[GENESIS$Age == "55 to under 60 years" & GENESIS$Gender == "Total", "2023"])+
                                    (census_amount_6064_2023/sum_1864_2023)*as.numeric(GENESIS[GENESIS$Age == "60 to under 65 years" & GENESIS$Gender == "Total", "2023"]))/100

sum_6574_2023 <- sum(census_amount_6569_2023, census_amount_7074_2023)

Long_term_care_rate_6574_2023 <- ((census_amount_6569_2023/sum_6574_2023)*as.numeric(GENESIS[GENESIS$Age == "65 to under 70 years" & GENESIS$Gender == "Total", "2023"])+
                                    (census_amount_7074_2023/sum_6574_2023)*as.numeric(GENESIS[GENESIS$Age == "70 to under 75 years" & GENESIS$Gender == "Total", "2023"]))/100


sum_75plus_2023 <- sum(census_amount_7579_2023, census_amount_8084_2023, census_amount_8589_2023, census_amount_9094_2023, census_amount_95plus_2023)

Long_term_care_rate_75plus_2023 <-((census_amount_7579_2023/sum_75plus_2023)*as.numeric(GENESIS[GENESIS$Age == "75 to under 80 years" & GENESIS$Gender == "Total", "2023"])+
                                     (census_amount_8084_2023/sum_75plus_2023)*as.numeric(GENESIS[GENESIS$Age == "80 to under 85 years" & GENESIS$Gender == "Total", "2023"])+
                                     (census_amount_8589_2023/sum_75plus_2023)*as.numeric(GENESIS[GENESIS$Age == "85 to under 90 years" & GENESIS$Gender == "Total", "2023"])+
                                     (census_amount_9094_2023/sum_75plus_2023)*as.numeric(GENESIS[GENESIS$Age == "90 to under 95 years" & GENESIS$Gender == "Total", "2023"])+
                                     (census_amount_95plus_2023/sum_75plus_2023)*as.numeric(GENESIS[GENESIS$Age == "95 years and over" & GENESIS$Gender == "Total", "2023"]))/100


sum_65plus_2023 <- sum(census_amount_6569_2023, census_amount_7074_2023,census_amount_7579_2023, census_amount_8084_2023, census_amount_8589_2023, census_amount_9094_2023, census_amount_95plus_2023)

Long_term_care_rate_65plus_2023 <-((census_amount_6569_2023/sum_65plus_2023)*as.numeric(GENESIS[GENESIS$Age == "65 to under 70 years" & GENESIS$Gender == "Total", "2023"])+
                                     (census_amount_7074_2023/sum_65plus_2023)*as.numeric(GENESIS[GENESIS$Age == "70 to under 75 years" & GENESIS$Gender == "Total", "2023"])+
                                     (census_amount_7579_2023/sum_65plus_2023)*as.numeric(GENESIS[GENESIS$Age == "75 to under 80 years" & GENESIS$Gender == "Total", "2023"])+
                                     (census_amount_8084_2023/sum_65plus_2023)*as.numeric(GENESIS[GENESIS$Age == "80 to under 85 years" & GENESIS$Gender == "Total", "2023"])+
                                     (census_amount_8589_2023/sum_65plus_2023)*as.numeric(GENESIS[GENESIS$Age == "85 to under 90 years" & GENESIS$Gender == "Total", "2023"])+
                                     (census_amount_9094_2023/sum_65plus_2023)*as.numeric(GENESIS[GENESIS$Age == "90 to under 95 years" & GENESIS$Gender == "Total", "2023"])+
                                     (census_amount_95plus_2023/sum_65plus_2023)*as.numeric(GENESIS[GENESIS$Age == "95 years and over" & GENESIS$Gender == "Total", "2023"]))/100


Long_term_care_rate_1864_2017
```

```
## [1] 0.009617073
```

```
Long_term_care_rate_1864_2019
```

```
## [1] 0.01208859
```

```
Long_term_care_rate_1864_2021
```

```
## [1] 0.01534732
```

```
Long_term_care_rate_1864_2023
```

```
## [1] 0.01787395
```

```
Long_term_care_rate_6574_2017
```

```
## [1] 0.04928208
```

```
Long_term_care_rate_6574_2019
```

```
## [1] 0.05923031
```

```
Long_term_care_rate_6574_2021
```

```
## [1] 0.07348887
```

```
Long_term_care_rate_6574_2023
```

```
## [1] 0.08456034
```

```
Long_term_care_rate_75plus_2017
```

```
## [1] 0.2512004
```

```
Long_term_care_rate_75plus_2019
```

```
## [1] 0.2922611
```

```
Long_term_care_rate_75plus_2021
```

```
## [1] 0.3477314
```

```
Long_term_care_rate_75plus_2023
```

```
## [1] 0.3903115
```

```
year_distribution_data <- as.data.frame(table(KODAP_data_complete$Beginn_Therapie))

Long_term_care_rate_1864_average <- (year_distribution_data[year_distribution_data$Var1 == 2018, "Freq"]/sum(year_distribution_data$Freq))*Long_term_care_rate_1864_2017+
  (year_distribution_data[year_distribution_data$Var1 == 2019, "Freq"]/sum(year_distribution_data$Freq))*Long_term_care_rate_1864_2019+
  (year_distribution_data[year_distribution_data$Var1 == 2020, "Freq"]/sum(year_distribution_data$Freq))*Long_term_care_rate_1864_2019+
  (year_distribution_data[year_distribution_data$Var1 == 2021, "Freq"]/sum(year_distribution_data$Freq))*Long_term_care_rate_1864_2021+
  (year_distribution_data[year_distribution_data$Var1 == 2022, "Freq"]/sum(year_distribution_data$Freq))*Long_term_care_rate_1864_2021+
  (year_distribution_data[year_distribution_data$Var1 == 2023, "Freq"]/sum(year_distribution_data$Freq))*Long_term_care_rate_1864_2023
  
Long_term_care_rate_6574_average <- (year_distribution_data[year_distribution_data$Var1 == 2018, "Freq"]/sum(year_distribution_data$Freq))*Long_term_care_rate_6574_2017+
  (year_distribution_data[year_distribution_data$Var1 == 2019, "Freq"]/sum(year_distribution_data$Freq))*Long_term_care_rate_6574_2019+
  (year_distribution_data[year_distribution_data$Var1 == 2020, "Freq"]/sum(year_distribution_data$Freq))*Long_term_care_rate_6574_2019+
  (year_distribution_data[year_distribution_data$Var1 == 2021, "Freq"]/sum(year_distribution_data$Freq))*Long_term_care_rate_6574_2021+
  (year_distribution_data[year_distribution_data$Var1 == 2022, "Freq"]/sum(year_distribution_data$Freq))*Long_term_care_rate_6574_2021+
  (year_distribution_data[year_distribution_data$Var1 == 2023, "Freq"]/sum(year_distribution_data$Freq))*Long_term_care_rate_6574_2023

Long_term_care_rate_75plus_average <- (year_distribution_data[year_distribution_data$Var1 == 2018, "Freq"]/sum(year_distribution_data$Freq))*Long_term_care_rate_75plus_2017+
  (year_distribution_data[year_distribution_data$Var1 == 2019, "Freq"]/sum(year_distribution_data$Freq))*Long_term_care_rate_75plus_2019+
  (year_distribution_data[year_distribution_data$Var1 == 2020, "Freq"]/sum(year_distribution_data$Freq))*Long_term_care_rate_75plus_2019+
  (year_distribution_data[year_distribution_data$Var1 == 2021, "Freq"]/sum(year_distribution_data$Freq))*Long_term_care_rate_75plus_2021+
  (year_distribution_data[year_distribution_data$Var1 == 2022, "Freq"]/sum(year_distribution_data$Freq))*Long_term_care_rate_75plus_2021+
  (year_distribution_data[year_distribution_data$Var1 == 2023, "Freq"]/sum(year_distribution_data$Freq))*Long_term_care_rate_75plus_2023

Long_term_care_rate_65plus_average <- (year_distribution_data[year_distribution_data$Var1 == 2018, "Freq"]/sum(year_distribution_data$Freq))*Long_term_care_rate_65plus_2017+
  (year_distribution_data[year_distribution_data$Var1 == 2019, "Freq"]/sum(year_distribution_data$Freq))*Long_term_care_rate_65plus_2019+
  (year_distribution_data[year_distribution_data$Var1 == 2020, "Freq"]/sum(year_distribution_data$Freq))*Long_term_care_rate_65plus_2019+
  (year_distribution_data[year_distribution_data$Var1 == 2021, "Freq"]/sum(year_distribution_data$Freq))*Long_term_care_rate_65plus_2021+
  (year_distribution_data[year_distribution_data$Var1 == 2022, "Freq"]/sum(year_distribution_data$Freq))*Long_term_care_rate_65plus_2021+
  (year_distribution_data[year_distribution_data$Var1 == 2023, "Freq"]/sum(year_distribution_data$Freq))*Long_term_care_rate_65plus_2023
```

## Main Analyses

```
#Read prevalence estimates
Prevalence_estimates <- read_excel("Prevalence_estimates.xlsx")
Prevalence_estimates
```

```
## # A tibble: 17 × 6
##    Diagnosis                  `1834y` `3549y` `5064y` `6574y` `75yplus`
##    <chr>                        <dbl>   <dbl>   <dbl>   <dbl>     <dbl>
##  1 Any mental disorder           35.8    28      26.4    19.6      19.6
##  2 Any mood disorder             15.1    10.3     7       5.9       5.9
##  3 Major Depressive Disorder     10       7.2     5.2     4.4       4.4
##  4 Dysthymia                      2.1     1.7     1.3     1.6       1.6
##  5 Any anxiety disorder          18.1    16.2    15.3    11.1      11.1
##  6 Panic disorder/Agoraphobia     4.2     4.1     4.1     3.5       3.5
##  7 Social phobia                  4.6     3.1     2.2     0.7       0.7
##  8 Specific phobias              12.3     9.5    10.9     8.4       8.4
##  9 GAD                            3.3     2       2.3     1.3       1.3
## 10 OCD                            7.2     3.6     2.2     1.1       1.1
## 11 PTSD                           3.7     2.5     1       1.8       1.8
## 12 Any somatoform disorder        4.2     3.8     3.6     2.1       2.1
## 13 Somatization disorder          0.9     0.6     0.9     0.8       0.8
## 14 Pain disorder                  4       3.8     3       1.6       1.6
## 15 Eating disorders               2.3     0.5     0.7     0.4       0.4
## 16 Substance use disorders        8.4     5.9     5.5     2.5       2.5
## 17 Psychotic disorders            4.2     2.2     2.5     1.3       1.3
```

```
##################
#####Analyses#####
##################

#####################
#Any Mental Disorder#
#####################

prevalence_1834 <- as.numeric(Prevalence_estimates[Prevalence_estimates$Diagnosis == "Any mental disorder", "1834y"])
prevalence_3549 <- as.numeric(Prevalence_estimates[Prevalence_estimates$Diagnosis == "Any mental disorder", "3549y"])
prevalence_5064 <- as.numeric(Prevalence_estimates[Prevalence_estimates$Diagnosis == "Any mental disorder", "5064y"])

prevalence_1864 <- (census_amount_1834/sum(census_amount_1834,census_amount_3549, census_amount_5064))*prevalence_1834+
  (census_amount_3549/sum(census_amount_1834,census_amount_3549, census_amount_5064))*prevalence_3549+
  (census_amount_5064/sum(census_amount_1834,census_amount_3549, census_amount_5064))*prevalence_5064

prevalence_6574 <- as.numeric(Prevalence_estimates[Prevalence_estimates$Diagnosis == "Any mental disorder", "6574y"])
prevalence_75plus <- as.numeric(Prevalence_estimates[Prevalence_estimates$Diagnosis == "Any mental disorder", "75yplus"])

#Ratios in reference population
expected_ratio_1864 <- (census_amount_1864*prevalence_1864)/((census_amount_1864*prevalence_1864)+(census_amount_6574*prevalence_6574)+(census_amount_75plus*prevalence_75plus))
expected_ratio_6574 <- (census_amount_6574*prevalence_6574)/((census_amount_1864*prevalence_1864)+(census_amount_6574*prevalence_6574)+(census_amount_75plus*prevalence_75plus))
expected_ratio_75plus <- (census_amount_75plus*prevalence_75plus)/((census_amount_1864*prevalence_1864)+(census_amount_6574*prevalence_6574)+(census_amount_75plus*prevalence_75plus))

expected_ratios <- c(expected_ratio_1864,
                     expected_ratio_6574,
                     expected_ratio_75plus)
round(expected_ratios*100,1)
```

```
## [1] 81.0  9.3  9.7
```

```
#Observed vs. expected: 
Observation <- as.vector(table(KODAP_data_complete$Age_Stepped))
Expected <- expected_ratios*length(KODAP_data_complete$Patient_ID)

Observation
```

```
## [1] 13218   324    93
```

```
round(Observation/length(KODAP_data_complete$Patient_ID)*100, 1)
```

```
## [1] 96.9  2.4  0.7
```

```
# Chi-squared test for given probabilities
test <- chisq.test(Observation, p = expected_ratios)
test
```

```
## 
##  Chi-squared test for given probabilities
## 
## data:  Observation
## X-squared = 2272.7, df = 2, p-value < 2.2e-16
```

```
# Post-hoc binomial-test for each category with Bonferroni-correction
binom.test(Observation[1], sum(Observation), expected_ratios[1])$p.value*3
```

```
## [1] 1.482197e-323
```

```
binom.test(Observation[2], sum(Observation), expected_ratios[2])$p.value*3
```

```
## [1] 3.860477e-234
```

```
binom.test(Observation[3], sum(Observation), expected_ratios[3])$p.value*3
```

```
## [1] 1.482197e-323
```

```
#Estimating underrepresentation compared to reference population
#Representation quotients
Observation/Expected
```

```
## [1] 1.19671990 0.25585825 0.07026915
```

```
#Confidence-intervals of Representation quotients

#Confidence-intervals around sample proportion
prop.test(x = Observation[2], n = sum(Observation), conf.level = .95)
```

```
## 
##  1-sample proportions test with continuity correction
## 
## data:  Observation[2] out of sum(Observation), null probability 0.5
## X-squared = 12368, df = 1, p-value < 2.2e-16
## alternative hypothesis: true p is not equal to 0.5
## 95 percent confidence interval:
##  0.02130213 0.02649473
## sample estimates:
##          p 
## 0.02376238
```

```
#Confidence-intervals around sample proportion relative to expected proportion
round(prop.test(x = Observation[2], n = sum(Observation), conf.level = .95)$estimate/expected_ratio_6574,2)
```

```
##    p 
## 0.26
```

```
round(as.vector(prop.test(x = Observation[2], n = sum(Observation), conf.level = .95)$conf.int)/expected_ratio_6574, 2)
```

```
## [1] 0.23 0.29
```

```
#Confidence-intervals around sample proportion
prop.test(x = Observation[3], n = sum(Observation), conf.level = .95)
```

```
## 
##  1-sample proportions test with continuity correction
## 
## data:  Observation[3] out of sum(Observation), null probability 0.5
## X-squared = 13264, df = 1, p-value < 2.2e-16
## alternative hypothesis: true p is not equal to 0.5
## 95 percent confidence interval:
##  0.005538338 0.008388176
## sample estimates:
##           p 
## 0.006820682
```

```
#Confidence-intervals around sample proportion relative to expected proportion
round(prop.test(x = Observation[3], n = sum(Observation), conf.level = .95)$estimate/expected_ratio_75plus,2)
```

```
##    p 
## 0.07
```

```
round(as.vector(prop.test(x = Observation[3], n = sum(Observation), conf.level = .95)$conf.int)/expected_ratio_75plus,2)
```

```
## [1] 0.06 0.09
```

```
#####################
#Any Mood Disorder#
#####################

prevalence_1834 <- as.numeric(Prevalence_estimates[Prevalence_estimates$Diagnosis == "Any mood disorder", "1834y"])
prevalence_3549 <- as.numeric(Prevalence_estimates[Prevalence_estimates$Diagnosis == "Any mood disorder", "3549y"])
prevalence_5064 <- as.numeric(Prevalence_estimates[Prevalence_estimates$Diagnosis == "Any mood disorder", "5064y"])

prevalence_1864 <- (census_amount_1834/sum(census_amount_1834,census_amount_3549, census_amount_5064))*prevalence_1834+
  (census_amount_3549/sum(census_amount_1834,census_amount_3549, census_amount_5064))*prevalence_3549+
  (census_amount_5064/sum(census_amount_1834,census_amount_3549, census_amount_5064))*prevalence_5064

prevalence_6574 <- as.numeric(Prevalence_estimates[Prevalence_estimates$Diagnosis == "Any mood disorder", "6574y"])
prevalence_75plus <- as.numeric(Prevalence_estimates[Prevalence_estimates$Diagnosis == "Any mood disorder", "75yplus"])

#Ratios in reference population
expected_ratio_1864 <- (census_amount_1864*prevalence_1864)/((census_amount_1864*prevalence_1864)+(census_amount_6574*prevalence_6574)+(census_amount_75plus*prevalence_75plus))
expected_ratio_6574 <- (census_amount_6574*prevalence_6574)/((census_amount_1864*prevalence_1864)+(census_amount_6574*prevalence_6574)+(census_amount_75plus*prevalence_75plus))
expected_ratio_75plus <- (census_amount_75plus*prevalence_75plus)/((census_amount_1864*prevalence_1864)+(census_amount_6574*prevalence_6574)+(census_amount_75plus*prevalence_75plus))

expected_ratios <- c(expected_ratio_1864,
                     expected_ratio_6574,
                     expected_ratio_75plus)
round(expected_ratios*100,1)
```

```
## [1] 83.4  8.1  8.5
```

```
KODAP_data_complete_any_mood_disorder <- KODAP_data_complete[KODAP_data_complete$ICD1_pre_recode %in% c("F30.X F31.X Manische Episode oder Bipolare Störungen",
                                                                                                        "F31.7 Bipolare affktive Störung, gegenwärtig remittiert",
                                                                                                        "F32 Depressive Episode",
                                                                                                        "F33 Rezidivierende depressive Störung",
                                                                                                        "F33.4 Rezidivierende depressive Störung, gegenwärtig remittiert",
                                                                                                        "F34 Anhaltende affektive Störungen",
                                                                                                        "F38.X Andere affektive Störung") |
                                                               KODAP_data_complete$ICD2_pre_recode %in% c("F30.X F31.X Manische Episode oder Bipolare Störungen",
                                                                                                          "F31.7 Bipolare affktive Störung, gegenwärtig remittiert",
                                                                                                          "F32 Depressive Episode",
                                                                                                          "F33 Rezidivierende depressive Störung",
                                                                                                          "F33.4 Rezidivierende depressive Störung, gegenwärtig remittiert",
                                                                                                          "F34 Anhaltende affektive Störungen",
                                                                                                          "F38.X Andere affektive Störung") |
                                                               KODAP_data_complete$ICD3_pre_recode %in% c("F30.X F31.X Manische Episode oder Bipolare Störungen",
                                                                                                          "F31.7 Bipolare affktive Störung, gegenwärtig remittiert",
                                                                                                          "F32 Depressive Episode",
                                                                                                          "F33 Rezidivierende depressive Störung",
                                                                                                          "F33.4 Rezidivierende depressive Störung, gegenwärtig remittiert",
                                                                                                          "F34 Anhaltende affektive Störungen",
                                                                                                          "F38.X Andere affektive Störung") |
                                                               KODAP_data_complete$ICD4_pre_recode %in% c("F30.X F31.X Manische Episode oder Bipolare Störungen",
                                                                                                          "F31.7 Bipolare affktive Störung, gegenwärtig remittiert",
                                                                                                          "F32 Depressive Episode",
                                                                                                          "F33 Rezidivierende depressive Störung",
                                                                                                          "F33.4 Rezidivierende depressive Störung, gegenwärtig remittiert",
                                                                                                          "F34 Anhaltende affektive Störungen",
                                                                                                          "F38.X Andere affektive Störung") |
                                                               KODAP_data_complete$ICD5_pre_recode %in% c("F30.X F31.X Manische Episode oder Bipolare Störungen",
                                                                                                          "F31.7 Bipolare affktive Störung, gegenwärtig remittiert",
                                                                                                          "F32 Depressive Episode",
                                                                                                          "F33 Rezidivierende depressive Störung",
                                                                                                          "F33.4 Rezidivierende depressive Störung, gegenwärtig remittiert",
                                                                                                          "F34 Anhaltende affektive Störungen",
                                                                                                          "F38.X Andere affektive Störung"),]

length(KODAP_data_complete_any_mood_disorder$Patient_ID)
```

```
## [1] 8244
```

```
#Observed vs. expected: 
Observation <- as.vector(table(KODAP_data_complete_any_mood_disorder$Age_Stepped))
Expected <- expected_ratios*length(KODAP_data_complete_any_mood_disorder$Patient_ID)

Observation
```

```
## [1] 8027  179   38
```

```
round(Observation/length(KODAP_data_complete_any_mood_disorder$Patient_ID)*100,1)
```

```
## [1] 97.4  2.2  0.5
```

```
# Chi-squared test for given probabilities
test <- chisq.test(Observation, p = expected_ratios)
test
```

```
## 
##  Chi-squared test for given probabilities
## 
## data:  Observation
## X-squared = 1174.8, df = 2, p-value < 2.2e-16
```

```
# Post-hoc binomial-test for each category with Bonferroni-correction
binom.test(Observation[1], sum(Observation), expected_ratios[1])$p.value*3
```

```
## [1] 1.482197e-323
```

```
binom.test(Observation[2], sum(Observation), expected_ratios[2])$p.value*3
```

```
## [1] 4.61793e-118
```

```
binom.test(Observation[3], sum(Observation), expected_ratios[3])$p.value*3
```

```
## [1] 5.096773e-252
```

```
#Estimating underrepresentation compared to reference population
#Representation quotients
Observation/Expected
```

```
## [1] 1.16716254 0.26786863 0.05441013
```

```
#Confidence-intervals of Representation quotients
#Confidence-intervals around sample proportion
prop.test(x = Observation[2], n = sum(Observation), conf.level = .95)
```

```
## 
##  1-sample proportions test with continuity correction
## 
## data:  Observation[2] out of sum(Observation), null probability 0.5
## X-squared = 7541.6, df = 1, p-value < 2.2e-16
## alternative hypothesis: true p is not equal to 0.5
## 95 percent confidence interval:
##  0.01872596 0.02515365
## sample estimates:
##          p 
## 0.02171276
```

```
#Confidence-intervals around sample proportion relative to expected proportion
round(prop.test(x = Observation[2], n = sum(Observation), conf.level = .95)$estimate/expected_ratio_6574,2)
```

```
##    p 
## 0.27
```

```
round(as.vector(prop.test(x = Observation[2], n = sum(Observation), conf.level = .95)$conf.int)/expected_ratio_6574,2)
```

```
## [1] 0.23 0.31
```

```
#Confidence-intervals around sample proportion
prop.test(x = Observation[3], n = sum(Observation), conf.level = .95)
```

```
## 
##  1-sample proportions test with continuity correction
## 
## data:  Observation[3] out of sum(Observation), null probability 0.5
## X-squared = 8090.7, df = 1, p-value < 2.2e-16
## alternative hypothesis: true p is not equal to 0.5
## 95 percent confidence interval:
##  0.003309074 0.006390114
## sample estimates:
##           p 
## 0.004609413
```

```
#Confidence-intervals around sample proportion relative to expected proportion
round(prop.test(x = Observation[3], n = sum(Observation), conf.level = .95)$estimate/expected_ratio_75plus,2)
```

```
##    p 
## 0.05
```

```
round(as.vector(prop.test(x = Observation[3], n = sum(Observation), conf.level = .95)$conf.int)/expected_ratio_75plus,2)
```

```
## [1] 0.04 0.08
```

```
###########################
#Major Depressive Disorder#
###########################
prevalence_1834 <- as.numeric(Prevalence_estimates[Prevalence_estimates$Diagnosis == "Major Depressive Disorder", "1834y"])
prevalence_3549 <- as.numeric(Prevalence_estimates[Prevalence_estimates$Diagnosis == "Major Depressive Disorder", "3549y"])
prevalence_5064 <- as.numeric(Prevalence_estimates[Prevalence_estimates$Diagnosis == "Major Depressive Disorder", "5064y"])

prevalence_1864 <- (census_amount_1834/sum(census_amount_1834,census_amount_3549, census_amount_5064))*prevalence_1834+
  (census_amount_3549/sum(census_amount_1834,census_amount_3549, census_amount_5064))*prevalence_3549+
  (census_amount_5064/sum(census_amount_1834,census_amount_3549, census_amount_5064))*prevalence_5064

prevalence_6574 <- as.numeric(Prevalence_estimates[Prevalence_estimates$Diagnosis == "Major Depressive Disorder", "6574y"])
prevalence_75plus <- as.numeric(Prevalence_estimates[Prevalence_estimates$Diagnosis == "Major Depressive Disorder", "75yplus"])

#Ratios in reference population
expected_ratio_1864 <- (census_amount_1864*prevalence_1864)/((census_amount_1864*prevalence_1864)+(census_amount_6574*prevalence_6574)+(census_amount_75plus*prevalence_75plus))
expected_ratio_6574 <- (census_amount_6574*prevalence_6574)/((census_amount_1864*prevalence_1864)+(census_amount_6574*prevalence_6574)+(census_amount_75plus*prevalence_75plus))
expected_ratio_75plus <- (census_amount_75plus*prevalence_75plus)/((census_amount_1864*prevalence_1864)+(census_amount_6574*prevalence_6574)+(census_amount_75plus*prevalence_75plus))

expected_ratios <- c(expected_ratio_1864,
                     expected_ratio_6574,
                     expected_ratio_75plus)
round(expected_ratios*100,1)
```

```
## [1] 82.4  8.6  9.0
```

```
KODAP_data_complete_MDD <- KODAP_data_complete[KODAP_data_complete$ICD1_pre_recode %in% c("F32 Depressive Episode",
                                                                                          "F33 Rezidivierende depressive Störung",
                                                                                          "F33.4 Rezidivierende depressive Störung, gegenwärtig remittiert") |
                                                 KODAP_data_complete$ICD2_pre_recode %in% c("F32 Depressive Episode",
                                                                                            "F33 Rezidivierende depressive Störung",
                                                                                            "F33.4 Rezidivierende depressive Störung, gegenwärtig remittiert") |
                                                 KODAP_data_complete$ICD3_pre_recode %in% c("F32 Depressive Episode",
                                                                                            "F33 Rezidivierende depressive Störung",
                                                                                            "F33.4 Rezidivierende depressive Störung, gegenwärtig remittiert") |
                                                 KODAP_data_complete$ICD4_pre_recode %in% c("F32 Depressive Episode",
                                                                                            "F33 Rezidivierende depressive Störung",
                                                                                            "F33.4 Rezidivierende depressive Störung, gegenwärtig remittiert")| 
                                                 KODAP_data_complete$ICD5_pre_recode %in% c("F32 Depressive Episode",
                                                                                            "F33 Rezidivierende depressive Störung",
                                                                                            "F33.4 Rezidivierende depressive Störung, gegenwärtig remittiert"),]

length(KODAP_data_complete_MDD$Patient_ID)
```

```
## [1] 7460
```

```
#Observed vs. expected: 
Observation <- as.vector(table(KODAP_data_complete_MDD$Age_Stepped))
Expected <- expected_ratios*length(KODAP_data_complete_MDD$Patient_ID)

Observation
```

```
## [1] 7261  163   36
```

```
round(Observation/length(KODAP_data_complete_MDD$Patient_ID)*100,1)
```

```
## [1] 97.3  2.2  0.5
```

```
# Chi-squared test for given probabilities
test <- chisq.test(Observation, p = expected_ratios)
test
```

```
## 
##  Chi-squared test for given probabilities
## 
## data:  Observation
## X-squared = 1162.4, df = 2, p-value < 2.2e-16
```

```
# Post-hoc binomial-test for each category with Bonferroni-correction
binom.test(Observation[1], sum(Observation), expected_ratios[1])$p.value*3
```

```
## [1] 0
```

```
binom.test(Observation[2], sum(Observation), expected_ratios[2])$p.value*3
```

```
## [1] 9.582918e-120
```

```
binom.test(Observation[3], sum(Observation), expected_ratios[3])$p.value*3
```

```
## [1] 5.973743e-244
```

```
#Estimating underrepresentation compared to reference population
#Representation quotients
Observation/Expected
```

```
## [1] 1.18153500 0.25357980 0.05358668
```

```
#Confidence-intervals of Representation quotients
#Confidence-intervals around sample proportion
prop.test(x = Observation[2], n = sum(Observation), conf.level = .95)
```

```
## 
##  1-sample proportions test with continuity correction
## 
## data:  Observation[2] out of sum(Observation), null probability 0.5
## X-squared = 6820.3, df = 1, p-value < 2.2e-16
## alternative hypothesis: true p is not equal to 0.5
## 95 percent confidence interval:
##  0.01870821 0.02549363
## sample estimates:
##          p 
## 0.02184987
```

```
#Confidence-intervals around sample proportion relative to expected proportion
round(prop.test(x = Observation[2], n = sum(Observation), conf.level = .95)$estimate/expected_ratio_6574,2)
```

```
##    p 
## 0.25
```

```
round(as.vector(prop.test(x = Observation[2], n = sum(Observation), conf.level = .95)$conf.int)/expected_ratio_6574,2)
```

```
## [1] 0.22 0.30
```

```
#Confidence-intervals around sample proportion
prop.test(x = Observation[3], n = sum(Observation), conf.level = .95)
```

```
## 
##  1-sample proportions test with continuity correction
## 
## data:  Observation[3] out of sum(Observation), null probability 0.5
## X-squared = 7314.7, df = 1, p-value < 2.2e-16
## alternative hypothesis: true p is not equal to 0.5
## 95 percent confidence interval:
##  0.003431670 0.006750952
## sample estimates:
##           p 
## 0.004825737
```

```
#Confidence-intervals around sample proportion relative to expected proportion
round(prop.test(x = Observation[3], n = sum(Observation), conf.level = .95)$estimate/expected_ratio_75plus,2)
```

```
##    p 
## 0.05
```

```
round(as.vector(prop.test(x = Observation[3], n = sum(Observation), conf.level = .95)$conf.int)/expected_ratio_75plus,2)
```

```
## [1] 0.04 0.07
```

```
###########################
#########Dysthymia#########
###########################
prevalence_1834 <- as.numeric(Prevalence_estimates[Prevalence_estimates$Diagnosis == "Dysthymia", "1834y"])
prevalence_3549 <- as.numeric(Prevalence_estimates[Prevalence_estimates$Diagnosis == "Dysthymia", "3549y"])
prevalence_5064 <- as.numeric(Prevalence_estimates[Prevalence_estimates$Diagnosis == "Dysthymia", "5064y"])

prevalence_1864 <- (census_amount_1834/sum(census_amount_1834,census_amount_3549, census_amount_5064))*prevalence_1834+
  (census_amount_3549/sum(census_amount_1834,census_amount_3549, census_amount_5064))*prevalence_3549+
  (census_amount_5064/sum(census_amount_1834,census_amount_3549, census_amount_5064))*prevalence_5064

prevalence_6574 <- as.numeric(Prevalence_estimates[Prevalence_estimates$Diagnosis == "Dysthymia", "6574y"])
prevalence_75plus <- as.numeric(Prevalence_estimates[Prevalence_estimates$Diagnosis == "Dysthymia", "75yplus"])

#Ratios in reference population
expected_ratio_1864 <- (census_amount_1864*prevalence_1864)/((census_amount_1864*prevalence_1864)+(census_amount_6574*prevalence_6574)+(census_amount_75plus*prevalence_75plus))
expected_ratio_6574 <- (census_amount_6574*prevalence_6574)/((census_amount_1864*prevalence_1864)+(census_amount_6574*prevalence_6574)+(census_amount_75plus*prevalence_75plus))
expected_ratio_75plus <- (census_amount_75plus*prevalence_75plus)/((census_amount_1864*prevalence_1864)+(census_amount_6574*prevalence_6574)+(census_amount_75plus*prevalence_75plus))

expected_ratios <- c(expected_ratio_1864,
                     expected_ratio_6574,
                     expected_ratio_75plus)
round(expected_ratios*100,1)
```

```
## [1] 74.6 12.4 13.0
```

```
KODAP_data_complete_Dysthymia <- KODAP_data_complete[KODAP_data_complete$ICD1_pre_clean %in% c("F34.1", "F34.10") |
                                                       KODAP_data_complete$ICD2_pre_clean %in% c("F34.1", "F34.10") |
                                                       KODAP_data_complete$ICD3_pre_clean %in% c("F34.1", "F34.10") | 
                                                       KODAP_data_complete$ICD4_pre_clean %in% c("F34.1", "F34.10") | 
                                                       KODAP_data_complete$ICD5_pre_clean %in% c("F34.1", "F34.10"),]


length(KODAP_data_complete_Dysthymia$Patient_ID)
```

```
## [1] 958
```

```
#Observed vs. expected: 
Observation <- as.vector(table(KODAP_data_complete_Dysthymia$Age_Stepped))
Expected <- expected_ratios*length(KODAP_data_complete_Dysthymia$Patient_ID)

Observation
```

```
## [1] 935  22   1
```

```
round(Observation/length(KODAP_data_complete_Dysthymia$Patient_ID)*100,1)
```

```
## [1] 97.6  2.3  0.1
```

```
# Chi-squared test for given probabilities
test <- chisq.test(Observation, p = expected_ratios)
test
```

```
## 
##  Chi-squared test for given probabilities
## 
## data:  Observation
## X-squared = 269.65, df = 2, p-value < 2.2e-16
```

```
# Post-hoc binomial-test for each category with Bonferroni-correction
binom.test(Observation[1], sum(Observation), expected_ratios[1])$p.value*3
```

```
## [1] 9.823106e-87
```

```
binom.test(Observation[2], sum(Observation), expected_ratios[2])$p.value*3
```

```
## [1] 2.899135e-29
```

```
binom.test(Observation[3], sum(Observation), expected_ratios[3])$p.value*3
```

```
## [1] 8.711903e-56
```

```
#Estimating underrepresentation compared to reference population
#Representation quotients
Observation/Expected
```

```
## [1] 1.308640900 0.184762252 0.008035592
```

```
#Confidence-intervals of Representation quotients
#Confidence-intervals around sample proportion
prop.test(x = Observation[2], n = sum(Observation), conf.level = .95)
```

```
## 
##  1-sample proportions test with continuity correction
## 
## data:  Observation[2] out of sum(Observation), null probability 0.5
## X-squared = 870.11, df = 1, p-value < 2.2e-16
## alternative hypothesis: true p is not equal to 0.5
## 95 percent confidence interval:
##  0.01479694 0.03514765
## sample estimates:
##          p 
## 0.02296451
```

```
#Confidence-intervals around sample proportion relative to expected proportion
round(prop.test(x = Observation[2], n = sum(Observation), conf.level = .95)$estimate/expected_ratio_6574,2)
```

```
##    p 
## 0.18
```

```
round(as.vector(prop.test(x = Observation[2], n = sum(Observation), conf.level = .95)$conf.int)/expected_ratio_6574,2)
```

```
## [1] 0.12 0.28
```

```
#Confidence-intervals around sample proportion
prop.test(x = Observation[3], n = sum(Observation), conf.level = .95)
```

```
## 
##  1-sample proportions test with continuity correction
## 
## data:  Observation[3] out of sum(Observation), null probability 0.5
## X-squared = 952.01, df = 1, p-value < 2.2e-16
## alternative hypothesis: true p is not equal to 0.5
## 95 percent confidence interval:
##  5.449108e-05 6.751188e-03
## sample estimates:
##           p 
## 0.001043841
```

```
#Confidence-intervals around sample proportion relative to expected proportion
round(prop.test(x = Observation[3], n = sum(Observation), conf.level = .95)$estimate/expected_ratio_75plus,2)
```

```
##    p 
## 0.01
```

```
round(as.vector(prop.test(x = Observation[3], n = sum(Observation), conf.level = .95)$conf.int)/expected_ratio_75plus,2)
```

```
## [1] 0.00 0.05
```

```
###########################
##Any anxiety disorder#####
###########################
prevalence_1834 <- as.numeric(Prevalence_estimates[Prevalence_estimates$Diagnosis == "Any anxiety disorder", "1834y"])
prevalence_3549 <- as.numeric(Prevalence_estimates[Prevalence_estimates$Diagnosis == "Any anxiety disorder", "3549y"])
prevalence_5064 <- as.numeric(Prevalence_estimates[Prevalence_estimates$Diagnosis == "Any anxiety disorder", "5064y"])

prevalence_1864 <- (census_amount_1834/sum(census_amount_1834,census_amount_3549, census_amount_5064))*prevalence_1834+
  (census_amount_3549/sum(census_amount_1834,census_amount_3549, census_amount_5064))*prevalence_3549+
  (census_amount_5064/sum(census_amount_1834,census_amount_3549, census_amount_5064))*prevalence_5064

prevalence_6574 <- as.numeric(Prevalence_estimates[Prevalence_estimates$Diagnosis == "Any anxiety disorder", "6574y"])
prevalence_75plus <- as.numeric(Prevalence_estimates[Prevalence_estimates$Diagnosis == "Any anxiety disorder", "75yplus"])

#Ratios in reference population
expected_ratio_1864 <- (census_amount_1864*prevalence_1864)/((census_amount_1864*prevalence_1864)+(census_amount_6574*prevalence_6574)+(census_amount_75plus*prevalence_75plus))
expected_ratio_6574 <- (census_amount_6574*prevalence_6574)/((census_amount_1864*prevalence_1864)+(census_amount_6574*prevalence_6574)+(census_amount_75plus*prevalence_75plus))
expected_ratio_75plus <- (census_amount_75plus*prevalence_75plus)/((census_amount_1864*prevalence_1864)+(census_amount_6574*prevalence_6574)+(census_amount_75plus*prevalence_75plus))

expected_ratios <- c(expected_ratio_1864,
                     expected_ratio_6574,
                     expected_ratio_75plus)
round(expected_ratios*100,1)
```

```
## [1] 80.6  9.5  9.9
```

```
KODAP_data_complete_any_anx_disorder <- KODAP_data_complete[KODAP_data_complete$ICD1_pre_recode %in% c("F40.0X F41.0 Agoraphobie/Panikstörung",
                                                                                                       "F40.1 Soziale Phobie",
                                                                                                       "F40.2 Spezifische Phobie",
                                                                                                       "F41.1 Generalisierte Angststörung",
                                                                                                       "F41.X F40.9 Andere phobische oder Angststörungen") |
                                                              KODAP_data_complete$ICD2_pre_recode %in% c("F40.0X F41.0 Agoraphobie/Panikstörung",
                                                                                                         "F40.1 Soziale Phobie",
                                                                                                         "F40.2 Spezifische Phobie",
                                                                                                         "F41.1 Generalisierte Angststörung",
                                                                                                         "F41.X F40.9 Andere phobische oder Angststörungen") | 
                                                              KODAP_data_complete$ICD3_pre_recode %in% c("F40.0X F41.0 Agoraphobie/Panikstörung",
                                                                                                         "F40.1 Soziale Phobie",
                                                                                                         "F40.2 Spezifische Phobie",
                                                                                                         "F41.1 Generalisierte Angststörung",
                                                                                                         "F41.X F40.9 Andere phobische oder Angststörungen") |
                                                              KODAP_data_complete$ICD4_pre_recode %in% c("F40.0X F41.0 Agoraphobie/Panikstörung",
                                                                                                         "F40.1 Soziale Phobie",
                                                                                                         "F40.2 Spezifische Phobie",
                                                                                                         "F41.1 Generalisierte Angststörung",
                                                                                                         "F41.X F40.9 Andere phobische oder Angststörungen") | 
                                                              KODAP_data_complete$ICD5_pre_recode %in% c("F40.0X F41.0 Agoraphobie/Panikstörung",
                                                                                                         "F40.1 Soziale Phobie",
                                                                                                         "F40.2 Spezifische Phobie",
                                                                                                         "F41.1 Generalisierte Angststörung",
                                                                                                         "F41.X F40.9 Andere phobische oder Angststörungen"),]

length(KODAP_data_complete_any_anx_disorder$Patient_ID)
```

```
## [1] 4453
```

```
#Observed vs. expected: 
Observation <- as.vector(table(KODAP_data_complete_any_anx_disorder$Age_Stepped))
Expected <- expected_ratios*length(KODAP_data_complete_any_anx_disorder$Patient_ID)

Observation
```

```
## [1] 4318  106   29
```

```
round(Observation/length(KODAP_data_complete_any_anx_disorder$Patient_ID)*100,1)
```

```
## [1] 97.0  2.4  0.7
```

```
# Chi-squared test for given probabilities
test <- chisq.test(Observation, p = expected_ratios)
test
```

```
## 
##  Chi-squared test for given probabilities
## 
## data:  Observation
## X-squared = 772.5, df = 2, p-value < 2.2e-16
```

```
# Post-hoc binomial-test for each category with Bonferroni-correction
binom.test(Observation[1], sum(Observation), expected_ratios[1])$p.value*3
```

```
## [1] 5.244667e-240
```

```
binom.test(Observation[2], sum(Observation), expected_ratios[2])$p.value*3
```

```
## [1] 1.363829e-80
```

```
binom.test(Observation[3], sum(Observation), expected_ratios[3])$p.value*3
```

```
## [1] 3.210344e-155
```

```
#Estimating underrepresentation compared to reference population
#Representation quotients
Observation/Expected
```

```
## [1] 1.20357702 0.25051327 0.06557679
```

```
#Confidence-intervals of Representation quotients
#Confidence-intervals around sample proportion
prop.test(x = Observation[2], n = sum(Observation), conf.level = .95)
```

```
## 
##  1-sample proportions test with continuity correction
## 
## data:  Observation[2] out of sum(Observation), null probability 0.5
## X-squared = 4037.2, df = 1, p-value < 2.2e-16
## alternative hypothesis: true p is not equal to 0.5
## 95 percent confidence interval:
##  0.01961854 0.02883120
## sample estimates:
##          p 
## 0.02380418
```

```
#Confidence-intervals around sample proportion relative to expected proportion
round(prop.test(x = Observation[2], n = sum(Observation), conf.level = .95)$estimate/expected_ratio_6574,2)
```

```
##    p 
## 0.25
```

```
round(as.vector(prop.test(x = Observation[2], n = sum(Observation), conf.level = .95)$conf.int)/expected_ratio_6574,2)
```

```
## [1] 0.21 0.30
```

```
#Confidence-intervals around sample proportion
prop.test(x = Observation[3], n = sum(Observation), conf.level = .95)
```

```
## 
##  1-sample proportions test with continuity correction
## 
## data:  Observation[3] out of sum(Observation), null probability 0.5
## X-squared = 4335.8, df = 1, p-value < 2.2e-16
## alternative hypothesis: true p is not equal to 0.5
## 95 percent confidence interval:
##  0.004446095 0.009469305
## sample estimates:
##           p 
## 0.006512464
```

```
#Confidence-intervals around sample proportion relative to expected proportion
round(prop.test(x = Observation[3], n = sum(Observation), conf.level = .95)$estimate/expected_ratio_75plus,2)
```

```
##    p 
## 0.07
```

```
round(as.vector(prop.test(x = Observation[3], n = sum(Observation), conf.level = .95)$conf.int)/expected_ratio_75plus,2)
```

```
## [1] 0.04 0.10
```

```
#################################
##Panic Disorder/Agoraphobia#####
#################################
prevalence_1834 <- as.numeric(Prevalence_estimates[Prevalence_estimates$Diagnosis == "Panic disorder/Agoraphobia", "1834y"])
prevalence_3549 <- as.numeric(Prevalence_estimates[Prevalence_estimates$Diagnosis == "Panic disorder/Agoraphobia", "3549y"])
prevalence_5064 <- as.numeric(Prevalence_estimates[Prevalence_estimates$Diagnosis == "Panic disorder/Agoraphobia", "5064y"])

prevalence_1864 <- (census_amount_1834/sum(census_amount_1834,census_amount_3549, census_amount_5064))*prevalence_1834+
  (census_amount_3549/sum(census_amount_1834,census_amount_3549, census_amount_5064))*prevalence_3549+
  (census_amount_5064/sum(census_amount_1834,census_amount_3549, census_amount_5064))*prevalence_5064

prevalence_6574 <- as.numeric(Prevalence_estimates[Prevalence_estimates$Diagnosis == "Panic disorder/Agoraphobia", "6574y"])
prevalence_75plus <- as.numeric(Prevalence_estimates[Prevalence_estimates$Diagnosis == "Panic disorder/Agoraphobia", "75yplus"])

#Ratios in reference population
expected_ratio_1864 <- (census_amount_1864*prevalence_1864)/((census_amount_1864*prevalence_1864)+(census_amount_6574*prevalence_6574)+(census_amount_75plus*prevalence_75plus))
expected_ratio_6574 <- (census_amount_6574*prevalence_6574)/((census_amount_1864*prevalence_1864)+(census_amount_6574*prevalence_6574)+(census_amount_75plus*prevalence_75plus))
expected_ratio_75plus <- (census_amount_75plus*prevalence_75plus)/((census_amount_1864*prevalence_1864)+(census_amount_6574*prevalence_6574)+(census_amount_75plus*prevalence_75plus))

expected_ratios <- c(expected_ratio_1864,
                     expected_ratio_6574,
                     expected_ratio_75plus)
round(expected_ratios*100,1)
```

```
## [1] 76.7 11.4 11.9
```

```
KODAP_data_complete_PanicAgora <- KODAP_data_complete[KODAP_data_complete$ICD1_pre_recode %in% c("F40.0X F41.0 Agoraphobie/Panikstörung") |
                                                        KODAP_data_complete$ICD2_pre_recode %in% c("F40.0X F41.0 Agoraphobie/Panikstörung") | 
                                                        KODAP_data_complete$ICD3_pre_recode %in% c("F40.0X F41.0 Agoraphobie/Panikstörung") |
                                                        KODAP_data_complete$ICD4_pre_recode %in% c("F40.0X F41.0 Agoraphobie/Panikstörung") | 
                                                        KODAP_data_complete$ICD5_pre_recode %in% c("F40.0X F41.0 Agoraphobie/Panikstörung"),]

length(KODAP_data_complete_PanicAgora$Patient_ID)
```

```
## [1] 1456
```

```
#Observed vs. expected: 
Observation <- as.vector(table(KODAP_data_complete_PanicAgora$Age_Stepped))
Expected <- expected_ratios*length(KODAP_data_complete_PanicAgora$Patient_ID)

Observation
```

```
## [1] 1400   47    9
```

```
round(Observation/length(KODAP_data_complete_PanicAgora$Patient_ID)*100,1)
```

```
## [1] 96.2  3.2  0.6
```

```
# Chi-squared test for given probabilities
test <- chisq.test(Observation, p = expected_ratios)
test
```

```
## 
##  Chi-squared test for given probabilities
## 
## data:  Observation
## X-squared = 312.33, df = 2, p-value < 2.2e-16
```

```
# Post-hoc binomial-test for each category with Bonferroni-correction
binom.test(Observation[1], sum(Observation), expected_ratios[1])$p.value*3
```

```
## [1] 1.114495e-94
```

```
binom.test(Observation[2], sum(Observation), expected_ratios[2])$p.value*3
```

```
## [1] 3.321778e-29
```

```
binom.test(Observation[3], sum(Observation), expected_ratios[3])$p.value*3
```

```
## [1] 6.078787e-65
```

```
#Estimating underrepresentation compared to reference population
#Representation quotients
Observation/Expected
```

```
## [1] 1.25323710 0.28363345 0.05196721
```

```
#Confidence-intervals of Representation quotients
#Confidence-intervals around sample proportion
prop.test(x = Observation[2], n = sum(Observation), conf.level = .95)
```

```
## 
##  1-sample proportions test with continuity correction
## 
## data:  Observation[2] out of sum(Observation), null probability 0.5
## X-squared = 1272.2, df = 1, p-value < 2.2e-16
## alternative hypothesis: true p is not equal to 0.5
## 95 percent confidence interval:
##  0.02406506 0.04304906
## sample estimates:
##          p 
## 0.03228022
```

```
#Confidence-intervals around sample proportion relative to expected proportion
round(prop.test(x = Observation[2], n = sum(Observation), conf.level = .95)$estimate/expected_ratio_6574,2)
```

```
##    p 
## 0.28
```

```
round(as.vector(prop.test(x = Observation[2], n = sum(Observation), conf.level = .95)$conf.int)/expected_ratio_6574,2)
```

```
## [1] 0.21 0.38
```

```
#Confidence-intervals around sample proportion
prop.test(x = Observation[3], n = sum(Observation), conf.level = .95)
```

```
## 
##  1-sample proportions test with continuity correction
## 
## data:  Observation[3] out of sum(Observation), null probability 0.5
## X-squared = 1418.2, df = 1, p-value < 2.2e-16
## alternative hypothesis: true p is not equal to 0.5
## 95 percent confidence interval:
##  0.003019616 0.012152642
## sample estimates:
##           p 
## 0.006181319
```

```
#Confidence-intervals around sample proportion relative to expected proportion
round(prop.test(x = Observation[3], n = sum(Observation), conf.level = .95)$estimate/expected_ratio_75plus,2)
```

```
##    p 
## 0.05
```

```
round(as.vector(prop.test(x = Observation[3], n = sum(Observation), conf.level = .95)$conf.int)/expected_ratio_75plus,2)
```

```
## [1] 0.03 0.10
```

```
#################################
##########Social phobia##########
#################################
prevalence_1834 <- as.numeric(Prevalence_estimates[Prevalence_estimates$Diagnosis == "Social phobia", "1834y"])
prevalence_3549 <- as.numeric(Prevalence_estimates[Prevalence_estimates$Diagnosis == "Social phobia", "3549y"])
prevalence_5064 <- as.numeric(Prevalence_estimates[Prevalence_estimates$Diagnosis == "Social phobia", "5064y"])

prevalence_1864 <- (census_amount_1834/sum(census_amount_1834,census_amount_3549, census_amount_5064))*prevalence_1834+
  (census_amount_3549/sum(census_amount_1834,census_amount_3549, census_amount_5064))*prevalence_3549+
  (census_amount_5064/sum(census_amount_1834,census_amount_3549, census_amount_5064))*prevalence_5064

prevalence_6574 <- as.numeric(Prevalence_estimates[Prevalence_estimates$Diagnosis == "Social phobia", "6574y"])
prevalence_75plus <- as.numeric(Prevalence_estimates[Prevalence_estimates$Diagnosis == "Social phobia", "75yplus"])

#Ratios in reference population
expected_ratio_1864 <- (census_amount_1864*prevalence_1864)/((census_amount_1864*prevalence_1864)+(census_amount_6574*prevalence_6574)+(census_amount_75plus*prevalence_75plus))
expected_ratio_6574 <- (census_amount_6574*prevalence_6574)/((census_amount_1864*prevalence_1864)+(census_amount_6574*prevalence_6574)+(census_amount_75plus*prevalence_75plus))
expected_ratio_75plus <- (census_amount_75plus*prevalence_75plus)/((census_amount_1864*prevalence_1864)+(census_amount_6574*prevalence_6574)+(census_amount_75plus*prevalence_75plus))

expected_ratios <- c(expected_ratio_1864,
                     expected_ratio_6574,
                     expected_ratio_75plus)
round(expected_ratios*100,1)
```

```
## [1] 92.8  3.5  3.7
```

```
KODAP_data_complete_socialphobia <- KODAP_data_complete[KODAP_data_complete$ICD1_pre_recode %in% c("F40.1 Soziale Phobie") |
                                                          KODAP_data_complete$ICD2_pre_recode %in% c("F40.1 Soziale Phobie") | 
                                                          KODAP_data_complete$ICD3_pre_recode %in% c("F40.1 Soziale Phobie") | 
                                                          KODAP_data_complete$ICD4_pre_recode %in% c("F40.1 Soziale Phobie") |
                                                          KODAP_data_complete$ICD5_pre_recode %in% c("F40.1 Soziale Phobie"),]


length(KODAP_data_complete_socialphobia$Patient_ID)
```

```
## [1] 1850
```

```
#Observed vs. expected: 
Observation <- as.vector(table(KODAP_data_complete_socialphobia$Age_Stepped))
Expected <- expected_ratios*length(KODAP_data_complete_socialphobia$Patient_ID)

Observation
```

```
## [1] 1830   16    4
```

```
round(Observation/length(KODAP_data_complete_socialphobia$Patient_ID)*100,1)
```

```
## [1] 98.9  0.9  0.2
```

```
# Chi-squared test for given probabilities
test <- chisq.test(Observation, p = expected_ratios)
test
```

```
## 
##  Chi-squared test for given probabilities
## 
## data:  Observation
## X-squared = 103.92, df = 2, p-value < 2.2e-16
```

```
# Post-hoc binomial-test for each category with Bonferroni-correction
binom.test(Observation[1], sum(Observation), expected_ratios[1])$p.value*3
```

```
## [1] 7.089412e-35
```

```
binom.test(Observation[2], sum(Observation), expected_ratios[2])$p.value*3
```

```
## [1] 1.300956e-12
```

```
binom.test(Observation[3], sum(Observation), expected_ratios[3])$p.value*3
```

```
## [1] 6.342283e-24
```

```
#Estimating underrepresentation compared to reference population
#Representation quotients
Observation/Expected
```

```
## [1] 1.06542385 0.24719537 0.05912997
```

```
#Confidence-intervals of Representation quotients
#Confidence-intervals around sample proportion
prop.test(x = Observation[2], n = sum(Observation), conf.level = .95)
```

```
## 
##  1-sample proportions test with continuity correction
## 
## data:  Observation[2] out of sum(Observation), null probability 0.5
## X-squared = 1784.6, df = 1, p-value < 2.2e-16
## alternative hypothesis: true p is not equal to 0.5
## 95 percent confidence interval:
##  0.005124665 0.014335622
## sample estimates:
##           p 
## 0.008648649
```

```
#Confidence-intervals around sample proportion relative to expected proportion
round(prop.test(x = Observation[2], n = sum(Observation), conf.level = .95)$estimate/expected_ratio_6574,2)
```

```
##    p 
## 0.25
```

```
round(as.vector(prop.test(x = Observation[2], n = sum(Observation), conf.level = .95)$conf.int)/expected_ratio_6574,2)
```

```
## [1] 0.15 0.41
```

```
#Confidence-intervals around sample proportion
prop.test(x = Observation[3], n = sum(Observation), conf.level = .95)
```

```
## 
##  1-sample proportions test with continuity correction
## 
## data:  Observation[3] out of sum(Observation), null probability 0.5
## X-squared = 1832, df = 1, p-value < 2.2e-16
## alternative hypothesis: true p is not equal to 0.5
## 95 percent confidence interval:
##  0.0006928545 0.0059315037
## sample estimates:
##           p 
## 0.002162162
```

```
#Confidence-intervals around sample proportion relative to expected proportion
round(prop.test(x = Observation[3], n = sum(Observation), conf.level = .95)$estimate/expected_ratio_75plus,2)
```

```
##    p 
## 0.06
```

```
round(as.vector(prop.test(x = Observation[3], n = sum(Observation), conf.level = .95)$conf.int)/expected_ratio_75plus,2)
```

```
## [1] 0.02 0.16
```

```
#################################
##########Specific phobia##########
#################################
prevalence_1834 <- as.numeric(Prevalence_estimates[Prevalence_estimates$Diagnosis == "Specific phobias", "1834y"])
prevalence_3549 <- as.numeric(Prevalence_estimates[Prevalence_estimates$Diagnosis == "Specific phobias", "3549y"])
prevalence_5064 <- as.numeric(Prevalence_estimates[Prevalence_estimates$Diagnosis == "Specific phobias", "5064y"])

prevalence_1864 <- (census_amount_1834/sum(census_amount_1834,census_amount_3549, census_amount_5064))*prevalence_1834+
  (census_amount_3549/sum(census_amount_1834,census_amount_3549, census_amount_5064))*prevalence_3549+
  (census_amount_5064/sum(census_amount_1834,census_amount_3549, census_amount_5064))*prevalence_5064

prevalence_6574 <- as.numeric(Prevalence_estimates[Prevalence_estimates$Diagnosis == "Specific phobias", "6574y"])
prevalence_75plus <- as.numeric(Prevalence_estimates[Prevalence_estimates$Diagnosis == "Specific phobias", "75yplus"])

#Ratios in reference population
expected_ratio_1864 <- (census_amount_1864*prevalence_1864)/((census_amount_1864*prevalence_1864)+(census_amount_6574*prevalence_6574)+(census_amount_75plus*prevalence_75plus))
expected_ratio_6574 <- (census_amount_6574*prevalence_6574)/((census_amount_1864*prevalence_1864)+(census_amount_6574*prevalence_6574)+(census_amount_75plus*prevalence_75plus))
expected_ratio_75plus <- (census_amount_75plus*prevalence_75plus)/((census_amount_1864*prevalence_1864)+(census_amount_6574*prevalence_6574)+(census_amount_75plus*prevalence_75plus))

expected_ratios <- c(expected_ratio_1864,
                     expected_ratio_6574,
                     expected_ratio_75plus)
round(expected_ratios*100,1)
```

```
## [1] 78.4 10.6 11.0
```

```
KODAP_data_complete_specificphobia <- KODAP_data_complete[KODAP_data_complete$ICD1_pre_recode %in% c("F40.2 Spezifische Phobie") |
                                                            KODAP_data_complete$ICD2_pre_recode %in% c("F40.2 Spezifische Phobie") | 
                                                            KODAP_data_complete$ICD3_pre_recode %in% c("F40.2 Spezifische Phobie") |
                                                            KODAP_data_complete$ICD4_pre_recode %in% c("F40.2 Spezifische Phobie") | 
                                                            KODAP_data_complete$ICD5_pre_recode %in% c("F40.2 Spezifische Phobie"),]


length(KODAP_data_complete_specificphobia$Patient_ID)
```

```
## [1] 703
```

```
#Observed vs. expected: 
Observation <- as.vector(table(KODAP_data_complete_specificphobia$Age_Stepped))
Expected <- expected_ratios*length(KODAP_data_complete_specificphobia$Patient_ID)

Observation
```

```
## [1] 678  22   3
```

```
round(Observation/length(KODAP_data_complete_specificphobia$Patient_ID)*100,1)
```

```
## [1] 96.4  3.1  0.4
```

```
# Chi-squared test for given probabilities
test <- chisq.test(Observation, p = expected_ratios)
test
```

```
## 
##  Chi-squared test for given probabilities
## 
## data:  Observation
## X-squared = 137.53, df = 2, p-value < 2.2e-16
```

```
# Post-hoc binomial-test for each category with Bonferroni-correction
binom.test(Observation[1], sum(Observation), expected_ratios[1])$p.value*3
```

```
## [1] 2.072222e-42
```

```
binom.test(Observation[2], sum(Observation), expected_ratios[2])$p.value*3
```

```
## [1] 6.419946e-13
```

```
binom.test(Observation[3], sum(Observation), expected_ratios[3])$p.value*3
```

```
## [1] 1.446374e-30
```

```
#Estimating underrepresentation compared to reference population
#Representation quotients
Observation/Expected
```

```
## [1] 1.22992128 0.29650350 0.03868617
```

```
#Confidence-intervals of Representation quotients
#Confidence-intervals around sample proportion
prop.test(x = Observation[2], n = sum(Observation), conf.level = .95)
```

```
## 
##  1-sample proportions test with continuity correction
## 
## data:  Observation[2] out of sum(Observation), null probability 0.5
## X-squared = 615.88, df = 1, p-value < 2.2e-16
## alternative hypothesis: true p is not equal to 0.5
## 95 percent confidence interval:
##  0.02018695 0.04777189
## sample estimates:
##          p 
## 0.03129445
```

```
#Confidence-intervals around sample proportion relative to expected proportion
round(prop.test(x = Observation[2], n = sum(Observation), conf.level = .95)$estimate/expected_ratio_6574,2)
```

```
##   p 
## 0.3
```

```
round(as.vector(prop.test(x = Observation[2], n = sum(Observation), conf.level = .95)$conf.int)/expected_ratio_6574,2)
```

```
## [1] 0.19 0.45
```

```
#Confidence-intervals around sample proportion
prop.test(x = Observation[3], n = sum(Observation), conf.level = .95)
```

```
## 
##  1-sample proportions test with continuity correction
## 
## data:  Observation[3] out of sum(Observation), null probability 0.5
## X-squared = 689.07, df = 1, p-value < 2.2e-16
## alternative hypothesis: true p is not equal to 0.5
## 95 percent confidence interval:
##  0.001102765 0.013513634
## sample estimates:
##           p 
## 0.004267425
```

```
#Confidence-intervals around sample proportion relative to expected proportion
round(prop.test(x = Observation[3], n = sum(Observation), conf.level = .95)$estimate/expected_ratio_75plus,2)
```

```
##    p 
## 0.04
```

```
round(as.vector(prop.test(x = Observation[3], n = sum(Observation), conf.level = .95)$conf.int)/expected_ratio_75plus,2)
```

```
## [1] 0.01 0.12
```

```
################################################
##########Generalized Anxiety Disorder##########
################################################
prevalence_1834 <- as.numeric(Prevalence_estimates[Prevalence_estimates$Diagnosis == "GAD", "1834y"])
prevalence_3549 <- as.numeric(Prevalence_estimates[Prevalence_estimates$Diagnosis == "GAD", "3549y"])
prevalence_5064 <- as.numeric(Prevalence_estimates[Prevalence_estimates$Diagnosis == "GAD", "5064y"])

prevalence_1864 <- (census_amount_1834/sum(census_amount_1834,census_amount_3549, census_amount_5064))*prevalence_1834+
  (census_amount_3549/sum(census_amount_1834,census_amount_3549, census_amount_5064))*prevalence_3549+
  (census_amount_5064/sum(census_amount_1834,census_amount_3549, census_amount_5064))*prevalence_5064

prevalence_6574 <- as.numeric(Prevalence_estimates[Prevalence_estimates$Diagnosis == "GAD", "6574y"])
prevalence_75plus <- as.numeric(Prevalence_estimates[Prevalence_estimates$Diagnosis == "GAD", "75yplus"])

#Ratios in reference population
expected_ratio_1864 <- (census_amount_1864*prevalence_1864)/((census_amount_1864*prevalence_1864)+(census_amount_6574*prevalence_6574)+(census_amount_75plus*prevalence_75plus))
expected_ratio_6574 <- (census_amount_6574*prevalence_6574)/((census_amount_1864*prevalence_1864)+(census_amount_6574*prevalence_6574)+(census_amount_75plus*prevalence_75plus))
expected_ratio_75plus <- (census_amount_75plus*prevalence_75plus)/((census_amount_1864*prevalence_1864)+(census_amount_6574*prevalence_6574)+(census_amount_75plus*prevalence_75plus))

expected_ratios <- c(expected_ratio_1864,
                     expected_ratio_6574,
                     expected_ratio_75plus)
round(expected_ratios*100,1)
```

```
## [1] 84.5  7.6  7.9
```

```
KODAP_data_complete_GAD <- KODAP_data_complete[KODAP_data_complete$ICD1_pre_recode %in% c("F41.1 Generalisierte Angststörung") |
                                                 KODAP_data_complete$ICD2_pre_recode %in% c("F41.1 Generalisierte Angststörung") | 
                                                 KODAP_data_complete$ICD3_pre_recode %in% c("F41.1 Generalisierte Angststörung") |
                                                 KODAP_data_complete$ICD4_pre_recode %in% c("F41.1 Generalisierte Angststörung") |
                                                 KODAP_data_complete$ICD5_pre_recode %in% c("F41.1 Generalisierte Angststörung"),]

length(KODAP_data_complete_GAD$Patient_ID)
```

```
## [1] 552
```

```
#Observed vs. expected: 
Observation <- as.vector(table(KODAP_data_complete_GAD$Age_Stepped))
Expected <- expected_ratios*length(KODAP_data_complete_GAD$Patient_ID)

Observation
```

```
## [1] 526  20   6
```

```
round(Observation/length(KODAP_data_complete_GAD$Patient_ID)*100,1)
```

```
## [1] 95.3  3.6  1.1
```

```
# Chi-squared test for given probabilities
test <- chisq.test(Observation, p = expected_ratios)
test
```

```
## 
##  Chi-squared test for given probabilities
## 
## data:  Observation
## X-squared = 51.696, df = 2, p-value = 5.946e-12
```

```
# Post-hoc binomial-test for each category with Bonferroni-correction
binom.test(Observation[1], sum(Observation), expected_ratios[1])$p.value*3
```

```
## [1] 5.844488e-15
```

```
binom.test(Observation[2], sum(Observation), expected_ratios[2])$p.value*3
```

```
## [1] 0.0004446198
```

```
binom.test(Observation[3], sum(Observation), expected_ratios[3])$p.value*3
```

```
## [1] 1.319313e-12
```

```
#Estimating underrepresentation compared to reference population
#Representation quotients
Observation/Expected
```

```
## [1] 1.1279988 0.4773477 0.1370198
```

```
#Confidence-intervals of Representation quotients
#Confidence-intervals around sample proportion
prop.test(x = Observation[2], n = sum(Observation), conf.level = .95)
```

```
## 
##  1-sample proportions test with continuity correction
## 
## data:  Observation[2] out of sum(Observation), null probability 0.5
## X-squared = 473.05, df = 1, p-value < 2.2e-16
## alternative hypothesis: true p is not equal to 0.5
## 95 percent confidence interval:
##  0.02285853 0.05637906
## sample estimates:
##          p 
## 0.03623188
```

```
#Confidence-intervals around sample proportion relative to expected proportion
round(prop.test(x = Observation[2], n = sum(Observation), conf.level = .95)$estimate/expected_ratio_6574,2)
```

```
##    p 
## 0.48
```

```
round(as.vector(prop.test(x = Observation[2], n = sum(Observation), conf.level = .95)$conf.int)/expected_ratio_6574,2)
```

```
## [1] 0.30 0.74
```

```
#Confidence-intervals around sample proportion
prop.test(x = Observation[3], n = sum(Observation), conf.level = .95)
```

```
## 
##  1-sample proportions test with continuity correction
## 
## data:  Observation[3] out of sum(Observation), null probability 0.5
## X-squared = 526.31, df = 1, p-value < 2.2e-16
## alternative hypothesis: true p is not equal to 0.5
## 95 percent confidence interval:
##  0.004426108 0.024731112
## sample estimates:
##          p 
## 0.01086957
```

```
#Confidence-intervals around sample proportion relative to expected proportion
round(prop.test(x = Observation[3], n = sum(Observation), conf.level = .95)$estimate/expected_ratio_75plus,2)
```

```
##    p 
## 0.14
```

```
round(as.vector(prop.test(x = Observation[3], n = sum(Observation), conf.level = .95)$conf.int)/expected_ratio_75plus,2)
```

```
## [1] 0.06 0.31
```

```
################################################
##########Obsessive compulsive disorders########
################################################
prevalence_1834 <- as.numeric(Prevalence_estimates[Prevalence_estimates$Diagnosis == "OCD", "1834y"])
prevalence_3549 <- as.numeric(Prevalence_estimates[Prevalence_estimates$Diagnosis == "OCD", "3549y"])
prevalence_5064 <- as.numeric(Prevalence_estimates[Prevalence_estimates$Diagnosis == "OCD", "5064y"])

prevalence_1864 <- (census_amount_1834/sum(census_amount_1834,census_amount_3549, census_amount_5064))*prevalence_1834+
  (census_amount_3549/sum(census_amount_1834,census_amount_3549, census_amount_5064))*prevalence_3549+
  (census_amount_5064/sum(census_amount_1834,census_amount_3549, census_amount_5064))*prevalence_5064

prevalence_6574 <- as.numeric(Prevalence_estimates[Prevalence_estimates$Diagnosis == "OCD", "6574y"])
prevalence_75plus <- as.numeric(Prevalence_estimates[Prevalence_estimates$Diagnosis == "OCD", "75yplus"])

#Ratios in reference population
expected_ratio_1864 <- (census_amount_1864*prevalence_1864)/((census_amount_1864*prevalence_1864)+(census_amount_6574*prevalence_6574)+(census_amount_75plus*prevalence_75plus))
expected_ratio_6574 <- (census_amount_6574*prevalence_6574)/((census_amount_1864*prevalence_1864)+(census_amount_6574*prevalence_6574)+(census_amount_75plus*prevalence_75plus))
expected_ratio_75plus <- (census_amount_75plus*prevalence_75plus)/((census_amount_1864*prevalence_1864)+(census_amount_6574*prevalence_6574)+(census_amount_75plus*prevalence_75plus))

expected_ratios <- c(expected_ratio_1864,
                     expected_ratio_6574,
                     expected_ratio_75plus)
round(expected_ratios*100,1)
```

```
## [1] 91.5  4.1  4.3
```

```
KODAP_data_complete_OCD <- KODAP_data_complete[KODAP_data_complete$ICD1_pre_recode %in% c("F42.X Zwangsstörung") |
                                                 KODAP_data_complete$ICD2_pre_recode %in% c("F42.X Zwangsstörung") | 
                                                 KODAP_data_complete$ICD3_pre_recode %in% c("F42.X Zwangsstörung") |
                                                 KODAP_data_complete$ICD4_pre_recode %in% c("F42.X Zwangsstörung") |
                                                 KODAP_data_complete$ICD5_pre_recode %in% c("F42.X Zwangsstörung"),]

length(KODAP_data_complete_OCD$Patient_ID)
```

```
## [1] 791
```

```
#Observed vs. expected: 
Observation <- as.vector(table(KODAP_data_complete_OCD$Age_Stepped))
Expected <- expected_ratios*length(KODAP_data_complete_OCD$Patient_ID)

Observation
```

```
## [1] 778  11   2
```

```
round(Observation/length(KODAP_data_complete_OCD$Patient_ID)*100,1)
```

```
## [1] 98.4  1.4  0.3
```

```
# Chi-squared test for given probabilities
test <- chisq.test(Observation, p = expected_ratios)
test
```

```
## 
##  Chi-squared test for given probabilities
## 
## data:  Observation
## X-squared = 48.952, df = 2, p-value = 2.345e-11
```

```
# Post-hoc binomial-test for each category with Bonferroni-correction
binom.test(Observation[1], sum(Observation), expected_ratios[1])$p.value*3
```

```
## [1] 7.126015e-16
```

```
binom.test(Observation[2], sum(Observation), expected_ratios[2])$p.value*3
```

```
## [1] 4.558771e-05
```

```
binom.test(Observation[3], sum(Observation), expected_ratios[3])$p.value*3
```

```
## [1] 2.09123e-12
```

```
#Estimating underrepresentation compared to reference population
#Representation quotients
Observation/Expected
```

```
## [1] 1.07474006 0.33524804 0.05832179
```

```
#Confidence-intervals of Representation quotients
#Confidence-intervals around sample proportion
prop.test(x = Observation[2], n = sum(Observation), conf.level = .95)
```

```
## 
##  1-sample proportions test with continuity correction
## 
## data:  Observation[2] out of sum(Observation), null probability 0.5
## X-squared = 745.67, df = 1, p-value < 2.2e-16
## alternative hypothesis: true p is not equal to 0.5
## 95 percent confidence interval:
##  0.007329893 0.025530476
## sample estimates:
##          p 
## 0.01390645
```

```
#Confidence-intervals around sample proportion relative to expected proportion
round(prop.test(x = Observation[2], n = sum(Observation), conf.level = .95)$estimate/expected_ratio_6574,2)
```

```
##    p 
## 0.34
```

```
round(as.vector(prop.test(x = Observation[2], n = sum(Observation), conf.level = .95)$conf.int)/expected_ratio_6574,2)
```

```
## [1] 0.18 0.62
```

```
#Confidence-intervals around sample proportion
prop.test(x = Observation[3], n = sum(Observation), conf.level = .95)
```

```
## 
##  1-sample proportions test with continuity correction
## 
## data:  Observation[3] out of sum(Observation), null probability 0.5
## X-squared = 781.03, df = 1, p-value < 2.2e-16
## alternative hypothesis: true p is not equal to 0.5
## 95 percent confidence interval:
##  0.0004380695 0.0101435327
## sample estimates:
##           p 
## 0.002528445
```

```
#Confidence-intervals around sample proportion relative to expected proportion
round(prop.test(x = Observation[3], n = sum(Observation), conf.level = .95)$estimate/expected_ratio_75plus,2)
```

```
##    p 
## 0.06
```

```
round(as.vector(prop.test(x = Observation[3], n = sum(Observation), conf.level = .95)$conf.int)/expected_ratio_75plus,2)
```

```
## [1] 0.01 0.23
```

```
################################################
##########Post traumatic stress disorder########
################################################
prevalence_1834 <- as.numeric(Prevalence_estimates[Prevalence_estimates$Diagnosis == "PTSD", "1834y"])
prevalence_3549 <- as.numeric(Prevalence_estimates[Prevalence_estimates$Diagnosis == "PTSD", "3549y"])
prevalence_5064 <- as.numeric(Prevalence_estimates[Prevalence_estimates$Diagnosis == "PTSD", "5064y"])

prevalence_1864 <- (census_amount_1834/sum(census_amount_1834,census_amount_3549, census_amount_5064))*prevalence_1834+
  (census_amount_3549/sum(census_amount_1834,census_amount_3549, census_amount_5064))*prevalence_3549+
  (census_amount_5064/sum(census_amount_1834,census_amount_3549, census_amount_5064))*prevalence_5064

prevalence_6574 <- as.numeric(Prevalence_estimates[Prevalence_estimates$Diagnosis == "PTSD", "6574y"])
prevalence_75plus <- as.numeric(Prevalence_estimates[Prevalence_estimates$Diagnosis == "PTSD", "75yplus"])

#Ratios in reference population
expected_ratio_1864 <- (census_amount_1864*prevalence_1864)/((census_amount_1864*prevalence_1864)+(census_amount_6574*prevalence_6574)+(census_amount_75plus*prevalence_75plus))
expected_ratio_6574 <- (census_amount_6574*prevalence_6574)/((census_amount_1864*prevalence_1864)+(census_amount_6574*prevalence_6574)+(census_amount_75plus*prevalence_75plus))
expected_ratio_75plus <- (census_amount_75plus*prevalence_75plus)/((census_amount_1864*prevalence_1864)+(census_amount_6574*prevalence_6574)+(census_amount_75plus*prevalence_75plus))

expected_ratios <- c(expected_ratio_1864,
                     expected_ratio_6574,
                     expected_ratio_75plus)
round(expected_ratios*100,1)
```

```
## [1] 78.3 10.6 11.1
```

```
KODAP_data_complete_PTSD <- KODAP_data_complete[KODAP_data_complete$ICD1_pre_recode %in% c("F43.1 Posttraumatische Belastungsstörung") |
                                                  KODAP_data_complete$ICD2_pre_recode %in% c("F43.1 Posttraumatische Belastungsstörung") | 
                                                  KODAP_data_complete$ICD3_pre_recode %in% c("F43.1 Posttraumatische Belastungsstörung") |
                                                  KODAP_data_complete$ICD4_pre_recode %in% c("F43.1 Posttraumatische Belastungsstörung") | 
                                                  KODAP_data_complete$ICD5_pre_recode %in% c("F43.1 Posttraumatische Belastungsstörung"),]

length(KODAP_data_complete_PTSD$Patient_ID)
```

```
## [1] 1067
```

```
#Observed vs. expected: 
Observation <- as.vector(table(KODAP_data_complete_PTSD$Age_Stepped))
Expected <- expected_ratios*length(KODAP_data_complete_PTSD$Patient_ID)

Observation
```

```
## [1] 1052   13    2
```

```
round(Observation/length(KODAP_data_complete_PTSD$Patient_ID)*100,1)
```

```
## [1] 98.6  1.2  0.2
```

```
# Chi-squared test for given probabilities
test <- chisq.test(Observation, p = expected_ratios)
test
```

```
## 
##  Chi-squared test for given probabilities
## 
## data:  Observation
## X-squared = 258.38, df = 2, p-value < 2.2e-16
```

```
# Post-hoc binomial-test for each category with Bonferroni-correction
binom.test(Observation[1], sum(Observation), expected_ratios[1])$p.value*3
```

```
## [1] 3.664279e-88
```

```
binom.test(Observation[2], sum(Observation), expected_ratios[2])$p.value*3
```

```
## [1] 3.194624e-34
```

```
binom.test(Observation[3], sum(Observation), expected_ratios[3])$p.value*3
```

```
## [1] 2.148891e-50
```

```
#Estimating underrepresentation compared to reference population
#Representation quotients
Observation/Expected
```

```
## [1] 1.25840984 0.11508209 0.01694032
```

```
#Confidence-intervals of Representation quotients
#Confidence-intervals around sample proportion
prop.test(x = Observation[2], n = sum(Observation), conf.level = .95)
```

```
## 
##  1-sample proportions test with continuity correction
## 
## data:  Observation[2] out of sum(Observation), null probability 0.5
## X-squared = 1013.7, df = 1, p-value < 2.2e-16
## alternative hypothesis: true p is not equal to 0.5
## 95 percent confidence interval:
##  0.006788272 0.021319397
## sample estimates:
##          p 
## 0.01218369
```

```
#Confidence-intervals around sample proportion relative to expected proportion
round(prop.test(x = Observation[2], n = sum(Observation), conf.level = .95)$estimate/expected_ratio_6574,2)
```

```
##    p 
## 0.12
```

```
round(as.vector(prop.test(x = Observation[2], n = sum(Observation), conf.level = .95)$conf.int)/expected_ratio_6574,2)
```

```
## [1] 0.06 0.20
```

```
#Confidence-intervals around sample proportion
prop.test(x = Observation[3], n = sum(Observation), conf.level = .95)
```

```
## 
##  1-sample proportions test with continuity correction
## 
## data:  Observation[3] out of sum(Observation), null probability 0.5
## X-squared = 1057, df = 1, p-value < 2.2e-16
## alternative hypothesis: true p is not equal to 0.5
## 95 percent confidence interval:
##  0.0003247314 0.0075301326
## sample estimates:
##           p 
## 0.001874414
```

```
#Confidence-intervals around sample proportion relative to expected proportion
round(prop.test(x = Observation[3], n = sum(Observation), conf.level = .95)$estimate/expected_ratio_75plus,2)
```

```
##    p 
## 0.02
```

```
round(as.vector(prop.test(x = Observation[3], n = sum(Observation), conf.level = .95)$conf.int)/expected_ratio_75plus,2)
```

```
## [1] 0.00 0.07
```

```
################################################
##########Any somatoform Disorders##################
################################################
prevalence_1834 <- as.numeric(Prevalence_estimates[Prevalence_estimates$Diagnosis == "Any somatoform disorder", "1834y"])
prevalence_3549 <- as.numeric(Prevalence_estimates[Prevalence_estimates$Diagnosis == "Any somatoform disorder", "3549y"])
prevalence_5064 <- as.numeric(Prevalence_estimates[Prevalence_estimates$Diagnosis == "Any somatoform disorder", "5064y"])

prevalence_1864 <- (census_amount_1834/sum(census_amount_1834,census_amount_3549, census_amount_5064))*prevalence_1834+
  (census_amount_3549/sum(census_amount_1834,census_amount_3549, census_amount_5064))*prevalence_3549+
  (census_amount_5064/sum(census_amount_1834,census_amount_3549, census_amount_5064))*prevalence_5064

prevalence_6574 <- as.numeric(Prevalence_estimates[Prevalence_estimates$Diagnosis == "Any somatoform disorder", "6574y"])
prevalence_75plus <- as.numeric(Prevalence_estimates[Prevalence_estimates$Diagnosis == "Any somatoform disorder", "75yplus"])

#Ratios in reference population
expected_ratio_1864 <- (census_amount_1864*prevalence_1864)/((census_amount_1864*prevalence_1864)+(census_amount_6574*prevalence_6574)+(census_amount_75plus*prevalence_75plus))
expected_ratio_6574 <- (census_amount_6574*prevalence_6574)/((census_amount_1864*prevalence_1864)+(census_amount_6574*prevalence_6574)+(census_amount_75plus*prevalence_75plus))
expected_ratio_75plus <- (census_amount_75plus*prevalence_75plus)/((census_amount_1864*prevalence_1864)+(census_amount_6574*prevalence_6574)+(census_amount_75plus*prevalence_75plus))

expected_ratios <- c(expected_ratio_1864,
                     expected_ratio_6574,
                     expected_ratio_75plus)
round(expected_ratios*100,1)
```

```
## [1] 83.7  8.0  8.3
```

```
KODAP_data_complete_Somatoform <- KODAP_data_complete[KODAP_data_complete$ICD1_pre_recode %in% c("F45.X Somatoforme Störungen") |
                                                        KODAP_data_complete$ICD2_pre_recode %in% c("F45.X Somatoforme Störungen") | 
                                                        KODAP_data_complete$ICD3_pre_recode %in% c("F45.X Somatoforme Störungen") |
                                                        KODAP_data_complete$ICD4_pre_recode %in% c("F45.X Somatoforme Störungen") |
                                                        KODAP_data_complete$ICD5_pre_recode %in% c("F45.X Somatoforme Störungen"),]

length(KODAP_data_complete_Somatoform$Patient_ID)
```

```
## [1] 1074
```

```
#Observed vs. expected: 
Observation <- as.vector(table(KODAP_data_complete_Somatoform$Age_Stepped))
Expected <- expected_ratios*length(KODAP_data_complete_Somatoform$Patient_ID)

Observation
```

```
## [1] 1009   48   17
```

```
round(Observation/length(KODAP_data_complete_Somatoform$Patient_ID)*100,1)
```

```
## [1] 93.9  4.5  1.6
```

```
# Chi-squared test for given probabilities
test <- chisq.test(Observation, p = expected_ratios)
test
```

```
## 
##  Chi-squared test for given probabilities
## 
## data:  Observation
## X-squared = 88.963, df = 2, p-value < 2.2e-16
```

```
# Post-hoc binomial-test for each category with Bonferroni-correction
binom.test(Observation[1], sum(Observation), expected_ratios[1])$p.value*3
```

```
## [1] 8.645902e-24
```

```
binom.test(Observation[2], sum(Observation), expected_ratios[2])$p.value*3
```

```
## [1] 1.828896e-05
```

```
binom.test(Observation[3], sum(Observation), expected_ratios[3])$p.value*3
```

```
## [1] 3.153289e-21
```

```
#Estimating underrepresentation compared to reference population
#Representation quotients
Observation/Expected
```

```
## [1] 1.1227562 0.5599319 0.1897449
```

```
#Confidence-intervals of Representation quotients
#Confidence-intervals around sample proportion
prop.test(x = Observation[2], n = sum(Observation), conf.level = .95)
```

```
## 
##  1-sample proportions test with continuity correction
## 
## data:  Observation[2] out of sum(Observation), null probability 0.5
## X-squared = 888.76, df = 1, p-value < 2.2e-16
## alternative hypothesis: true p is not equal to 0.5
## 95 percent confidence interval:
##  0.03347037 0.05928157
## sample estimates:
##          p 
## 0.04469274
```

```
#Confidence-intervals around sample proportion relative to expected proportion
round(prop.test(x = Observation[2], n = sum(Observation), conf.level = .95)$estimate/expected_ratio_6574,2)
```

```
##    p 
## 0.56
```

```
round(as.vector(prop.test(x = Observation[2], n = sum(Observation), conf.level = .95)$conf.int)/expected_ratio_6574,2)
```

```
## [1] 0.42 0.74
```

```
#Confidence-intervals around sample proportion
prop.test(x = Observation[3], n = sum(Observation), conf.level = .95)
```

```
## 
##  1-sample proportions test with continuity correction
## 
## data:  Observation[3] out of sum(Observation), null probability 0.5
## X-squared = 1005.1, df = 1, p-value < 2.2e-16
## alternative hypothesis: true p is not equal to 0.5
## 95 percent confidence interval:
##  0.009547387 0.025770512
## sample estimates:
##          p 
## 0.01582868
```

```
#Confidence-intervals around sample proportion relative to expected proportion
round(prop.test(x = Observation[3], n = sum(Observation), conf.level = .95)$estimate/expected_ratio_75plus,2)
```

```
##    p 
## 0.19
```

```
round(as.vector(prop.test(x = Observation[3], n = sum(Observation), conf.level = .95)$conf.int)/expected_ratio_75plus,2)
```

```
## [1] 0.11 0.31
```

```
################################################
##########Somatization disorder##################
################################################
prevalence_1834 <- as.numeric(Prevalence_estimates[Prevalence_estimates$Diagnosis == "Somatization disorder", "1834y"])
prevalence_3549 <- as.numeric(Prevalence_estimates[Prevalence_estimates$Diagnosis == "Somatization disorder", "3549y"])
prevalence_5064 <- as.numeric(Prevalence_estimates[Prevalence_estimates$Diagnosis == "Somatization disorder", "5064y"])

prevalence_1864 <- (census_amount_1834/sum(census_amount_1834,census_amount_3549, census_amount_5064))*prevalence_1834+
  (census_amount_3549/sum(census_amount_1834,census_amount_3549, census_amount_5064))*prevalence_3549+
  (census_amount_5064/sum(census_amount_1834,census_amount_3549, census_amount_5064))*prevalence_5064

prevalence_6574 <- as.numeric(Prevalence_estimates[Prevalence_estimates$Diagnosis == "Somatization disorder", "6574y"])
prevalence_75plus <- as.numeric(Prevalence_estimates[Prevalence_estimates$Diagnosis == "Somatization disorder", "75yplus"])

#Ratios in reference population
expected_ratio_1864 <- (census_amount_1864*prevalence_1864)/((census_amount_1864*prevalence_1864)+(census_amount_6574*prevalence_6574)+(census_amount_75plus*prevalence_75plus))
expected_ratio_6574 <- (census_amount_6574*prevalence_6574)/((census_amount_1864*prevalence_1864)+(census_amount_6574*prevalence_6574)+(census_amount_75plus*prevalence_75plus))
expected_ratio_75plus <- (census_amount_75plus*prevalence_75plus)/((census_amount_1864*prevalence_1864)+(census_amount_6574*prevalence_6574)+(census_amount_75plus*prevalence_75plus))

expected_ratios <- c(expected_ratio_1864,
                     expected_ratio_6574,
                     expected_ratio_75plus)
round(expected_ratios*100,1)
```

```
## [1] 73.8 12.8 13.4
```

```
KODAP_data_complete_Somatization <- KODAP_data_complete[KODAP_data_complete$ICD1_pre_clean %in% c("F45.0","F45.00", "F45.1", "F45.10") |
                                                        KODAP_data_complete$ICD2_pre_clean %in% c("F45.0","F45.00", "F45.1", "F45.10") | 
                                                        KODAP_data_complete$ICD3_pre_clean %in% c("F45.0","F45.00", "F45.1", "F45.10") |
                                                        KODAP_data_complete$ICD4_pre_clean %in% c("F45.0","F45.00", "F45.1", "F45.10") |
                                                        KODAP_data_complete$ICD5_pre_clean %in% c("F45.0","F45.00", "F45.1", "F45.10"),]

length(KODAP_data_complete_Somatization$Patient_ID)
```

```
## [1] 211
```

```
#Observed vs. expected: 
Observation <- as.vector(table(KODAP_data_complete_Somatization$Age_Stepped))
Expected <- expected_ratios*length(KODAP_data_complete_Somatization$Patient_ID)

Observation
```

```
## [1] 203   7   1
```

```
round(Observation/length(KODAP_data_complete_Somatization$Patient_ID)*100,1)
```

```
## [1] 96.2  3.3  0.5
```

```
# Chi-squared test for given probabilities
test <- chisq.test(Observation, p = expected_ratios)
test
```

```
## 
##  Chi-squared test for given probabilities
## 
## data:  Observation
## X-squared = 55.336, df = 2, p-value = 9.636e-13
```

```
# Post-hoc binomial-test for each category with Bonferroni-correction
binom.test(Observation[1], sum(Observation), expected_ratios[1])$p.value*3
```

```
## [1] 1.943582e-17
```

```
binom.test(Observation[2], sum(Observation), expected_ratios[2])$p.value*3
```

```
## [1] 1.177964e-05
```

```
binom.test(Observation[3], sum(Observation), expected_ratios[3])$p.value*3
```

```
## [1] 1.182352e-11
```

```
#Estimating underrepresentation compared to reference population
#Representation quotients
Observation/Expected
```

```
## [1] 1.30288177 0.25938667 0.03545495
```

```
#Confidence-intervals of Representation quotients
#Confidence-intervals around sample proportion
prop.test(x = Observation[2], n = sum(Observation), conf.level = .95)
```

```
## 
##  1-sample proportions test with continuity correction
## 
## data:  Observation[2] out of sum(Observation), null probability 0.5
## X-squared = 182.07, df = 1, p-value < 2.2e-16
## alternative hypothesis: true p is not equal to 0.5
## 95 percent confidence interval:
##  0.01461394 0.06996360
## sample estimates:
##          p 
## 0.03317536
```

```
#Confidence-intervals around sample proportion relative to expected proportion
round(prop.test(x = Observation[2], n = sum(Observation), conf.level = .95)$estimate/expected_ratio_6574,2)
```

```
##    p 
## 0.26
```

```
round(as.vector(prop.test(x = Observation[2], n = sum(Observation), conf.level = .95)$conf.int)/expected_ratio_6574,2)
```

```
## [1] 0.11 0.55
```

```
#Confidence-intervals around sample proportion
prop.test(x = Observation[3], n = sum(Observation), conf.level = .95)
```

```
## 
##  1-sample proportions test with continuity correction
## 
## data:  Observation[3] out of sum(Observation), null probability 0.5
## X-squared = 205.04, df = 1, p-value < 2.2e-16
## alternative hypothesis: true p is not equal to 0.5
## 95 percent confidence interval:
##  0.0002474437 0.0302007408
## sample estimates:
##           p 
## 0.004739336
```

```
#Confidence-intervals around sample proportion relative to expected proportion
round(prop.test(x = Observation[3], n = sum(Observation), conf.level = .95)$estimate/expected_ratio_75plus,2)
```

```
##    p 
## 0.04
```

```
round(as.vector(prop.test(x = Observation[3], n = sum(Observation), conf.level = .95)$conf.int)/expected_ratio_75plus,2)
```

```
## [1] 0.00 0.23
```

```
################################################
##########Pain disorder##################
################################################
prevalence_1834 <- as.numeric(Prevalence_estimates[Prevalence_estimates$Diagnosis == "Pain disorder", "1834y"])
prevalence_3549 <- as.numeric(Prevalence_estimates[Prevalence_estimates$Diagnosis == "Pain disorder", "3549y"])
prevalence_5064 <- as.numeric(Prevalence_estimates[Prevalence_estimates$Diagnosis == "Pain disorder", "5064y"])

prevalence_1864 <- (census_amount_1834/sum(census_amount_1834,census_amount_3549, census_amount_5064))*prevalence_1834+
  (census_amount_3549/sum(census_amount_1834,census_amount_3549, census_amount_5064))*prevalence_3549+
  (census_amount_5064/sum(census_amount_1834,census_amount_3549, census_amount_5064))*prevalence_5064

prevalence_6574 <- as.numeric(Prevalence_estimates[Prevalence_estimates$Diagnosis == "Pain disorder", "6574y"])
prevalence_75plus <- as.numeric(Prevalence_estimates[Prevalence_estimates$Diagnosis == "Pain disorder", "75yplus"])

#Ratios in reference population
expected_ratio_1864 <- (census_amount_1864*prevalence_1864)/((census_amount_1864*prevalence_1864)+(census_amount_6574*prevalence_6574)+(census_amount_75plus*prevalence_75plus))
expected_ratio_6574 <- (census_amount_6574*prevalence_6574)/((census_amount_1864*prevalence_1864)+(census_amount_6574*prevalence_6574)+(census_amount_75plus*prevalence_75plus))
expected_ratio_75plus <- (census_amount_75plus*prevalence_75plus)/((census_amount_1864*prevalence_1864)+(census_amount_6574*prevalence_6574)+(census_amount_75plus*prevalence_75plus))

expected_ratios <- c(expected_ratio_1864,
                     expected_ratio_6574,
                     expected_ratio_75plus)
round(expected_ratios*100,1)
```

```
## [1] 86.2  6.8  7.1
```

```
KODAP_data_complete_Pain <- KODAP_data_complete[KODAP_data_complete$ICD1_pre_clean %in% c("F45.4","F45.40", "F45.41") |
                                                          KODAP_data_complete$ICD2_pre_clean %in% c("F45.4","F45.40", "F45.41") | 
                                                          KODAP_data_complete$ICD3_pre_clean %in% c("F45.4","F45.40", "F45.41") |
                                                          KODAP_data_complete$ICD4_pre_clean %in% c("F45.4","F45.40", "F45.41") |
                                                          KODAP_data_complete$ICD5_pre_clean %in% c("F45.4","F45.40", "F45.41"),]

length(KODAP_data_complete_Pain$Patient_ID)
```

```
## [1] 508
```

```
#Observed vs. expected: 
Observation <- as.vector(table(KODAP_data_complete_Pain$Age_Stepped))
Expected <- expected_ratios*length(KODAP_data_complete_Pain$Patient_ID)

Observation
```

```
## [1] 465  30  13
```

```
round(Observation/length(KODAP_data_complete_Pain$Patient_ID)*100,1)
```

```
## [1] 91.5  5.9  2.6
```

```
# Chi-squared test for given probabilities
test <- chisq.test(Observation, p = expected_ratios)
test
```

```
## 
##  Chi-squared test for given probabilities
## 
## data:  Observation
## X-squared = 16.893, df = 2, p-value = 0.0002146
```

```
# Post-hoc binomial-test for each category with Bonferroni-correction
binom.test(Observation[1], sum(Observation), expected_ratios[1])$p.value*3
```

```
## [1] 0.0007199377
```

```
binom.test(Observation[2], sum(Observation), expected_ratios[2])$p.value*3
```

```
## [1] 1.441711
```

```
binom.test(Observation[3], sum(Observation), expected_ratios[3])$p.value*3
```

```
## [1] 3.690537e-05
```

```
#Estimating underrepresentation compared to reference population
#Representation quotients
Observation/Expected
```

```
## [1] 1.0623855 0.8726746 0.3618277
```

```
#Confidence-intervals of Representation quotients
#Confidence-intervals around sample proportion
prop.test(x = Observation[2], n = sum(Observation), conf.level = .95)
```

```
## 
##  1-sample proportions test with continuity correction
## 
## data:  Observation[2] out of sum(Observation), null probability 0.5
## X-squared = 393.32, df = 1, p-value < 2.2e-16
## alternative hypothesis: true p is not equal to 0.5
## 95 percent confidence interval:
##  0.04085658 0.08418493
## sample estimates:
##          p 
## 0.05905512
```

```
#Confidence-intervals around sample proportion relative to expected proportion
round(prop.test(x = Observation[2], n = sum(Observation), conf.level = .95)$estimate/expected_ratio_6574,2)
```

```
##    p 
## 0.87
```

```
round(as.vector(prop.test(x = Observation[2], n = sum(Observation), conf.level = .95)$conf.int)/expected_ratio_6574,2)
```

```
## [1] 0.60 1.24
```

```
#Confidence-intervals around sample proportion
prop.test(x = Observation[3], n = sum(Observation), conf.level = .95)
```

```
## 
##  1-sample proportions test with continuity correction
## 
## data:  Observation[3] out of sum(Observation), null probability 0.5
## X-squared = 455.44, df = 1, p-value < 2.2e-16
## alternative hypothesis: true p is not equal to 0.5
## 95 percent confidence interval:
##  0.01428679 0.04450754
## sample estimates:
##          p 
## 0.02559055
```

```
#Confidence-intervals around sample proportion relative to expected proportion
round(prop.test(x = Observation[3], n = sum(Observation), conf.level = .95)$estimate/expected_ratio_75plus,2)
```

```
##    p 
## 0.36
```

```
round(as.vector(prop.test(x = Observation[3], n = sum(Observation), conf.level = .95)$conf.int)/expected_ratio_75plus,2)
```

```
## [1] 0.20 0.63
```

```
################################################
##########Eating disorders##################
################################################
prevalence_1834 <- as.numeric(Prevalence_estimates[Prevalence_estimates$Diagnosis == "Eating disorders", "1834y"])
prevalence_3549 <- as.numeric(Prevalence_estimates[Prevalence_estimates$Diagnosis == "Eating disorders", "3549y"])
prevalence_5064 <- as.numeric(Prevalence_estimates[Prevalence_estimates$Diagnosis == "Eating disorders", "5064y"])

prevalence_1864 <- (census_amount_1834/sum(census_amount_1834,census_amount_3549, census_amount_5064))*prevalence_1834+
  (census_amount_3549/sum(census_amount_1834,census_amount_3549, census_amount_5064))*prevalence_3549+
  (census_amount_5064/sum(census_amount_1834,census_amount_3549, census_amount_5064))*prevalence_5064

prevalence_6574 <- as.numeric(Prevalence_estimates[Prevalence_estimates$Diagnosis == "Eating disorders", "6574y"])
prevalence_75plus <- as.numeric(Prevalence_estimates[Prevalence_estimates$Diagnosis == "Eating disorders", "75yplus"])

#Ratios in reference population
expected_ratio_1864 <- (census_amount_1864*prevalence_1864)/((census_amount_1864*prevalence_1864)+(census_amount_6574*prevalence_6574)+(census_amount_75plus*prevalence_75plus))
expected_ratio_6574 <- (census_amount_6574*prevalence_6574)/((census_amount_1864*prevalence_1864)+(census_amount_6574*prevalence_6574)+(census_amount_75plus*prevalence_75plus))
expected_ratio_75plus <- (census_amount_75plus*prevalence_75plus)/((census_amount_1864*prevalence_1864)+(census_amount_6574*prevalence_6574)+(census_amount_75plus*prevalence_75plus))

expected_ratios <- c(expected_ratio_1864,
                     expected_ratio_6574,
                     expected_ratio_75plus)
round(expected_ratios*100,1)
```

```
## [1] 89.0  5.4  5.6
```

```
KODAP_data_complete_Eating <- KODAP_data_complete[KODAP_data_complete$ICD1_pre_recode %in% c("F50.X Essstörung") |
                                                    KODAP_data_complete$ICD2_pre_recode %in% c("F50.X Essstörung") | 
                                                    KODAP_data_complete$ICD3_pre_recode %in% c("F50.X Essstörung") |
                                                    KODAP_data_complete$ICD4_pre_recode %in% c("F50.X Essstörung") |
                                                    KODAP_data_complete$ICD5_pre_recode %in% c("F50.X Essstörung"),]

length(KODAP_data_complete_Eating$Patient_ID)
```

```
## [1] 857
```

```
#Observed vs. expected: 
Observation <- as.vector(table(KODAP_data_complete_Eating$Age_Stepped))
Expected <- expected_ratios*length(KODAP_data_complete_Eating$Patient_ID)

Observation
```

```
## [1] 849   8
```

```
Observation[3] <- 0
Observation
```

```
## [1] 849   8   0
```

```
round(Observation/length(KODAP_data_complete_Eating$Patient_ID)*100,1)
```

```
## [1] 99.1  0.9  0.0
```

```
# Chi-squared test for given probabilities
test <- chisq.test(Observation, p = expected_ratios)
test
```

```
## 
##  Chi-squared test for given probabilities
## 
## data:  Observation
## X-squared = 89.395, df = 2, p-value < 2.2e-16
```

```
# Post-hoc binomial-test for each category with Bonferroni-correction
binom.test(Observation[1], sum(Observation), expected_ratios[1])$p.value*3
```

```
## [1] 8.208025e-32
```

```
binom.test(Observation[2], sum(Observation), expected_ratios[2])$p.value*3
```

```
## [1] 1.213906e-11
```

```
binom.test(Observation[3], sum(Observation), expected_ratios[3])$p.value*3
```

```
## [1] 1.441333e-21
```

```
#Estimating underrepresentation compared to reference population
#Representation quotients
Observation/Expected
```

```
## [1] 1.1130814 0.1735878 0.0000000
```

```
#Confidence-intervals of Representation quotients
#Confidence-intervals around sample proportion
prop.test(x = Observation[2], n = sum(Observation), conf.level = .95)
```

```
## 
##  1-sample proportions test with continuity correction
## 
## data:  Observation[2] out of sum(Observation), null probability 0.5
## X-squared = 823.34, df = 1, p-value < 2.2e-16
## alternative hypothesis: true p is not equal to 0.5
## 95 percent confidence interval:
##  0.004346906 0.019076925
## sample estimates:
##           p 
## 0.009334889
```

```
#Confidence-intervals around sample proportion relative to expected proportion
round(prop.test(x = Observation[2], n = sum(Observation), conf.level = .95)$estimate/expected_ratio_6574,2)
```

```
##    p 
## 0.17
```

```
round(as.vector(prop.test(x = Observation[2], n = sum(Observation), conf.level = .95)$conf.int)/expected_ratio_6574,2)
```

```
## [1] 0.08 0.35
```

```
#Confidence-intervals around sample proportion
prop.test(x = Observation[3], n = sum(Observation), conf.level = .95)
```

```
## 
##  1-sample proportions test with continuity correction
## 
## data:  Observation[3] out of sum(Observation), null probability 0.5
## X-squared = 855, df = 1, p-value < 2.2e-16
## alternative hypothesis: true p is not equal to 0.5
## 95 percent confidence interval:
##  0.000000000 0.005563187
## sample estimates:
## p 
## 0
```

```
#Confidence-intervals around sample proportion relative to expected proportion
round(prop.test(x = Observation[3], n = sum(Observation), conf.level = .95)$estimate/expected_ratio_75plus,2)
```

```
## p 
## 0
```

```
round(as.vector(prop.test(x = Observation[3], n = sum(Observation), conf.level = .95)$conf.int)/expected_ratio_75plus,2)
```

```
## [1] 0.0 0.1
```

```
#########################################
##########Substance-use disorders########
#########################################
prevalence_1834 <- as.numeric(Prevalence_estimates[Prevalence_estimates$Diagnosis == "Substance use disorders", "1834y"])
prevalence_3549 <- as.numeric(Prevalence_estimates[Prevalence_estimates$Diagnosis == "Substance use disorders", "3549y"])
prevalence_5064 <- as.numeric(Prevalence_estimates[Prevalence_estimates$Diagnosis == "Substance use disorders", "5064y"])

prevalence_1864 <- (census_amount_1834/sum(census_amount_1834,census_amount_3549, census_amount_5064))*prevalence_1834+
  (census_amount_3549/sum(census_amount_1834,census_amount_3549, census_amount_5064))*prevalence_3549+
  (census_amount_5064/sum(census_amount_1834,census_amount_3549, census_amount_5064))*prevalence_5064

prevalence_6574 <- as.numeric(Prevalence_estimates[Prevalence_estimates$Diagnosis == "Substance use disorders", "6574y"])
prevalence_75plus <- as.numeric(Prevalence_estimates[Prevalence_estimates$Diagnosis == "Substance use disorders", "75yplus"])

#Ratios in reference population
expected_ratio_1864 <- (census_amount_1864*prevalence_1864)/((census_amount_1864*prevalence_1864)+(census_amount_6574*prevalence_6574)+(census_amount_75plus*prevalence_75plus))
expected_ratio_6574 <- (census_amount_6574*prevalence_6574)/((census_amount_1864*prevalence_1864)+(census_amount_6574*prevalence_6574)+(census_amount_75plus*prevalence_75plus))
expected_ratio_75plus <- (census_amount_75plus*prevalence_75plus)/((census_amount_1864*prevalence_1864)+(census_amount_6574*prevalence_6574)+(census_amount_75plus*prevalence_75plus))

expected_ratios <- c(expected_ratio_1864,
                     expected_ratio_6574,
                     expected_ratio_75plus)
round(expected_ratios*100,1)
```

```
## [1] 88.0  5.9  6.1
```

```
KODAP_data_complete_SubstanceUse <- KODAP_data_complete[KODAP_data_complete$ICD1_pre_recode %in% c("F1X.X Psychische und Verhaltensstörungen durch psychotrope Substanzen") |
                                                          KODAP_data_complete$ICD2_pre_recode %in% c("F1X.X Psychische und Verhaltensstörungen durch psychotrope Substanzen") | 
                                                          KODAP_data_complete$ICD3_pre_recode %in% c("F1X.X Psychische und Verhaltensstörungen durch psychotrope Substanzen") |
                                                          KODAP_data_complete$ICD4_pre_recode %in% c("F1X.X Psychische und Verhaltensstörungen durch psychotrope Substanzen") |
                                                          KODAP_data_complete$ICD5_pre_recode %in% c("F1X.X Psychische und Verhaltensstörungen durch psychotrope Substanzen"),]

length(KODAP_data_complete_SubstanceUse$Patient_ID)
```

```
## [1] 764
```

```
#Observed vs. expected: 
Observation <- as.vector(table(KODAP_data_complete_SubstanceUse$Age_Stepped))
Expected <- expected_ratios*length(KODAP_data_complete_SubstanceUse$Patient_ID)

Observation
```

```
## [1] 748  15   1
```

```
round(Observation/length(KODAP_data_complete_SubstanceUse$Patient_ID)*100,1)
```

```
## [1] 97.9  2.0  0.1
```

```
# Chi-squared test for given probabilities
test <- chisq.test(Observation, p = expected_ratios)
test
```

```
## 
##  Chi-squared test for given probabilities
## 
## data:  Observation
## X-squared = 73.277, df = 2, p-value < 2.2e-16
```

```
# Post-hoc binomial-test for each category with Bonferroni-correction
binom.test(Observation[1], sum(Observation), expected_ratios[1])$p.value*3
```

```
## [1] 1.93638e-23
```

```
binom.test(Observation[2], sum(Observation), expected_ratios[2])$p.value*3
```

```
## [1] 8.330415e-07
```

```
binom.test(Observation[3], sum(Observation), expected_ratios[3])$p.value*3
```

```
## [1] 3.308868e-19
```

```
#Estimating underrepresentation compared to reference population
#Representation quotients
Observation/Expected
```

```
## [1] 1.1126184 0.3344931 0.0213365
```

```
#Confidence-intervals of Representation quotients
#Confidence-intervals around sample proportion
prop.test(x = Observation[2], n = sum(Observation), conf.level = .95)
```

```
## 
##  1-sample proportions test with continuity correction
## 
## data:  Observation[2] out of sum(Observation), null probability 0.5
## X-squared = 703.26, df = 1, p-value < 2.2e-16
## alternative hypothesis: true p is not equal to 0.5
## 95 percent confidence interval:
##  0.01143871 0.03294463
## sample estimates:
##          p 
## 0.01963351
```

```
#Confidence-intervals around sample proportion relative to expected proportion
round(prop.test(x = Observation[2], n = sum(Observation), conf.level = .95)$estimate/expected_ratio_6574,2)
```

```
##    p 
## 0.33
```

```
round(as.vector(prop.test(x = Observation[2], n = sum(Observation), conf.level = .95)$conf.int)/expected_ratio_6574,2)
```

```
## [1] 0.19 0.56
```

```
#Confidence-intervals around sample proportion
prop.test(x = Observation[3], n = sum(Observation), conf.level = .95)
```

```
## 
##  1-sample proportions test with continuity correction
## 
## data:  Observation[3] out of sum(Observation), null probability 0.5
## X-squared = 758.01, df = 1, p-value < 2.2e-16
## alternative hypothesis: true p is not equal to 0.5
## 95 percent confidence interval:
##  6.832858e-05 8.456433e-03
## sample estimates:
##           p 
## 0.001308901
```

```
#Confidence-intervals around sample proportion relative to expected proportion
round(prop.test(x = Observation[3], n = sum(Observation), conf.level = .95)$estimate/expected_ratio_75plus,2)
```

```
##    p 
## 0.02
```

```
round(as.vector(prop.test(x = Observation[3], n = sum(Observation), conf.level = .95)$conf.int)/expected_ratio_75plus,2)
```

```
## [1] 0.00 0.14
```

```
#########################################
##########Psychotic disorders############
#########################################
prevalence_1834 <- as.numeric(Prevalence_estimates[Prevalence_estimates$Diagnosis == "Psychotic disorders", "1834y"])
prevalence_3549 <- as.numeric(Prevalence_estimates[Prevalence_estimates$Diagnosis == "Psychotic disorders", "3549y"])
prevalence_5064 <- as.numeric(Prevalence_estimates[Prevalence_estimates$Diagnosis == "Psychotic disorders", "5064y"])

prevalence_1864 <- (census_amount_1834/sum(census_amount_1834,census_amount_3549, census_amount_5064))*prevalence_1834+
  (census_amount_3549/sum(census_amount_1834,census_amount_3549, census_amount_5064))*prevalence_3549+
  (census_amount_5064/sum(census_amount_1834,census_amount_3549, census_amount_5064))*prevalence_5064

prevalence_6574 <- as.numeric(Prevalence_estimates[Prevalence_estimates$Diagnosis == "Psychotic disorders", "6574y"])
prevalence_75plus <- as.numeric(Prevalence_estimates[Prevalence_estimates$Diagnosis == "Psychotic disorders", "75yplus"])

#Ratios in reference population
expected_ratio_1864 <- (census_amount_1864*prevalence_1864)/((census_amount_1864*prevalence_1864)+(census_amount_6574*prevalence_6574)+(census_amount_75plus*prevalence_75plus))
expected_ratio_6574 <- (census_amount_6574*prevalence_6574)/((census_amount_1864*prevalence_1864)+(census_amount_6574*prevalence_6574)+(census_amount_75plus*prevalence_75plus))
expected_ratio_75plus <- (census_amount_75plus*prevalence_75plus)/((census_amount_1864*prevalence_1864)+(census_amount_6574*prevalence_6574)+(census_amount_75plus*prevalence_75plus))

expected_ratios <- c(expected_ratio_1864,
                     expected_ratio_6574,
                     expected_ratio_75plus)
round(expected_ratios*100,1)
```

```
## [1] 86.4  6.6  6.9
```

```
KODAP_data_complete_Psychotic <- KODAP_data_complete[KODAP_data_complete$ICD1_pre_recode %in% c("F20.X Schizophrenie",
                                                                                                "F21 F22 F23 F24 F28 F29 Psychotische oder wahnhafte Störungen (außer Schizophrenie)",
                                                                                                "F25.X Schizoaffektive Störungen") |
                                                       KODAP_data_complete$ICD2_pre_recode %in% c("F20.X Schizophrenie",
                                                                                                  "F21 F22 F23 F24 F28 F29 Psychotische oder wahnhafte Störungen (außer Schizophrenie)",
                                                                                                  "F25.X Schizoaffektive Störungen") | 
                                                       KODAP_data_complete$ICD3_pre_recode %in% c("F20.X Schizophrenie",
                                                                                                  "F21 F22 F23 F24 F28 F29 Psychotische oder wahnhafte Störungen (außer Schizophrenie)",
                                                                                                  "F25.X Schizoaffektive Störungen") |
                                                       KODAP_data_complete$ICD4_pre_recode %in% c("F20.X Schizophrenie",
                                                                                                  "F21 F22 F23 F24 F28 F29 Psychotische oder wahnhafte Störungen (außer Schizophrenie)",
                                                                                                  "F25.X Schizoaffektive Störungen") |
                                                       KODAP_data_complete$ICD5_pre_recode %in% c("F20.X Schizophrenie",
                                                                                                  "F21 F22 F23 F24 F28 F29 Psychotische oder wahnhafte Störungen (außer Schizophrenie)",
                                                                                                  "F25.X Schizoaffektive Störungen"),]

length(KODAP_data_complete_Psychotic$Patient_ID)
```

```
## [1] 336
```

```
#Observed vs. expected: 
Observation <- as.vector(table(KODAP_data_complete_Psychotic$Age_Stepped))
Expected <- expected_ratios*length(KODAP_data_complete_Psychotic$Patient_ID)

Observation
```

```
## [1] 331   4   1
```

```
round(Observation/length(KODAP_data_complete_Psychotic$Patient_ID)*100,1)
```

```
## [1] 98.5  1.2  0.3
```

```
# Chi-squared test for given probabilities
test <- chisq.test(Observation, p = expected_ratios)
test
```

```
## 
##  Chi-squared test for given probabilities
## 
## data:  Observation
## X-squared = 42.102, df = 2, p-value = 7.207e-10
```

```
# Post-hoc binomial-test for each category with Bonferroni-correction
binom.test(Observation[1], sum(Observation), expected_ratios[1])$p.value*3
```

```
## [1] 9.935806e-15
```

```
binom.test(Observation[2], sum(Observation), expected_ratios[2])$p.value*3
```

```
## [1] 8.411055e-06
```

```
binom.test(Observation[3], sum(Observation), expected_ratios[3])$p.value*3
```

```
## [1] 6.119362e-09
```

```
#Estimating underrepresentation compared to reference population
#Representation quotients
Observation/Expected
```

```
## [1] 1.14000572 0.17919911 0.04286503
```

```
#Confidence-intervals of Representation quotients
#Confidence-intervals around sample proportion
prop.test(x = Observation[2], n = sum(Observation), conf.level = .95)
```

```
## 
##  1-sample proportions test with continuity correction
## 
## data:  Observation[2] out of sum(Observation), null probability 0.5
## X-squared = 318.24, df = 1, p-value < 2.2e-16
## alternative hypothesis: true p is not equal to 0.5
## 95 percent confidence interval:
##  0.003820372 0.032295397
## sample estimates:
##          p 
## 0.01190476
```

```
#Confidence-intervals around sample proportion relative to expected proportion
round(prop.test(x = Observation[2], n = sum(Observation), conf.level = .95)$estimate/expected_ratio_6574,2)
```

```
##    p 
## 0.18
```

```
round(as.vector(prop.test(x = Observation[2], n = sum(Observation), conf.level = .95)$conf.int)/expected_ratio_6574,2)
```

```
## [1] 0.06 0.49
```

```
#Confidence-intervals around sample proportion
prop.test(x = Observation[3], n = sum(Observation), conf.level = .95)
```

```
## 
##  1-sample proportions test with continuity correction
## 
## data:  Observation[3] out of sum(Observation), null probability 0.5
## X-squared = 330.03, df = 1, p-value < 2.2e-16
## alternative hypothesis: true p is not equal to 0.5
## 95 percent confidence interval:
##  0.0001553772 0.0190996535
## sample estimates:
##          p 
## 0.00297619
```

```
#Confidence-intervals around sample proportion relative to expected proportion
round(prop.test(x = Observation[3], n = sum(Observation), conf.level = .95)$estimate/expected_ratio_75plus,2)
```

```
##    p 
## 0.04
```

```
round(as.vector(prop.test(x = Observation[3], n = sum(Observation), conf.level = .95)$conf.int)/expected_ratio_75plus,2)
```

```
## [1] 0.00 0.28
```

## Subgroups of working-age adults

```
#Read prevalence estimates
Prevalence_estimates <- read_excel("Prevalence_estimates.xlsx")
Prevalence_estimates
```

```
## # A tibble: 17 × 6
##    Diagnosis                  `1834y` `3549y` `5064y` `6574y` `75yplus`
##    <chr>                        <dbl>   <dbl>   <dbl>   <dbl>     <dbl>
##  1 Any mental disorder           35.8    28      26.4    19.6      19.6
##  2 Any mood disorder             15.1    10.3     7       5.9       5.9
##  3 Major Depressive Disorder     10       7.2     5.2     4.4       4.4
##  4 Dysthymia                      2.1     1.7     1.3     1.6       1.6
##  5 Any anxiety disorder          18.1    16.2    15.3    11.1      11.1
##  6 Panic disorder/Agoraphobia     4.2     4.1     4.1     3.5       3.5
##  7 Social phobia                  4.6     3.1     2.2     0.7       0.7
##  8 Specific phobias              12.3     9.5    10.9     8.4       8.4
##  9 GAD                            3.3     2       2.3     1.3       1.3
## 10 OCD                            7.2     3.6     2.2     1.1       1.1
## 11 PTSD                           3.7     2.5     1       1.8       1.8
## 12 Any somatoform disorder        4.2     3.8     3.6     2.1       2.1
## 13 Somatization disorder          0.9     0.6     0.9     0.8       0.8
## 14 Pain disorder                  4       3.8     3       1.6       1.6
## 15 Eating disorders               2.3     0.5     0.7     0.4       0.4
## 16 Substance use disorders        8.4     5.9     5.5     2.5       2.5
## 17 Psychotic disorders            4.2     2.2     2.5     1.3       1.3
```

```
##################
#####Analyses#####
##################

#Built Subset Datasets
KODAP_data_complete_s1 <- subset(KODAP_data_complete, Age_Stepped_2 %in% c(0,3,4))
KODAP_data_complete_s2 <- subset(KODAP_data_complete, Age_Stepped_2 %in% c(1,3,4))
KODAP_data_complete_s3 <- subset(KODAP_data_complete, Age_Stepped_2 %in% c(2,3,4))

#####################
#Any Mental Disorder#
#####################
prevalence_1834 <- as.numeric(Prevalence_estimates[Prevalence_estimates$Diagnosis == "Any mental disorder", "1834y"])
prevalence_3549 <- as.numeric(Prevalence_estimates[Prevalence_estimates$Diagnosis == "Any mental disorder", "3549y"])
prevalence_5064 <- as.numeric(Prevalence_estimates[Prevalence_estimates$Diagnosis == "Any mental disorder", "5064y"])
prevalence_6574 <- as.numeric(Prevalence_estimates[Prevalence_estimates$Diagnosis == "Any mental disorder", "6574y"])
prevalence_75plus <- as.numeric(Prevalence_estimates[Prevalence_estimates$Diagnosis == "Any mental disorder", "75yplus"])

expected_ratio_1834 <- (census_amount_1834*prevalence_1834)/((census_amount_1834*prevalence_1834)+(census_amount_3549*prevalence_3549)+(census_amount_5064*prevalence_5064)+(census_amount_6574*prevalence_6574)+(census_amount_75plus*prevalence_75plus))
expected_ratio_3549 <- (census_amount_3549*prevalence_3549)/((census_amount_1834*prevalence_1834)+(census_amount_3549*prevalence_3549)+(census_amount_5064*prevalence_5064)+(census_amount_6574*prevalence_6574)+(census_amount_75plus*prevalence_75plus))
expected_ratio_5064 <- (census_amount_5064*prevalence_5064)/((census_amount_1834*prevalence_1834)+(census_amount_3549*prevalence_3549)+(census_amount_5064*prevalence_5064)+(census_amount_6574*prevalence_6574)+(census_amount_75plus*prevalence_75plus))
expected_ratio_6574 <- (census_amount_6574*prevalence_6574)/((census_amount_1834*prevalence_1834)+(census_amount_3549*prevalence_3549)+(census_amount_5064*prevalence_5064)+(census_amount_6574*prevalence_6574)+(census_amount_75plus*prevalence_75plus))
expected_ratio_75plus <- (census_amount_75plus*prevalence_75plus)/((census_amount_1834*prevalence_1834)+(census_amount_3549*prevalence_3549)+(census_amount_5064*prevalence_5064)+(census_amount_6574*prevalence_6574)+(census_amount_75plus*prevalence_75plus))

round(c(expected_ratio_1834,expected_ratio_3549,expected_ratio_5064,expected_ratio_6574,expected_ratio_75plus), 2)
```

```
## [1] 0.31 0.23 0.27 0.09 0.10
```

```
table(KODAP_data_complete$Age_Stepped_2)
```

```
## 
##    0    1    2    3    4 
## 7621 3144 2453  324   93
```

```
round(table(KODAP_data_complete$Age_Stepped_2)/length(KODAP_data_complete$Age_Stepped_2)*100, 1)
```

```
## 
##    0    1    2    3    4 
## 55.9 23.1 18.0  2.4  0.7
```

```
#18-34 years
prevalence_1834 <- as.numeric(Prevalence_estimates[Prevalence_estimates$Diagnosis == "Any mental disorder", "1834y"])
prevalence_3549 <- as.numeric(Prevalence_estimates[Prevalence_estimates$Diagnosis == "Any mental disorder", "3549y"])
prevalence_5064 <- as.numeric(Prevalence_estimates[Prevalence_estimates$Diagnosis == "Any mental disorder", "5064y"])

prevalence_1864 <- prevalence_1834

prevalence_6574 <- as.numeric(Prevalence_estimates[Prevalence_estimates$Diagnosis == "Any mental disorder", "6574y"])
prevalence_75plus <- as.numeric(Prevalence_estimates[Prevalence_estimates$Diagnosis == "Any mental disorder", "75yplus"])

#Ratios in reference population
expected_ratio_1864 <- (census_amount_1834*prevalence_1864)/((census_amount_1834*prevalence_1864)+(census_amount_6574*prevalence_6574)+(census_amount_75plus*prevalence_75plus))
expected_ratio_6574 <- (census_amount_6574*prevalence_6574)/((census_amount_1834*prevalence_1864)+(census_amount_6574*prevalence_6574)+(census_amount_75plus*prevalence_75plus))
expected_ratio_75plus <- (census_amount_75plus*prevalence_75plus)/((census_amount_1834*prevalence_1864)+(census_amount_6574*prevalence_6574)+(census_amount_75plus*prevalence_75plus))

expected_ratios <- c(expected_ratio_1864,
                     expected_ratio_6574,
                     expected_ratio_75plus)
round(expected_ratios,2)
```

```
## [1] 0.62 0.18 0.19
```

```
#Observed vs. expected: 
Observation <- as.vector(table(KODAP_data_complete_s1$Age_Stepped))

Observation
```

```
## [1] 7621  324   93
```

```
round(Observation/length(KODAP_data_complete_s1$Patient_ID)*100, 1)
```

```
## [1] 94.8  4.0  1.2
```

```
# Chi-squared test for given probabilities
test <- chisq.test(Observation, p = expected_ratios)
test
```

```
## 
##  Chi-squared test for given probabilities
## 
## data:  Observation
## X-squared = 3622.7, df = 2, p-value < 2.2e-16
```

```
# Post-hoc binomial-test for each category with Bonferroni-correction
binom.test(Observation[1], sum(Observation), expected_ratios[1])$p.value*3
```

```
## [1] 1.482197e-323
```

```
binom.test(Observation[2], sum(Observation), expected_ratios[2])$p.value*3
```

```
## [1] 0
```

```
binom.test(Observation[3], sum(Observation), expected_ratios[3])$p.value*3
```

```
## [1] 1.482197e-323
```

```
#Estimating underrepresentation compared to reference population
#Representation quotients
Observation/Expected
```

```
## [1] 26.247684 14.515128  3.986448
```

```
#Confidence-intervals of Representation quotients

#Confidence-intervals around sample proportion
prop.test(x = Observation[2], n = sum(Observation), conf.level = .95)
```

```
## 
##  1-sample proportions test with continuity correction
## 
## data:  Observation[2] out of sum(Observation), null probability 0.5
## X-squared = 6792.4, df = 1, p-value < 2.2e-16
## alternative hypothesis: true p is not equal to 0.5
## 95 percent confidence interval:
##  0.03616484 0.04489775
## sample estimates:
##          p 
## 0.04030853
```

```
#Confidence-intervals around sample proportion relative to expected proportion
round(prop.test(x = Observation[2], n = sum(Observation), conf.level = .95)$estimate/expected_ratio_6574,2)
```

```
##    p 
## 0.22
```

```
round(as.vector(prop.test(x = Observation[2], n = sum(Observation), conf.level = .95)$conf.int)/expected_ratio_6574, 2)
```

```
## [1] 0.20 0.24
```

```
#Confidence-intervals around sample proportion
prop.test(x = Observation[3], n = sum(Observation), conf.level = .95)
```

```
## 
##  1-sample proportions test with continuity correction
## 
## data:  Observation[3] out of sum(Observation), null probability 0.5
## X-squared = 7668.4, df = 1, p-value < 2.2e-16
## alternative hypothesis: true p is not equal to 0.5
## 95 percent confidence interval:
##  0.009398471 0.014220596
## sample estimates:
##          p 
## 0.01157004
```

```
#Confidence-intervals around sample proportion relative to expected proportion
round(prop.test(x = Observation[3], n = sum(Observation), conf.level = .95)$estimate/expected_ratio_75plus,2)
```

```
##    p 
## 0.06
```

```
round(as.vector(prop.test(x = Observation[3], n = sum(Observation), conf.level = .95)$conf.int)/expected_ratio_75plus,2)
```

```
## [1] 0.05 0.07
```

```
#35-49 years
prevalence_1834 <- as.numeric(Prevalence_estimates[Prevalence_estimates$Diagnosis == "Any mental disorder", "1834y"])
prevalence_3549 <- as.numeric(Prevalence_estimates[Prevalence_estimates$Diagnosis == "Any mental disorder", "3549y"])
prevalence_5064 <- as.numeric(Prevalence_estimates[Prevalence_estimates$Diagnosis == "Any mental disorder", "5064y"])

prevalence_1864 <- prevalence_3549

prevalence_6574 <- as.numeric(Prevalence_estimates[Prevalence_estimates$Diagnosis == "Any mental disorder", "6574y"])
prevalence_75plus <- as.numeric(Prevalence_estimates[Prevalence_estimates$Diagnosis == "Any mental disorder", "75yplus"])

#Ratios in reference population
expected_ratio_1864 <- (census_amount_3549*prevalence_1864)/((census_amount_3549*prevalence_1864)+(census_amount_6574*prevalence_6574)+(census_amount_75plus*prevalence_75plus))
expected_ratio_6574 <- (census_amount_6574*prevalence_6574)/((census_amount_3549*prevalence_1864)+(census_amount_6574*prevalence_6574)+(census_amount_75plus*prevalence_75plus))
expected_ratio_75plus <- (census_amount_75plus*prevalence_75plus)/((census_amount_3549*prevalence_1864)+(census_amount_6574*prevalence_6574)+(census_amount_75plus*prevalence_75plus))

expected_ratios <- c(expected_ratio_1864,
                     expected_ratio_6574,
                     expected_ratio_75plus)
round(expected_ratios,2)
```

```
## [1] 0.55 0.22 0.23
```

```
#Observed vs. expected: 
Observation <- as.vector(table(KODAP_data_complete_s2$Age_Stepped))

Observation
```

```
## [1] 3144  324   93
```

```
round(Observation/length(KODAP_data_complete_s2$Patient_ID)*100, 1)
```

```
## [1] 88.3  9.1  2.6
```

```
# Chi-squared test for given probabilities
test <- chisq.test(Observation, p = expected_ratios)
test
```

```
## 
##  Chi-squared test for given probabilities
## 
## data:  Observation
## X-squared = 1651.8, df = 2, p-value < 2.2e-16
```

```
# Post-hoc binomial-test for each category with Bonferroni-correction
binom.test(Observation[1], sum(Observation), expected_ratios[1])$p.value*3
```

```
## [1] 0
```

```
binom.test(Observation[2], sum(Observation), expected_ratios[2])$p.value*3
```

```
## [1] 8.079779e-94
```

```
binom.test(Observation[3], sum(Observation), expected_ratios[3])$p.value*3
```

```
## [1] 2.351106e-269
```

```
#Estimating underrepresentation compared to reference population
#Representation quotients
Observation/Expected
```

```
## [1] 10.828332 14.515128  3.986448
```

```
#Confidence-intervals of Representation quotients

#Confidence-intervals around sample proportion
prop.test(x = Observation[2], n = sum(Observation), conf.level = .95)
```

```
## 
##  1-sample proportions test with continuity correction
## 
## data:  Observation[2] out of sum(Observation), null probability 0.5
## X-squared = 2381.3, df = 1, p-value < 2.2e-16
## alternative hypothesis: true p is not equal to 0.5
## 95 percent confidence interval:
##  0.08184183 0.10102412
## sample estimates:
##          p 
## 0.09098568
```

```
#Confidence-intervals around sample proportion relative to expected proportion
round(prop.test(x = Observation[2], n = sum(Observation), conf.level = .95)$estimate/expected_ratio_6574,2)
```

```
##    p 
## 0.41
```

```
round(as.vector(prop.test(x = Observation[2], n = sum(Observation), conf.level = .95)$conf.int)/expected_ratio_6574, 2)
```

```
## [1] 0.37 0.46
```

```
#Confidence-intervals around sample proportion
prop.test(x = Observation[3], n = sum(Observation), conf.level = .95)
```

```
## 
##  1-sample proportions test with continuity correction
## 
## data:  Observation[3] out of sum(Observation), null probability 0.5
## X-squared = 3196.8, df = 1, p-value < 2.2e-16
## alternative hypothesis: true p is not equal to 0.5
## 95 percent confidence interval:
##  0.02124020 0.03204086
## sample estimates:
##          p 
## 0.02611626
```

```
#Confidence-intervals around sample proportion relative to expected proportion
round(prop.test(x = Observation[3], n = sum(Observation), conf.level = .95)$estimate/expected_ratio_75plus,2)
```

```
##    p 
## 0.11
```

```
round(as.vector(prop.test(x = Observation[3], n = sum(Observation), conf.level = .95)$conf.int)/expected_ratio_75plus,2)
```

```
## [1] 0.09 0.14
```

```
#50-64 years
prevalence_1834 <- as.numeric(Prevalence_estimates[Prevalence_estimates$Diagnosis == "Any mental disorder", "1834y"])
prevalence_3549 <- as.numeric(Prevalence_estimates[Prevalence_estimates$Diagnosis == "Any mental disorder", "3549y"])
prevalence_5064 <- as.numeric(Prevalence_estimates[Prevalence_estimates$Diagnosis == "Any mental disorder", "5064y"])

prevalence_1864 <- prevalence_5064

prevalence_6574 <- as.numeric(Prevalence_estimates[Prevalence_estimates$Diagnosis == "Any mental disorder", "6574y"])
prevalence_75plus <- as.numeric(Prevalence_estimates[Prevalence_estimates$Diagnosis == "Any mental disorder", "75yplus"])

#Ratios in reference population
expected_ratio_1864 <- (census_amount_5064*prevalence_1864)/((census_amount_5064*prevalence_1864)+(census_amount_6574*prevalence_6574)+(census_amount_75plus*prevalence_75plus))
expected_ratio_6574 <- (census_amount_6574*prevalence_6574)/((census_amount_5064*prevalence_1864)+(census_amount_6574*prevalence_6574)+(census_amount_75plus*prevalence_75plus))
expected_ratio_75plus <- (census_amount_75plus*prevalence_75plus)/((census_amount_5064*prevalence_1864)+(census_amount_6574*prevalence_6574)+(census_amount_75plus*prevalence_75plus))

expected_ratios <- c(expected_ratio_1864,
                     expected_ratio_6574,
                     expected_ratio_75plus)
round(expected_ratios,2)
```

```
## [1] 0.58 0.20 0.21
```

```
#Observed vs. expected: 
Observation <- as.vector(table(KODAP_data_complete_s3$Age_Stepped))

Observation
```

```
## [1] 2453  324   93
```

```
round(Observation/length(KODAP_data_complete_s3$Patient_ID)*100, 1)
```

```
## [1] 85.5 11.3  3.2
```

```
# Chi-squared test for given probabilities
test <- chisq.test(Observation, p = expected_ratios)
test
```

```
## 
##  Chi-squared test for given probabilities
## 
## data:  Observation
## X-squared = 921.22, df = 2, p-value < 2.2e-16
```

```
# Post-hoc binomial-test for each category with Bonferroni-correction
binom.test(Observation[1], sum(Observation), expected_ratios[1])$p.value*3
```

```
## [1] 1.25446e-218
```

```
binom.test(Observation[2], sum(Observation), expected_ratios[2])$p.value*3
```

```
## [1] 7.903984e-38
```

```
binom.test(Observation[3], sum(Observation), expected_ratios[3])$p.value*3
```

```
## [1] 9.47345e-175
```

```
#Estimating underrepresentation compared to reference population
#Representation quotients
Observation/Expected
```

```
## [1]  8.448441 14.515128  3.986448
```

```
#Confidence-intervals of Representation quotients

#Confidence-intervals around sample proportion
prop.test(x = Observation[2], n = sum(Observation), conf.level = .95)
```

```
## 
##  1-sample proportions test with continuity correction
## 
## data:  Observation[2] out of sum(Observation), null probability 0.5
## X-squared = 1718.8, df = 1, p-value < 2.2e-16
## alternative hypothesis: true p is not equal to 0.5
## 95 percent confidence interval:
##  0.1016616 0.1251728
## sample estimates:
##        p 
## 0.112892
```

```
#Confidence-intervals around sample proportion relative to expected proportion
round(prop.test(x = Observation[2], n = sum(Observation), conf.level = .95)$estimate/expected_ratio_6574,2)
```

```
##    p 
## 0.55
```

```
round(as.vector(prop.test(x = Observation[2], n = sum(Observation), conf.level = .95)$conf.int)/expected_ratio_6574, 2)
```

```
## [1] 0.50 0.61
```

```
#Confidence-intervals around sample proportion
prop.test(x = Observation[3], n = sum(Observation), conf.level = .95)
```

```
## 
##  1-sample proportions test with continuity correction
## 
## data:  Observation[3] out of sum(Observation), null probability 0.5
## X-squared = 2508.2, df = 1, p-value < 2.2e-16
## alternative hypothesis: true p is not equal to 0.5
## 95 percent confidence interval:
##  0.02636800 0.03972387
## sample estimates:
##          p 
## 0.03240418
```

```
#Confidence-intervals around sample proportion relative to expected proportion
round(prop.test(x = Observation[3], n = sum(Observation), conf.level = .95)$estimate/expected_ratio_75plus,2)
```

```
##    p 
## 0.15
```

```
round(as.vector(prop.test(x = Observation[3], n = sum(Observation), conf.level = .95)$conf.int)/expected_ratio_75plus,2)
```

```
## [1] 0.12 0.19
```

```
#####################
#Any Mood Disorder#
#####################
prevalence_1834 <- as.numeric(Prevalence_estimates[Prevalence_estimates$Diagnosis == "Any mood disorder", "1834y"])
prevalence_3549 <- as.numeric(Prevalence_estimates[Prevalence_estimates$Diagnosis == "Any mood disorder", "3549y"])
prevalence_5064 <- as.numeric(Prevalence_estimates[Prevalence_estimates$Diagnosis == "Any mood disorder", "5064y"])
prevalence_6574 <- as.numeric(Prevalence_estimates[Prevalence_estimates$Diagnosis == "Any mood disorder", "6574y"])
prevalence_75plus <- as.numeric(Prevalence_estimates[Prevalence_estimates$Diagnosis == "Any mood disorder", "75yplus"])

expected_ratio_1834 <- (census_amount_1834*prevalence_1834)/((census_amount_1834*prevalence_1834)+(census_amount_3549*prevalence_3549)+(census_amount_5064*prevalence_5064)+(census_amount_6574*prevalence_6574)+(census_amount_75plus*prevalence_75plus))
expected_ratio_3549 <- (census_amount_3549*prevalence_3549)/((census_amount_1834*prevalence_1834)+(census_amount_3549*prevalence_3549)+(census_amount_5064*prevalence_5064)+(census_amount_6574*prevalence_6574)+(census_amount_75plus*prevalence_75plus))
expected_ratio_5064 <- (census_amount_5064*prevalence_5064)/((census_amount_1834*prevalence_1834)+(census_amount_3549*prevalence_3549)+(census_amount_5064*prevalence_5064)+(census_amount_6574*prevalence_6574)+(census_amount_75plus*prevalence_75plus))
expected_ratio_6574 <- (census_amount_6574*prevalence_6574)/((census_amount_1834*prevalence_1834)+(census_amount_3549*prevalence_3549)+(census_amount_5064*prevalence_5064)+(census_amount_6574*prevalence_6574)+(census_amount_75plus*prevalence_75plus))
expected_ratio_75plus <- (census_amount_75plus*prevalence_75plus)/((census_amount_1834*prevalence_1834)+(census_amount_3549*prevalence_3549)+(census_amount_5064*prevalence_5064)+(census_amount_6574*prevalence_6574)+(census_amount_75plus*prevalence_75plus))

round(c(expected_ratio_1834,expected_ratio_3549,expected_ratio_5064,expected_ratio_6574,expected_ratio_75plus), 2)
```

```
## [1] 0.39 0.25 0.20 0.08 0.08
```

```
KODAP_data_complete_any_mood_disorder <- KODAP_data_complete[KODAP_data_complete$ICD1_pre_recode %in% c("F30.X F31.X Manische Episode oder Bipolare Störungen",
                                                                                                              "F31.7 Bipolare affktive Störung, gegenwärtig remittiert",
                                                                                                              "F32 Depressive Episode",
                                                                                                              "F33 Rezidivierende depressive Störung",
                                                                                                              "F33.4 Rezidivierende depressive Störung, gegenwärtig remittiert",
                                                                                                              "F34 Anhaltende affektive Störungen",
                                                                                                              "F38.X Andere affektive Störung") |
                                                               KODAP_data_complete$ICD2_pre_recode %in% c("F30.X F31.X Manische Episode oder Bipolare Störungen",
                                                                                                                "F31.7 Bipolare affktive Störung, gegenwärtig remittiert",
                                                                                                                "F32 Depressive Episode",
                                                                                                                "F33 Rezidivierende depressive Störung",
                                                                                                                "F33.4 Rezidivierende depressive Störung, gegenwärtig remittiert",
                                                                                                                "F34 Anhaltende affektive Störungen",
                                                                                                                "F38.X Andere affektive Störung") |
                                                               KODAP_data_complete$ICD3_pre_recode %in% c("F30.X F31.X Manische Episode oder Bipolare Störungen",
                                                                                                                "F31.7 Bipolare affktive Störung, gegenwärtig remittiert",
                                                                                                                "F32 Depressive Episode",
                                                                                                                "F33 Rezidivierende depressive Störung",
                                                                                                                "F33.4 Rezidivierende depressive Störung, gegenwärtig remittiert",
                                                                                                                "F34 Anhaltende affektive Störungen",
                                                                                                                "F38.X Andere affektive Störung") |
                                                               KODAP_data_complete$ICD4_pre_recode %in% c("F30.X F31.X Manische Episode oder Bipolare Störungen",
                                                                                                                "F31.7 Bipolare affktive Störung, gegenwärtig remittiert",
                                                                                                                "F32 Depressive Episode",
                                                                                                                "F33 Rezidivierende depressive Störung",
                                                                                                                "F33.4 Rezidivierende depressive Störung, gegenwärtig remittiert",
                                                                                                                "F34 Anhaltende affektive Störungen",
                                                                                                                "F38.X Andere affektive Störung") |
                                                               KODAP_data_complete$ICD5_pre_recode %in% c("F30.X F31.X Manische Episode oder Bipolare Störungen",
                                                                                                                "F31.7 Bipolare affktive Störung, gegenwärtig remittiert",
                                                                                                                "F32 Depressive Episode",
                                                                                                                "F33 Rezidivierende depressive Störung",
                                                                                                                "F33.4 Rezidivierende depressive Störung, gegenwärtig remittiert",
                                                                                                                "F34 Anhaltende affektive Störungen",
                                                                                                                "F38.X Andere affektive Störung"),]

table(KODAP_data_complete_any_mood_disorder$Age_Stepped_2)
```

```
## 
##    0    1    2    3    4 
## 4464 1877 1686  179   38
```

```
round(table(KODAP_data_complete_any_mood_disorder$Age_Stepped_2)/length(KODAP_data_complete_any_mood_disorder$Age_Stepped_2)*100, 1)
```

```
## 
##    0    1    2    3    4 
## 54.1 22.8 20.5  2.2  0.5
```

```
#18-34 years
prevalence_1834 <- as.numeric(Prevalence_estimates[Prevalence_estimates$Diagnosis == "Any mood disorder", "1834y"])
prevalence_3549 <- as.numeric(Prevalence_estimates[Prevalence_estimates$Diagnosis == "Any mood disorder", "3549y"])
prevalence_5064 <- as.numeric(Prevalence_estimates[Prevalence_estimates$Diagnosis == "Any mood disorder", "5064y"])

prevalence_1864 <- prevalence_1834

prevalence_6574 <- as.numeric(Prevalence_estimates[Prevalence_estimates$Diagnosis == "Any mood disorder", "6574y"])
prevalence_75plus <- as.numeric(Prevalence_estimates[Prevalence_estimates$Diagnosis == "Any mood disorder", "75yplus"])

#Ratios in reference population
expected_ratio_1864 <- (census_amount_1834*prevalence_1864)/((census_amount_1834*prevalence_1864)+(census_amount_6574*prevalence_6574)+(census_amount_75plus*prevalence_75plus))
expected_ratio_6574 <- (census_amount_6574*prevalence_6574)/((census_amount_1834*prevalence_1864)+(census_amount_6574*prevalence_6574)+(census_amount_75plus*prevalence_75plus))
expected_ratio_75plus <- (census_amount_75plus*prevalence_75plus)/((census_amount_1834*prevalence_1864)+(census_amount_6574*prevalence_6574)+(census_amount_75plus*prevalence_75plus))

expected_ratios <- c(expected_ratio_1864,
                     expected_ratio_6574,
                     expected_ratio_75plus)
round(expected_ratios,2)
```

```
## [1] 0.70 0.15 0.15
```

```
KODAP_data_complete_any_mood_disorder <- KODAP_data_complete_s1[KODAP_data_complete_s1$ICD1_pre_recode %in% c("F30.X F31.X Manische Episode oder Bipolare Störungen",
                                                                                                        "F31.7 Bipolare affktive Störung, gegenwärtig remittiert",
                                                                                                        "F32 Depressive Episode",
                                                                                                        "F33 Rezidivierende depressive Störung",
                                                                                                        "F33.4 Rezidivierende depressive Störung, gegenwärtig remittiert",
                                                                                                        "F34 Anhaltende affektive Störungen",
                                                                                                        "F38.X Andere affektive Störung") |
                                                                  KODAP_data_complete_s1$ICD2_pre_recode %in% c("F30.X F31.X Manische Episode oder Bipolare Störungen",
                                                                                                          "F31.7 Bipolare affktive Störung, gegenwärtig remittiert",
                                                                                                          "F32 Depressive Episode",
                                                                                                          "F33 Rezidivierende depressive Störung",
                                                                                                          "F33.4 Rezidivierende depressive Störung, gegenwärtig remittiert",
                                                                                                          "F34 Anhaltende affektive Störungen",
                                                                                                          "F38.X Andere affektive Störung") |
                                                                  KODAP_data_complete_s1$ICD3_pre_recode %in% c("F30.X F31.X Manische Episode oder Bipolare Störungen",
                                                                                                          "F31.7 Bipolare affktive Störung, gegenwärtig remittiert",
                                                                                                          "F32 Depressive Episode",
                                                                                                          "F33 Rezidivierende depressive Störung",
                                                                                                          "F33.4 Rezidivierende depressive Störung, gegenwärtig remittiert",
                                                                                                          "F34 Anhaltende affektive Störungen",
                                                                                                          "F38.X Andere affektive Störung") |
                                                                  KODAP_data_complete_s1$ICD4_pre_recode %in% c("F30.X F31.X Manische Episode oder Bipolare Störungen",
                                                                                                          "F31.7 Bipolare affktive Störung, gegenwärtig remittiert",
                                                                                                          "F32 Depressive Episode",
                                                                                                          "F33 Rezidivierende depressive Störung",
                                                                                                          "F33.4 Rezidivierende depressive Störung, gegenwärtig remittiert",
                                                                                                          "F34 Anhaltende affektive Störungen",
                                                                                                          "F38.X Andere affektive Störung") |
                                                                  KODAP_data_complete_s1$ICD5_pre_recode %in% c("F30.X F31.X Manische Episode oder Bipolare Störungen",
                                                                                                          "F31.7 Bipolare affktive Störung, gegenwärtig remittiert",
                                                                                                          "F32 Depressive Episode",
                                                                                                          "F33 Rezidivierende depressive Störung",
                                                                                                          "F33.4 Rezidivierende depressive Störung, gegenwärtig remittiert",
                                                                                                          "F34 Anhaltende affektive Störungen",
                                                                                                          "F38.X Andere affektive Störung"),]

length(KODAP_data_complete_any_mood_disorder$Patient_ID)
```

```
## [1] 4681
```

```
#Observed vs. expected: 
Observation <- as.vector(table(KODAP_data_complete_any_mood_disorder$Age_Stepped))
Expected <- expected_ratios*length(KODAP_data_complete_any_mood_disorder$Patient_ID)

Observation
```

```
## [1] 4464  179   38
```

```
round(Observation/length(KODAP_data_complete_any_mood_disorder$Patient_ID)*100,1)
```

```
## [1] 95.4  3.8  0.8
```

```
# Chi-squared test for given probabilities
test <- chisq.test(Observation, p = expected_ratios)
test
```

```
## 
##  Chi-squared test for given probabilities
## 
## data:  Observation
## X-squared = 1457.2, df = 2, p-value < 2.2e-16
```

```
# Post-hoc binomial-test for each category with Bonferroni-correction
binom.test(Observation[1], sum(Observation), expected_ratios[1])$p.value*3
```

```
## [1] 0
```

```
binom.test(Observation[2], sum(Observation), expected_ratios[2])$p.value*3
```

```
## [1] 1.400025e-131
```

```
binom.test(Observation[3], sum(Observation), expected_ratios[3])$p.value*3
```

```
## [1] 5.946114e-273
```

```
#Estimating underrepresentation compared to reference population
#Representation quotients
Observation/Expected
```

```
## [1] 1.36417267 0.25987270 0.05278597
```

```
#Confidence-intervals of Representation quotients
#Confidence-intervals around sample proportion
prop.test(x = Observation[2], n = sum(Observation), conf.level = .95)
```

```
## 
##  1-sample proportions test with continuity correction
## 
## data:  Observation[2] out of sum(Observation), null probability 0.5
## X-squared = 3990.5, df = 1, p-value < 2.2e-16
## alternative hypothesis: true p is not equal to 0.5
## 95 percent confidence interval:
##  0.03301440 0.04423693
## sample estimates:
##          p 
## 0.03823969
```

```
#Confidence-intervals around sample proportion relative to expected proportion
round(prop.test(x = Observation[2], n = sum(Observation), conf.level = .95)$estimate/expected_ratio_6574,2)
```

```
##    p 
## 0.26
```

```
round(as.vector(prop.test(x = Observation[2], n = sum(Observation), conf.level = .95)$conf.int)/expected_ratio_6574,2)
```

```
## [1] 0.22 0.30
```

```
#Confidence-intervals around sample proportion
prop.test(x = Observation[3], n = sum(Observation), conf.level = .95)
```

```
## 
##  1-sample proportions test with continuity correction
## 
## data:  Observation[3] out of sum(Observation), null probability 0.5
## X-squared = 4528.3, df = 1, p-value < 2.2e-16
## alternative hypothesis: true p is not equal to 0.5
## 95 percent confidence interval:
##  0.005830144 0.011245467
## sample estimates:
##           p 
## 0.008117924
```

```
#Confidence-intervals around sample proportion relative to expected proportion
round(prop.test(x = Observation[3], n = sum(Observation), conf.level = .95)$estimate/expected_ratio_75plus,2)
```

```
##    p 
## 0.05
```

```
round(as.vector(prop.test(x = Observation[3], n = sum(Observation), conf.level = .95)$conf.int)/expected_ratio_75plus,2)
```

```
## [1] 0.04 0.07
```

```
#35-49 years
prevalence_1834 <- as.numeric(Prevalence_estimates[Prevalence_estimates$Diagnosis == "Any mood disorder", "1834y"])
prevalence_3549 <- as.numeric(Prevalence_estimates[Prevalence_estimates$Diagnosis == "Any mood disorder", "3549y"])
prevalence_5064 <- as.numeric(Prevalence_estimates[Prevalence_estimates$Diagnosis == "Any mood disorder", "5064y"])

prevalence_1864 <- prevalence_3549

prevalence_6574 <- as.numeric(Prevalence_estimates[Prevalence_estimates$Diagnosis == "Any mood disorder", "6574y"])
prevalence_75plus <- as.numeric(Prevalence_estimates[Prevalence_estimates$Diagnosis == "Any mood disorder", "75yplus"])

#Ratios in reference population
expected_ratio_1864 <- (census_amount_3549*prevalence_1864)/((census_amount_3549*prevalence_1864)+(census_amount_6574*prevalence_6574)+(census_amount_75plus*prevalence_75plus))
expected_ratio_6574 <- (census_amount_6574*prevalence_6574)/((census_amount_3549*prevalence_1864)+(census_amount_6574*prevalence_6574)+(census_amount_75plus*prevalence_75plus))
expected_ratio_75plus <- (census_amount_75plus*prevalence_75plus)/((census_amount_3549*prevalence_1864)+(census_amount_6574*prevalence_6574)+(census_amount_75plus*prevalence_75plus))

expected_ratios <- c(expected_ratio_1864,
                     expected_ratio_6574,
                     expected_ratio_75plus)
round(expected_ratios,2)
```

```
## [1] 0.60 0.20 0.21
```

```
KODAP_data_complete_any_mood_disorder <- KODAP_data_complete_s2[KODAP_data_complete_s2$ICD1_pre_recode %in% c("F30.X F31.X Manische Episode oder Bipolare Störungen",
                                                                                                              "F31.7 Bipolare affktive Störung, gegenwärtig remittiert",
                                                                                                              "F32 Depressive Episode",
                                                                                                              "F33 Rezidivierende depressive Störung",
                                                                                                              "F33.4 Rezidivierende depressive Störung, gegenwärtig remittiert",
                                                                                                              "F34 Anhaltende affektive Störungen",
                                                                                                              "F38.X Andere affektive Störung") |
                                                                  KODAP_data_complete_s2$ICD2_pre_recode %in% c("F30.X F31.X Manische Episode oder Bipolare Störungen",
                                                                                                                "F31.7 Bipolare affktive Störung, gegenwärtig remittiert",
                                                                                                                "F32 Depressive Episode",
                                                                                                                "F33 Rezidivierende depressive Störung",
                                                                                                                "F33.4 Rezidivierende depressive Störung, gegenwärtig remittiert",
                                                                                                                "F34 Anhaltende affektive Störungen",
                                                                                                                "F38.X Andere affektive Störung") |
                                                                  KODAP_data_complete_s2$ICD3_pre_recode %in% c("F30.X F31.X Manische Episode oder Bipolare Störungen",
                                                                                                                "F31.7 Bipolare affktive Störung, gegenwärtig remittiert",
                                                                                                                "F32 Depressive Episode",
                                                                                                                "F33 Rezidivierende depressive Störung",
                                                                                                                "F33.4 Rezidivierende depressive Störung, gegenwärtig remittiert",
                                                                                                                "F34 Anhaltende affektive Störungen",
                                                                                                                "F38.X Andere affektive Störung") |
                                                                  KODAP_data_complete_s2$ICD4_pre_recode %in% c("F30.X F31.X Manische Episode oder Bipolare Störungen",
                                                                                                                "F31.7 Bipolare affktive Störung, gegenwärtig remittiert",
                                                                                                                "F32 Depressive Episode",
                                                                                                                "F33 Rezidivierende depressive Störung",
                                                                                                                "F33.4 Rezidivierende depressive Störung, gegenwärtig remittiert",
                                                                                                                "F34 Anhaltende affektive Störungen",
                                                                                                                "F38.X Andere affektive Störung") |
                                                                  KODAP_data_complete_s2$ICD5_pre_recode %in% c("F30.X F31.X Manische Episode oder Bipolare Störungen",
                                                                                                                "F31.7 Bipolare affktive Störung, gegenwärtig remittiert",
                                                                                                                "F32 Depressive Episode",
                                                                                                                "F33 Rezidivierende depressive Störung",
                                                                                                                "F33.4 Rezidivierende depressive Störung, gegenwärtig remittiert",
                                                                                                                "F34 Anhaltende affektive Störungen",
                                                                                                                "F38.X Andere affektive Störung"),]

length(KODAP_data_complete_any_mood_disorder$Patient_ID)
```

```
## [1] 2094
```

```
#Observed vs. expected: 
Observation <- as.vector(table(KODAP_data_complete_any_mood_disorder$Age_Stepped))
Expected <- expected_ratios*length(KODAP_data_complete_any_mood_disorder$Patient_ID)

Observation
```

```
## [1] 1877  179   38
```

```
round(Observation/length(KODAP_data_complete_any_mood_disorder$Patient_ID)*100,1)
```

```
## [1] 89.6  8.5  1.8
```

```
# Chi-squared test for given probabilities
test <- chisq.test(Observation, p = expected_ratios)
test
```

```
## 
##  Chi-squared test for given probabilities
## 
## data:  Observation
## X-squared = 806.85, df = 2, p-value < 2.2e-16
```

```
# Post-hoc binomial-test for each category with Bonferroni-correction
binom.test(Observation[1], sum(Observation), expected_ratios[1])$p.value*3
```

```
## [1] 3.876927e-205
```

```
binom.test(Observation[2], sum(Observation), expected_ratios[2])$p.value*3
```

```
## [1] 1.041346e-44
```

```
binom.test(Observation[3], sum(Observation), expected_ratios[3])$p.value*3
```

```
## [1] 6.729488e-151
```

```
#Estimating underrepresentation compared to reference population
#Representation quotients
Observation/Expected
```

```
## [1] 1.50235586 0.43342028 0.08803738
```

```
#Confidence-intervals of Representation quotients
#Confidence-intervals around sample proportion
prop.test(x = Observation[2], n = sum(Observation), conf.level = .95)
```

```
## 
##  1-sample proportions test with continuity correction
## 
## data:  Observation[2] out of sum(Observation), null probability 0.5
## X-squared = 1437.5, df = 1, p-value < 2.2e-16
## alternative hypothesis: true p is not equal to 0.5
## 95 percent confidence interval:
##  0.07402955 0.09848338
## sample estimates:
##          p 
## 0.08548233
```

```
#Confidence-intervals around sample proportion relative to expected proportion
round(prop.test(x = Observation[2], n = sum(Observation), conf.level = .95)$estimate/expected_ratio_6574,2)
```

```
##    p 
## 0.43
```

```
round(as.vector(prop.test(x = Observation[2], n = sum(Observation), conf.level = .95)$conf.int)/expected_ratio_6574,2)
```

```
## [1] 0.38 0.50
```

```
#Confidence-intervals around sample proportion
prop.test(x = Observation[3], n = sum(Observation), conf.level = .95)
```

```
## 
##  1-sample proportions test with continuity correction
## 
## data:  Observation[3] out of sum(Observation), null probability 0.5
## X-squared = 1942.8, df = 1, p-value < 2.2e-16
## alternative hypothesis: true p is not equal to 0.5
## 95 percent confidence interval:
##  0.01304785 0.02508378
## sample estimates:
##          p 
## 0.01814709
```

```
#Confidence-intervals around sample proportion relative to expected proportion
round(prop.test(x = Observation[3], n = sum(Observation), conf.level = .95)$estimate/expected_ratio_75plus,2)
```

```
##    p 
## 0.09
```

```
round(as.vector(prop.test(x = Observation[3], n = sum(Observation), conf.level = .95)$conf.int)/expected_ratio_75plus,2)
```

```
## [1] 0.06 0.12
```

```
#50-64 years
prevalence_1834 <- as.numeric(Prevalence_estimates[Prevalence_estimates$Diagnosis == "Any mood disorder", "1834y"])
prevalence_3549 <- as.numeric(Prevalence_estimates[Prevalence_estimates$Diagnosis == "Any mood disorder", "3549y"])
prevalence_5064 <- as.numeric(Prevalence_estimates[Prevalence_estimates$Diagnosis == "Any mood disorder", "5064y"])

prevalence_1864 <- prevalence_5064

prevalence_6574 <- as.numeric(Prevalence_estimates[Prevalence_estimates$Diagnosis == "Any mood disorder", "6574y"])
prevalence_75plus <- as.numeric(Prevalence_estimates[Prevalence_estimates$Diagnosis == "Any mood disorder", "75yplus"])

#Ratios in reference population
expected_ratio_1864 <- (census_amount_5064*prevalence_1864)/((census_amount_5064*prevalence_1864)+(census_amount_6574*prevalence_6574)+(census_amount_75plus*prevalence_75plus))
expected_ratio_6574 <- (census_amount_6574*prevalence_6574)/((census_amount_5064*prevalence_1864)+(census_amount_6574*prevalence_6574)+(census_amount_75plus*prevalence_75plus))
expected_ratio_75plus <- (census_amount_75plus*prevalence_75plus)/((census_amount_5064*prevalence_1864)+(census_amount_6574*prevalence_6574)+(census_amount_75plus*prevalence_75plus))

expected_ratios <- c(expected_ratio_1864,
                     expected_ratio_6574,
                     expected_ratio_75plus)
round(expected_ratios,2)
```

```
## [1] 0.55 0.22 0.23
```

```
KODAP_data_complete_any_mood_disorder <- KODAP_data_complete_s3[KODAP_data_complete_s3$ICD1_pre_recode %in% c("F30.X F31.X Manische Episode oder Bipolare Störungen",
                                                                                                              "F31.7 Bipolare affktive Störung, gegenwärtig remittiert",
                                                                                                              "F32 Depressive Episode",
                                                                                                              "F33 Rezidivierende depressive Störung",
                                                                                                              "F33.4 Rezidivierende depressive Störung, gegenwärtig remittiert",
                                                                                                              "F34 Anhaltende affektive Störungen",
                                                                                                              "F38.X Andere affektive Störung") |
                                                                  KODAP_data_complete_s3$ICD2_pre_recode %in% c("F30.X F31.X Manische Episode oder Bipolare Störungen",
                                                                                                                "F31.7 Bipolare affktive Störung, gegenwärtig remittiert",
                                                                                                                "F32 Depressive Episode",
                                                                                                                "F33 Rezidivierende depressive Störung",
                                                                                                                "F33.4 Rezidivierende depressive Störung, gegenwärtig remittiert",
                                                                                                                "F34 Anhaltende affektive Störungen",
                                                                                                                "F38.X Andere affektive Störung") |
                                                                  KODAP_data_complete_s3$ICD3_pre_recode %in% c("F30.X F31.X Manische Episode oder Bipolare Störungen",
                                                                                                                "F31.7 Bipolare affktive Störung, gegenwärtig remittiert",
                                                                                                                "F32 Depressive Episode",
                                                                                                                "F33 Rezidivierende depressive Störung",
                                                                                                                "F33.4 Rezidivierende depressive Störung, gegenwärtig remittiert",
                                                                                                                "F34 Anhaltende affektive Störungen",
                                                                                                                "F38.X Andere affektive Störung") |
                                                                  KODAP_data_complete_s3$ICD4_pre_recode %in% c("F30.X F31.X Manische Episode oder Bipolare Störungen",
                                                                                                                "F31.7 Bipolare affktive Störung, gegenwärtig remittiert",
                                                                                                                "F32 Depressive Episode",
                                                                                                                "F33 Rezidivierende depressive Störung",
                                                                                                                "F33.4 Rezidivierende depressive Störung, gegenwärtig remittiert",
                                                                                                                "F34 Anhaltende affektive Störungen",
                                                                                                                "F38.X Andere affektive Störung") |
                                                                  KODAP_data_complete_s3$ICD5_pre_recode %in% c("F30.X F31.X Manische Episode oder Bipolare Störungen",
                                                                                                                "F31.7 Bipolare affktive Störung, gegenwärtig remittiert",
                                                                                                                "F32 Depressive Episode",
                                                                                                                "F33 Rezidivierende depressive Störung",
                                                                                                                "F33.4 Rezidivierende depressive Störung, gegenwärtig remittiert",
                                                                                                                "F34 Anhaltende affektive Störungen",
                                                                                                                "F38.X Andere affektive Störung"),]

length(KODAP_data_complete_any_mood_disorder$Patient_ID)
```

```
## [1] 1903
```

```
#Observed vs. expected: 
Observation <- as.vector(table(KODAP_data_complete_any_mood_disorder$Age_Stepped))
Expected <- expected_ratios*length(KODAP_data_complete_any_mood_disorder$Patient_ID)

Observation
```

```
## [1] 1686  179   38
```

```
round(Observation/length(KODAP_data_complete_any_mood_disorder$Patient_ID)*100,1)
```

```
## [1] 88.6  9.4  2.0
```

```
# Chi-squared test for given probabilities
test <- chisq.test(Observation, p = expected_ratios)
test
```

```
## 
##  Chi-squared test for given probabilities
## 
## data:  Observation
## X-squared = 885.1, df = 2, p-value < 2.2e-16
```

```
# Post-hoc binomial-test for each category with Bonferroni-correction
binom.test(Observation[1], sum(Observation), expected_ratios[1])$p.value*3
```

```
## [1] 1.664017e-219
```

```
binom.test(Observation[2], sum(Observation), expected_ratios[2])$p.value*3
```

```
## [1] 8.151518e-47
```

```
binom.test(Observation[3], sum(Observation), expected_ratios[3])$p.value*3
```

```
## [1] 2.15282e-155
```

```
#Estimating underrepresentation compared to reference population
#Representation quotients
Observation/Expected
```

```
## [1] 1.60616664 0.42901808 0.08714319
```

```
#Confidence-intervals of Representation quotients
#Confidence-intervals around sample proportion
prop.test(x = Observation[2], n = sum(Observation), conf.level = .95)
```

```
## 
##  1-sample proportions test with continuity correction
## 
## data:  Observation[2] out of sum(Observation), null probability 0.5
## X-squared = 1252.7, df = 1, p-value < 2.2e-16
## alternative hypothesis: true p is not equal to 0.5
## 95 percent confidence interval:
##  0.08150615 0.10828611
## sample estimates:
##          p 
## 0.09406201
```

```
#Confidence-intervals around sample proportion relative to expected proportion
round(prop.test(x = Observation[2], n = sum(Observation), conf.level = .95)$estimate/expected_ratio_6574,2)
```

```
##    p 
## 0.43
```

```
round(as.vector(prop.test(x = Observation[2], n = sum(Observation), conf.level = .95)$conf.int)/expected_ratio_6574,2)
```

```
## [1] 0.37 0.49
```

```
#Confidence-intervals around sample proportion
prop.test(x = Observation[3], n = sum(Observation), conf.level = .95)
```

```
## 
##  1-sample proportions test with continuity correction
## 
## data:  Observation[3] out of sum(Observation), null probability 0.5
## X-squared = 1752.1, df = 1, p-value < 2.2e-16
## alternative hypothesis: true p is not equal to 0.5
## 95 percent confidence interval:
##  0.01436043 0.02759045
## sample estimates:
##          p 
## 0.01996847
```

```
#Confidence-intervals around sample proportion relative to expected proportion
round(prop.test(x = Observation[3], n = sum(Observation), conf.level = .95)$estimate/expected_ratio_75plus,2)
```

```
##    p 
## 0.09
```

```
round(as.vector(prop.test(x = Observation[3], n = sum(Observation), conf.level = .95)$conf.int)/expected_ratio_75plus,2)
```

```
## [1] 0.06 0.12
```

```
###########################
#Major Depressive Disorder#
###########################
prevalence_1834 <- as.numeric(Prevalence_estimates[Prevalence_estimates$Diagnosis == "Major Depressive Disorder", "1834y"])
prevalence_3549 <- as.numeric(Prevalence_estimates[Prevalence_estimates$Diagnosis == "Major Depressive Disorder", "3549y"])
prevalence_5064 <- as.numeric(Prevalence_estimates[Prevalence_estimates$Diagnosis == "Major Depressive Disorder", "5064y"])
prevalence_6574 <- as.numeric(Prevalence_estimates[Prevalence_estimates$Diagnosis == "Major Depressive Disorder", "6574y"])
prevalence_75plus <- as.numeric(Prevalence_estimates[Prevalence_estimates$Diagnosis == "Major Depressive Disorder", "75yplus"])

expected_ratio_1834 <- (census_amount_1834*prevalence_1834)/((census_amount_1834*prevalence_1834)+(census_amount_3549*prevalence_3549)+(census_amount_5064*prevalence_5064)+(census_amount_6574*prevalence_6574)+(census_amount_75plus*prevalence_75plus))
expected_ratio_3549 <- (census_amount_3549*prevalence_3549)/((census_amount_1834*prevalence_1834)+(census_amount_3549*prevalence_3549)+(census_amount_5064*prevalence_5064)+(census_amount_6574*prevalence_6574)+(census_amount_75plus*prevalence_75plus))
expected_ratio_5064 <- (census_amount_5064*prevalence_5064)/((census_amount_1834*prevalence_1834)+(census_amount_3549*prevalence_3549)+(census_amount_5064*prevalence_5064)+(census_amount_6574*prevalence_6574)+(census_amount_75plus*prevalence_75plus))
expected_ratio_6574 <- (census_amount_6574*prevalence_6574)/((census_amount_1834*prevalence_1834)+(census_amount_3549*prevalence_3549)+(census_amount_5064*prevalence_5064)+(census_amount_6574*prevalence_6574)+(census_amount_75plus*prevalence_75plus))
expected_ratio_75plus <- (census_amount_75plus*prevalence_75plus)/((census_amount_1834*prevalence_1834)+(census_amount_3549*prevalence_3549)+(census_amount_5064*prevalence_5064)+(census_amount_6574*prevalence_6574)+(census_amount_75plus*prevalence_75plus))

round(c(expected_ratio_1834,expected_ratio_3549,expected_ratio_5064,expected_ratio_6574,expected_ratio_75plus), 2)
```

```
## [1] 0.36 0.24 0.22 0.09 0.09
```

```
KODAP_data_complete_MDD <- KODAP_data_complete[KODAP_data_complete$ICD1_pre_recode %in% c("F32 Depressive Episode",
                                                                                                        "F33 Rezidivierende depressive Störung",
                                                                                                        "F33.4 Rezidivierende depressive Störung, gegenwärtig remittiert") |
                                                               KODAP_data_complete$ICD2_pre_recode %in% c("F32 Depressive Episode",
                                                                                                          "F33 Rezidivierende depressive Störung",
                                                                                                          "F33.4 Rezidivierende depressive Störung, gegenwärtig remittiert") |
                                                               KODAP_data_complete$ICD3_pre_recode %in% c("F32 Depressive Episode",
                                                                                                          "F33 Rezidivierende depressive Störung",
                                                                                                          "F33.4 Rezidivierende depressive Störung, gegenwärtig remittiert") |
                                                               KODAP_data_complete$ICD4_pre_recode %in% c("F32 Depressive Episode",
                                                                                                          "F33 Rezidivierende depressive Störung",
                                                                                                          "F33.4 Rezidivierende depressive Störung, gegenwärtig remittiert") |
                                                               KODAP_data_complete$ICD5_pre_recode %in% c("F32 Depressive Episode",
                                                                                                          "F33 Rezidivierende depressive Störung",
                                                                                                          "F33.4 Rezidivierende depressive Störung, gegenwärtig remittiert"),]

table(KODAP_data_complete_MDD$Age_Stepped_2)
```

```
## 
##    0    1    2    3    4 
## 4050 1696 1515  163   36
```

```
round(table(KODAP_data_complete_MDD$Age_Stepped_2)/length(KODAP_data_complete_MDD$Age_Stepped_2)*100, 1)
```

```
## 
##    0    1    2    3    4 
## 54.3 22.7 20.3  2.2  0.5
```

```
#18-34 years
prevalence_1834 <- as.numeric(Prevalence_estimates[Prevalence_estimates$Diagnosis == "Major Depressive Disorder", "1834y"])
prevalence_3549 <- as.numeric(Prevalence_estimates[Prevalence_estimates$Diagnosis == "Major Depressive Disorder", "3549y"])
prevalence_5064 <- as.numeric(Prevalence_estimates[Prevalence_estimates$Diagnosis == "Major Depressive Disorder", "5064y"])

prevalence_1864 <- prevalence_1834

prevalence_6574 <- as.numeric(Prevalence_estimates[Prevalence_estimates$Diagnosis == "Major Depressive Disorder", "6574y"])
prevalence_75plus <- as.numeric(Prevalence_estimates[Prevalence_estimates$Diagnosis == "Major Depressive Disorder", "75yplus"])

#Ratios in reference population
expected_ratio_1864 <- (census_amount_1834*prevalence_1864)/((census_amount_1834*prevalence_1864)+(census_amount_6574*prevalence_6574)+(census_amount_75plus*prevalence_75plus))
expected_ratio_6574 <- (census_amount_6574*prevalence_6574)/((census_amount_1834*prevalence_1864)+(census_amount_6574*prevalence_6574)+(census_amount_75plus*prevalence_75plus))
expected_ratio_75plus <- (census_amount_75plus*prevalence_75plus)/((census_amount_1834*prevalence_1864)+(census_amount_6574*prevalence_6574)+(census_amount_75plus*prevalence_75plus))

expected_ratios <- c(expected_ratio_1864,
                     expected_ratio_6574,
                     expected_ratio_75plus)
round(expected_ratios,2)
```

```
## [1] 0.67 0.16 0.17
```

```
KODAP_data_complete_MDD <- KODAP_data_complete_s1[KODAP_data_complete_s1$ICD1_pre_recode %in% c("F32 Depressive Episode",
                                                                                                              "F33 Rezidivierende depressive Störung",
                                                                                                              "F33.4 Rezidivierende depressive Störung, gegenwärtig remittiert") |
                                                                  KODAP_data_complete_s1$ICD2_pre_recode %in% c("F32 Depressive Episode",
                                                                                                                "F33 Rezidivierende depressive Störung",
                                                                                                                "F33.4 Rezidivierende depressive Störung, gegenwärtig remittiert") |
                                                                  KODAP_data_complete_s1$ICD3_pre_recode %in% c("F32 Depressive Episode",
                                                                                                                "F33 Rezidivierende depressive Störung",
                                                                                                                "F33.4 Rezidivierende depressive Störung, gegenwärtig remittiert") |
                                                                  KODAP_data_complete_s1$ICD4_pre_recode %in% c("F32 Depressive Episode",
                                                                                                                "F33 Rezidivierende depressive Störung",
                                                                                                                "F33.4 Rezidivierende depressive Störung, gegenwärtig remittiert") |
                                                                  KODAP_data_complete_s1$ICD5_pre_recode %in% c("F32 Depressive Episode",
                                                                                                                "F33 Rezidivierende depressive Störung",
                                                                                                                "F33.4 Rezidivierende depressive Störung, gegenwärtig remittiert"),]

length(KODAP_data_complete_MDD$Patient_ID)
```

```
## [1] 4249
```

```
#Observed vs. expected: 
Observation <- as.vector(table(KODAP_data_complete_MDD$Age_Stepped))
Expected <- expected_ratios*length(KODAP_data_complete_MDD$Patient_ID)

Observation
```

```
## [1] 4050  163   36
```

```
round(Observation/length(KODAP_data_complete_MDD$Patient_ID)*100,1)
```

```
## [1] 95.3  3.8  0.8
```

```
# Chi-squared test for given probabilities
test <- chisq.test(Observation, p = expected_ratios)
test
```

```
## 
##  Chi-squared test for given probabilities
## 
## data:  Observation
## X-squared = 1523.7, df = 2, p-value < 2.2e-16
```

```
# Post-hoc binomial-test for each category with Bonferroni-correction
binom.test(Observation[1], sum(Observation), expected_ratios[1])$p.value*3
```

```
## [1] 1.482197e-323
```

```
binom.test(Observation[2], sum(Observation), expected_ratios[2])$p.value*3
```

```
## [1] 1.227732e-139
```

```
binom.test(Observation[3], sum(Observation), expected_ratios[3])$p.value*3
```

```
## [1] 6.112485e-273
```

```
#Estimating underrepresentation compared to reference population
#Representation quotients
Observation/Expected
```

```
## [1] 1.41523310 0.24029538 0.05077941
```

```
#Confidence-intervals of Representation quotients
#Confidence-intervals around sample proportion
prop.test(x = Observation[2], n = sum(Observation), conf.level = .95)
```

```
## 
##  1-sample proportions test with continuity correction
## 
## data:  Observation[2] out of sum(Observation), null probability 0.5
## X-squared = 3620.2, df = 1, p-value < 2.2e-16
## alternative hypothesis: true p is not equal to 0.5
## 95 percent confidence interval:
##  0.03288231 0.04469253
## sample estimates:
##          p 
## 0.03836197
```

```
#Confidence-intervals around sample proportion relative to expected proportion
round(prop.test(x = Observation[2], n = sum(Observation), conf.level = .95)$estimate/expected_ratio_6574,2)
```

```
##    p 
## 0.24
```

```
round(as.vector(prop.test(x = Observation[2], n = sum(Observation), conf.level = .95)$conf.int)/expected_ratio_6574,2)
```

```
## [1] 0.21 0.28
```

```
#Confidence-intervals around sample proportion
prop.test(x = Observation[3], n = sum(Observation), conf.level = .95)
```

```
## 
##  1-sample proportions test with continuity correction
## 
## data:  Observation[3] out of sum(Observation), null probability 0.5
## X-squared = 4104.3, df = 1, p-value < 2.2e-16
## alternative hypothesis: true p is not equal to 0.5
## 95 percent confidence interval:
##  0.006027553 0.011842989
## sample estimates:
##           p 
## 0.008472582
```

```
#Confidence-intervals around sample proportion relative to expected proportion
round(prop.test(x = Observation[3], n = sum(Observation), conf.level = .95)$estimate/expected_ratio_75plus,2)
```

```
##    p 
## 0.05
```

```
round(as.vector(prop.test(x = Observation[3], n = sum(Observation), conf.level = .95)$conf.int)/expected_ratio_75plus,2)
```

```
## [1] 0.04 0.07
```

```
#35-49 years
prevalence_1834 <- as.numeric(Prevalence_estimates[Prevalence_estimates$Diagnosis == "Major Depressive Disorder", "1834y"])
prevalence_3549 <- as.numeric(Prevalence_estimates[Prevalence_estimates$Diagnosis == "Major Depressive Disorder", "3549y"])
prevalence_5064 <- as.numeric(Prevalence_estimates[Prevalence_estimates$Diagnosis == "Major Depressive Disorder", "5064y"])

prevalence_1864 <- prevalence_3549

prevalence_6574 <- as.numeric(Prevalence_estimates[Prevalence_estimates$Diagnosis == "Major Depressive Disorder", "6574y"])
prevalence_75plus <- as.numeric(Prevalence_estimates[Prevalence_estimates$Diagnosis == "Major Depressive Disorder", "75yplus"])

#Ratios in reference population
expected_ratio_1864 <- (census_amount_3549*prevalence_1864)/((census_amount_3549*prevalence_1864)+(census_amount_6574*prevalence_6574)+(census_amount_75plus*prevalence_75plus))
expected_ratio_6574 <- (census_amount_6574*prevalence_6574)/((census_amount_3549*prevalence_1864)+(census_amount_6574*prevalence_6574)+(census_amount_75plus*prevalence_75plus))
expected_ratio_75plus <- (census_amount_75plus*prevalence_75plus)/((census_amount_3549*prevalence_1864)+(census_amount_6574*prevalence_6574)+(census_amount_75plus*prevalence_75plus))

expected_ratios <- c(expected_ratio_1864,
                     expected_ratio_6574,
                     expected_ratio_75plus)
round(expected_ratios,2)
```

```
## [1] 0.58 0.20 0.21
```

```
KODAP_data_complete_MDD <- KODAP_data_complete_s2[KODAP_data_complete_s2$ICD1_pre_recode %in% c("F32 Depressive Episode",
                                                                                                              "F33 Rezidivierende depressive Störung",
                                                                                                              "F33.4 Rezidivierende depressive Störung, gegenwärtig remittiert") |
                                                                  KODAP_data_complete_s2$ICD2_pre_recode %in% c("F32 Depressive Episode",
                                                                                                                "F33 Rezidivierende depressive Störung",
                                                                                                                "F33.4 Rezidivierende depressive Störung, gegenwärtig remittiert") |
                                                                  KODAP_data_complete_s2$ICD3_pre_recode %in% c("F32 Depressive Episode",
                                                                                                                "F33 Rezidivierende depressive Störung",
                                                                                                                "F33.4 Rezidivierende depressive Störung, gegenwärtig remittiert") |
                                                                  KODAP_data_complete_s2$ICD4_pre_recode %in% c("F32 Depressive Episode",
                                                                                                                "F33 Rezidivierende depressive Störung",
                                                                                                                "F33.4 Rezidivierende depressive Störung, gegenwärtig remittiert") |
                                                                  KODAP_data_complete_s2$ICD5_pre_recode %in% c("F32 Depressive Episode",
                                                                                                                "F33 Rezidivierende depressive Störung",
                                                                                                                "F33.4 Rezidivierende depressive Störung, gegenwärtig remittiert"),]

length(KODAP_data_complete_MDD$Patient_ID)
```

```
## [1] 1895
```

```
#Observed vs. expected: 
Observation <- as.vector(table(KODAP_data_complete_MDD$Age_Stepped))
Expected <- expected_ratios*length(KODAP_data_complete_MDD$Patient_ID)

Observation
```

```
## [1] 1696  163   36
```

```
round(Observation/length(KODAP_data_complete_MDD$Patient_ID)*100,1)
```

```
## [1] 89.5  8.6  1.9
```

```
# Chi-squared test for given probabilities
test <- chisq.test(Observation, p = expected_ratios)
test
```

```
## 
##  Chi-squared test for given probabilities
## 
## data:  Observation
## X-squared = 789.29, df = 2, p-value < 2.2e-16
```

```
# Post-hoc binomial-test for each category with Bonferroni-correction
binom.test(Observation[1], sum(Observation), expected_ratios[1])$p.value*3
```

```
## [1] 3.244867e-200
```

```
binom.test(Observation[2], sum(Observation), expected_ratios[2])$p.value*3
```

```
## [1] 1.355558e-44
```

```
binom.test(Observation[3], sum(Observation), expected_ratios[3])$p.value*3
```

```
## [1] 2.743262e-142
```

```
#Estimating underrepresentation compared to reference population
#Representation quotients
Observation/Expected
```

```
## [1] 1.54048719 0.41981902 0.08871648
```

```
#Confidence-intervals of Representation quotients
#Confidence-intervals around sample proportion
prop.test(x = Observation[2], n = sum(Observation), conf.level = .95)
```

```
## 
##  1-sample proportions test with continuity correction
## 
## data:  Observation[2] out of sum(Observation), null probability 0.5
## X-squared = 1297.4, df = 1, p-value < 2.2e-16
## alternative hypothesis: true p is not equal to 0.5
## 95 percent confidence interval:
##  0.07396834 0.09977324
## sample estimates:
##          p 
## 0.08601583
```

```
#Confidence-intervals around sample proportion relative to expected proportion
round(prop.test(x = Observation[2], n = sum(Observation), conf.level = .95)$estimate/expected_ratio_6574,2)
```

```
##    p 
## 0.42
```

```
round(as.vector(prop.test(x = Observation[2], n = sum(Observation), conf.level = .95)$conf.int)/expected_ratio_6574,2)
```

```
## [1] 0.36 0.49
```

```
#Confidence-intervals around sample proportion
prop.test(x = Observation[3], n = sum(Observation), conf.level = .95)
```

```
## 
##  1-sample proportions test with continuity correction
## 
## data:  Observation[3] out of sum(Observation), null probability 0.5
## X-squared = 1751.8, df = 1, p-value < 2.2e-16
## alternative hypothesis: true p is not equal to 0.5
## 95 percent confidence interval:
##  0.01353163 0.02649171
## sample estimates:
##          p 
## 0.01899736
```

```
#Confidence-intervals around sample proportion relative to expected proportion
round(prop.test(x = Observation[3], n = sum(Observation), conf.level = .95)$estimate/expected_ratio_75plus,2)
```

```
##    p 
## 0.09
```

```
round(as.vector(prop.test(x = Observation[3], n = sum(Observation), conf.level = .95)$conf.int)/expected_ratio_75plus,2)
```

```
## [1] 0.06 0.12
```

```
#50-64 years
prevalence_1834 <- as.numeric(Prevalence_estimates[Prevalence_estimates$Diagnosis == "Major Depressive Disorder", "1834y"])
prevalence_3549 <- as.numeric(Prevalence_estimates[Prevalence_estimates$Diagnosis == "Major Depressive Disorder", "3549y"])
prevalence_5064 <- as.numeric(Prevalence_estimates[Prevalence_estimates$Diagnosis == "Major Depressive Disorder", "5064y"])

prevalence_1864 <- prevalence_5064

prevalence_6574 <- as.numeric(Prevalence_estimates[Prevalence_estimates$Diagnosis == "Major Depressive Disorder", "6574y"])
prevalence_75plus <- as.numeric(Prevalence_estimates[Prevalence_estimates$Diagnosis == "Major Depressive Disorder", "75yplus"])

#Ratios in reference population
expected_ratio_1864 <- (census_amount_5064*prevalence_1864)/((census_amount_5064*prevalence_1864)+(census_amount_6574*prevalence_6574)+(census_amount_75plus*prevalence_75plus))
expected_ratio_6574 <- (census_amount_6574*prevalence_6574)/((census_amount_5064*prevalence_1864)+(census_amount_6574*prevalence_6574)+(census_amount_75plus*prevalence_75plus))
expected_ratio_75plus <- (census_amount_75plus*prevalence_75plus)/((census_amount_5064*prevalence_1864)+(census_amount_6574*prevalence_6574)+(census_amount_75plus*prevalence_75plus))

expected_ratios <- c(expected_ratio_1864,
                     expected_ratio_6574,
                     expected_ratio_75plus)
round(expected_ratios,2)
```

```
## [1] 0.55 0.22 0.23
```

```
KODAP_data_complete_MDD <- KODAP_data_complete_s3[KODAP_data_complete_s3$ICD1_pre_recode %in% c("F32 Depressive Episode",
                                                                                                              "F33 Rezidivierende depressive Störung",
                                                                                                              "F33.4 Rezidivierende depressive Störung, gegenwärtig remittiert") |
                                                                  KODAP_data_complete_s3$ICD2_pre_recode %in% c("F32 Depressive Episode",
                                                                                                                "F33 Rezidivierende depressive Störung",
                                                                                                                "F33.4 Rezidivierende depressive Störung, gegenwärtig remittiert") |
                                                                  KODAP_data_complete_s3$ICD3_pre_recode %in% c("F32 Depressive Episode",
                                                                                                                "F33 Rezidivierende depressive Störung",
                                                                                                                "F33.4 Rezidivierende depressive Störung, gegenwärtig remittiert") |
                                                                  KODAP_data_complete_s3$ICD4_pre_recode %in% c("F32 Depressive Episode",
                                                                                                                "F33 Rezidivierende depressive Störung",
                                                                                                                "F33.4 Rezidivierende depressive Störung, gegenwärtig remittiert") |
                                                                  KODAP_data_complete_s3$ICD5_pre_recode %in% c("F32 Depressive Episode",
                                                                                                                "F33 Rezidivierende depressive Störung",
                                                                                                                "F33.4 Rezidivierende depressive Störung, gegenwärtig remittiert"),]

length(KODAP_data_complete_MDD$Patient_ID)
```

```
## [1] 1714
```

```
#Observed vs. expected: 
Observation <- as.vector(table(KODAP_data_complete_MDD$Age_Stepped))
Expected <- expected_ratios*length(KODAP_data_complete_MDD$Patient_ID)

Observation
```

```
## [1] 1515  163   36
```

```
round(Observation/length(KODAP_data_complete_MDD$Patient_ID)*100,1)
```

```
## [1] 88.4  9.5  2.1
```

```
# Chi-squared test for given probabilities
test <- chisq.test(Observation, p = expected_ratios)
test
```

```
## 
##  Chi-squared test for given probabilities
## 
## data:  Observation
## X-squared = 791.75, df = 2, p-value < 2.2e-16
```

```
# Post-hoc binomial-test for each category with Bonferroni-correction
binom.test(Observation[1], sum(Observation), expected_ratios[1])$p.value*3
```

```
## [1] 6.137925e-196
```

```
binom.test(Observation[2], sum(Observation), expected_ratios[2])$p.value*3
```

```
## [1] 1.023715e-41
```

```
binom.test(Observation[3], sum(Observation), expected_ratios[3])$p.value*3
```

```
## [1] 1.627408e-138
```

```
#Estimating underrepresentation compared to reference population
#Representation quotients
Observation/Expected
```

```
## [1] 1.60522031 0.43281648 0.09146312
```

```
#Confidence-intervals of Representation quotients
#Confidence-intervals around sample proportion
prop.test(x = Observation[2], n = sum(Observation), conf.level = .95)
```

```
## 
##  1-sample proportions test with continuity correction
## 
## data:  Observation[2] out of sum(Observation), null probability 0.5
## X-squared = 1122.4, df = 1, p-value < 2.2e-16
## alternative hypothesis: true p is not equal to 0.5
## 95 percent confidence interval:
##  0.08183082 0.11021635
## sample estimates:
##          p 
## 0.09509918
```

```
#Confidence-intervals around sample proportion relative to expected proportion
round(prop.test(x = Observation[2], n = sum(Observation), conf.level = .95)$estimate/expected_ratio_6574,2)
```

```
##    p 
## 0.43
```

```
round(as.vector(prop.test(x = Observation[2], n = sum(Observation), conf.level = .95)$conf.int)/expected_ratio_6574,2)
```

```
## [1] 0.37 0.50
```

```
#Confidence-intervals around sample proportion
prop.test(x = Observation[3], n = sum(Observation), conf.level = .95)
```

```
## 
##  1-sample proportions test with continuity correction
## 
## data:  Observation[3] out of sum(Observation), null probability 0.5
## X-squared = 1571.1, df = 1, p-value < 2.2e-16
## alternative hypothesis: true p is not equal to 0.5
## 95 percent confidence interval:
##  0.01496409 0.02927602
## sample estimates:
##         p 
## 0.0210035
```

```
#Confidence-intervals around sample proportion relative to expected proportion
round(prop.test(x = Observation[3], n = sum(Observation), conf.level = .95)$estimate/expected_ratio_75plus,2)
```

```
##    p 
## 0.09
```

```
round(as.vector(prop.test(x = Observation[3], n = sum(Observation), conf.level = .95)$conf.int)/expected_ratio_75plus,2)
```

```
## [1] 0.07 0.13
```

```
###########################
#Dysthymia#
###########################
prevalence_1834 <- as.numeric(Prevalence_estimates[Prevalence_estimates$Diagnosis == "Dysthymia", "1834y"])
prevalence_3549 <- as.numeric(Prevalence_estimates[Prevalence_estimates$Diagnosis == "Dysthymia", "3549y"])
prevalence_5064 <- as.numeric(Prevalence_estimates[Prevalence_estimates$Diagnosis == "Dysthymia", "5064y"])
prevalence_6574 <- as.numeric(Prevalence_estimates[Prevalence_estimates$Diagnosis == "Dysthymia", "6574y"])
prevalence_75plus <- as.numeric(Prevalence_estimates[Prevalence_estimates$Diagnosis == "Dysthymia", "75yplus"])

expected_ratio_1834 <- (census_amount_1834*prevalence_1834)/((census_amount_1834*prevalence_1834)+(census_amount_3549*prevalence_3549)+(census_amount_5064*prevalence_5064)+(census_amount_6574*prevalence_6574)+(census_amount_75plus*prevalence_75plus))
expected_ratio_3549 <- (census_amount_3549*prevalence_3549)/((census_amount_1834*prevalence_1834)+(census_amount_3549*prevalence_3549)+(census_amount_5064*prevalence_5064)+(census_amount_6574*prevalence_6574)+(census_amount_75plus*prevalence_75plus))
expected_ratio_5064 <- (census_amount_5064*prevalence_5064)/((census_amount_1834*prevalence_1834)+(census_amount_3549*prevalence_3549)+(census_amount_5064*prevalence_5064)+(census_amount_6574*prevalence_6574)+(census_amount_75plus*prevalence_75plus))
expected_ratio_6574 <- (census_amount_6574*prevalence_6574)/((census_amount_1834*prevalence_1834)+(census_amount_3549*prevalence_3549)+(census_amount_5064*prevalence_5064)+(census_amount_6574*prevalence_6574)+(census_amount_75plus*prevalence_75plus))
expected_ratio_75plus <- (census_amount_75plus*prevalence_75plus)/((census_amount_1834*prevalence_1834)+(census_amount_3549*prevalence_3549)+(census_amount_5064*prevalence_5064)+(census_amount_6574*prevalence_6574)+(census_amount_75plus*prevalence_75plus))

round(c(expected_ratio_1834,expected_ratio_3549,expected_ratio_5064,expected_ratio_6574,expected_ratio_75plus), 2)
```

```
## [1] 0.30 0.23 0.21 0.12 0.13
```

```
KODAP_data_complete_Dysthymia <- KODAP_data_complete[KODAP_data_complete$ICD1_pre_clean %in% c("F34.1", "F34.10") |
                                                       KODAP_data_complete$ICD2_pre_clean %in% c("F34.1", "F34.10") |
                                                       KODAP_data_complete$ICD3_pre_clean %in% c("F34.1", "F34.10") | 
                                                       KODAP_data_complete$ICD4_pre_clean %in% c("F34.1", "F34.10") | 
                                                       KODAP_data_complete$ICD5_pre_clean %in% c("F34.1", "F34.10"),]

table(KODAP_data_complete_Dysthymia$Age_Stepped_2)
```

```
## 
##   0   1   2   3   4 
## 508 224 203  22   1
```

```
round(table(KODAP_data_complete_Dysthymia$Age_Stepped_2)/length(KODAP_data_complete_Dysthymia$Age_Stepped_2)*100, 1)
```

```
## 
##    0    1    2    3    4 
## 53.0 23.4 21.2  2.3  0.1
```

```
#18-34 years
prevalence_1834 <- as.numeric(Prevalence_estimates[Prevalence_estimates$Diagnosis == "Dysthymia", "1834y"])
prevalence_3549 <- as.numeric(Prevalence_estimates[Prevalence_estimates$Diagnosis == "Dysthymia", "3549y"])
prevalence_5064 <- as.numeric(Prevalence_estimates[Prevalence_estimates$Diagnosis == "Dysthymia", "5064y"])

prevalence_1864 <- prevalence_1834

prevalence_6574 <- as.numeric(Prevalence_estimates[Prevalence_estimates$Diagnosis == "Dysthymia", "6574y"])
prevalence_75plus <- as.numeric(Prevalence_estimates[Prevalence_estimates$Diagnosis == "Dysthymia", "75yplus"])

#Ratios in reference population
expected_ratio_1864 <- (census_amount_1834*prevalence_1864)/((census_amount_1834*prevalence_1864)+(census_amount_6574*prevalence_6574)+(census_amount_75plus*prevalence_75plus))
expected_ratio_6574 <- (census_amount_6574*prevalence_6574)/((census_amount_1834*prevalence_1864)+(census_amount_6574*prevalence_6574)+(census_amount_75plus*prevalence_75plus))
expected_ratio_75plus <- (census_amount_75plus*prevalence_75plus)/((census_amount_1834*prevalence_1864)+(census_amount_6574*prevalence_6574)+(census_amount_75plus*prevalence_75plus))

expected_ratios <- c(expected_ratio_1864,
                     expected_ratio_6574,
                     expected_ratio_75plus)
round(expected_ratios,2)
```

```
## [1] 0.54 0.22 0.23
```

```
KODAP_data_complete_Dysthymia <- KODAP_data_complete_s1[KODAP_data_complete_s1$ICD1_pre_clean %in% c("F34.1", "F34.10") |
                                                          KODAP_data_complete_s1$ICD2_pre_clean %in% c("F34.1", "F34.10") |
                                                          KODAP_data_complete_s1$ICD3_pre_clean %in% c("F34.1", "F34.10") | 
                                                          KODAP_data_complete_s1$ICD4_pre_clean %in% c("F34.1", "F34.10") | 
                                                          KODAP_data_complete_s1$ICD5_pre_clean %in% c("F34.1", "F34.10"),]

length(KODAP_data_complete_Dysthymia$Patient_ID)
```

```
## [1] 531
```

```
#Observed vs. expected: 
Observation <- as.vector(table(KODAP_data_complete_Dysthymia$Age_Stepped))
Expected <- expected_ratios*length(KODAP_data_complete_Dysthymia$Patient_ID)

Observation
```

```
## [1] 508  22   1
```

```
round(Observation/length(KODAP_data_complete_Dysthymia$Patient_ID)*100,1)
```

```
## [1] 95.7  4.1  0.2
```

```
# Chi-squared test for given probabilities
test <- chisq.test(Observation, p = expected_ratios)
test
```

```
## 
##  Chi-squared test for given probabilities
## 
## data:  Observation
## X-squared = 367.05, df = 2, p-value < 2.2e-16
```

```
# Post-hoc binomial-test for each category with Bonferroni-correction
binom.test(Observation[1], sum(Observation), expected_ratios[1])$p.value*3
```

```
## [1] 2.008176e-102
```

```
binom.test(Observation[2], sum(Observation), expected_ratios[2])$p.value*3
```

```
## [1] 1.927829e-31
```

```
binom.test(Observation[3], sum(Observation), expected_ratios[3])$p.value*3
```

```
## [1] 5.032302e-59
```

```
#Estimating underrepresentation compared to reference population
#Representation quotients
Observation/Expected
```

```
## [1] 1.7597575 0.1856730 0.0080752
```

```
#Confidence-intervals of Representation quotients
#Confidence-intervals around sample proportion
prop.test(x = Observation[2], n = sum(Observation), conf.level = .95)
```

```
## 
##  1-sample proportions test with continuity correction
## 
## data:  Observation[2] out of sum(Observation), null probability 0.5
## X-squared = 444.81, df = 1, p-value < 2.2e-16
## alternative hypothesis: true p is not equal to 0.5
## 95 percent confidence interval:
##  0.02676267 0.06304503
## sample estimates:
##          p 
## 0.04143126
```

```
#Confidence-intervals around sample proportion relative to expected proportion
round(prop.test(x = Observation[2], n = sum(Observation), conf.level = .95)$estimate/expected_ratio_6574,2)
```

```
##    p 
## 0.19
```

```
round(as.vector(prop.test(x = Observation[2], n = sum(Observation), conf.level = .95)$conf.int)/expected_ratio_6574,2)
```

```
## [1] 0.12 0.28
```

```
#Confidence-intervals around sample proportion
prop.test(x = Observation[3], n = sum(Observation), conf.level = .95)
```

```
## 
##  1-sample proportions test with continuity correction
## 
## data:  Observation[3] out of sum(Observation), null probability 0.5
## X-squared = 525.02, df = 1, p-value < 2.2e-16
## alternative hypothesis: true p is not equal to 0.5
## 95 percent confidence interval:
##  0.0000983132 0.0121389098
## sample estimates:
##           p 
## 0.001883239
```

```
#Confidence-intervals around sample proportion relative to expected proportion
round(prop.test(x = Observation[3], n = sum(Observation), conf.level = .95)$estimate/expected_ratio_75plus,2)
```

```
##    p 
## 0.01
```

```
round(as.vector(prop.test(x = Observation[3], n = sum(Observation), conf.level = .95)$conf.int)/expected_ratio_75plus,2)
```

```
## [1] 0.00 0.05
```

```
#35-49 years
prevalence_1834 <- as.numeric(Prevalence_estimates[Prevalence_estimates$Diagnosis == "Dysthymia", "1834y"])
prevalence_3549 <- as.numeric(Prevalence_estimates[Prevalence_estimates$Diagnosis == "Dysthymia", "3549y"])
prevalence_5064 <- as.numeric(Prevalence_estimates[Prevalence_estimates$Diagnosis == "Dysthymia", "5064y"])

prevalence_1864 <- prevalence_3549

prevalence_6574 <- as.numeric(Prevalence_estimates[Prevalence_estimates$Diagnosis == "Dysthymia", "6574y"])
prevalence_75plus <- as.numeric(Prevalence_estimates[Prevalence_estimates$Diagnosis == "Dysthymia", "75yplus"])

#Ratios in reference population
expected_ratio_1864 <- (census_amount_3549*prevalence_1864)/((census_amount_3549*prevalence_1864)+(census_amount_6574*prevalence_6574)+(census_amount_75plus*prevalence_75plus))
expected_ratio_6574 <- (census_amount_6574*prevalence_6574)/((census_amount_3549*prevalence_1864)+(census_amount_6574*prevalence_6574)+(census_amount_75plus*prevalence_75plus))
expected_ratio_75plus <- (census_amount_75plus*prevalence_75plus)/((census_amount_3549*prevalence_1864)+(census_amount_6574*prevalence_6574)+(census_amount_75plus*prevalence_75plus))

expected_ratios <- c(expected_ratio_1864,
                     expected_ratio_6574,
                     expected_ratio_75plus)
round(expected_ratios,2)
```

```
## [1] 0.47 0.26 0.27
```

```
KODAP_data_complete_Dysthymia <- KODAP_data_complete_s2[KODAP_data_complete_s2$ICD1_pre_clean %in% c("F34.1", "F34.10") |
                                                          KODAP_data_complete_s2$ICD2_pre_clean %in% c("F34.1", "F34.10") |
                                                          KODAP_data_complete_s2$ICD3_pre_clean %in% c("F34.1", "F34.10") | 
                                                          KODAP_data_complete_s2$ICD4_pre_clean %in% c("F34.1", "F34.10") | 
                                                          KODAP_data_complete_s2$ICD5_pre_clean %in% c("F34.1", "F34.10"),]

length(KODAP_data_complete_Dysthymia$Patient_ID)
```

```
## [1] 247
```

```
#Observed vs. expected: 
Observation <- as.vector(table(KODAP_data_complete_Dysthymia$Age_Stepped))
Expected <- expected_ratios*length(KODAP_data_complete_Dysthymia$Patient_ID)

Observation
```

```
## [1] 224  22   1
```

```
round(Observation/length(KODAP_data_complete_Dysthymia$Patient_ID)*100,1)
```

```
## [1] 90.7  8.9  0.4
```

```
# Chi-squared test for given probabilities
test <- chisq.test(Observation, p = expected_ratios)
test
```

```
## 
##  Chi-squared test for given probabilities
## 
## data:  Observation
## X-squared = 189.42, df = 2, p-value < 2.2e-16
```

```
# Post-hoc binomial-test for each category with Bonferroni-correction
binom.test(Observation[1], sum(Observation), expected_ratios[1])$p.value*3
```

```
## [1] 6.385702e-47
```

```
binom.test(Observation[2], sum(Observation), expected_ratios[2])$p.value*3
```

```
## [1] 1.240164e-10
```

```
binom.test(Observation[3], sum(Observation), expected_ratios[3])$p.value*3
```

```
## [1] 1.036754e-31
```

```
#Estimating underrepresentation compared to reference population
#Representation quotients
Observation/Expected
```

```
## [1] 1.91423590 0.34614774 0.01505449
```

```
#Confidence-intervals of Representation quotients
#Confidence-intervals around sample proportion
prop.test(x = Observation[2], n = sum(Observation), conf.level = .95)
```

```
## 
##  1-sample proportions test with continuity correction
## 
## data:  Observation[2] out of sum(Observation), null probability 0.5
## X-squared = 165.2, df = 1, p-value < 2.2e-16
## alternative hypothesis: true p is not equal to 0.5
## 95 percent confidence interval:
##  0.05791464 0.13350995
## sample estimates:
##          p 
## 0.08906883
```

```
#Confidence-intervals around sample proportion relative to expected proportion
round(prop.test(x = Observation[2], n = sum(Observation), conf.level = .95)$estimate/expected_ratio_6574,2)
```

```
##    p 
## 0.35
```

```
round(as.vector(prop.test(x = Observation[2], n = sum(Observation), conf.level = .95)$conf.int)/expected_ratio_6574,2)
```

```
## [1] 0.23 0.52
```

```
#Confidence-intervals around sample proportion
prop.test(x = Observation[3], n = sum(Observation), conf.level = .95)
```

```
## 
##  1-sample proportions test with continuity correction
## 
## data:  Observation[3] out of sum(Observation), null probability 0.5
## X-squared = 241.04, df = 1, p-value < 2.2e-16
## alternative hypothesis: true p is not equal to 0.5
## 95 percent confidence interval:
##  0.0002113729 0.0258703016
## sample estimates:
##           p 
## 0.004048583
```

```
#Confidence-intervals around sample proportion relative to expected proportion
round(prop.test(x = Observation[3], n = sum(Observation), conf.level = .95)$estimate/expected_ratio_75plus,2)
```

```
##    p 
## 0.02
```

```
round(as.vector(prop.test(x = Observation[3], n = sum(Observation), conf.level = .95)$conf.int)/expected_ratio_75plus,2)
```

```
## [1] 0.0 0.1
```

```
#50-64 years
prevalence_1834 <- as.numeric(Prevalence_estimates[Prevalence_estimates$Diagnosis == "Dysthymia", "1834y"])
prevalence_3549 <- as.numeric(Prevalence_estimates[Prevalence_estimates$Diagnosis == "Dysthymia", "3549y"])
prevalence_5064 <- as.numeric(Prevalence_estimates[Prevalence_estimates$Diagnosis == "Dysthymia", "5064y"])

prevalence_1864 <- prevalence_5064

prevalence_6574 <- as.numeric(Prevalence_estimates[Prevalence_estimates$Diagnosis == "Dysthymia", "6574y"])
prevalence_75plus <- as.numeric(Prevalence_estimates[Prevalence_estimates$Diagnosis == "Dysthymia", "75yplus"])

#Ratios in reference population
expected_ratio_1864 <- (census_amount_5064*prevalence_1864)/((census_amount_5064*prevalence_1864)+(census_amount_6574*prevalence_6574)+(census_amount_75plus*prevalence_75plus))
expected_ratio_6574 <- (census_amount_6574*prevalence_6574)/((census_amount_5064*prevalence_1864)+(census_amount_6574*prevalence_6574)+(census_amount_75plus*prevalence_75plus))
expected_ratio_75plus <- (census_amount_75plus*prevalence_75plus)/((census_amount_5064*prevalence_1864)+(census_amount_6574*prevalence_6574)+(census_amount_75plus*prevalence_75plus))

expected_ratios <- c(expected_ratio_1864,
                     expected_ratio_6574,
                     expected_ratio_75plus)
round(expected_ratios,2)
```

```
## [1] 0.46 0.27 0.28
```

```
KODAP_data_complete_Dysthymia <- KODAP_data_complete_s3[KODAP_data_complete_s3$ICD1_pre_clean %in% c("F34.1", "F34.10") |
                                                          KODAP_data_complete_s3$ICD2_pre_clean %in% c("F34.1", "F34.10") |
                                                          KODAP_data_complete_s3$ICD3_pre_clean %in% c("F34.1", "F34.10") | 
                                                          KODAP_data_complete_s3$ICD4_pre_clean %in% c("F34.1", "F34.10") | 
                                                          KODAP_data_complete_s3$ICD5_pre_clean %in% c("F34.1", "F34.10"),]

length(KODAP_data_complete_Dysthymia$Patient_ID)
```

```
## [1] 226
```

```
#Observed vs. expected: 
Observation <- as.vector(table(KODAP_data_complete_Dysthymia$Age_Stepped))
Expected <- expected_ratios*length(KODAP_data_complete_Dysthymia$Patient_ID)

Observation
```

```
## [1] 203  22   1
```

```
round(Observation/length(KODAP_data_complete_Dysthymia$Patient_ID)*100,1)
```

```
## [1] 89.8  9.7  0.4
```

```
# Chi-squared test for given probabilities
test <- chisq.test(Observation, p = expected_ratios)
test
```

```
## 
##  Chi-squared test for given probabilities
## 
## data:  Observation
## X-squared = 180.87, df = 2, p-value < 2.2e-16
```

```
# Post-hoc binomial-test for each category with Bonferroni-correction
binom.test(Observation[1], sum(Observation), expected_ratios[1])$p.value*3
```

```
## [1] 4.775104e-44
```

```
binom.test(Observation[2], sum(Observation), expected_ratios[2])$p.value*3
```

```
## [1] 1.603655e-09
```

```
binom.test(Observation[3], sum(Observation), expected_ratios[3])$p.value*3
```

```
## [1] 6.64903e-30
```

```
#Estimating underrepresentation compared to reference population
#Representation quotients
Observation/Expected
```

```
## [1] 1.9644403 0.3668026 0.0159528
```

```
#Confidence-intervals of Representation quotients
#Confidence-intervals around sample proportion
prop.test(x = Observation[2], n = sum(Observation), conf.level = .95)
```

```
## 
##  1-sample proportions test with continuity correction
## 
## data:  Observation[2] out of sum(Observation), null probability 0.5
## X-squared = 144.96, df = 1, p-value < 2.2e-16
## alternative hypothesis: true p is not equal to 0.5
## 95 percent confidence interval:
##  0.0633699 0.1455325
## sample estimates:
##          p 
## 0.09734513
```

```
#Confidence-intervals around sample proportion relative to expected proportion
round(prop.test(x = Observation[2], n = sum(Observation), conf.level = .95)$estimate/expected_ratio_6574,2)
```

```
##    p 
## 0.37
```

```
round(as.vector(prop.test(x = Observation[2], n = sum(Observation), conf.level = .95)$conf.int)/expected_ratio_6574,2)
```

```
## [1] 0.24 0.55
```

```
#Confidence-intervals around sample proportion
prop.test(x = Observation[3], n = sum(Observation), conf.level = .95)
```

```
## 
##  1-sample proportions test with continuity correction
## 
## data:  Observation[3] out of sum(Observation), null probability 0.5
## X-squared = 220.04, df = 1, p-value < 2.2e-16
## alternative hypothesis: true p is not equal to 0.5
## 95 percent confidence interval:
##  0.0002310174 0.0282316966
## sample estimates:
##           p 
## 0.004424779
```

```
#Confidence-intervals around sample proportion relative to expected proportion
round(prop.test(x = Observation[3], n = sum(Observation), conf.level = .95)$estimate/expected_ratio_75plus,2)
```

```
##    p 
## 0.02
```

```
round(as.vector(prop.test(x = Observation[3], n = sum(Observation), conf.level = .95)$conf.int)/expected_ratio_75plus,2)
```

```
## [1] 0.0 0.1
```

```
###########################
#Any anxiety disorder#
###########################
prevalence_1834 <- as.numeric(Prevalence_estimates[Prevalence_estimates$Diagnosis == "Any anxiety disorder", "1834y"])
prevalence_3549 <- as.numeric(Prevalence_estimates[Prevalence_estimates$Diagnosis == "Any anxiety disorder", "3549y"])
prevalence_5064 <- as.numeric(Prevalence_estimates[Prevalence_estimates$Diagnosis == "Any anxiety disorder", "5064y"])
prevalence_6574 <- as.numeric(Prevalence_estimates[Prevalence_estimates$Diagnosis == "Any anxiety disorder", "6574y"])
prevalence_75plus <- as.numeric(Prevalence_estimates[Prevalence_estimates$Diagnosis == "Any anxiety disorder", "75yplus"])

expected_ratio_1834 <- (census_amount_1834*prevalence_1834)/((census_amount_1834*prevalence_1834)+(census_amount_3549*prevalence_3549)+(census_amount_5064*prevalence_5064)+(census_amount_6574*prevalence_6574)+(census_amount_75plus*prevalence_75plus))
expected_ratio_3549 <- (census_amount_3549*prevalence_3549)/((census_amount_1834*prevalence_1834)+(census_amount_3549*prevalence_3549)+(census_amount_5064*prevalence_5064)+(census_amount_6574*prevalence_6574)+(census_amount_75plus*prevalence_75plus))
expected_ratio_5064 <- (census_amount_5064*prevalence_5064)/((census_amount_1834*prevalence_1834)+(census_amount_3549*prevalence_3549)+(census_amount_5064*prevalence_5064)+(census_amount_6574*prevalence_6574)+(census_amount_75plus*prevalence_75plus))
expected_ratio_6574 <- (census_amount_6574*prevalence_6574)/((census_amount_1834*prevalence_1834)+(census_amount_3549*prevalence_3549)+(census_amount_5064*prevalence_5064)+(census_amount_6574*prevalence_6574)+(census_amount_75plus*prevalence_75plus))
expected_ratio_75plus <- (census_amount_75plus*prevalence_75plus)/((census_amount_1834*prevalence_1834)+(census_amount_3549*prevalence_3549)+(census_amount_5064*prevalence_5064)+(census_amount_6574*prevalence_6574)+(census_amount_75plus*prevalence_75plus))

round(c(expected_ratio_1834,expected_ratio_3549,expected_ratio_5064,expected_ratio_6574,expected_ratio_75plus), 2)
```

```
## [1] 0.29 0.24 0.28 0.10 0.10
```

```
KODAP_data_complete_any_anx_disorder <- KODAP_data_complete[KODAP_data_complete$ICD1_pre_recode %in% c("F40.0X F41.0 Agoraphobie/Panikstörung",
                                                                                          "F40.1 Soziale Phobie",
                                                                                          "F40.2 Spezifische Phobie",
                                                                                          "F41.1 Generalisierte Angststörung",
                                                                                          "F41.X F40.9 Andere phobische oder Angststörungen") |
                                                 KODAP_data_complete$ICD2_pre_recode %in% c("F40.0X F41.0 Agoraphobie/Panikstörung",
                                                                                            "F40.1 Soziale Phobie",
                                                                                            "F40.2 Spezifische Phobie",
                                                                                            "F41.1 Generalisierte Angststörung",
                                                                                            "F41.X F40.9 Andere phobische oder Angststörungen") |
                                                 KODAP_data_complete$ICD3_pre_recode %in% c("F40.0X F41.0 Agoraphobie/Panikstörung",
                                                                                            "F40.1 Soziale Phobie",
                                                                                            "F40.2 Spezifische Phobie",
                                                                                            "F41.1 Generalisierte Angststörung",
                                                                                            "F41.X F40.9 Andere phobische oder Angststörungen") |
                                                 KODAP_data_complete$ICD4_pre_recode %in% c("F40.0X F41.0 Agoraphobie/Panikstörung",
                                                                                            "F40.1 Soziale Phobie",
                                                                                            "F40.2 Spezifische Phobie",
                                                                                            "F41.1 Generalisierte Angststörung",
                                                                                            "F41.X F40.9 Andere phobische oder Angststörungen") |
                                                 KODAP_data_complete$ICD5_pre_recode %in% c("F40.0X F41.0 Agoraphobie/Panikstörung",
                                                                                            "F40.1 Soziale Phobie",
                                                                                            "F40.2 Spezifische Phobie",
                                                                                            "F41.1 Generalisierte Angststörung",
                                                                                            "F41.X F40.9 Andere phobische oder Angststörungen"),]

table(KODAP_data_complete_any_anx_disorder$Age_Stepped_2)
```

```
## 
##    0    1    2    3    4 
## 2725  929  664  106   29
```

```
round(table(KODAP_data_complete_any_anx_disorder$Age_Stepped_2)/length(KODAP_data_complete_any_anx_disorder$Age_Stepped_2)*100, 1)
```

```
## 
##    0    1    2    3    4 
## 61.2 20.9 14.9  2.4  0.7
```

```
#18-34 years
prevalence_1834 <- as.numeric(Prevalence_estimates[Prevalence_estimates$Diagnosis == "Any anxiety disorder", "1834y"])
prevalence_3549 <- as.numeric(Prevalence_estimates[Prevalence_estimates$Diagnosis == "Any anxiety disorder", "3549y"])
prevalence_5064 <- as.numeric(Prevalence_estimates[Prevalence_estimates$Diagnosis == "Any anxiety disorder", "5064y"])

prevalence_1864 <- prevalence_1834

prevalence_6574 <- as.numeric(Prevalence_estimates[Prevalence_estimates$Diagnosis == "Any anxiety disorder", "6574y"])
prevalence_75plus <- as.numeric(Prevalence_estimates[Prevalence_estimates$Diagnosis == "Any anxiety disorder", "75yplus"])

#Ratios in reference population
expected_ratio_1864 <- (census_amount_1834*prevalence_1864)/((census_amount_1834*prevalence_1864)+(census_amount_6574*prevalence_6574)+(census_amount_75plus*prevalence_75plus))
expected_ratio_6574 <- (census_amount_6574*prevalence_6574)/((census_amount_1834*prevalence_1864)+(census_amount_6574*prevalence_6574)+(census_amount_75plus*prevalence_75plus))
expected_ratio_75plus <- (census_amount_75plus*prevalence_75plus)/((census_amount_1834*prevalence_1864)+(census_amount_6574*prevalence_6574)+(census_amount_75plus*prevalence_75plus))

expected_ratios <- c(expected_ratio_1864,
                     expected_ratio_6574,
                     expected_ratio_75plus)
round(expected_ratios,2)
```

```
## [1] 0.60 0.20 0.21
```

```
KODAP_data_complete_any_anx_disorder <- KODAP_data_complete_s1[KODAP_data_complete_s1$ICD1_pre_recode %in% c("F40.0X F41.0 Agoraphobie/Panikstörung",
                                                                                                              "F40.1 Soziale Phobie",
                                                                                                              "F40.2 Spezifische Phobie",
                                                                                                              "F41.1 Generalisierte Angststörung",
                                                                                                              "F41.X F40.9 Andere phobische oder Angststörungen") |
                                                                  KODAP_data_complete_s1$ICD2_pre_recode %in% c("F40.0X F41.0 Agoraphobie/Panikstörung",
                                                                                                                "F40.1 Soziale Phobie",
                                                                                                                "F40.2 Spezifische Phobie",
                                                                                                                "F41.1 Generalisierte Angststörung",
                                                                                                                "F41.X F40.9 Andere phobische oder Angststörungen") |
                                                                  KODAP_data_complete_s1$ICD3_pre_recode %in% c("F40.0X F41.0 Agoraphobie/Panikstörung",
                                                                                                                "F40.1 Soziale Phobie",
                                                                                                                "F40.2 Spezifische Phobie",
                                                                                                                "F41.1 Generalisierte Angststörung",
                                                                                                                "F41.X F40.9 Andere phobische oder Angststörungen") |
                                                                  KODAP_data_complete_s1$ICD4_pre_recode %in% c("F40.0X F41.0 Agoraphobie/Panikstörung",
                                                                                                                "F40.1 Soziale Phobie",
                                                                                                                "F40.2 Spezifische Phobie",
                                                                                                                "F41.1 Generalisierte Angststörung",
                                                                                                                "F41.X F40.9 Andere phobische oder Angststörungen") |
                                                                  KODAP_data_complete_s1$ICD5_pre_recode %in% c("F40.0X F41.0 Agoraphobie/Panikstörung",
                                                                                                                "F40.1 Soziale Phobie",
                                                                                                                "F40.2 Spezifische Phobie",
                                                                                                                "F41.1 Generalisierte Angststörung",
                                                                                                                "F41.X F40.9 Andere phobische oder Angststörungen"),]

length(KODAP_data_complete_any_anx_disorder$Patient_ID)
```

```
## [1] 2860
```

```
#Observed vs. expected: 
Observation <- as.vector(table(KODAP_data_complete_any_anx_disorder$Age_Stepped))
Expected <- expected_ratios*length(KODAP_data_complete_any_anx_disorder$Patient_ID)

Observation
```

```
## [1] 2725  106   29
```

```
round(Observation/length(KODAP_data_complete_any_anx_disorder$Patient_ID)*100,1)
```

```
## [1] 95.3  3.7  1.0
```

```
# Chi-squared test for given probabilities
test <- chisq.test(Observation, p = expected_ratios)
test
```

```
## 
##  Chi-squared test for given probabilities
## 
## data:  Observation
## X-squared = 1512, df = 2, p-value < 2.2e-16
```

```
# Post-hoc binomial-test for each category with Bonferroni-correction
binom.test(Observation[1], sum(Observation), expected_ratios[1])$p.value*3
```

```
## [1] 0
```

```
binom.test(Observation[2], sum(Observation), expected_ratios[2])$p.value*3
```

```
## [1] 9.249153e-142
```

```
binom.test(Observation[3], sum(Observation), expected_ratios[3])$p.value*3
```

```
## [1] 2.89704e-234
```

```
#Estimating underrepresentation compared to reference population
#Representation quotients
Observation/Expected
```

```
## [1] 1.59656533 0.18798327 0.04920833
```

```
#Confidence-intervals of Representation quotients
#Confidence-intervals around sample proportion
prop.test(x = Observation[2], n = sum(Observation), conf.level = .95)
```

```
## 
##  1-sample proportions test with continuity correction
## 
## data:  Observation[2] out of sum(Observation), null probability 0.5
## X-squared = 2449.9, df = 1, p-value < 2.2e-16
## alternative hypothesis: true p is not equal to 0.5
## 95 percent confidence interval:
##  0.03057815 0.04482088
## sample estimates:
##          p 
## 0.03706294
```

```
#Confidence-intervals around sample proportion relative to expected proportion
round(prop.test(x = Observation[2], n = sum(Observation), conf.level = .95)$estimate/expected_ratio_6574,2)
```

```
##    p 
## 0.19
```

```
round(as.vector(prop.test(x = Observation[2], n = sum(Observation), conf.level = .95)$conf.int)/expected_ratio_6574,2)
```

```
## [1] 0.16 0.23
```

```
#Confidence-intervals around sample proportion
prop.test(x = Observation[3], n = sum(Observation), conf.level = .95)
```

```
## 
##  1-sample proportions test with continuity correction
## 
## data:  Observation[3] out of sum(Observation), null probability 0.5
## X-squared = 2743.2, df = 1, p-value < 2.2e-16
## alternative hypothesis: true p is not equal to 0.5
## 95 percent confidence interval:
##  0.006925647 0.014729790
## sample estimates:
##          p 
## 0.01013986
```

```
#Confidence-intervals around sample proportion relative to expected proportion
round(prop.test(x = Observation[3], n = sum(Observation), conf.level = .95)$estimate/expected_ratio_75plus,2)
```

```
##    p 
## 0.05
```

```
round(as.vector(prop.test(x = Observation[3], n = sum(Observation), conf.level = .95)$conf.int)/expected_ratio_75plus,2)
```

```
## [1] 0.03 0.07
```

```
#35-49 years
prevalence_1834 <- as.numeric(Prevalence_estimates[Prevalence_estimates$Diagnosis == "Any anxiety disorder", "1834y"])
prevalence_3549 <- as.numeric(Prevalence_estimates[Prevalence_estimates$Diagnosis == "Any anxiety disorder", "3549y"])
prevalence_5064 <- as.numeric(Prevalence_estimates[Prevalence_estimates$Diagnosis == "Any anxiety disorder", "5064y"])

prevalence_1864 <- prevalence_3549

prevalence_6574 <- as.numeric(Prevalence_estimates[Prevalence_estimates$Diagnosis == "Any anxiety disorder", "6574y"])
prevalence_75plus <- as.numeric(Prevalence_estimates[Prevalence_estimates$Diagnosis == "Any anxiety disorder", "75yplus"])

#Ratios in reference population
expected_ratio_1864 <- (census_amount_3549*prevalence_1864)/((census_amount_3549*prevalence_1864)+(census_amount_6574*prevalence_6574)+(census_amount_75plus*prevalence_75plus))
expected_ratio_6574 <- (census_amount_6574*prevalence_6574)/((census_amount_3549*prevalence_1864)+(census_amount_6574*prevalence_6574)+(census_amount_75plus*prevalence_75plus))
expected_ratio_75plus <- (census_amount_75plus*prevalence_75plus)/((census_amount_3549*prevalence_1864)+(census_amount_6574*prevalence_6574)+(census_amount_75plus*prevalence_75plus))

expected_ratios <- c(expected_ratio_1864,
                     expected_ratio_6574,
                     expected_ratio_75plus)
round(expected_ratios,2)
```

```
## [1] 0.55 0.22 0.23
```

```
KODAP_data_complete_any_anx_disorder <- KODAP_data_complete_s2[KODAP_data_complete_s2$ICD1_pre_recode %in% c("F40.0X F41.0 Agoraphobie/Panikstörung",
                                                                                                              "F40.1 Soziale Phobie",
                                                                                                              "F40.2 Spezifische Phobie",
                                                                                                              "F41.1 Generalisierte Angststörung",
                                                                                                              "F41.X F40.9 Andere phobische oder Angststörungen") |
                                                                  KODAP_data_complete_s2$ICD2_pre_recode %in% c("F40.0X F41.0 Agoraphobie/Panikstörung",
                                                                                                                "F40.1 Soziale Phobie",
                                                                                                                "F40.2 Spezifische Phobie",
                                                                                                                "F41.1 Generalisierte Angststörung",
                                                                                                                "F41.X F40.9 Andere phobische oder Angststörungen") |
                                                                  KODAP_data_complete_s2$ICD3_pre_recode %in% c("F40.0X F41.0 Agoraphobie/Panikstörung",
                                                                                                                "F40.1 Soziale Phobie",
                                                                                                                "F40.2 Spezifische Phobie",
                                                                                                                "F41.1 Generalisierte Angststörung",
                                                                                                                "F41.X F40.9 Andere phobische oder Angststörungen") |
                                                                  KODAP_data_complete_s2$ICD4_pre_recode %in% c("F40.0X F41.0 Agoraphobie/Panikstörung",
                                                                                                                "F40.1 Soziale Phobie",
                                                                                                                "F40.2 Spezifische Phobie",
                                                                                                                "F41.1 Generalisierte Angststörung",
                                                                                                                "F41.X F40.9 Andere phobische oder Angststörungen") |
                                                                  KODAP_data_complete_s2$ICD5_pre_recode %in% c("F40.0X F41.0 Agoraphobie/Panikstörung",
                                                                                                                "F40.1 Soziale Phobie",
                                                                                                                "F40.2 Spezifische Phobie",
                                                                                                                "F41.1 Generalisierte Angststörung",
                                                                                                                "F41.X F40.9 Andere phobische oder Angststörungen"),]

length(KODAP_data_complete_any_anx_disorder$Patient_ID)
```

```
## [1] 1064
```

```
#Observed vs. expected: 
Observation <- as.vector(table(KODAP_data_complete_any_anx_disorder$Age_Stepped))
Expected <- expected_ratios*length(KODAP_data_complete_any_anx_disorder$Patient_ID)

Observation
```

```
## [1] 929 106  29
```

```
round(Observation/length(KODAP_data_complete_any_anx_disorder$Patient_ID)*100,1)
```

```
## [1] 87.3 10.0  2.7
```

```
# Chi-squared test for given probabilities
test <- chisq.test(Observation, p = expected_ratios)
test
```

```
## 
##  Chi-squared test for given probabilities
## 
## data:  Observation
## X-squared = 454.82, df = 2, p-value < 2.2e-16
```

```
# Post-hoc binomial-test for each category with Bonferroni-correction
binom.test(Observation[1], sum(Observation), expected_ratios[1])$p.value*3
```

```
## [1] 5.933046e-112
```

```
binom.test(Observation[2], sum(Observation), expected_ratios[2])$p.value*3
```

```
## [1] 6.070823e-24
```

```
binom.test(Observation[3], sum(Observation), expected_ratios[3])$p.value*3
```

```
## [1] 1.89698e-78
```

```
#Estimating underrepresentation compared to reference population
#Representation quotients
Observation/Expected
```

```
## [1] 1.5791805 0.4556972 0.1192878
```

```
#Confidence-intervals of Representation quotients
#Confidence-intervals around sample proportion
prop.test(x = Observation[2], n = sum(Observation), conf.level = .95)
```

```
## 
##  1-sample proportions test with continuity correction
## 
## data:  Observation[2] out of sum(Observation), null probability 0.5
## X-squared = 680.64, df = 1, p-value < 2.2e-16
## alternative hypothesis: true p is not equal to 0.5
## 95 percent confidence interval:
##  0.08261257 0.11959103
## sample estimates:
##          p 
## 0.09962406
```

```
#Confidence-intervals around sample proportion relative to expected proportion
round(prop.test(x = Observation[2], n = sum(Observation), conf.level = .95)$estimate/expected_ratio_6574,2)
```

```
##    p 
## 0.46
```

```
round(as.vector(prop.test(x = Observation[2], n = sum(Observation), conf.level = .95)$conf.int)/expected_ratio_6574,2)
```

```
## [1] 0.38 0.55
```

```
#Confidence-intervals around sample proportion
prop.test(x = Observation[3], n = sum(Observation), conf.level = .95)
```

```
## 
##  1-sample proportions test with continuity correction
## 
## data:  Observation[3] out of sum(Observation), null probability 0.5
## X-squared = 949.27, df = 1, p-value < 2.2e-16
## alternative hypothesis: true p is not equal to 0.5
## 95 percent confidence interval:
##  0.01865566 0.03941759
## sample estimates:
##          p 
## 0.02725564
```

```
#Confidence-intervals around sample proportion relative to expected proportion
round(prop.test(x = Observation[3], n = sum(Observation), conf.level = .95)$estimate/expected_ratio_75plus,2)
```

```
##    p 
## 0.12
```

```
round(as.vector(prop.test(x = Observation[3], n = sum(Observation), conf.level = .95)$conf.int)/expected_ratio_75plus,2)
```

```
## [1] 0.08 0.17
```

```
#50-64 years
prevalence_1834 <- as.numeric(Prevalence_estimates[Prevalence_estimates$Diagnosis == "Any anxiety disorder", "1834y"])
prevalence_3549 <- as.numeric(Prevalence_estimates[Prevalence_estimates$Diagnosis == "Any anxiety disorder", "3549y"])
prevalence_5064 <- as.numeric(Prevalence_estimates[Prevalence_estimates$Diagnosis == "Any anxiety disorder", "5064y"])

prevalence_1864 <- prevalence_5064

prevalence_6574 <- as.numeric(Prevalence_estimates[Prevalence_estimates$Diagnosis == "Any anxiety disorder", "6574y"])
prevalence_75plus <- as.numeric(Prevalence_estimates[Prevalence_estimates$Diagnosis == "Any anxiety disorder", "75yplus"])

#Ratios in reference population
expected_ratio_1864 <- (census_amount_5064*prevalence_1864)/((census_amount_5064*prevalence_1864)+(census_amount_6574*prevalence_6574)+(census_amount_75plus*prevalence_75plus))
expected_ratio_6574 <- (census_amount_6574*prevalence_6574)/((census_amount_5064*prevalence_1864)+(census_amount_6574*prevalence_6574)+(census_amount_75plus*prevalence_75plus))
expected_ratio_75plus <- (census_amount_75plus*prevalence_75plus)/((census_amount_5064*prevalence_1864)+(census_amount_6574*prevalence_6574)+(census_amount_75plus*prevalence_75plus))

expected_ratios <- c(expected_ratio_1864,
                     expected_ratio_6574,
                     expected_ratio_75plus)
round(expected_ratios,2)
```

```
## [1] 0.59 0.20 0.21
```

```
KODAP_data_complete_any_anx_disorder <- KODAP_data_complete_s3[KODAP_data_complete_s3$ICD1_pre_recode %in% c("F40.0X F41.0 Agoraphobie/Panikstörung",
                                                                                                              "F40.1 Soziale Phobie",
                                                                                                              "F40.2 Spezifische Phobie",
                                                                                                              "F41.1 Generalisierte Angststörung",
                                                                                                              "F41.X F40.9 Andere phobische oder Angststörungen") |
                                                                  KODAP_data_complete_s3$ICD2_pre_recode %in% c("F40.0X F41.0 Agoraphobie/Panikstörung",
                                                                                                                "F40.1 Soziale Phobie",
                                                                                                                "F40.2 Spezifische Phobie",
                                                                                                                "F41.1 Generalisierte Angststörung",
                                                                                                                "F41.X F40.9 Andere phobische oder Angststörungen") |
                                                                  KODAP_data_complete_s3$ICD3_pre_recode %in% c("F40.0X F41.0 Agoraphobie/Panikstörung",
                                                                                                                "F40.1 Soziale Phobie",
                                                                                                                "F40.2 Spezifische Phobie",
                                                                                                                "F41.1 Generalisierte Angststörung",
                                                                                                                "F41.X F40.9 Andere phobische oder Angststörungen") |
                                                                  KODAP_data_complete_s3$ICD4_pre_recode %in% c("F40.0X F41.0 Agoraphobie/Panikstörung",
                                                                                                                "F40.1 Soziale Phobie",
                                                                                                                "F40.2 Spezifische Phobie",
                                                                                                                "F41.1 Generalisierte Angststörung",
                                                                                                                "F41.X F40.9 Andere phobische oder Angststörungen") |
                                                                  KODAP_data_complete_s3$ICD5_pre_recode %in% c("F40.0X F41.0 Agoraphobie/Panikstörung",
                                                                                                                "F40.1 Soziale Phobie",
                                                                                                                "F40.2 Spezifische Phobie",
                                                                                                                "F41.1 Generalisierte Angststörung",
                                                                                                                "F41.X F40.9 Andere phobische oder Angststörungen"),]

length(KODAP_data_complete_any_anx_disorder$Patient_ID)
```

```
## [1] 799
```

```
#Observed vs. expected: 
Observation <- as.vector(table(KODAP_data_complete_any_anx_disorder$Age_Stepped))
Expected <- expected_ratios*length(KODAP_data_complete_any_anx_disorder$Patient_ID)

Observation
```

```
## [1] 664 106  29
```

```
round(Observation/length(KODAP_data_complete_any_anx_disorder$Patient_ID)*100,1)
```

```
## [1] 83.1 13.3  3.6
```

```
# Chi-squared test for given probabilities
test <- chisq.test(Observation, p = expected_ratios)
test
```

```
## 
##  Chi-squared test for given probabilities
## 
## data:  Observation
## X-squared = 213.78, df = 2, p-value < 2.2e-16
```

```
# Post-hoc binomial-test for each category with Bonferroni-correction
binom.test(Observation[1], sum(Observation), expected_ratios[1])$p.value*3
```

```
## [1] 1.280559e-48
```

```
binom.test(Observation[2], sum(Observation), expected_ratios[2])$p.value*3
```

```
## [1] 1.361843e-06
```

```
binom.test(Observation[3], sum(Observation), expected_ratios[3])$p.value*3
```

```
## [1] 1.612686e-45
```

```
#Estimating underrepresentation compared to reference population
#Representation quotients
Observation/Expected
```

```
## [1] 1.4125144 0.6590868 0.1725290
```

```
#Confidence-intervals of Representation quotients
#Confidence-intervals around sample proportion
prop.test(x = Observation[2], n = sum(Observation), conf.level = .95)
```

```
## 
##  1-sample proportions test with continuity correction
## 
## data:  Observation[2] out of sum(Observation), null probability 0.5
## X-squared = 429.78, df = 1, p-value < 2.2e-16
## alternative hypothesis: true p is not equal to 0.5
## 95 percent confidence interval:
##  0.1103173 0.1586226
## sample estimates:
##         p 
## 0.1326658
```

```
#Confidence-intervals around sample proportion relative to expected proportion
round(prop.test(x = Observation[2], n = sum(Observation), conf.level = .95)$estimate/expected_ratio_6574,2)
```

```
##    p 
## 0.66
```

```
round(as.vector(prop.test(x = Observation[2], n = sum(Observation), conf.level = .95)$conf.int)/expected_ratio_6574,2)
```

```
## [1] 0.55 0.79
```

```
#Confidence-intervals around sample proportion
prop.test(x = Observation[3], n = sum(Observation), conf.level = .95)
```

```
## 
##  1-sample proportions test with continuity correction
## 
## data:  Observation[3] out of sum(Observation), null probability 0.5
## X-squared = 685.36, df = 1, p-value < 2.2e-16
## alternative hypothesis: true p is not equal to 0.5
## 95 percent confidence interval:
##  0.02487130 0.05236749
## sample estimates:
##          p 
## 0.03629537
```

```
#Confidence-intervals around sample proportion relative to expected proportion
round(prop.test(x = Observation[3], n = sum(Observation), conf.level = .95)$estimate/expected_ratio_75plus,2)
```

```
##    p 
## 0.17
```

```
round(as.vector(prop.test(x = Observation[3], n = sum(Observation), conf.level = .95)$conf.int)/expected_ratio_75plus,2)
```

```
## [1] 0.12 0.25
```

```
###########################
#Panic Disorder/Agoraphobia#
###########################
prevalence_1834 <- as.numeric(Prevalence_estimates[Prevalence_estimates$Diagnosis == "Panic disorder/Agoraphobia", "1834y"])
prevalence_3549 <- as.numeric(Prevalence_estimates[Prevalence_estimates$Diagnosis == "Panic disorder/Agoraphobia", "3549y"])
prevalence_5064 <- as.numeric(Prevalence_estimates[Prevalence_estimates$Diagnosis == "Panic disorder/Agoraphobia", "5064y"])
prevalence_6574 <- as.numeric(Prevalence_estimates[Prevalence_estimates$Diagnosis == "Panic disorder/Agoraphobia", "6574y"])
prevalence_75plus <- as.numeric(Prevalence_estimates[Prevalence_estimates$Diagnosis == "Panic disorder/Agoraphobia", "75yplus"])

expected_ratio_1834 <- (census_amount_1834*prevalence_1834)/((census_amount_1834*prevalence_1834)+(census_amount_3549*prevalence_3549)+(census_amount_5064*prevalence_5064)+(census_amount_6574*prevalence_6574)+(census_amount_75plus*prevalence_75plus))
expected_ratio_3549 <- (census_amount_3549*prevalence_3549)/((census_amount_1834*prevalence_1834)+(census_amount_3549*prevalence_3549)+(census_amount_5064*prevalence_5064)+(census_amount_6574*prevalence_6574)+(census_amount_75plus*prevalence_75plus))
expected_ratio_5064 <- (census_amount_5064*prevalence_5064)/((census_amount_1834*prevalence_1834)+(census_amount_3549*prevalence_3549)+(census_amount_5064*prevalence_5064)+(census_amount_6574*prevalence_6574)+(census_amount_75plus*prevalence_75plus))
expected_ratio_6574 <- (census_amount_6574*prevalence_6574)/((census_amount_1834*prevalence_1834)+(census_amount_3549*prevalence_3549)+(census_amount_5064*prevalence_5064)+(census_amount_6574*prevalence_6574)+(census_amount_75plus*prevalence_75plus))
expected_ratio_75plus <- (census_amount_75plus*prevalence_75plus)/((census_amount_1834*prevalence_1834)+(census_amount_3549*prevalence_3549)+(census_amount_5064*prevalence_5064)+(census_amount_6574*prevalence_6574)+(census_amount_75plus*prevalence_75plus))

round(c(expected_ratio_1834,expected_ratio_3549,expected_ratio_5064,expected_ratio_6574,expected_ratio_75plus), 2)
```

```
## [1] 0.25 0.23 0.28 0.11 0.12
```

```
KODAP_data_complete_PanicAgora <- KODAP_data_complete[KODAP_data_complete$ICD1_pre_recode %in% c("F40.0X F41.0 Agoraphobie/Panikstörung") |
                                                              KODAP_data_complete$ICD2_pre_recode %in% c("F40.0X F41.0 Agoraphobie/Panikstörung") |
                                                              KODAP_data_complete$ICD3_pre_recode %in% c("F40.0X F41.0 Agoraphobie/Panikstörung") |
                                                              KODAP_data_complete$ICD4_pre_recode %in% c("F40.0X F41.0 Agoraphobie/Panikstörung") |
                                                              KODAP_data_complete$ICD5_pre_recode %in% c("F40.0X F41.0 Agoraphobie/Panikstörung"),]

table(KODAP_data_complete_PanicAgora$Age_Stepped_2)
```

```
## 
##   0   1   2   3   4 
## 731 370 299  47   9
```

```
round(table(KODAP_data_complete_PanicAgora$Age_Stepped_2)/length(KODAP_data_complete_PanicAgora$Age_Stepped_2)*100, 1)
```

```
## 
##    0    1    2    3    4 
## 50.2 25.4 20.5  3.2  0.6
```

```
#18-34 years
prevalence_1834 <- as.numeric(Prevalence_estimates[Prevalence_estimates$Diagnosis == "Panic disorder/Agoraphobia", "1834y"])
prevalence_3549 <- as.numeric(Prevalence_estimates[Prevalence_estimates$Diagnosis == "Panic disorder/Agoraphobia", "3549y"])
prevalence_5064 <- as.numeric(Prevalence_estimates[Prevalence_estimates$Diagnosis == "Panic disorder/Agoraphobia", "5064y"])

prevalence_1864 <- prevalence_1834

prevalence_6574 <- as.numeric(Prevalence_estimates[Prevalence_estimates$Diagnosis == "Panic disorder/Agoraphobia", "6574y"])
prevalence_75plus <- as.numeric(Prevalence_estimates[Prevalence_estimates$Diagnosis == "Panic disorder/Agoraphobia", "75yplus"])

#Ratios in reference population
expected_ratio_1864 <- (census_amount_1834*prevalence_1864)/((census_amount_1834*prevalence_1864)+(census_amount_6574*prevalence_6574)+(census_amount_75plus*prevalence_75plus))
expected_ratio_6574 <- (census_amount_6574*prevalence_6574)/((census_amount_1834*prevalence_1864)+(census_amount_6574*prevalence_6574)+(census_amount_75plus*prevalence_75plus))
expected_ratio_75plus <- (census_amount_75plus*prevalence_75plus)/((census_amount_1834*prevalence_1864)+(census_amount_6574*prevalence_6574)+(census_amount_75plus*prevalence_75plus))

expected_ratios <- c(expected_ratio_1864,
                     expected_ratio_6574,
                     expected_ratio_75plus)
round(expected_ratios,2)
```

```
## [1] 0.52 0.23 0.24
```

```
KODAP_data_complete_PanicAgora <- KODAP_data_complete_s1[KODAP_data_complete_s1$ICD1_pre_recode %in% c("F40.0X F41.0 Agoraphobie/Panikstörung") |
                                                                 KODAP_data_complete_s1$ICD2_pre_recode %in% c("F40.0X F41.0 Agoraphobie/Panikstörung") |
                                                                 KODAP_data_complete_s1$ICD3_pre_recode %in% c("F40.0X F41.0 Agoraphobie/Panikstörung") |
                                                                 KODAP_data_complete_s1$ICD4_pre_recode %in% c("F40.0X F41.0 Agoraphobie/Panikstörung") |
                                                                 KODAP_data_complete_s1$ICD5_pre_recode %in% c("F40.0X F41.0 Agoraphobie/Panikstörung"),]

length(KODAP_data_complete_PanicAgora$Patient_ID)
```

```
## [1] 787
```

```
#Observed vs. expected: 
Observation <- as.vector(table(KODAP_data_complete_PanicAgora$Age_Stepped))
Expected <- expected_ratios*length(KODAP_data_complete_PanicAgora$Patient_ID)

Observation
```

```
## [1] 731  47   9
```

```
round(Observation/length(KODAP_data_complete_PanicAgora$Patient_ID)*100,1)
```

```
## [1] 92.9  6.0  1.1
```

```
# Chi-squared test for given probabilities
test <- chisq.test(Observation, p = expected_ratios)
test
```

```
## 
##  Chi-squared test for given probabilities
## 
## data:  Observation
## X-squared = 527.79, df = 2, p-value < 2.2e-16
```

```
# Post-hoc binomial-test for each category with Bonferroni-correction
binom.test(Observation[1], sum(Observation), expected_ratios[1])$p.value*3
```

```
## [1] 2.347813e-138
```

```
binom.test(Observation[2], sum(Observation), expected_ratios[2])$p.value*3
```

```
## [1] 4.350377e-39
```

```
binom.test(Observation[3], sum(Observation), expected_ratios[3])$p.value*3
```

```
## [1] 7.678958e-80
```

```
#Estimating underrepresentation compared to reference population
#Representation quotients
Observation/Expected
```

```
## [1] 1.78164136 0.25516409 0.04675107
```

```
#Confidence-intervals of Representation quotients
#Confidence-intervals around sample proportion
prop.test(x = Observation[2], n = sum(Observation), conf.level = .95)
```

```
## 
##  1-sample proportions test with continuity correction
## 
## data:  Observation[2] out of sum(Observation), null probability 0.5
## X-squared = 608.47, df = 1, p-value < 2.2e-16
## alternative hypothesis: true p is not equal to 0.5
## 95 percent confidence interval:
##  0.04465484 0.07922572
## sample estimates:
##          p 
## 0.05972046
```

```
#Confidence-intervals around sample proportion relative to expected proportion
round(prop.test(x = Observation[2], n = sum(Observation), conf.level = .95)$estimate/expected_ratio_6574,2)
```

```
##    p 
## 0.26
```

```
round(as.vector(prop.test(x = Observation[2], n = sum(Observation), conf.level = .95)$conf.int)/expected_ratio_6574,2)
```

```
## [1] 0.19 0.34
```

```
#Confidence-intervals around sample proportion
prop.test(x = Observation[3], n = sum(Observation), conf.level = .95)
```

```
## 
##  1-sample proportions test with continuity correction
## 
## data:  Observation[3] out of sum(Observation), null probability 0.5
## X-squared = 749.46, df = 1, p-value < 2.2e-16
## alternative hypothesis: true p is not equal to 0.5
## 95 percent confidence interval:
##  0.005591072 0.022412707
## sample estimates:
##          p 
## 0.01143583
```

```
#Confidence-intervals around sample proportion relative to expected proportion
round(prop.test(x = Observation[3], n = sum(Observation), conf.level = .95)$estimate/expected_ratio_75plus,2)
```

```
##    p 
## 0.05
```

```
round(as.vector(prop.test(x = Observation[3], n = sum(Observation), conf.level = .95)$conf.int)/expected_ratio_75plus,2)
```

```
## [1] 0.02 0.09
```

```
#35-49 years
prevalence_1834 <- as.numeric(Prevalence_estimates[Prevalence_estimates$Diagnosis == "Panic disorder/Agoraphobia", "1834y"])
prevalence_3549 <- as.numeric(Prevalence_estimates[Prevalence_estimates$Diagnosis == "Panic disorder/Agoraphobia", "3549y"])
prevalence_5064 <- as.numeric(Prevalence_estimates[Prevalence_estimates$Diagnosis == "Panic disorder/Agoraphobia", "5064y"])

prevalence_1864 <- prevalence_3549

prevalence_6574 <- as.numeric(Prevalence_estimates[Prevalence_estimates$Diagnosis == "Panic disorder/Agoraphobia", "6574y"])
prevalence_75plus <- as.numeric(Prevalence_estimates[Prevalence_estimates$Diagnosis == "Panic disorder/Agoraphobia", "75yplus"])

#Ratios in reference population
expected_ratio_1864 <- (census_amount_3549*prevalence_1864)/((census_amount_3549*prevalence_1864)+(census_amount_6574*prevalence_6574)+(census_amount_75plus*prevalence_75plus))
expected_ratio_6574 <- (census_amount_6574*prevalence_6574)/((census_amount_3549*prevalence_1864)+(census_amount_6574*prevalence_6574)+(census_amount_75plus*prevalence_75plus))
expected_ratio_75plus <- (census_amount_75plus*prevalence_75plus)/((census_amount_3549*prevalence_1864)+(census_amount_6574*prevalence_6574)+(census_amount_75plus*prevalence_75plus))

expected_ratios <- c(expected_ratio_1864,
                     expected_ratio_6574,
                     expected_ratio_75plus)
round(expected_ratios,2)
```

```
## [1] 0.50 0.25 0.26
```

```
KODAP_data_complete_PanicAgora <- KODAP_data_complete_s2[KODAP_data_complete_s2$ICD1_pre_recode %in% c("F40.0X F41.0 Agoraphobie/Panikstörung") |
                                                                 KODAP_data_complete_s2$ICD2_pre_recode %in% c("F40.0X F41.0 Agoraphobie/Panikstörung") |
                                                                 KODAP_data_complete_s2$ICD3_pre_recode %in% c("F40.0X F41.0 Agoraphobie/Panikstörung") |
                                                                 KODAP_data_complete_s2$ICD4_pre_recode %in% c("F40.0X F41.0 Agoraphobie/Panikstörung") |
                                                                 KODAP_data_complete_s2$ICD5_pre_recode %in% c("F40.0X F41.0 Agoraphobie/Panikstörung"),]

length(KODAP_data_complete_PanicAgora$Patient_ID)
```

```
## [1] 426
```

```
#Observed vs. expected: 
Observation <- as.vector(table(KODAP_data_complete_PanicAgora$Age_Stepped))
Expected <- expected_ratios*length(KODAP_data_complete_PanicAgora$Patient_ID)

Observation
```

```
## [1] 370  47   9
```

```
round(Observation/length(KODAP_data_complete_PanicAgora$Patient_ID)*100,1)
```

```
## [1] 86.9 11.0  2.1
```

```
# Chi-squared test for given probabilities
test <- chisq.test(Observation, p = expected_ratios)
test
```

```
## 
##  Chi-squared test for given probabilities
## 
## data:  Observation
## X-squared = 241, df = 2, p-value < 2.2e-16
```

```
# Post-hoc binomial-test for each category with Bonferroni-correction
binom.test(Observation[1], sum(Observation), expected_ratios[1])$p.value*3
```

```
## [1] 4.383763e-58
```

```
binom.test(Observation[2], sum(Observation), expected_ratios[2])$p.value*3
```

```
## [1] 7.7275e-12
```

```
binom.test(Observation[3], sum(Observation), expected_ratios[3])$p.value*3
```

```
## [1] 7.870622e-41
```

```
#Estimating underrepresentation compared to reference population
#Representation quotients
Observation/Expected
```

```
## [1] 1.74360092 0.44959501 0.08237462
```

```
#Confidence-intervals of Representation quotients
#Confidence-intervals around sample proportion
prop.test(x = Observation[2], n = sum(Observation), conf.level = .95)
```

```
## 
##  1-sample proportions test with continuity correction
## 
## data:  Observation[2] out of sum(Observation), null probability 0.5
## X-squared = 257.19, df = 1, p-value < 2.2e-16
## alternative hypothesis: true p is not equal to 0.5
## 95 percent confidence interval:
##  0.0829624 0.1449315
## sample estimates:
##         p 
## 0.1103286
```

```
#Confidence-intervals around sample proportion relative to expected proportion
round(prop.test(x = Observation[2], n = sum(Observation), conf.level = .95)$estimate/expected_ratio_6574,2)
```

```
##    p 
## 0.45
```

```
round(as.vector(prop.test(x = Observation[2], n = sum(Observation), conf.level = .95)$conf.int)/expected_ratio_6574,2)
```

```
## [1] 0.34 0.59
```

```
#Confidence-intervals around sample proportion
prop.test(x = Observation[3], n = sum(Observation), conf.level = .95)
```

```
## 
##  1-sample proportions test with continuity correction
## 
## data:  Observation[3] out of sum(Observation), null probability 0.5
## X-squared = 388.85, df = 1, p-value < 2.2e-16
## alternative hypothesis: true p is not equal to 0.5
## 95 percent confidence interval:
##  0.01034477 0.04116685
## sample estimates:
##          p 
## 0.02112676
```

```
#Confidence-intervals around sample proportion relative to expected proportion
round(prop.test(x = Observation[3], n = sum(Observation), conf.level = .95)$estimate/expected_ratio_75plus,2)
```

```
##    p 
## 0.08
```

```
round(as.vector(prop.test(x = Observation[3], n = sum(Observation), conf.level = .95)$conf.int)/expected_ratio_75plus,2)
```

```
## [1] 0.04 0.16
```

```
#50-64 years
prevalence_1834 <- as.numeric(Prevalence_estimates[Prevalence_estimates$Diagnosis == "Panic disorder/Agoraphobia", "1834y"])
prevalence_3549 <- as.numeric(Prevalence_estimates[Prevalence_estimates$Diagnosis == "Panic disorder/Agoraphobia", "3549y"])
prevalence_5064 <- as.numeric(Prevalence_estimates[Prevalence_estimates$Diagnosis == "Panic disorder/Agoraphobia", "5064y"])

prevalence_1864 <- prevalence_5064

prevalence_6574 <- as.numeric(Prevalence_estimates[Prevalence_estimates$Diagnosis == "Panic disorder/Agoraphobia", "6574y"])
prevalence_75plus <- as.numeric(Prevalence_estimates[Prevalence_estimates$Diagnosis == "Panic disorder/Agoraphobia", "75yplus"])

#Ratios in reference population
expected_ratio_1864 <- (census_amount_5064*prevalence_1864)/((census_amount_5064*prevalence_1864)+(census_amount_6574*prevalence_6574)+(census_amount_75plus*prevalence_75plus))
expected_ratio_6574 <- (census_amount_6574*prevalence_6574)/((census_amount_5064*prevalence_1864)+(census_amount_6574*prevalence_6574)+(census_amount_75plus*prevalence_75plus))
expected_ratio_75plus <- (census_amount_75plus*prevalence_75plus)/((census_amount_5064*prevalence_1864)+(census_amount_6574*prevalence_6574)+(census_amount_75plus*prevalence_75plus))

expected_ratios <- c(expected_ratio_1864,
                     expected_ratio_6574,
                     expected_ratio_75plus)
round(expected_ratios,2)
```

```
## [1] 0.55 0.22 0.23
```

```
KODAP_data_complete_PanicAgora <- KODAP_data_complete_s3[KODAP_data_complete_s3$ICD1_pre_recode %in% c("F40.0X F41.0 Agoraphobie/Panikstörung") |
                                                                 KODAP_data_complete_s3$ICD2_pre_recode %in% c("F40.0X F41.0 Agoraphobie/Panikstörung") |
                                                                 KODAP_data_complete_s3$ICD3_pre_recode %in% c("F40.0X F41.0 Agoraphobie/Panikstörung") |
                                                                 KODAP_data_complete_s3$ICD4_pre_recode %in% c("F40.0X F41.0 Agoraphobie/Panikstörung") |
                                                                 KODAP_data_complete_s3$ICD5_pre_recode %in% c("F40.0X F41.0 Agoraphobie/Panikstörung"),]

length(KODAP_data_complete_PanicAgora$Patient_ID)
```

```
## [1] 355
```

```
#Observed vs. expected: 
Observation <- as.vector(table(KODAP_data_complete_PanicAgora$Age_Stepped))
Expected <- expected_ratios*length(KODAP_data_complete_PanicAgora$Patient_ID)

Observation
```

```
## [1] 299  47   9
```

```
round(Observation/length(KODAP_data_complete_PanicAgora$Patient_ID)*100,1)
```

```
## [1] 84.2 13.2  2.5
```

```
# Chi-squared test for given probabilities
test <- chisq.test(Observation, p = expected_ratios)
test
```

```
## 
##  Chi-squared test for given probabilities
## 
## data:  Observation
## X-squared = 133.34, df = 2, p-value < 2.2e-16
```

```
# Post-hoc binomial-test for each category with Bonferroni-correction
binom.test(Observation[1], sum(Observation), expected_ratios[1])$p.value*3
```

```
## [1] 2.252067e-31
```

```
binom.test(Observation[2], sum(Observation), expected_ratios[2])$p.value*3
```

```
## [1] 8.755743e-05
```

```
binom.test(Observation[3], sum(Observation), expected_ratios[3])$p.value*3
```

```
## [1] 9.071155e-28
```

```
#Estimating underrepresentation compared to reference population
#Representation quotients
Observation/Expected
```

```
## [1] 1.5356883 0.5996379 0.1098654
```

```
#Confidence-intervals of Representation quotients
#Confidence-intervals around sample proportion
prop.test(x = Observation[2], n = sum(Observation), conf.level = .95)
```

```
## 
##  1-sample proportions test with continuity correction
## 
## data:  Observation[2] out of sum(Observation), null probability 0.5
## X-squared = 190.42, df = 1, p-value < 2.2e-16
## alternative hypothesis: true p is not equal to 0.5
## 95 percent confidence interval:
##  0.09980566 0.17316442
## sample estimates:
##         p 
## 0.1323944
```

```
#Confidence-intervals around sample proportion relative to expected proportion
round(prop.test(x = Observation[2], n = sum(Observation), conf.level = .95)$estimate/expected_ratio_6574,2)
```

```
##   p 
## 0.6
```

```
round(as.vector(prop.test(x = Observation[2], n = sum(Observation), conf.level = .95)$conf.int)/expected_ratio_6574,2)
```

```
## [1] 0.45 0.78
```

```
#Confidence-intervals around sample proportion
prop.test(x = Observation[3], n = sum(Observation), conf.level = .95)
```

```
## 
##  1-sample proportions test with continuity correction
## 
## data:  Observation[3] out of sum(Observation), null probability 0.5
## X-squared = 318.02, df = 1, p-value < 2.2e-16
## alternative hypothesis: true p is not equal to 0.5
## 95 percent confidence interval:
##  0.0124220 0.0492759
## sample estimates:
##          p 
## 0.02535211
```

```
#Confidence-intervals around sample proportion relative to expected proportion
round(prop.test(x = Observation[3], n = sum(Observation), conf.level = .95)$estimate/expected_ratio_75plus,2)
```

```
##    p 
## 0.11
```

```
round(as.vector(prop.test(x = Observation[3], n = sum(Observation), conf.level = .95)$conf.int)/expected_ratio_75plus,2)
```

```
## [1] 0.05 0.21
```

```
###########################
#Social phobia#
###########################
prevalence_1834 <- as.numeric(Prevalence_estimates[Prevalence_estimates$Diagnosis == "Social phobia", "1834y"])
prevalence_3549 <- as.numeric(Prevalence_estimates[Prevalence_estimates$Diagnosis == "Social phobia", "3549y"])
prevalence_5064 <- as.numeric(Prevalence_estimates[Prevalence_estimates$Diagnosis == "Social phobia", "5064y"])
prevalence_6574 <- as.numeric(Prevalence_estimates[Prevalence_estimates$Diagnosis == "Social phobia", "6574y"])
prevalence_75plus <- as.numeric(Prevalence_estimates[Prevalence_estimates$Diagnosis == "Social phobia", "75yplus"])

expected_ratio_1834 <- (census_amount_1834*prevalence_1834)/((census_amount_1834*prevalence_1834)+(census_amount_3549*prevalence_3549)+(census_amount_5064*prevalence_5064)+(census_amount_6574*prevalence_6574)+(census_amount_75plus*prevalence_75plus))
expected_ratio_3549 <- (census_amount_3549*prevalence_3549)/((census_amount_1834*prevalence_1834)+(census_amount_3549*prevalence_3549)+(census_amount_5064*prevalence_5064)+(census_amount_6574*prevalence_6574)+(census_amount_75plus*prevalence_75plus))
expected_ratio_5064 <- (census_amount_5064*prevalence_5064)/((census_amount_1834*prevalence_1834)+(census_amount_3549*prevalence_3549)+(census_amount_5064*prevalence_5064)+(census_amount_6574*prevalence_6574)+(census_amount_75plus*prevalence_75plus))
expected_ratio_6574 <- (census_amount_6574*prevalence_6574)/((census_amount_1834*prevalence_1834)+(census_amount_3549*prevalence_3549)+(census_amount_5064*prevalence_5064)+(census_amount_6574*prevalence_6574)+(census_amount_75plus*prevalence_75plus))
expected_ratio_75plus <- (census_amount_75plus*prevalence_75plus)/((census_amount_1834*prevalence_1834)+(census_amount_3549*prevalence_3549)+(census_amount_5064*prevalence_5064)+(census_amount_6574*prevalence_6574)+(census_amount_75plus*prevalence_75plus))

round(c(expected_ratio_1834,expected_ratio_3549,expected_ratio_5064,expected_ratio_6574,expected_ratio_75plus), 2)
```

```
## [1] 0.43 0.27 0.23 0.03 0.04
```

```
KODAP_data_complete_socialphobia <- KODAP_data_complete[KODAP_data_complete$ICD1_pre_recode %in% c("F40.1 Soziale Phobie") |
                                                              KODAP_data_complete$ICD2_pre_recode %in% c("F40.1 Soziale Phobie") |
                                                              KODAP_data_complete$ICD3_pre_recode %in% c("F40.1 Soziale Phobie") |
                                                              KODAP_data_complete$ICD4_pre_recode %in% c("F40.1 Soziale Phobie") |
                                                              KODAP_data_complete$ICD5_pre_recode %in% c("F40.1 Soziale Phobie"),]

table(KODAP_data_complete_socialphobia$Age_Stepped_2)
```

```
## 
##    0    1    2    3    4 
## 1407  295  128   16    4
```

```
round(table(KODAP_data_complete_socialphobia$Age_Stepped_2)/length(KODAP_data_complete_socialphobia$Age_Stepped_2)*100, 1)
```

```
## 
##    0    1    2    3    4 
## 76.1 15.9  6.9  0.9  0.2
```

```
#18-34 years
prevalence_1834 <- as.numeric(Prevalence_estimates[Prevalence_estimates$Diagnosis == "Social phobia", "1834y"])
prevalence_3549 <- as.numeric(Prevalence_estimates[Prevalence_estimates$Diagnosis == "Social phobia", "3549y"])
prevalence_5064 <- as.numeric(Prevalence_estimates[Prevalence_estimates$Diagnosis == "Social phobia", "5064y"])

prevalence_1864 <- prevalence_1834

prevalence_6574 <- as.numeric(Prevalence_estimates[Prevalence_estimates$Diagnosis == "Social phobia", "6574y"])
prevalence_75plus <- as.numeric(Prevalence_estimates[Prevalence_estimates$Diagnosis == "Social phobia", "75yplus"])

#Ratios in reference population
expected_ratio_1864 <- (census_amount_1834*prevalence_1864)/((census_amount_1834*prevalence_1864)+(census_amount_6574*prevalence_6574)+(census_amount_75plus*prevalence_75plus))
expected_ratio_6574 <- (census_amount_6574*prevalence_6574)/((census_amount_1834*prevalence_1864)+(census_amount_6574*prevalence_6574)+(census_amount_75plus*prevalence_75plus))
expected_ratio_75plus <- (census_amount_75plus*prevalence_75plus)/((census_amount_1834*prevalence_1864)+(census_amount_6574*prevalence_6574)+(census_amount_75plus*prevalence_75plus))

expected_ratios <- c(expected_ratio_1864,
                     expected_ratio_6574,
                     expected_ratio_75plus)
round(expected_ratios,2)
```

```
## [1] 0.86 0.07 0.07
```

```
KODAP_data_complete_socialphobia <- KODAP_data_complete_s1[KODAP_data_complete_s1$ICD1_pre_recode %in% c("F40.1 Soziale Phobie") |
                                                           KODAP_data_complete_s1$ICD2_pre_recode %in% c("F40.1 Soziale Phobie") |
                                                           KODAP_data_complete_s1$ICD3_pre_recode %in% c("F40.1 Soziale Phobie") |
                                                           KODAP_data_complete_s1$ICD4_pre_recode %in% c("F40.1 Soziale Phobie") |
                                                           KODAP_data_complete_s1$ICD5_pre_recode %in% c("F40.1 Soziale Phobie"),]

length(KODAP_data_complete_socialphobia$Patient_ID)
```

```
## [1] 1427
```

```
#Observed vs. expected: 
Observation <- as.vector(table(KODAP_data_complete_socialphobia$Age_Stepped))
Expected <- expected_ratios*length(KODAP_data_complete_socialphobia$Patient_ID)

Observation
```

```
## [1] 1407   16    4
```

```
round(Observation/length(KODAP_data_complete_socialphobia$Patient_ID)*100,1)
```

```
## [1] 98.6  1.1  0.3
```

```
# Chi-squared test for given probabilities
test <- chisq.test(Observation, p = expected_ratios)
test
```

```
## 
##  Chi-squared test for given probabilities
## 
## data:  Observation
## X-squared = 195.58, df = 2, p-value < 2.2e-16
```

```
# Post-hoc binomial-test for each category with Bonferroni-correction
binom.test(Observation[1], sum(Observation), expected_ratios[1])$p.value*3
```

```
## [1] 7.003197e-67
```

```
binom.test(Observation[2], sum(Observation), expected_ratios[2])$p.value*3
```

```
## [1] 6.415478e-26
```

```
binom.test(Observation[3], sum(Observation), expected_ratios[3])$p.value*3
```

```
## [1] 2.729888e-40
```

```
#Estimating underrepresentation compared to reference population
#Representation quotients
Observation/Expected
```

```
## [1] 1.15129299 0.15970153 0.03820115
```

```
#Confidence-intervals of Representation quotients
#Confidence-intervals around sample proportion
prop.test(x = Observation[2], n = sum(Observation), conf.level = .95)
```

```
## 
##  1-sample proportions test with continuity correction
## 
## data:  Observation[2] out of sum(Observation), null probability 0.5
## X-squared = 1361.8, df = 1, p-value < 2.2e-16
## alternative hypothesis: true p is not equal to 0.5
## 95 percent confidence interval:
##  0.006646199 0.018566480
## sample estimates:
##          p 
## 0.01121233
```

```
#Confidence-intervals around sample proportion relative to expected proportion
round(prop.test(x = Observation[2], n = sum(Observation), conf.level = .95)$estimate/expected_ratio_6574,2)
```

```
##    p 
## 0.16
```

```
round(as.vector(prop.test(x = Observation[2], n = sum(Observation), conf.level = .95)$conf.int)/expected_ratio_6574,2)
```

```
## [1] 0.09 0.26
```

```
#Confidence-intervals around sample proportion
prop.test(x = Observation[3], n = sum(Observation), conf.level = .95)
```

```
## 
##  1-sample proportions test with continuity correction
## 
## data:  Observation[3] out of sum(Observation), null probability 0.5
## X-squared = 1409.1, df = 1, p-value < 2.2e-16
## alternative hypothesis: true p is not equal to 0.5
## 95 percent confidence interval:
##  0.0008983203 0.0076840834
## sample estimates:
##           p 
## 0.002803083
```

```
#Confidence-intervals around sample proportion relative to expected proportion
round(prop.test(x = Observation[3], n = sum(Observation), conf.level = .95)$estimate/expected_ratio_75plus,2)
```

```
##    p 
## 0.04
```

```
round(as.vector(prop.test(x = Observation[3], n = sum(Observation), conf.level = .95)$conf.int)/expected_ratio_75plus,2)
```

```
## [1] 0.01 0.10
```

```
#35-49 years
prevalence_1834 <- as.numeric(Prevalence_estimates[Prevalence_estimates$Diagnosis == "Social phobia", "1834y"])
prevalence_3549 <- as.numeric(Prevalence_estimates[Prevalence_estimates$Diagnosis == "Social phobia", "3549y"])
prevalence_5064 <- as.numeric(Prevalence_estimates[Prevalence_estimates$Diagnosis == "Social phobia", "5064y"])

prevalence_1864 <- prevalence_3549

prevalence_6574 <- as.numeric(Prevalence_estimates[Prevalence_estimates$Diagnosis == "Social phobia", "6574y"])
prevalence_75plus <- as.numeric(Prevalence_estimates[Prevalence_estimates$Diagnosis == "Social phobia", "75yplus"])

#Ratios in reference population
expected_ratio_1864 <- (census_amount_3549*prevalence_1864)/((census_amount_3549*prevalence_1864)+(census_amount_6574*prevalence_6574)+(census_amount_75plus*prevalence_75plus))
expected_ratio_6574 <- (census_amount_6574*prevalence_6574)/((census_amount_3549*prevalence_1864)+(census_amount_6574*prevalence_6574)+(census_amount_75plus*prevalence_75plus))
expected_ratio_75plus <- (census_amount_75plus*prevalence_75plus)/((census_amount_3549*prevalence_1864)+(census_amount_6574*prevalence_6574)+(census_amount_75plus*prevalence_75plus))

expected_ratios <- c(expected_ratio_1864,
                     expected_ratio_6574,
                     expected_ratio_75plus)
round(expected_ratios,2)
```

```
## [1] 0.79 0.10 0.11
```

```
KODAP_data_complete_socialphobia <- KODAP_data_complete_s2[KODAP_data_complete_s2$ICD1_pre_recode %in% c("F40.1 Soziale Phobie") |
                                                           KODAP_data_complete_s2$ICD2_pre_recode %in% c("F40.1 Soziale Phobie") |
                                                           KODAP_data_complete_s2$ICD3_pre_recode %in% c("F40.1 Soziale Phobie") |
                                                           KODAP_data_complete_s2$ICD4_pre_recode %in% c("F40.1 Soziale Phobie") |
                                                           KODAP_data_complete_s2$ICD5_pre_recode %in% c("F40.1 Soziale Phobie"),]

length(KODAP_data_complete_socialphobia$Patient_ID)
```

```
## [1] 315
```

```
#Observed vs. expected: 
Observation <- as.vector(table(KODAP_data_complete_socialphobia$Age_Stepped))
Expected <- expected_ratios*length(KODAP_data_complete_socialphobia$Patient_ID)

Observation
```

```
## [1] 295  16   4
```

```
round(Observation/length(KODAP_data_complete_socialphobia$Patient_ID)*100,1)
```

```
## [1] 93.7  5.1  1.3
```

```
# Chi-squared test for given probabilities
test <- chisq.test(Observation, p = expected_ratios)
test
```

```
## 
##  Chi-squared test for given probabilities
## 
## data:  Observation
## X-squared = 43.267, df = 2, p-value = 4.025e-10
```

```
# Post-hoc binomial-test for each category with Bonferroni-correction
binom.test(Observation[1], sum(Observation), expected_ratios[1])$p.value*3
```

```
## [1] 2.120926e-12
```

```
binom.test(Observation[2], sum(Observation), expected_ratios[2])$p.value*3
```

```
## [1] 0.004490586
```

```
binom.test(Observation[3], sum(Observation), expected_ratios[3])$p.value*3
```

```
## [1] 1.774582e-10
```

```
#Estimating underrepresentation compared to reference population
#Representation quotients
Observation/Expected
```

```
## [1] 1.1860866 0.4936742 0.1180885
```

```
#Confidence-intervals of Representation quotients
#Confidence-intervals around sample proportion
prop.test(x = Observation[2], n = sum(Observation), conf.level = .95)
```

```
## 
##  1-sample proportions test with continuity correction
## 
## data:  Observation[2] out of sum(Observation), null probability 0.5
## X-squared = 252.46, df = 1, p-value < 2.2e-16
## alternative hypothesis: true p is not equal to 0.5
## 95 percent confidence interval:
##  0.03028244 0.08281635
## sample estimates:
##          p 
## 0.05079365
```

```
#Confidence-intervals around sample proportion relative to expected proportion
round(prop.test(x = Observation[2], n = sum(Observation), conf.level = .95)$estimate/expected_ratio_6574,2)
```

```
##    p 
## 0.49
```

```
round(as.vector(prop.test(x = Observation[2], n = sum(Observation), conf.level = .95)$conf.int)/expected_ratio_6574,2)
```

```
## [1] 0.29 0.80
```

```
#Confidence-intervals around sample proportion
prop.test(x = Observation[3], n = sum(Observation), conf.level = .95)
```

```
## 
##  1-sample proportions test with continuity correction
## 
## data:  Observation[3] out of sum(Observation), null probability 0.5
## X-squared = 297.26, df = 1, p-value < 2.2e-16
## alternative hypothesis: true p is not equal to 0.5
## 95 percent confidence interval:
##  0.004075547 0.034417169
## sample estimates:
##          p 
## 0.01269841
```

```
#Confidence-intervals around sample proportion relative to expected proportion
round(prop.test(x = Observation[3], n = sum(Observation), conf.level = .95)$estimate/expected_ratio_75plus,2)
```

```
##    p 
## 0.12
```

```
round(as.vector(prop.test(x = Observation[3], n = sum(Observation), conf.level = .95)$conf.int)/expected_ratio_75plus,2)
```

```
## [1] 0.04 0.32
```

```
#50-64 years
prevalence_1834 <- as.numeric(Prevalence_estimates[Prevalence_estimates$Diagnosis == "Social phobia", "1834y"])
prevalence_3549 <- as.numeric(Prevalence_estimates[Prevalence_estimates$Diagnosis == "Social phobia", "3549y"])
prevalence_5064 <- as.numeric(Prevalence_estimates[Prevalence_estimates$Diagnosis == "Social phobia", "5064y"])

prevalence_1864 <- prevalence_5064

prevalence_6574 <- as.numeric(Prevalence_estimates[Prevalence_estimates$Diagnosis == "Social phobia", "6574y"])
prevalence_75plus <- as.numeric(Prevalence_estimates[Prevalence_estimates$Diagnosis == "Social phobia", "75yplus"])

#Ratios in reference population
expected_ratio_1864 <- (census_amount_5064*prevalence_1864)/((census_amount_5064*prevalence_1864)+(census_amount_6574*prevalence_6574)+(census_amount_75plus*prevalence_75plus))
expected_ratio_6574 <- (census_amount_6574*prevalence_6574)/((census_amount_5064*prevalence_1864)+(census_amount_6574*prevalence_6574)+(census_amount_75plus*prevalence_75plus))
expected_ratio_75plus <- (census_amount_75plus*prevalence_75plus)/((census_amount_5064*prevalence_1864)+(census_amount_6574*prevalence_6574)+(census_amount_75plus*prevalence_75plus))

expected_ratios <- c(expected_ratio_1864,
                     expected_ratio_6574,
                     expected_ratio_75plus)
round(expected_ratios,2)
```

```
## [1] 0.77 0.11 0.12
```

```
KODAP_data_complete_socialphobia <- KODAP_data_complete_s3[KODAP_data_complete_s3$ICD1_pre_recode %in% c("F40.1 Soziale Phobie") |
                                                           KODAP_data_complete_s3$ICD2_pre_recode %in% c("F40.1 Soziale Phobie") |
                                                           KODAP_data_complete_s3$ICD3_pre_recode %in% c("F40.1 Soziale Phobie") |
                                                           KODAP_data_complete_s3$ICD4_pre_recode %in% c("F40.1 Soziale Phobie") |
                                                           KODAP_data_complete_s3$ICD5_pre_recode %in% c("F40.1 Soziale Phobie"),]

length(KODAP_data_complete_socialphobia$Patient_ID)
```

```
## [1] 148
```

```
#Observed vs. expected: 
Observation <- as.vector(table(KODAP_data_complete_socialphobia$Age_Stepped))
Expected <- expected_ratios*length(KODAP_data_complete_socialphobia$Patient_ID)

Observation
```

```
## [1] 128  16   4
```

```
round(Observation/length(KODAP_data_complete_socialphobia$Patient_ID)*100,1)
```

```
## [1] 86.5 10.8  2.7
```

```
# Chi-squared test for given probabilities
test <- chisq.test(Observation, p = expected_ratios)
test
```

```
## 
##  Chi-squared test for given probabilities
## 
## data:  Observation
## X-squared = 12.64, df = 2, p-value = 0.0018
```

```
# Post-hoc binomial-test for each category with Bonferroni-correction
binom.test(Observation[1], sum(Observation), expected_ratios[1])$p.value*3
```

```
## [1] 0.01035804
```

```
binom.test(Observation[2], sum(Observation), expected_ratios[2])$p.value*3
```

```
## [1] 2.692998
```

```
binom.test(Observation[3], sum(Observation), expected_ratios[3])$p.value*3
```

```
## [1] 0.0003292119
```

```
#Estimating underrepresentation compared to reference population
#Representation quotients
Observation/Expected
```

```
## [1] 1.1302657 0.9415832 0.2252299
```

```
#Confidence-intervals of Representation quotients
#Confidence-intervals around sample proportion
prop.test(x = Observation[2], n = sum(Observation), conf.level = .95)
```

```
## 
##  1-sample proportions test with continuity correction
## 
## data:  Observation[2] out of sum(Observation), null probability 0.5
## X-squared = 89.358, df = 1, p-value < 2.2e-16
## alternative hypothesis: true p is not equal to 0.5
## 95 percent confidence interval:
##  0.06500968 0.17233195
## sample estimates:
##         p 
## 0.1081081
```

```
#Confidence-intervals around sample proportion relative to expected proportion
round(prop.test(x = Observation[2], n = sum(Observation), conf.level = .95)$estimate/expected_ratio_6574,2)
```

```
##    p 
## 0.94
```

```
round(as.vector(prop.test(x = Observation[2], n = sum(Observation), conf.level = .95)$conf.int)/expected_ratio_6574,2)
```

```
## [1] 0.57 1.50
```

```
#Confidence-intervals around sample proportion
prop.test(x = Observation[3], n = sum(Observation), conf.level = .95)
```

```
## 
##  1-sample proportions test with continuity correction
## 
## data:  Observation[3] out of sum(Observation), null probability 0.5
## X-squared = 130.55, df = 1, p-value < 2.2e-16
## alternative hypothesis: true p is not equal to 0.5
## 95 percent confidence interval:
##  0.008692983 0.072068030
## sample estimates:
##          p 
## 0.02702703
```

```
#Confidence-intervals around sample proportion relative to expected proportion
round(prop.test(x = Observation[3], n = sum(Observation), conf.level = .95)$estimate/expected_ratio_75plus,2)
```

```
##    p 
## 0.23
```

```
round(as.vector(prop.test(x = Observation[3], n = sum(Observation), conf.level = .95)$conf.int)/expected_ratio_75plus,2)
```

```
## [1] 0.07 0.60
```

```
###########################
#Specific phobias#
###########################
prevalence_1834 <- as.numeric(Prevalence_estimates[Prevalence_estimates$Diagnosis == "Specific phobias", "1834y"])
prevalence_3549 <- as.numeric(Prevalence_estimates[Prevalence_estimates$Diagnosis == "Specific phobias", "3549y"])
prevalence_5064 <- as.numeric(Prevalence_estimates[Prevalence_estimates$Diagnosis == "Specific phobias", "5064y"])
prevalence_6574 <- as.numeric(Prevalence_estimates[Prevalence_estimates$Diagnosis == "Specific phobias", "6574y"])
prevalence_75plus <- as.numeric(Prevalence_estimates[Prevalence_estimates$Diagnosis == "Specific phobias", "75yplus"])

expected_ratio_1834 <- (census_amount_1834*prevalence_1834)/((census_amount_1834*prevalence_1834)+(census_amount_3549*prevalence_3549)+(census_amount_5064*prevalence_5064)+(census_amount_6574*prevalence_6574)+(census_amount_75plus*prevalence_75plus))
expected_ratio_3549 <- (census_amount_3549*prevalence_3549)/((census_amount_1834*prevalence_1834)+(census_amount_3549*prevalence_3549)+(census_amount_5064*prevalence_5064)+(census_amount_6574*prevalence_6574)+(census_amount_75plus*prevalence_75plus))
expected_ratio_5064 <- (census_amount_5064*prevalence_5064)/((census_amount_1834*prevalence_1834)+(census_amount_3549*prevalence_3549)+(census_amount_5064*prevalence_5064)+(census_amount_6574*prevalence_6574)+(census_amount_75plus*prevalence_75plus))
expected_ratio_6574 <- (census_amount_6574*prevalence_6574)/((census_amount_1834*prevalence_1834)+(census_amount_3549*prevalence_3549)+(census_amount_5064*prevalence_5064)+(census_amount_6574*prevalence_6574)+(census_amount_75plus*prevalence_75plus))
expected_ratio_75plus <- (census_amount_75plus*prevalence_75plus)/((census_amount_1834*prevalence_1834)+(census_amount_3549*prevalence_3549)+(census_amount_5064*prevalence_5064)+(census_amount_6574*prevalence_6574)+(census_amount_75plus*prevalence_75plus))

round(c(expected_ratio_1834,expected_ratio_3549,expected_ratio_5064,expected_ratio_6574,expected_ratio_75plus), 2)
```

```
## [1] 0.29 0.21 0.29 0.11 0.11
```

```
KODAP_data_complete_specificphobia <- KODAP_data_complete[KODAP_data_complete$ICD1_pre_recode %in% c("F40.2 Spezifische Phobie") |
                                                              KODAP_data_complete$ICD2_pre_recode %in% c("F40.2 Spezifische Phobie") |
                                                              KODAP_data_complete$ICD3_pre_recode %in% c("F40.2 Spezifische Phobie") |
                                                              KODAP_data_complete$ICD4_pre_recode %in% c("F40.2 Spezifische Phobie") |
                                                              KODAP_data_complete$ICD5_pre_recode %in% c("F40.2 Spezifische Phobie"),]

table(KODAP_data_complete_specificphobia$Age_Stepped_2)
```

```
## 
##   0   1   2   3   4 
## 407 154 117  22   3
```

```
round(table(KODAP_data_complete_specificphobia$Age_Stepped_2)/length(KODAP_data_complete_specificphobia$Age_Stepped_2)*100, 1)
```

```
## 
##    0    1    2    3    4 
## 57.9 21.9 16.6  3.1  0.4
```

```
#18-34 years
prevalence_1834 <- as.numeric(Prevalence_estimates[Prevalence_estimates$Diagnosis == "Specific phobias", "1834y"])
prevalence_3549 <- as.numeric(Prevalence_estimates[Prevalence_estimates$Diagnosis == "Specific phobias", "3549y"])
prevalence_5064 <- as.numeric(Prevalence_estimates[Prevalence_estimates$Diagnosis == "Specific phobias", "5064y"])

prevalence_1864 <- prevalence_1834

prevalence_6574 <- as.numeric(Prevalence_estimates[Prevalence_estimates$Diagnosis == "Specific phobias", "6574y"])
prevalence_75plus <- as.numeric(Prevalence_estimates[Prevalence_estimates$Diagnosis == "Specific phobias", "75yplus"])

#Ratios in reference population
expected_ratio_1864 <- (census_amount_1834*prevalence_1864)/((census_amount_1834*prevalence_1864)+(census_amount_6574*prevalence_6574)+(census_amount_75plus*prevalence_75plus))
expected_ratio_6574 <- (census_amount_6574*prevalence_6574)/((census_amount_1834*prevalence_1864)+(census_amount_6574*prevalence_6574)+(census_amount_75plus*prevalence_75plus))
expected_ratio_75plus <- (census_amount_75plus*prevalence_75plus)/((census_amount_1834*prevalence_1864)+(census_amount_6574*prevalence_6574)+(census_amount_75plus*prevalence_75plus))

expected_ratios <- c(expected_ratio_1864,
                     expected_ratio_6574,
                     expected_ratio_75plus)
round(expected_ratios,2)
```

```
## [1] 0.57 0.21 0.22
```

```
KODAP_data_complete_specificphobia <- KODAP_data_complete_s1[KODAP_data_complete_s1$ICD1_pre_recode %in% c("F40.2 Spezifische Phobie") |
                                                             KODAP_data_complete_s1$ICD2_pre_recode %in% c("F40.2 Spezifische Phobie") |
                                                             KODAP_data_complete_s1$ICD3_pre_recode %in% c("F40.2 Spezifische Phobie") |
                                                             KODAP_data_complete_s1$ICD4_pre_recode %in% c("F40.2 Spezifische Phobie") |
                                                             KODAP_data_complete_s1$ICD5_pre_recode %in% c("F40.2 Spezifische Phobie"),]

length(KODAP_data_complete_specificphobia$Patient_ID)
```

```
## [1] 432
```

```
#Observed vs. expected: 
Observation <- as.vector(table(KODAP_data_complete_specificphobia$Age_Stepped))
Expected <- expected_ratios*length(KODAP_data_complete_specificphobia$Patient_ID)

Observation
```

```
## [1] 407  22   3
```

```
round(Observation/length(KODAP_data_complete_specificphobia$Patient_ID)*100,1)
```

```
## [1] 94.2  5.1  0.7
```

```
# Chi-squared test for given probabilities
test <- chisq.test(Observation, p = expected_ratios)
test
```

```
## 
##  Chi-squared test for given probabilities
## 
## data:  Observation
## X-squared = 245.39, df = 2, p-value < 2.2e-16
```

```
# Post-hoc binomial-test for each category with Bonferroni-correction
binom.test(Observation[1], sum(Observation), expected_ratios[1])$p.value*3
```

```
## [1] 6.210734e-68
```

```
binom.test(Observation[2], sum(Observation), expected_ratios[2])$p.value*3
```

```
## [1] 3.918618e-20
```

```
binom.test(Observation[3], sum(Observation), expected_ratios[3])$p.value*3
```

```
## [1] 4.551319e-41
```

```
#Estimating underrepresentation compared to reference population
#Representation quotients
Observation/Expected
```

```
## [1] 1.65100426 0.24257153 0.03164942
```

```
#Confidence-intervals of Representation quotients
#Confidence-intervals around sample proportion
prop.test(x = Observation[2], n = sum(Observation), conf.level = .95)
```

```
## 
##  1-sample proportions test with continuity correction
## 
## data:  Observation[2] out of sum(Observation), null probability 0.5
## X-squared = 346.69, df = 1, p-value < 2.2e-16
## alternative hypothesis: true p is not equal to 0.5
## 95 percent confidence interval:
##  0.03293848 0.07726170
## sample estimates:
##          p 
## 0.05092593
```

```
#Confidence-intervals around sample proportion relative to expected proportion
round(prop.test(x = Observation[2], n = sum(Observation), conf.level = .95)$estimate/expected_ratio_6574,2)
```

```
##    p 
## 0.24
```

```
round(as.vector(prop.test(x = Observation[2], n = sum(Observation), conf.level = .95)$conf.int)/expected_ratio_6574,2)
```

```
## [1] 0.16 0.37
```

```
#Confidence-intervals around sample proportion
prop.test(x = Observation[3], n = sum(Observation), conf.level = .95)
```

```
## 
##  1-sample proportions test with continuity correction
## 
## data:  Observation[3] out of sum(Observation), null probability 0.5
## X-squared = 418.11, df = 1, p-value < 2.2e-16
## alternative hypothesis: true p is not equal to 0.5
## 95 percent confidence interval:
##  0.001795201 0.021904551
## sample estimates:
##           p 
## 0.006944444
```

```
#Confidence-intervals around sample proportion relative to expected proportion
round(prop.test(x = Observation[3], n = sum(Observation), conf.level = .95)$estimate/expected_ratio_75plus,2)
```

```
##    p 
## 0.03
```

```
round(as.vector(prop.test(x = Observation[3], n = sum(Observation), conf.level = .95)$conf.int)/expected_ratio_75plus,2)
```

```
## [1] 0.01 0.10
```

```
#35-49 years
prevalence_1834 <- as.numeric(Prevalence_estimates[Prevalence_estimates$Diagnosis == "Specific phobias", "1834y"])
prevalence_3549 <- as.numeric(Prevalence_estimates[Prevalence_estimates$Diagnosis == "Specific phobias", "3549y"])
prevalence_5064 <- as.numeric(Prevalence_estimates[Prevalence_estimates$Diagnosis == "Specific phobias", "5064y"])

prevalence_1864 <- prevalence_3549

prevalence_6574 <- as.numeric(Prevalence_estimates[Prevalence_estimates$Diagnosis == "Specific phobias", "6574y"])
prevalence_75plus <- as.numeric(Prevalence_estimates[Prevalence_estimates$Diagnosis == "Specific phobias", "75yplus"])

#Ratios in reference population
expected_ratio_1864 <- (census_amount_3549*prevalence_1864)/((census_amount_3549*prevalence_1864)+(census_amount_6574*prevalence_6574)+(census_amount_75plus*prevalence_75plus))
expected_ratio_6574 <- (census_amount_6574*prevalence_6574)/((census_amount_3549*prevalence_1864)+(census_amount_6574*prevalence_6574)+(census_amount_75plus*prevalence_75plus))
expected_ratio_75plus <- (census_amount_75plus*prevalence_75plus)/((census_amount_3549*prevalence_1864)+(census_amount_6574*prevalence_6574)+(census_amount_75plus*prevalence_75plus))

expected_ratios <- c(expected_ratio_1864,
                     expected_ratio_6574,
                     expected_ratio_75plus)
round(expected_ratios,2)
```

```
## [1] 0.49 0.25 0.26
```

```
KODAP_data_complete_specificphobia <- KODAP_data_complete_s2[KODAP_data_complete_s2$ICD1_pre_recode %in% c("F40.2 Spezifische Phobie") |
                                                             KODAP_data_complete_s2$ICD2_pre_recode %in% c("F40.2 Spezifische Phobie") |
                                                             KODAP_data_complete_s2$ICD3_pre_recode %in% c("F40.2 Spezifische Phobie") |
                                                             KODAP_data_complete_s2$ICD4_pre_recode %in% c("F40.2 Spezifische Phobie") |
                                                             KODAP_data_complete_s2$ICD5_pre_recode %in% c("F40.2 Spezifische Phobie"),]

length(KODAP_data_complete_specificphobia$Patient_ID)
```

```
## [1] 179
```

```
#Observed vs. expected: 
Observation <- as.vector(table(KODAP_data_complete_specificphobia$Age_Stepped))
Expected <- expected_ratios*length(KODAP_data_complete_specificphobia$Patient_ID)

Observation
```

```
## [1] 154  22   3
```

```
round(Observation/length(KODAP_data_complete_specificphobia$Patient_ID)*100,1)
```

```
## [1] 86.0 12.3  1.7
```

```
# Chi-squared test for given probabilities
test <- chisq.test(Observation, p = expected_ratios)
test
```

```
## 
##  Chi-squared test for given probabilities
## 
## data:  Observation
## X-squared = 102.78, df = 2, p-value < 2.2e-16
```

```
# Post-hoc binomial-test for each category with Bonferroni-correction
binom.test(Observation[1], sum(Observation), expected_ratios[1])$p.value*3
```

```
## [1] 7.562553e-25
```

```
binom.test(Observation[2], sum(Observation), expected_ratios[2])$p.value*3
```

```
## [1] 0.000125186
```

```
binom.test(Observation[3], sum(Observation), expected_ratios[3])$p.value*3
```

```
## [1] 5.342115e-19
```

```
#Estimating underrepresentation compared to reference population
#Representation quotients
Observation/Expected
```

```
## [1] 1.75814235 0.49222397 0.06422271
```

```
#Confidence-intervals of Representation quotients
#Confidence-intervals around sample proportion
prop.test(x = Observation[2], n = sum(Observation), conf.level = .95)
```

```
## 
##  1-sample proportions test with continuity correction
## 
## data:  Observation[2] out of sum(Observation), null probability 0.5
## X-squared = 100.31, df = 1, p-value < 2.2e-16
## alternative hypothesis: true p is not equal to 0.5
## 95 percent confidence interval:
##  0.08030065 0.18225298
## sample estimates:
##        p 
## 0.122905
```

```
#Confidence-intervals around sample proportion relative to expected proportion
round(prop.test(x = Observation[2], n = sum(Observation), conf.level = .95)$estimate/expected_ratio_6574,2)
```

```
##    p 
## 0.49
```

```
round(as.vector(prop.test(x = Observation[2], n = sum(Observation), conf.level = .95)$conf.int)/expected_ratio_6574,2)
```

```
## [1] 0.32 0.73
```

```
#Confidence-intervals around sample proportion
prop.test(x = Observation[3], n = sum(Observation), conf.level = .95)
```

```
## 
##  1-sample proportions test with continuity correction
## 
## data:  Observation[3] out of sum(Observation), null probability 0.5
## X-squared = 165.27, df = 1, p-value < 2.2e-16
## alternative hypothesis: true p is not equal to 0.5
## 95 percent confidence interval:
##  0.004338373 0.052111880
## sample estimates:
##          p 
## 0.01675978
```

```
#Confidence-intervals around sample proportion relative to expected proportion
round(prop.test(x = Observation[3], n = sum(Observation), conf.level = .95)$estimate/expected_ratio_75plus,2)
```

```
##    p 
## 0.06
```

```
round(as.vector(prop.test(x = Observation[3], n = sum(Observation), conf.level = .95)$conf.int)/expected_ratio_75plus,2)
```

```
## [1] 0.02 0.20
```

```
#50-64 years
prevalence_1834 <- as.numeric(Prevalence_estimates[Prevalence_estimates$Diagnosis == "Specific phobias", "1834y"])
prevalence_3549 <- as.numeric(Prevalence_estimates[Prevalence_estimates$Diagnosis == "Specific phobias", "3549y"])
prevalence_5064 <- as.numeric(Prevalence_estimates[Prevalence_estimates$Diagnosis == "Specific phobias", "5064y"])

prevalence_1864 <- prevalence_5064

prevalence_6574 <- as.numeric(Prevalence_estimates[Prevalence_estimates$Diagnosis == "Specific phobias", "6574y"])
prevalence_75plus <- as.numeric(Prevalence_estimates[Prevalence_estimates$Diagnosis == "Specific phobias", "75yplus"])

#Ratios in reference population
expected_ratio_1864 <- (census_amount_5064*prevalence_1864)/((census_amount_5064*prevalence_1864)+(census_amount_6574*prevalence_6574)+(census_amount_75plus*prevalence_75plus))
expected_ratio_6574 <- (census_amount_6574*prevalence_6574)/((census_amount_5064*prevalence_1864)+(census_amount_6574*prevalence_6574)+(census_amount_75plus*prevalence_75plus))
expected_ratio_75plus <- (census_amount_75plus*prevalence_75plus)/((census_amount_5064*prevalence_1864)+(census_amount_6574*prevalence_6574)+(census_amount_75plus*prevalence_75plus))

expected_ratios <- c(expected_ratio_1864,
                     expected_ratio_6574,
                     expected_ratio_75plus)
round(expected_ratios,2)
```

```
## [1] 0.57 0.21 0.22
```

```
KODAP_data_complete_specificphobia <- KODAP_data_complete_s3[KODAP_data_complete_s3$ICD1_pre_recode %in% c("F40.2 Spezifische Phobie") |
                                                             KODAP_data_complete_s3$ICD2_pre_recode %in% c("F40.2 Spezifische Phobie") |
                                                             KODAP_data_complete_s3$ICD3_pre_recode %in% c("F40.2 Spezifische Phobie") |
                                                             KODAP_data_complete_s3$ICD4_pre_recode %in% c("F40.2 Spezifische Phobie") |
                                                             KODAP_data_complete_s3$ICD5_pre_recode %in% c("F40.2 Spezifische Phobie"),]

length(KODAP_data_complete_specificphobia$Patient_ID)
```

```
## [1] 142
```

```
#Observed vs. expected: 
Observation <- as.vector(table(KODAP_data_complete_specificphobia$Age_Stepped))
Expected <- expected_ratios*length(KODAP_data_complete_specificphobia$Patient_ID)

Observation
```

```
## [1] 117  22   3
```

```
round(Observation/length(KODAP_data_complete_specificphobia$Patient_ID)*100,1)
```

```
## [1] 82.4 15.5  2.1
```

```
# Chi-squared test for given probabilities
test <- chisq.test(Observation, p = expected_ratios)
test
```

```
## 
##  Chi-squared test for given probabilities
## 
## data:  Observation
## X-squared = 42.692, df = 2, p-value = 5.366e-10
```

```
# Post-hoc binomial-test for each category with Bonferroni-correction
binom.test(Observation[1], sum(Observation), expected_ratios[1])$p.value*3
```

```
## [1] 1.183246e-09
```

```
binom.test(Observation[2], sum(Observation), expected_ratios[2])$p.value*3
```

```
## [1] 0.3660296
```

```
binom.test(Observation[3], sum(Observation), expected_ratios[3])$p.value*3
```

```
## [1] 4.056432e-11
```

```
#Estimating underrepresentation compared to reference population
#Representation quotients
Observation/Expected
```

```
## [1] 1.43633485 0.74316156 0.09696369
```

```
#Confidence-intervals of Representation quotients
#Confidence-intervals around sample proportion
prop.test(x = Observation[2], n = sum(Observation), conf.level = .95)
```

```
## 
##  1-sample proportions test with continuity correction
## 
## data:  Observation[2] out of sum(Observation), null probability 0.5
## X-squared = 66.261, df = 1, p-value = 3.951e-16
## alternative hypothesis: true p is not equal to 0.5
## 95 percent confidence interval:
##  0.1016958 0.2273905
## sample estimates:
##         p 
## 0.1549296
```

```
#Confidence-intervals around sample proportion relative to expected proportion
round(prop.test(x = Observation[2], n = sum(Observation), conf.level = .95)$estimate/expected_ratio_6574,2)
```

```
##    p 
## 0.74
```

```
round(as.vector(prop.test(x = Observation[2], n = sum(Observation), conf.level = .95)$conf.int)/expected_ratio_6574,2)
```

```
## [1] 0.49 1.09
```

```
#Confidence-intervals around sample proportion
prop.test(x = Observation[3], n = sum(Observation), conf.level = .95)
```

```
## 
##  1-sample proportions test with continuity correction
## 
## data:  Observation[3] out of sum(Observation), null probability 0.5
## X-squared = 128.35, df = 1, p-value < 2.2e-16
## alternative hypothesis: true p is not equal to 0.5
## 95 percent confidence interval:
##  0.005472072 0.065275438
## sample estimates:
##          p 
## 0.02112676
```

```
#Confidence-intervals around sample proportion relative to expected proportion
round(prop.test(x = Observation[3], n = sum(Observation), conf.level = .95)$estimate/expected_ratio_75plus,2)
```

```
##   p 
## 0.1
```

```
round(as.vector(prop.test(x = Observation[3], n = sum(Observation), conf.level = .95)$conf.int)/expected_ratio_75plus,2)
```

```
## [1] 0.03 0.30
```

```
###########################
#Generalized Anxiety Disorder#
###########################
prevalence_1834 <- as.numeric(Prevalence_estimates[Prevalence_estimates$Diagnosis == "GAD", "1834y"])
prevalence_3549 <- as.numeric(Prevalence_estimates[Prevalence_estimates$Diagnosis == "GAD", "3549y"])
prevalence_5064 <- as.numeric(Prevalence_estimates[Prevalence_estimates$Diagnosis == "GAD", "5064y"])
prevalence_6574 <- as.numeric(Prevalence_estimates[Prevalence_estimates$Diagnosis == "GAD", "6574y"])
prevalence_75plus <- as.numeric(Prevalence_estimates[Prevalence_estimates$Diagnosis == "GAD", "75yplus"])

expected_ratio_1834 <- (census_amount_1834*prevalence_1834)/((census_amount_1834*prevalence_1834)+(census_amount_3549*prevalence_3549)+(census_amount_5064*prevalence_5064)+(census_amount_6574*prevalence_6574)+(census_amount_75plus*prevalence_75plus))
expected_ratio_3549 <- (census_amount_3549*prevalence_3549)/((census_amount_1834*prevalence_1834)+(census_amount_3549*prevalence_3549)+(census_amount_5064*prevalence_5064)+(census_amount_6574*prevalence_6574)+(census_amount_75plus*prevalence_75plus))
expected_ratio_5064 <- (census_amount_5064*prevalence_5064)/((census_amount_1834*prevalence_1834)+(census_amount_3549*prevalence_3549)+(census_amount_5064*prevalence_5064)+(census_amount_6574*prevalence_6574)+(census_amount_75plus*prevalence_75plus))
expected_ratio_6574 <- (census_amount_6574*prevalence_6574)/((census_amount_1834*prevalence_1834)+(census_amount_3549*prevalence_3549)+(census_amount_5064*prevalence_5064)+(census_amount_6574*prevalence_6574)+(census_amount_75plus*prevalence_75plus))
expected_ratio_75plus <- (census_amount_75plus*prevalence_75plus)/((census_amount_1834*prevalence_1834)+(census_amount_3549*prevalence_3549)+(census_amount_5064*prevalence_5064)+(census_amount_6574*prevalence_6574)+(census_amount_75plus*prevalence_75plus))

round(c(expected_ratio_1834,expected_ratio_3549,expected_ratio_5064,expected_ratio_6574,expected_ratio_75plus), 2)
```

```
## [1] 0.36 0.20 0.28 0.08 0.08
```

```
KODAP_data_complete_GAD <- KODAP_data_complete[KODAP_data_complete$ICD1_pre_recode %in% c("F41.1 Generalisierte Angststörung") |
                                                            KODAP_data_complete$ICD2_pre_recode %in% c("F41.1 Generalisierte Angststörung") |
                                                            KODAP_data_complete$ICD3_pre_recode %in% c("F41.1 Generalisierte Angststörung") |
                                                            KODAP_data_complete$ICD4_pre_recode %in% c("F41.1 Generalisierte Angststörung") |
                                                            KODAP_data_complete$ICD5_pre_recode %in% c("F41.1 Generalisierte Angststörung"),]

table(KODAP_data_complete_GAD$Age_Stepped_2)
```

```
## 
##   0   1   2   3   4 
## 254 147 125  20   6
```

```
round(table(KODAP_data_complete_GAD$Age_Stepped_2)/length(KODAP_data_complete_GAD$Age_Stepped_2)*100, 1)
```

```
## 
##    0    1    2    3    4 
## 46.0 26.6 22.6  3.6  1.1
```

```
#18-34 years
prevalence_1834 <- as.numeric(Prevalence_estimates[Prevalence_estimates$Diagnosis == "GAD", "1834y"])
prevalence_3549 <- as.numeric(Prevalence_estimates[Prevalence_estimates$Diagnosis == "GAD", "3549y"])
prevalence_5064 <- as.numeric(Prevalence_estimates[Prevalence_estimates$Diagnosis == "GAD", "5064y"])

prevalence_1864 <- prevalence_1834

prevalence_6574 <- as.numeric(Prevalence_estimates[Prevalence_estimates$Diagnosis == "GAD", "6574y"])
prevalence_75plus <- as.numeric(Prevalence_estimates[Prevalence_estimates$Diagnosis == "GAD", "75yplus"])

#Ratios in reference population
expected_ratio_1864 <- (census_amount_1834*prevalence_1864)/((census_amount_1834*prevalence_1864)+(census_amount_6574*prevalence_6574)+(census_amount_75plus*prevalence_75plus))
expected_ratio_6574 <- (census_amount_6574*prevalence_6574)/((census_amount_1834*prevalence_1864)+(census_amount_6574*prevalence_6574)+(census_amount_75plus*prevalence_75plus))
expected_ratio_75plus <- (census_amount_75plus*prevalence_75plus)/((census_amount_1834*prevalence_1864)+(census_amount_6574*prevalence_6574)+(census_amount_75plus*prevalence_75plus))

expected_ratios <- c(expected_ratio_1864,
                     expected_ratio_6574,
                     expected_ratio_75plus)
round(expected_ratios,2)
```

```
## [1] 0.70 0.15 0.15
```

```
KODAP_data_complete_GAD <- KODAP_data_complete_s1[KODAP_data_complete_s1$ICD1_pre_recode %in% c("F41.1 Generalisierte Angststörung") |
                                                               KODAP_data_complete_s1$ICD2_pre_recode %in% c("F41.1 Generalisierte Angststörung") |
                                                               KODAP_data_complete_s1$ICD3_pre_recode %in% c("F41.1 Generalisierte Angststörung") |
                                                               KODAP_data_complete_s1$ICD4_pre_recode %in% c("F41.1 Generalisierte Angststörung") |
                                                               KODAP_data_complete_s1$ICD5_pre_recode %in% c("F41.1 Generalisierte Angststörung"),]

length(KODAP_data_complete_GAD$Patient_ID)
```

```
## [1] 280
```

```
#Observed vs. expected: 
Observation <- as.vector(table(KODAP_data_complete_GAD$Age_Stepped))
Expected <- expected_ratios*length(KODAP_data_complete_GAD$Patient_ID)

Observation
```

```
## [1] 254  20   6
```

```
round(Observation/length(KODAP_data_complete_GAD$Patient_ID)*100,1)
```

```
## [1] 90.7  7.1  2.1
```

```
# Chi-squared test for given probabilities
test <- chisq.test(Observation, p = expected_ratios)
test
```

```
## 
##  Chi-squared test for given probabilities
## 
## data:  Observation
## X-squared = 60.904, df = 2, p-value = 5.955e-14
```

```
# Post-hoc binomial-test for each category with Bonferroni-correction
binom.test(Observation[1], sum(Observation), expected_ratios[1])$p.value*3
```

```
## [1] 1.331386e-16
```

```
binom.test(Observation[2], sum(Observation), expected_ratios[2])$p.value*3
```

```
## [1] 0.0004063366
```

```
binom.test(Observation[3], sum(Observation), expected_ratios[3])$p.value*3
```

```
## [1] 4.57727e-13
```

```
#Estimating underrepresentation compared to reference population
#Representation quotients
Observation/Expected
```

```
## [1] 1.3008649 0.4826548 0.1385432
```

```
#Confidence-intervals of Representation quotients
#Confidence-intervals around sample proportion
prop.test(x = Observation[2], n = sum(Observation), conf.level = .95)
```

```
## 
##  1-sample proportions test with continuity correction
## 
## data:  Observation[2] out of sum(Observation), null probability 0.5
## X-squared = 204, df = 1, p-value < 2.2e-16
## alternative hypothesis: true p is not equal to 0.5
## 95 percent confidence interval:
##  0.0452875 0.1098397
## sample estimates:
##          p 
## 0.07142857
```

```
#Confidence-intervals around sample proportion relative to expected proportion
round(prop.test(x = Observation[2], n = sum(Observation), conf.level = .95)$estimate/expected_ratio_6574,2)
```

```
##    p 
## 0.48
```

```
round(as.vector(prop.test(x = Observation[2], n = sum(Observation), conf.level = .95)$conf.int)/expected_ratio_6574,2)
```

```
## [1] 0.31 0.74
```

```
#Confidence-intervals around sample proportion
prop.test(x = Observation[3], n = sum(Observation), conf.level = .95)
```

```
## 
##  1-sample proportions test with continuity correction
## 
## data:  Observation[3] out of sum(Observation), null probability 0.5
## X-squared = 254.6, df = 1, p-value < 2.2e-16
## alternative hypothesis: true p is not equal to 0.5
## 95 percent confidence interval:
##  0.008740337 0.048335768
## sample estimates:
##          p 
## 0.02142857
```

```
#Confidence-intervals around sample proportion relative to expected proportion
round(prop.test(x = Observation[3], n = sum(Observation), conf.level = .95)$estimate/expected_ratio_75plus,2)
```

```
##    p 
## 0.14
```

```
round(as.vector(prop.test(x = Observation[3], n = sum(Observation), conf.level = .95)$conf.int)/expected_ratio_75plus,2)
```

```
## [1] 0.06 0.31
```

```
#35-49 years
prevalence_1834 <- as.numeric(Prevalence_estimates[Prevalence_estimates$Diagnosis == "GAD", "1834y"])
prevalence_3549 <- as.numeric(Prevalence_estimates[Prevalence_estimates$Diagnosis == "GAD", "3549y"])
prevalence_5064 <- as.numeric(Prevalence_estimates[Prevalence_estimates$Diagnosis == "GAD", "5064y"])

prevalence_1864 <- prevalence_3549

prevalence_6574 <- as.numeric(Prevalence_estimates[Prevalence_estimates$Diagnosis == "GAD", "6574y"])
prevalence_75plus <- as.numeric(Prevalence_estimates[Prevalence_estimates$Diagnosis == "GAD", "75yplus"])

#Ratios in reference population
expected_ratio_1864 <- (census_amount_3549*prevalence_1864)/((census_amount_3549*prevalence_1864)+(census_amount_6574*prevalence_6574)+(census_amount_75plus*prevalence_75plus))
expected_ratio_6574 <- (census_amount_6574*prevalence_6574)/((census_amount_3549*prevalence_1864)+(census_amount_6574*prevalence_6574)+(census_amount_75plus*prevalence_75plus))
expected_ratio_75plus <- (census_amount_75plus*prevalence_75plus)/((census_amount_3549*prevalence_1864)+(census_amount_6574*prevalence_6574)+(census_amount_75plus*prevalence_75plus))

expected_ratios <- c(expected_ratio_1864,
                     expected_ratio_6574,
                     expected_ratio_75plus)
round(expected_ratios,2)
```

```
## [1] 0.57 0.21 0.22
```

```
KODAP_data_complete_GAD <- KODAP_data_complete_s2[KODAP_data_complete_s2$ICD1_pre_recode %in% c("F41.1 Generalisierte Angststörung") |
                                                               KODAP_data_complete_s2$ICD2_pre_recode %in% c("F41.1 Generalisierte Angststörung") |
                                                               KODAP_data_complete_s2$ICD3_pre_recode %in% c("F41.1 Generalisierte Angststörung") |
                                                               KODAP_data_complete_s2$ICD4_pre_recode %in% c("F41.1 Generalisierte Angststörung") |
                                                               KODAP_data_complete_s2$ICD5_pre_recode %in% c("F41.1 Generalisierte Angststörung"),]

length(KODAP_data_complete_GAD$Patient_ID)
```

```
## [1] 173
```

```
#Observed vs. expected: 
Observation <- as.vector(table(KODAP_data_complete_GAD$Age_Stepped))
Expected <- expected_ratios*length(KODAP_data_complete_GAD$Patient_ID)

Observation
```

```
## [1] 147  20   6
```

```
round(Observation/length(KODAP_data_complete_GAD$Patient_ID)*100,1)
```

```
## [1] 85.0 11.6  3.5
```

```
# Chi-squared test for given probabilities
test <- chisq.test(Observation, p = expected_ratios)
test
```

```
## 
##  Chi-squared test for given probabilities
## 
## data:  Observation
## X-squared = 59.559, df = 2, p-value = 1.166e-13
```

```
# Post-hoc binomial-test for each category with Bonferroni-correction
binom.test(Observation[1], sum(Observation), expected_ratios[1])$p.value*3
```

```
## [1] 7.14875e-15
```

```
binom.test(Observation[2], sum(Observation), expected_ratios[2])$p.value*3
```

```
## [1] 0.004372577
```

```
binom.test(Observation[3], sum(Observation), expected_ratios[3])$p.value*3
```

```
## [1] 1.60061e-11
```

```
#Estimating underrepresentation compared to reference population
#Representation quotients
Observation/Expected
```

```
## [1] 1.5015559 0.5446321 0.1563334
```

```
#Confidence-intervals of Representation quotients
#Confidence-intervals around sample proportion
prop.test(x = Observation[2], n = sum(Observation), conf.level = .95)
```

```
## 
##  1-sample proportions test with continuity correction
## 
## data:  Observation[2] out of sum(Observation), null probability 0.5
## X-squared = 100.72, df = 1, p-value < 2.2e-16
## alternative hypothesis: true p is not equal to 0.5
## 95 percent confidence interval:
##  0.07376618 0.17513438
## sample estimates:
##         p 
## 0.1156069
```

```
#Confidence-intervals around sample proportion relative to expected proportion
round(prop.test(x = Observation[2], n = sum(Observation), conf.level = .95)$estimate/expected_ratio_6574,2)
```

```
##    p 
## 0.54
```

```
round(as.vector(prop.test(x = Observation[2], n = sum(Observation), conf.level = .95)$conf.int)/expected_ratio_6574,2)
```

```
## [1] 0.35 0.83
```

```
#Confidence-intervals around sample proportion
prop.test(x = Observation[3], n = sum(Observation), conf.level = .95)
```

```
## 
##  1-sample proportions test with continuity correction
## 
## data:  Observation[3] out of sum(Observation), null probability 0.5
## X-squared = 147.98, df = 1, p-value < 2.2e-16
## alternative hypothesis: true p is not equal to 0.5
## 95 percent confidence interval:
##  0.01417609 0.07738998
## sample estimates:
##          p 
## 0.03468208
```

```
#Confidence-intervals around sample proportion relative to expected proportion
round(prop.test(x = Observation[3], n = sum(Observation), conf.level = .95)$estimate/expected_ratio_75plus,2)
```

```
##    p 
## 0.16
```

```
round(as.vector(prop.test(x = Observation[3], n = sum(Observation), conf.level = .95)$conf.int)/expected_ratio_75plus,2)
```

```
## [1] 0.06 0.35
```

```
#50-64 years
prevalence_1834 <- as.numeric(Prevalence_estimates[Prevalence_estimates$Diagnosis == "GAD", "1834y"])
prevalence_3549 <- as.numeric(Prevalence_estimates[Prevalence_estimates$Diagnosis == "GAD", "3549y"])
prevalence_5064 <- as.numeric(Prevalence_estimates[Prevalence_estimates$Diagnosis == "GAD", "5064y"])

prevalence_1864 <- prevalence_5064

prevalence_6574 <- as.numeric(Prevalence_estimates[Prevalence_estimates$Diagnosis == "GAD", "6574y"])
prevalence_75plus <- as.numeric(Prevalence_estimates[Prevalence_estimates$Diagnosis == "GAD", "75yplus"])

#Ratios in reference population
expected_ratio_1864 <- (census_amount_5064*prevalence_1864)/((census_amount_5064*prevalence_1864)+(census_amount_6574*prevalence_6574)+(census_amount_75plus*prevalence_75plus))
expected_ratio_6574 <- (census_amount_6574*prevalence_6574)/((census_amount_5064*prevalence_1864)+(census_amount_6574*prevalence_6574)+(census_amount_75plus*prevalence_75plus))
expected_ratio_75plus <- (census_amount_75plus*prevalence_75plus)/((census_amount_5064*prevalence_1864)+(census_amount_6574*prevalence_6574)+(census_amount_75plus*prevalence_75plus))

expected_ratios <- c(expected_ratio_1864,
                     expected_ratio_6574,
                     expected_ratio_75plus)
round(expected_ratios,2)
```

```
## [1] 0.65 0.17 0.18
```

```
KODAP_data_complete_GAD <- KODAP_data_complete_s3[KODAP_data_complete_s3$ICD1_pre_recode %in% c("F41.1 Generalisierte Angststörung") |
                                                               KODAP_data_complete_s3$ICD2_pre_recode %in% c("F41.1 Generalisierte Angststörung") |
                                                               KODAP_data_complete_s3$ICD3_pre_recode %in% c("F41.1 Generalisierte Angststörung") |
                                                               KODAP_data_complete_s3$ICD4_pre_recode %in% c("F41.1 Generalisierte Angststörung") |
                                                               KODAP_data_complete_s3$ICD5_pre_recode %in% c("F41.1 Generalisierte Angststörung"),]

length(KODAP_data_complete_GAD$Patient_ID)
```

```
## [1] 151
```

```
#Observed vs. expected: 
Observation <- as.vector(table(KODAP_data_complete_GAD$Age_Stepped))
Expected <- expected_ratios*length(KODAP_data_complete_GAD$Patient_ID)

Observation
```

```
## [1] 125  20   6
```

```
round(Observation/length(KODAP_data_complete_GAD$Patient_ID)*100,1)
```

```
## [1] 82.8 13.2  4.0
```

```
# Chi-squared test for given probabilities
test <- chisq.test(Observation, p = expected_ratios)
test
```

```
## 
##  Chi-squared test for given probabilities
## 
## data:  Observation
## X-squared = 25.563, df = 2, p-value = 2.813e-06
```

```
# Post-hoc binomial-test for each category with Bonferroni-correction
binom.test(Observation[1], sum(Observation), expected_ratios[1])$p.value*3
```

```
## [1] 4.065834e-06
```

```
binom.test(Observation[2], sum(Observation), expected_ratios[2])$p.value*3
```

```
## [1] 0.7057972
```

```
binom.test(Observation[3], sum(Observation), expected_ratios[3])$p.value*3
```

```
## [1] 1.094089e-06
```

```
#Estimating underrepresentation compared to reference population
#Representation quotients
Observation/Expected
```

```
## [1] 1.2790750 0.7677927 0.2203903
```

```
#Confidence-intervals of Representation quotients
#Confidence-intervals around sample proportion
prop.test(x = Observation[2], n = sum(Observation), conf.level = .95)
```

```
## 
##  1-sample proportions test with continuity correction
## 
## data:  Observation[2] out of sum(Observation), null probability 0.5
## X-squared = 80.132, df = 1, p-value < 2.2e-16
## alternative hypothesis: true p is not equal to 0.5
## 95 percent confidence interval:
##  0.0847233 0.1995018
## sample estimates:
##         p 
## 0.1324503
```

```
#Confidence-intervals around sample proportion relative to expected proportion
round(prop.test(x = Observation[2], n = sum(Observation), conf.level = .95)$estimate/expected_ratio_6574,2)
```

```
##    p 
## 0.77
```

```
round(as.vector(prop.test(x = Observation[2], n = sum(Observation), conf.level = .95)$conf.int)/expected_ratio_6574,2)
```

```
## [1] 0.49 1.16
```

```
#Confidence-intervals around sample proportion
prop.test(x = Observation[3], n = sum(Observation), conf.level = .95)
```

```
## 
##  1-sample proportions test with continuity correction
## 
## data:  Observation[3] out of sum(Observation), null probability 0.5
## X-squared = 126.12, df = 1, p-value < 2.2e-16
## alternative hypothesis: true p is not equal to 0.5
## 95 percent confidence interval:
##  0.01625461 0.08830168
## sample estimates:
##         p 
## 0.0397351
```

```
#Confidence-intervals around sample proportion relative to expected proportion
round(prop.test(x = Observation[3], n = sum(Observation), conf.level = .95)$estimate/expected_ratio_75plus,2)
```

```
##    p 
## 0.22
```

```
round(as.vector(prop.test(x = Observation[3], n = sum(Observation), conf.level = .95)$conf.int)/expected_ratio_75plus,2)
```

```
## [1] 0.09 0.49
```

```
###########################
#Obsessive Compulsive Disorder#
###########################
prevalence_1834 <- as.numeric(Prevalence_estimates[Prevalence_estimates$Diagnosis == "OCD", "1834y"])
prevalence_3549 <- as.numeric(Prevalence_estimates[Prevalence_estimates$Diagnosis == "OCD", "3549y"])
prevalence_5064 <- as.numeric(Prevalence_estimates[Prevalence_estimates$Diagnosis == "OCD", "5064y"])
prevalence_6574 <- as.numeric(Prevalence_estimates[Prevalence_estimates$Diagnosis == "OCD", "6574y"])
prevalence_75plus <- as.numeric(Prevalence_estimates[Prevalence_estimates$Diagnosis == "OCD", "75yplus"])

expected_ratio_1834 <- (census_amount_1834*prevalence_1834)/((census_amount_1834*prevalence_1834)+(census_amount_3549*prevalence_3549)+(census_amount_5064*prevalence_5064)+(census_amount_6574*prevalence_6574)+(census_amount_75plus*prevalence_75plus))
expected_ratio_3549 <- (census_amount_3549*prevalence_3549)/((census_amount_1834*prevalence_1834)+(census_amount_3549*prevalence_3549)+(census_amount_5064*prevalence_5064)+(census_amount_6574*prevalence_6574)+(census_amount_75plus*prevalence_75plus))
expected_ratio_5064 <- (census_amount_5064*prevalence_5064)/((census_amount_1834*prevalence_1834)+(census_amount_3549*prevalence_3549)+(census_amount_5064*prevalence_5064)+(census_amount_6574*prevalence_6574)+(census_amount_75plus*prevalence_75plus))
expected_ratio_6574 <- (census_amount_6574*prevalence_6574)/((census_amount_1834*prevalence_1834)+(census_amount_3549*prevalence_3549)+(census_amount_5064*prevalence_5064)+(census_amount_6574*prevalence_6574)+(census_amount_75plus*prevalence_75plus))
expected_ratio_75plus <- (census_amount_75plus*prevalence_75plus)/((census_amount_1834*prevalence_1834)+(census_amount_3549*prevalence_3549)+(census_amount_5064*prevalence_5064)+(census_amount_6574*prevalence_6574)+(census_amount_75plus*prevalence_75plus))

round(c(expected_ratio_1834,expected_ratio_3549,expected_ratio_5064,expected_ratio_6574,expected_ratio_75plus), 2)
```

```
## [1] 0.50 0.24 0.18 0.04 0.04
```

```
KODAP_data_complete_OCD <- KODAP_data_complete[KODAP_data_complete$ICD1_pre_recode %in% c("F42.X Zwangsstörung") |
                                                            KODAP_data_complete$ICD2_pre_recode %in% c("F42.X Zwangsstörung") |
                                                            KODAP_data_complete$ICD3_pre_recode %in% c("F42.X Zwangsstörung") |
                                                            KODAP_data_complete$ICD4_pre_recode %in% c("F42.X Zwangsstörung") |
                                                            KODAP_data_complete$ICD5_pre_recode %in% c("F42.X Zwangsstörung"),]

table(KODAP_data_complete_OCD$Age_Stepped_2)
```

```
## 
##   0   1   2   3   4 
## 534 182  62  11   2
```

```
round(table(KODAP_data_complete_OCD$Age_Stepped_2)/length(KODAP_data_complete_OCD$Age_Stepped_2)*100, 1)
```

```
## 
##    0    1    2    3    4 
## 67.5 23.0  7.8  1.4  0.3
```

```
#18-34 years
prevalence_1834 <- as.numeric(Prevalence_estimates[Prevalence_estimates$Diagnosis == "OCD", "1834y"])
prevalence_3549 <- as.numeric(Prevalence_estimates[Prevalence_estimates$Diagnosis == "OCD", "3549y"])
prevalence_5064 <- as.numeric(Prevalence_estimates[Prevalence_estimates$Diagnosis == "OCD", "5064y"])

prevalence_1864 <- prevalence_1834

prevalence_6574 <- as.numeric(Prevalence_estimates[Prevalence_estimates$Diagnosis == "OCD", "6574y"])
prevalence_75plus <- as.numeric(Prevalence_estimates[Prevalence_estimates$Diagnosis == "OCD", "75yplus"])

#Ratios in reference population
expected_ratio_1864 <- (census_amount_1834*prevalence_1864)/((census_amount_1834*prevalence_1864)+(census_amount_6574*prevalence_6574)+(census_amount_75plus*prevalence_75plus))
expected_ratio_6574 <- (census_amount_6574*prevalence_6574)/((census_amount_1834*prevalence_1864)+(census_amount_6574*prevalence_6574)+(census_amount_75plus*prevalence_75plus))
expected_ratio_75plus <- (census_amount_75plus*prevalence_75plus)/((census_amount_1834*prevalence_1864)+(census_amount_6574*prevalence_6574)+(census_amount_75plus*prevalence_75plus))

expected_ratios <- c(expected_ratio_1864,
                     expected_ratio_6574,
                     expected_ratio_75plus)
round(expected_ratios,2)
```

```
## [1] 0.86 0.07 0.07
```

```
KODAP_data_complete_OCD <- KODAP_data_complete_s1[KODAP_data_complete_s1$ICD1_pre_recode %in% c("F42.X Zwangsstörung") |
                                                    KODAP_data_complete_s1$ICD2_pre_recode %in% c("F42.X Zwangsstörung") |
                                                    KODAP_data_complete_s1$ICD3_pre_recode %in% c("F42.X Zwangsstörung") |
                                                    KODAP_data_complete_s1$ICD4_pre_recode %in% c("F42.X Zwangsstörung") |
                                                    KODAP_data_complete_s1$ICD5_pre_recode %in% c("F42.X Zwangsstörung"),]

length(KODAP_data_complete_OCD$Patient_ID)
```

```
## [1] 547
```

```
#Observed vs. expected: 
Observation <- as.vector(table(KODAP_data_complete_OCD$Age_Stepped))
Expected <- expected_ratios*length(KODAP_data_complete_OCD$Patient_ID)

Observation
```

```
## [1] 534  11   2
```

```
round(Observation/length(KODAP_data_complete_OCD$Patient_ID)*100,1)
```

```
## [1] 97.6  2.0  0.4
```

```
# Chi-squared test for given probabilities
test <- chisq.test(Observation, p = expected_ratios)
test
```

```
## 
##  Chi-squared test for given probabilities
## 
## data:  Observation
## X-squared = 65.297, df = 2, p-value = 6.622e-15
```

```
# Post-hoc binomial-test for each category with Bonferroni-correction
binom.test(Observation[1], sum(Observation), expected_ratios[1])$p.value*3
```

```
## [1] 3.530773e-21
```

```
binom.test(Observation[2], sum(Observation), expected_ratios[2])$p.value*3
```

```
## [1] 4.686373e-07
```

```
binom.test(Observation[3], sum(Observation), expected_ratios[3])$p.value*3
```

```
## [1] 3.145261e-15
```

```
#Estimating underrepresentation compared to reference population
#Representation quotients
Observation/Expected
```

```
## [1] 1.14055714 0.28546037 0.04966043
```

```
#Confidence-intervals of Representation quotients
#Confidence-intervals around sample proportion
prop.test(x = Observation[2], n = sum(Observation), conf.level = .95)
```

```
## 
##  1-sample proportions test with continuity correction
## 
## data:  Observation[2] out of sum(Observation), null probability 0.5
## X-squared = 501.97, df = 1, p-value < 2.2e-16
## alternative hypothesis: true p is not equal to 0.5
## 95 percent confidence interval:
##  0.01060965 0.03680152
## sample estimates:
##          p 
## 0.02010969
```

```
#Confidence-intervals around sample proportion relative to expected proportion
round(prop.test(x = Observation[2], n = sum(Observation), conf.level = .95)$estimate/expected_ratio_6574,2)
```

```
##    p 
## 0.29
```

```
round(as.vector(prop.test(x = Observation[2], n = sum(Observation), conf.level = .95)$conf.int)/expected_ratio_6574,2)
```

```
## [1] 0.15 0.52
```

```
#Confidence-intervals around sample proportion
prop.test(x = Observation[3], n = sum(Observation), conf.level = .95)
```

```
## 
##  1-sample proportions test with continuity correction
## 
## data:  Observation[3] out of sum(Observation), null probability 0.5
## X-squared = 537.05, df = 1, p-value < 2.2e-16
## alternative hypothesis: true p is not equal to 0.5
## 95 percent confidence interval:
##  0.0006335564 0.0146333230
## sample estimates:
##           p 
## 0.003656307
```

```
#Confidence-intervals around sample proportion relative to expected proportion
round(prop.test(x = Observation[3], n = sum(Observation), conf.level = .95)$estimate/expected_ratio_75plus,2)
```

```
##    p 
## 0.05
```

```
round(as.vector(prop.test(x = Observation[3], n = sum(Observation), conf.level = .95)$conf.int)/expected_ratio_75plus,2)
```

```
## [1] 0.01 0.20
```

```
#35-49 years
prevalence_1834 <- as.numeric(Prevalence_estimates[Prevalence_estimates$Diagnosis == "OCD", "1834y"])
prevalence_3549 <- as.numeric(Prevalence_estimates[Prevalence_estimates$Diagnosis == "OCD", "3549y"])
prevalence_5064 <- as.numeric(Prevalence_estimates[Prevalence_estimates$Diagnosis == "OCD", "5064y"])

prevalence_1864 <- prevalence_3549

prevalence_6574 <- as.numeric(Prevalence_estimates[Prevalence_estimates$Diagnosis == "OCD", "6574y"])
prevalence_75plus <- as.numeric(Prevalence_estimates[Prevalence_estimates$Diagnosis == "OCD", "75yplus"])

#Ratios in reference population
expected_ratio_1864 <- (census_amount_3549*prevalence_1864)/((census_amount_3549*prevalence_1864)+(census_amount_6574*prevalence_6574)+(census_amount_75plus*prevalence_75plus))
expected_ratio_6574 <- (census_amount_6574*prevalence_6574)/((census_amount_3549*prevalence_1864)+(census_amount_6574*prevalence_6574)+(census_amount_75plus*prevalence_75plus))
expected_ratio_75plus <- (census_amount_75plus*prevalence_75plus)/((census_amount_3549*prevalence_1864)+(census_amount_6574*prevalence_6574)+(census_amount_75plus*prevalence_75plus))

expected_ratios <- c(expected_ratio_1864,
                     expected_ratio_6574,
                     expected_ratio_75plus)
round(expected_ratios,2)
```

```
## [1] 0.73 0.13 0.14
```

```
KODAP_data_complete_OCD <- KODAP_data_complete_s2[KODAP_data_complete_s2$ICD1_pre_recode %in% c("F42.X Zwangsstörung") |
                                                    KODAP_data_complete_s2$ICD2_pre_recode %in% c("F42.X Zwangsstörung") |
                                                    KODAP_data_complete_s2$ICD3_pre_recode %in% c("F42.X Zwangsstörung") |
                                                    KODAP_data_complete_s2$ICD4_pre_recode %in% c("F42.X Zwangsstörung") |
                                                    KODAP_data_complete_s2$ICD5_pre_recode %in% c("F42.X Zwangsstörung"),]

length(KODAP_data_complete_OCD$Patient_ID)
```

```
## [1] 195
```

```
#Observed vs. expected: 
Observation <- as.vector(table(KODAP_data_complete_OCD$Age_Stepped))
Expected <- expected_ratios*length(KODAP_data_complete_OCD$Patient_ID)

Observation
```

```
## [1] 182  11   2
```

```
round(Observation/length(KODAP_data_complete_OCD$Patient_ID)*100,1)
```

```
## [1] 93.3  5.6  1.0
```

```
# Chi-squared test for given probabilities
test <- chisq.test(Observation, p = expected_ratios)
test
```

```
## 
##  Chi-squared test for given probabilities
## 
## data:  Observation
## X-squared = 41.064, df = 2, p-value = 1.211e-09
```

```
# Post-hoc binomial-test for each category with Bonferroni-correction
binom.test(Observation[1], sum(Observation), expected_ratios[1])$p.value*3
```

```
## [1] 4.902649e-12
```

```
binom.test(Observation[2], sum(Observation), expected_ratios[2])$p.value*3
```

```
## [1] 0.003795649
```

```
binom.test(Observation[3], sum(Observation), expected_ratios[3])$p.value*3
```

```
## [1] 1.130132e-09
```

```
#Estimating underrepresentation compared to reference population
#Representation quotients
Observation/Expected
```

```
## [1] 1.2699121 0.4352785 0.0757237
```

```
#Confidence-intervals of Representation quotients
#Confidence-intervals around sample proportion
prop.test(x = Observation[2], n = sum(Observation), conf.level = .95)
```

```
## 
##  1-sample proportions test with continuity correction
## 
## data:  Observation[2] out of sum(Observation), null probability 0.5
## X-squared = 151.71, df = 1, p-value < 2.2e-16
## alternative hypothesis: true p is not equal to 0.5
## 95 percent confidence interval:
##  0.02993022 0.10132864
## sample estimates:
##          p 
## 0.05641026
```

```
#Confidence-intervals around sample proportion relative to expected proportion
round(prop.test(x = Observation[2], n = sum(Observation), conf.level = .95)$estimate/expected_ratio_6574,2)
```

```
##    p 
## 0.44
```

```
round(as.vector(prop.test(x = Observation[2], n = sum(Observation), conf.level = .95)$conf.int)/expected_ratio_6574,2)
```

```
## [1] 0.23 0.78
```

```
#Confidence-intervals around sample proportion
prop.test(x = Observation[3], n = sum(Observation), conf.level = .95)
```

```
## 
##  1-sample proportions test with continuity correction
## 
## data:  Observation[3] out of sum(Observation), null probability 0.5
## X-squared = 185.13, df = 1, p-value < 2.2e-16
## alternative hypothesis: true p is not equal to 0.5
## 95 percent confidence interval:
##  0.00177848 0.04048321
## sample estimates:
##          p 
## 0.01025641
```

```
#Confidence-intervals around sample proportion relative to expected proportion
round(prop.test(x = Observation[3], n = sum(Observation), conf.level = .95)$estimate/expected_ratio_75plus,2)
```

```
##    p 
## 0.08
```

```
round(as.vector(prop.test(x = Observation[3], n = sum(Observation), conf.level = .95)$conf.int)/expected_ratio_75plus,2)
```

```
## [1] 0.01 0.30
```

```
#50-64 years
prevalence_1834 <- as.numeric(Prevalence_estimates[Prevalence_estimates$Diagnosis == "OCD", "1834y"])
prevalence_3549 <- as.numeric(Prevalence_estimates[Prevalence_estimates$Diagnosis == "OCD", "3549y"])
prevalence_5064 <- as.numeric(Prevalence_estimates[Prevalence_estimates$Diagnosis == "OCD", "5064y"])

prevalence_1864 <- prevalence_5064

prevalence_6574 <- as.numeric(Prevalence_estimates[Prevalence_estimates$Diagnosis == "OCD", "6574y"])
prevalence_75plus <- as.numeric(Prevalence_estimates[Prevalence_estimates$Diagnosis == "OCD", "75yplus"])

#Ratios in reference population
expected_ratio_1864 <- (census_amount_5064*prevalence_1864)/((census_amount_5064*prevalence_1864)+(census_amount_6574*prevalence_6574)+(census_amount_75plus*prevalence_75plus))
expected_ratio_6574 <- (census_amount_6574*prevalence_6574)/((census_amount_5064*prevalence_1864)+(census_amount_6574*prevalence_6574)+(census_amount_75plus*prevalence_75plus))
expected_ratio_75plus <- (census_amount_75plus*prevalence_75plus)/((census_amount_5064*prevalence_1864)+(census_amount_6574*prevalence_6574)+(census_amount_75plus*prevalence_75plus))

expected_ratios <- c(expected_ratio_1864,
                     expected_ratio_6574,
                     expected_ratio_75plus)
round(expected_ratios,2)
```

```
## [1] 0.67 0.16 0.17
```

```
KODAP_data_complete_OCD <- KODAP_data_complete_s3[KODAP_data_complete_s3$ICD1_pre_recode %in% c("F42.X Zwangsstörung") |
                                                    KODAP_data_complete_s3$ICD2_pre_recode %in% c("F42.X Zwangsstörung") |
                                                    KODAP_data_complete_s3$ICD3_pre_recode %in% c("F42.X Zwangsstörung") |
                                                    KODAP_data_complete_s3$ICD4_pre_recode %in% c("F42.X Zwangsstörung") |
                                                    KODAP_data_complete_s3$ICD5_pre_recode %in% c("F42.X Zwangsstörung"),]

length(KODAP_data_complete_OCD$Patient_ID)
```

```
## [1] 75
```

```
#Observed vs. expected: 
Observation <- as.vector(table(KODAP_data_complete_OCD$Age_Stepped))
Expected <- expected_ratios*length(KODAP_data_complete_OCD$Patient_ID)

Observation
```

```
## [1] 62 11  2
```

```
round(Observation/length(KODAP_data_complete_OCD$Patient_ID)*100,1)
```

```
## [1] 82.7 14.7  2.7
```

```
# Chi-squared test for given probabilities
test <- chisq.test(Observation, p = expected_ratios)
test
```

```
## 
##  Chi-squared test for given probabilities
## 
## data:  Observation
## X-squared = 11.431, df = 2, p-value = 0.003294
```

```
# Post-hoc binomial-test for each category with Bonferroni-correction
binom.test(Observation[1], sum(Observation), expected_ratios[1])$p.value*3
```

```
## [1] 0.01295206
```

```
binom.test(Observation[2], sum(Observation), expected_ratios[2])$p.value*3
```

```
## [1] 2.625811
```

```
binom.test(Observation[3], sum(Observation), expected_ratios[3])$p.value*3
```

```
## [1] 0.000800487
```

```
#Estimating underrepresentation compared to reference population
#Representation quotients
Observation/Expected
```

```
## [1] 1.2253050 0.9219741 0.1603922
```

```
#Confidence-intervals of Representation quotients
#Confidence-intervals around sample proportion
prop.test(x = Observation[2], n = sum(Observation), conf.level = .95)
```

```
## 
##  1-sample proportions test with continuity correction
## 
## data:  Observation[2] out of sum(Observation), null probability 0.5
## X-squared = 36.053, df = 1, p-value = 1.92e-09
## alternative hypothesis: true p is not equal to 0.5
## 95 percent confidence interval:
##  0.07896563 0.25153073
## sample estimates:
##         p 
## 0.1466667
```

```
#Confidence-intervals around sample proportion relative to expected proportion
round(prop.test(x = Observation[2], n = sum(Observation), conf.level = .95)$estimate/expected_ratio_6574,2)
```

```
##    p 
## 0.92
```

```
round(as.vector(prop.test(x = Observation[2], n = sum(Observation), conf.level = .95)$conf.int)/expected_ratio_6574,2)
```

```
## [1] 0.50 1.58
```

```
#Confidence-intervals around sample proportion
prop.test(x = Observation[3], n = sum(Observation), conf.level = .95)
```

```
## 
##  1-sample proportions test with continuity correction
## 
## data:  Observation[3] out of sum(Observation), null probability 0.5
## X-squared = 65.333, df = 1, p-value = 6.324e-16
## alternative hypothesis: true p is not equal to 0.5
## 95 percent confidence interval:
##  0.004632317 0.101754797
## sample estimates:
##          p 
## 0.02666667
```

```
#Confidence-intervals around sample proportion relative to expected proportion
round(prop.test(x = Observation[3], n = sum(Observation), conf.level = .95)$estimate/expected_ratio_75plus,2)
```

```
##    p 
## 0.16
```

```
round(as.vector(prop.test(x = Observation[3], n = sum(Observation), conf.level = .95)$conf.int)/expected_ratio_75plus,2)
```

```
## [1] 0.03 0.61
```

```
###########################
#Post Traumatic Stress Disorder#
###########################
prevalence_1834 <- as.numeric(Prevalence_estimates[Prevalence_estimates$Diagnosis == "PTSD", "1834y"])
prevalence_3549 <- as.numeric(Prevalence_estimates[Prevalence_estimates$Diagnosis == "PTSD", "3549y"])
prevalence_5064 <- as.numeric(Prevalence_estimates[Prevalence_estimates$Diagnosis == "PTSD", "5064y"])
prevalence_6574 <- as.numeric(Prevalence_estimates[Prevalence_estimates$Diagnosis == "PTSD", "6574y"])
prevalence_75plus <- as.numeric(Prevalence_estimates[Prevalence_estimates$Diagnosis == "PTSD", "75yplus"])

expected_ratio_1834 <- (census_amount_1834*prevalence_1834)/((census_amount_1834*prevalence_1834)+(census_amount_3549*prevalence_3549)+(census_amount_5064*prevalence_5064)+(census_amount_6574*prevalence_6574)+(census_amount_75plus*prevalence_75plus))
expected_ratio_3549 <- (census_amount_3549*prevalence_3549)/((census_amount_1834*prevalence_1834)+(census_amount_3549*prevalence_3549)+(census_amount_5064*prevalence_5064)+(census_amount_6574*prevalence_6574)+(census_amount_75plus*prevalence_75plus))
expected_ratio_5064 <- (census_amount_5064*prevalence_5064)/((census_amount_1834*prevalence_1834)+(census_amount_3549*prevalence_3549)+(census_amount_5064*prevalence_5064)+(census_amount_6574*prevalence_6574)+(census_amount_75plus*prevalence_75plus))
[truncated: 659,987 more chars]
