# Supplementary material for: Quantifying age-related disparities in outpatient psychotherapy utilization: a representation quotient analysis of routine data from 29 university clinics in Germany
Source: BMC Health Serv Res. 2025 Nov 25;25:1558. doi: 10.1186/s12913-025-13714-5 (PMC12670761; doi:10.1186/s12913-025-13714-5)
Supplement: Supplementary file 2 — Supplementary Material 2: Supplement A [file 12913_2025_13714_MOESM2_ESM.pdf]

Quantifying age-related disparities in outpatient psychotherapy utilization: A representation quotient analysis of routine data from 29 university clinics in Germany

Supplement A: Example calculations

1 Example for Calculation of Age Group Distributions in Reference Population

To quantify representation of older adults among the sample, we compared the proportions of older adult groups in the sample with their proportions in the population of adults with a mental disorder. In the following, we provide a step-by-step explanation of the rationale and calculations by which we approximated these population proportions. Probabilistically, the metrics that we aimed to approximate can be expressed as the conditional probability of an individual belonging to a specific age group, given that they have a mental disorder $P(A_i \mid D)$

Using Bayes' theorem, this probability can be expressed as:

Box 1

$$P(A_i \mid D) = \frac{P(D \mid A_i) \cdot P(A_i)}{P(D)}$$

Where:
- $P(A_i \mid D)$: probability that a randomly selected adult individual with a mental disorder belongs to a specific age group (the distribution we want to derive)
- $P(D \mid A_i)$: the prevalence of mental disorders within the respective age group
- $P(A_i)$: represents the general adult population proportion of the respective age group
- $P(D)$: the total proportion of the adult population affected by mental disorders

To apply this framework empirically, we required estimates for:

- $P(A_i)$: We used age distribution in the general adult population, derived from census data, which is outlined in Point 1.1.
- $P(D \mid A_i)$: We used prevalence rates of mental disorders per age group, obtained from epidemiological studies, which is outlined in Point 1.2.
- An exemplary application of the Formula from Box 1 to the obtained estimates is given in Point 1.3.

1.1 Age Group Distribution in the Adult German Population

Age group distributions in the adult German population were approximated using census data from the Federal Statistics Office in Germany. Among the 13,635 adults included in our dataset, 2,095 (15.4%) began therapy in 2018, 2,625 (19.3%) in 2019, 2,432 (17.8%) in 2020, 2,410 (17.6%) in 2021, 2,255 (16.5%) in 2022, and 1,818 (13.3%) in 2023. Hence, we retrieved the relative proportions of working-age adults (18–64 years), young-old adults (65–74 years), and old-old adults (75+ years) in the adult German population for the years 2018 to 2023 from the census statistics, that were as follows:

Year	Working-Age (18–64)	Young-Old (65–74)	Old-Old (75+)
2018	0.7426	0.1211	0.1363
2019	0.7399	0.1228	0.1373
2020	0.7370	0.1278	0.1352
2021	0.7345	0.1318	0.1337
2022	0.7329	0.1346	0.1325
2023	0.7294	0.1377	0.1329

To derive a sample-specific age distribution in the German population, we weighted them by the proportion of the sample originating from each year. The sample-specific proportion of each age group was calculated using the following formula:

Box 2

$$\hat{P}(A_i) = \sum_y w_y \cdot P_y(A_i)$$

Where:
- $\hat{P}(A_i)$: estimated proportion of a specific age group in the sample-adjusted population
- $w_y = n_y/N$: proportion of the sample from year y
- $P_y(A_i)$: proportion of the respective age group in the German population for year y

Hence, the sample-specific proportion of each age group was calculated as follows:

$$\hat{P}(\text{WorkAge}) = \left(\frac{2095}{13635} \cdot 0.7426\right) + \left(\frac{2625}{13635} \cdot 0.7399\right) + \left(\frac{2432}{13635} \cdot 0.7370\right) + \left(\frac{2410}{13635} \cdot 0.7345\right) + \left(\frac{2255}{13635} \cdot 0.7329\right) + \left(\frac{1818}{13635} \cdot 0.7294\right) = 0.7363$$

$$\hat{P}(\text{YoungOld}) = \left(\frac{2095}{13635} \cdot 0.1211\right) + \left(\frac{2625}{13635} \cdot 0.1228\right) + \left(\frac{2432}{13635} \cdot 0.1278\right) + \left(\frac{2410}{13635} \cdot 0.1318\right) + \left(\frac{2255}{13635} \cdot 0.1346\right) + \left(\frac{1818}{13635} \cdot 0.1377\right) = 0.1290$$

$$\hat{P}(\text{OldOld}) = \left(\frac{2095}{13635} \cdot 0.1363\right) + \left(\frac{2625}{13635} \cdot 0.1373\right) + \left(\frac{2432}{13635} \cdot 0.1352\right) + \left(\frac{2410}{13635} \cdot 0.1337\right) + \left(\frac{2255}{13635} \cdot 0.1325\right) + \left(\frac{1818}{13635} \cdot 0.1329\right) = 0.1348$$

1.2 Age-Specific Prevalence Rates of Mental Disorders

To estimate the probability of having a mental disorder given being in a certain age group, we used prevalence estimates from the population-based study conducted in Germany by Jacobi et al. (2014, 2015). The authors reported age-specific prevalence rates for the following groups: 18–34 years, 35–49 years, 50–64 years, and 65–79 years. To derive a pooled prevalence for working-age adults (18–64 years), we weighted the prevalence estimates of the subgroups according to their respective share within the total working-age population. This calculation followed the same approach as outlined in Step 1.1, using census data from 2018 to 2023 using the following formula:

Box 3

$$P(D \mid A_{\text{18-64}}) = \sum_{s} w_s \cdot P(D \mid A_s)$$

Where:
- $P(D \mid A_{\text{18-64}})$: prevalence of mental disorders among the full working-age population (18–64 years)
- s: index for the three working-age subgroups (18–34, 35–49, 50–64 years)
- w_s: proportion of subgroup s within the working-age population
- $P(D \mid A_s)$: prevalence of mental disorders in subgroup s

The estimated proportions of each subgroup within the working-age population were: 35.8% for individuals aged 18–34 years, 28.0% for individuals aged 35–49 years, and 26.4% for individuals aged 50–64 years. Applying the formular above to the reported prevalence rates, we obtained the pooled prevalence for working-age adults. For the young-old and old-old adults, we assumed prevalences according to the estimates provided by Jacobi et al. (2014, 2015) for the age group 65+ years (i.e., 19.6%):

$$\hat{P}(D \mid A_{\text{WorkAge}}) = (0.325 \cdot 0.358) + (0.304 \cdot 0.280) + (0.371 \cdot 0.264) = 0.299$$

$$\hat{P}(D \mid A_{\text{YoungOld}}) = \hat{P}(D \mid A_{\text{OldOld}}) = 0.196$$

1.3 Step 1.3: Calculation of Age Group Distribution in the Reference Population

Based on the previous calculations, we now use the formula from Box 1 to calculate the probability that a randomly selected adult individual with a mental disorder (D) belongs to a specific age group (A_i). Applying the formula, we compute the age group distribution among individuals with a mental disorder as follows:

$$P(A_{\text{WorkAge}} \mid D) = \frac{0.299 \cdot 0.7363}{(0.299 \cdot 0.7363) + (0.196 \cdot 0.1290) + (0.196 \cdot 0.1348)} = 0.8101$$

$$P(A_{\text{YoungOld}} \mid D) = \frac{0.196 \cdot 0.1290}{(0.299 \cdot 0.7363) + (0.196 \cdot 0.1290) + (0.196 \cdot 0.1348)} = 0.0929$$

$$P(A_{\text{OldOld}} \mid D) = \frac{0.196 \cdot 0.1348}{(0.299 \cdot 0.7363) + (0.196 \cdot 0.1290) + (0.196 \cdot 0.1348)} = 0.0971$$

Thus, under the assumptions of our benchmarking scenario, the age distribution within the population of adult individuals with a mental disorder is as follows: 81.0% working-age adults (18–64 years), 9.3% young-old adults (65–74 years), 9.7% old-old adults (75+ years). This distribution serves as the reference for evaluating the observed age composition in the sample.

2 Adjusting for Age-Specific Long-Term Care Needs

One of our sensitivity analyses involved a reanalysis while adjusting for age group specific rates in long-term care needs. Therefore, instead of basing our estimations on the general German population, we only referred to people that are not currently in need of long-term care. Therefore, we calculated the probability of being in a certain age group given that one is not in need of long-term care according to the following formula:

Box 4

$$P(A_i \mid \neg LTC) = \frac{P(A_i) \cdot (1 - P(LTC \mid A_i))}{\sum_g P(A_g) \cdot \left(1 - P(LTC \mid A_g)\right)}$$

Where:
- $P(A_i \mid \neg LTC)$: probability of being in age group A_i given not being in need of long-term care
- $P(A_i)$: proportion of the respective age group in the general population
- $P(LTC \mid A_i)$: probability of being in need of long-term care given age group A_i
- A_g: all age groups considered in the denominator sum

To estimate the probability of being in need of long-term care given that one is in the respective age group, we used age-specific long-term care dependency rates from the German Federal Statistical Office. These were specified for age groups at intervals of 5 years from 20 to 90 years. Further, the statistics was available for the years 2017, 2019, 2021, and 2023. For each year, we calculated the average rate of long-term care dependency in the three age groups by weighting the rates from all 5-year intervals with their proportion of the respective age group in the general German population. The average long-term care dependency rates for the three age-groups were calculated as follows:

Box 5

$$\hat{P}(LTC \mid A_i \cap y) = \sum_{s=1}^{3} w_s \cdot \hat{P}(LTC \mid s \cap y)$$

Where:
- $\hat{P}(LTC \mid A_i \cap y)$: estimated probability of being in need of long-term care given that a person is in age group A_i in year y
- w_s: proportion of each subgroup s within the respective age group A_i
- $\hat{P}(LTC \mid s \cap y)$: probability of being in need of long-term care for subgroup s in year y

For example, the rates of long-term care dependency in the year 2017 as well as the respective proportion in the general population were as follows:

Age Group	LTC Rate (%)	Population Proportion (%)
20–24	0.62	7.63
25–29	0.51	6.40
30–34	0.49	6.39
35–39	0.50	6.24
40–44	0.59	5.78
45–49	0.74	7.18
50–54	1.05	8.42
55–59	1.59	7.73
60–64	2.43	6.49
65–69	3.80	5.69
70–74	6.40	4.36
75–79	11.46	5.11
80–84	23.29	3.48
85–89	44.45	1.81
90–94	67.32	0.75
95+	84.26	0.17

Applying the formula above, the average long-term care dependency rates were calculated as follows:

$$\begin{aligned}\hat{P}(LTC \mid A_{\text{WorkAge}} \cap 2017) = {} & \frac{7.63}{62.26} \cdot 0.0062 + \frac{6.40}{62.26} \cdot 0.0051 + \frac{6.39}{62.26} \cdot 0.0049 + \\ & \frac{6.24}{62.26} \cdot 0.0050 + \frac{5.78}{62.26} \cdot 0.0059 + \frac{7.18}{62.26} \cdot 0.0074 + \\ & \frac{8.42}{62.26} \cdot 0.0105 + \frac{7.73}{62.26} \cdot 0.0159 + \frac{6.49}{62.26} \cdot 0.0243 = 0.0096\end{aligned}$$

$$\hat{P}(LTC \mid A_{\text{YoungOld}} \cap 2017) = \frac{5.69}{10.05} \cdot 0.038 + \frac{4.36}{10.05} \cdot 0.064 = 0.0493$$

$$\begin{aligned}\hat{P}(LTC \mid A_{\text{OldOld}} \cap 2017) = {} & \frac{5.11}{11.32} \cdot 0.1146 + \frac{3.48}{11.32} \cdot 0.2329 + \frac{1.81}{11.32} \cdot 0.4445 + \\ & \frac{0.75}{11.32} \cdot 0.6732 + \frac{0.17}{11.32} \cdot 0.8426 = 0.2512\end{aligned}$$

This procedure was repeated for 2019, 2021, and 2023 which produced the following results.

$$\hat{P}(LTC \mid A_{\text{WorkAge}} \cap 2019) = 0.01209$$
$$\hat{P}(LTC \mid A_{\text{WorkAge}} \cap 2021) = 0.0153$$
$$\hat{P}(LTC \mid A_{\text{WorkAge}} \cap 2023) = 0.0179$$

$$\hat{P}(LTC \mid A_{\text{YoungOld}} \cap 2019) = 0.0592$$
$$\hat{P}(LTC \mid A_{\text{YoungOld}} \cap 2021) = 0.0735$$
$$\hat{P}(LTC \mid A_{\text{YoungOld}} \cap 2023) = 0.0846$$

$$\hat{P}(LTC \mid A_{\text{OldOld}} \cap 2019) = 0.2923$$
$$\hat{P}(LTC \mid A_{\text{OldOld}} \cap 2021) = 0.3477$$
$$\hat{P}(LTC \mid A_{\text{OldOld}} \cap 2023) = 0.3903$$

These long-term care statistics were then weighted with the sample proportion that stem from each year to calculate a sample-specific age distribution in the German population. Thereby, the last available long-term care rate was used for each year from which there were patients in the sample. Hence, the average sample specific long-term care rates were calculated as follows:

Box 6

$$\hat{P}(LTC \mid A_i) = \sum_{y=2018}^{2023} w_y \cdot \hat{P}(LTC \mid A_i \cap y)$$

Where:
- $\hat{P}(LTC \mid A_i)$: estimated probability of being in long-term care given that a person is in age group A_i
- w_y: proportion of the sample from year y, calculated as N_y/N_{total}
- $\hat{P}(LTC \mid A_i \cap y)$: probability of being in long-term care for age group A_i in year y

Hence, the sample specific long-term care rates were as follows:

$$\hat{P}(LTC \mid A_{\text{WorkAge}}) = \frac{2095}{13635} \cdot 0.0096 + \frac{2625}{13635} \cdot 0.0121 + \frac{2432}{13635} \cdot 0.0121 + \frac{2410}{13635} \cdot 0.0153 + \frac{2255}{13635} \cdot 0.0153 + \frac{1818}{13635} \cdot 0.0179 = 0.0136$$

$$\hat{P}(LTC \mid A_{\text{YoungOld}}) = \frac{2095}{13635} \cdot 0.0493 + \frac{2625}{13635} \cdot 0.0592 + \frac{2432}{13635} \cdot 0.0592 + \frac{2410}{13635} \cdot 0.0735 + \frac{2255}{13635} \cdot 0.0735 + \frac{1818}{13635} \cdot 0.0846 = 0.0660$$

$$\hat{P}(LTC \mid A_{\text{OldOld}}) = \frac{2095}{13635} \cdot 0.2512 + \frac{2625}{13635} \cdot 0.2923 + \frac{2432}{13635} \cdot 0.2923 + \frac{2410}{13635} \cdot 0.3477 + \frac{2255}{13635} \cdot 0.3477 + \frac{1818}{13635} \cdot 0.3903 = 0.3180$$

Hence, applying the formula from Box 4, we retrieved the following probabilities of being in a certain age group in the population of people that are not in need of long-term care.

$$\hat{P}(A_{\text{WorkAge}} \mid \neg LTC) = \frac{0.7363 \cdot (1 - 0.0136)}{(0.7363 \cdot (1 - 0.0136)) + (0.1290 \cdot (1 - 0.0660)) + (0.1348 \cdot (1 - 0.3180))} = 0.7738$$

$$\hat{P}(A_{\text{YoungOld}} \mid \neg LTC) = \frac{0.1290 \cdot (1 - 0.0660)}{(0.7363 \cdot (1 - 0.0136)) + (0.1290 \cdot (1 - 0.0660)) + (0.1348 \cdot (1 - 0.3180))} = 0.1289$$

$$\hat{P}(A_{\text{OldOld}} \mid \neg LTC) = \frac{0.1348 \cdot (1 - 0.3180)}{(0.7363 \cdot (1 - 0.0136)) + (0.1290 \cdot (1 - 0.0660)) + (0.1348 \cdot (1 - 0.3180))} = 0.097$$
