# Supplementary material for: Quantifying age-related disparities in outpatient psychotherapy utilization: a representation quotient analysis of routine data from 29 university clinics in Germany
Source: BMC Health Serv Res. 2025 Nov 25;25:1558. doi: 10.1186/s12913-025-13714-5 (PMC12670761; doi:10.1186/s12913-025-13714-5)
Supplement: Supplementary file 3 — Supplementary Material 3: Supplement B [file 12913_2025_13714_MOESM3_ESM.docx]

**Quantifying age-related disparities in outpatient psychotherapy utilization: A representation quotient analysis of routine data from 29 university clinics in Germany -**

**Supplement B**

**Table S1. Groupwise prevalence assumptions based on Jacobi et al. (2014, 2015).**

| Disorder | Prevalences (%) | | | | |
| --- | --- | --- | --- | --- | --- |
|  | Early working-age (18-34 years) | Middle working-age (35-49 years) | Late working-age (50-64 years) | Young-old (65-74 years) | Old-old (≥ 75 years) |
| Any mental disorder | 35.8 | 28.0 | 26.4 | 19.6 | 19.6 |
| Any mood disorder | 15.1 | 10.3 | 7.0 | 5.9 | 5.9 |
| *Major Depressive Disorder* | 10.0 | 7.2 | 5.2 | 4.4 | 4.4 |
| *Dysthymia* | 2.1 | 1.7 | 1.3 | 1.6 | 1.6 |
| Any anxiety disorder | 18.1 | 16.2 | 15.3 | 11.1 | 11.1 |
| *Panic disorder/Agoraphobia* | 4.2 | 4.1 | 4.1 | 3.5 | 3.5 |
| *Social phobia* | 4.6 | 3.1 | 2.2 | 0.7 | 0.7 |
| *Specific phobias* | 12.3 | 9.5 | 10.9 | 8.4 | 8.4 |
| *GAD* | 3.3 | 2.0 | 2.3 | 1.3 | 1.3 |
| OCD | 7.2 | 3.6 | 2.2 | 1.1 | 1.1 |
| PTSD | 3.7 | 2.5 | 1.0 | 1.8 | 1.8 |
| Any somatoform disorder | 4.2 | 3.8 | 3.6 | 2.1 | 2.1 |
| *Somatization disorder* | 0.9 | 0.6 | 0.9 | 0.8 | 0.8 |
| *Pain disorder* | 4.0 | 3.8 | 3.0 | 1.6 | 1.6 |
| Eating disorders | 2.3 | 0.5 | 0.7 | 0.4 | 0.4 |
| Substance use disorders | 8.4 | 5.9 | 5.5 | 2.5 | 2.5 |
| Psychotic disorders | 4.2 | 2.2 | 2.5 | 1.3 | 1.3 |

**Table S2. Groupwise prevalence separated by gender groups assumptions based on Jacobi et al. (2014, 2015).**

| Disorder | Prevalences (%) | | | | | | | | | | |
| --- | --- | --- | --- | --- | --- | --- | --- | --- | --- | --- | --- |
|  | Female | | | | | Male | | | | | |
|  | Early working-age (18-34 years) | Middle working-age (35-49 years) | Late working-age (50-64 years) | Young-old (65-74 years) | Old-old (≥ 75 years) | Early working-age (18-34 years) | Middle working-age (35-49 years) | Late working-age (50-64 years) | Young-old (65-74 years) | Old-old (≥ 75 years) |  |
| Any mental disorder | 41.8 | 34.9 | 31.6 | 24.4 | 24.4 | 30.0 | 21.2 | 21.2 | 14.3 | 14.3 |  |
| Any mood disorder | 20.7 | 14.8 | 7.8 | 8.4 | 8.4 | 9.6 | 5.9 | 6.3 | 3.0 | 3.0 |  |
| *Major Depressive Disorder* | 15.1 | 10.6 | 5.8 | 6.2 | 6.2 | 4.9 | 3.9 | 4.6 | 2.3 | 2.3 |  |
| *Dysthymia* | 2.4 | 2.4 | 1.4 | 2.3 | 2.3 | 1.9 | 0.9 | 1.2 | 0.8 | 0.8 |  |
| Any anxiety disorder | 26.5 | 21.8 | 21.4 | 14.9 | 14.9 | 9.9 | 10.7 | 9.2 | 6.7 | 6.7 |  |
| *Panic disorder/Agoraphobia* | 6.1 | 5.5 | 6.0 | 4.8 | 4.8 | 2.2 | 2.7 | 2.3 | 2.0 | 2.0 |  |
| *Social phobia* | 6.8 | 4.2 | 2.5 | 0.7 | 0.7 | 2.5 | 2.1 | 1.8 | 0.7 | 0.7 |  |
| *Specific phobias* | 19.9 | 13.8 | 16.7 | 11.3 | 11.3 | 4.9 | 5.3 | 5.1 | 5.1 | 5.1 |  |
| *GAD* | 4.7 | 2.3 | 2.8 | 2.1 | 2.1 | 1.9 | 1.7 | 1.8 | 0.4 | 0.4 |  |
| OCD | 7.4 | 4.2 | 2.2 | 2.0 | 2.0 | 7.1 | 3.0 | 2.1 | 0.2 | 0.2 |  |
| PTSD | 6.1 | 3.8 | 1.7 | 2.9 | 2.9 | 1.4 | 1.3 | 0.3 | 0.6 | 0.6 |  |
| Any somatoform disorder | 6.5 | 6.4 | 4.6 | 3.0 | 3.0 | 2.0 | 1.2 | 2.7 | 1.0 | 1.0 |  |
| *Somatization disorder* | 1.3 | 1.2 | 0.3 | 1.1 | 1.1 | 0.5 | 0.1 | 1.5 | 0.5 | 0.5 |  |
| *Pain disorder* | 6.4 | 6.3 | 4.5 | 2.5 | 2.5 | 1.5 | 1.2 | 1.6 | 0.6 | 0.6 |  |
| Eating disorders | 3.6 | 0.8 | 0.9 | 0.3 | 0.3 | 1.0 | 0.1 | 0.4 | 0.5 | 0.5 |  |
| Substance use disorders | 3.9 | 4.4 | 3.0 | 2.6 | 2.6 | 12.8 | 7.5 | 8.0 | 2.4 | 2.4 |  |
| Psychotic disorders | 5.6 | 2.6 | 2.4 | 1.6 | 1.6 | 2.9 | 1.8 | 2.5 | 1.0 | 1.0 |  |

**Table S3. Detailed age distribution among the sample.**

|  | 18 | 19 | 20 | 21 | 22 | 23 | 24 | 25 | 26 | 27 | 28 | 29 | 30 | 31 | 32 | 33 | 34 |
| --- | --- | --- | --- | --- | --- | --- | --- | --- | --- | --- | --- | --- | --- | --- | --- | --- | --- |
| Any mental disorder | 125 (0.92) | 239 (1.75) | 347 (2.54) | 507 (3.72) | 566 (4.15) | 645 (4.73) | 617 (4.53) | 591 (4.33) | 573 (4.20) | 503 (3.69) | 507 (3.72) | 461 (3.38) | 467 (3.43) | 397 (2.91) | 398 (2.92) | 343 (2.52) | 335 (2.46) |
| Any mood disorder | 61 (0.74) | 133 (1.61) | 198 (2.40) | 291 (3.53) | 331 (4.02) | 399 (4.84) | 343 (4.16) | 342 (4.15) | 332 (4.03) | 298 (3.61) | 304 (3.69) | 272 (3.30) | 269 (3.26) | 247 (3.00) | 250 (3.03) | 219 (2.66) | 175 (2.12) |
| *Major Depressive Disorder* | 54 (0.72) | 121 (1.62) | 182 (2.44) | 273 (3.66) | 296 (3.97) | 360 (4.83) | 298 (3.99) | 315 (4.22) | 309 (4.14) | 264 (3.54) | 275 (3.69) | 248 (3.32) | 245 (3.28) | 228 (3.06) | 231 (3.10) | 193 (2.59) | 158 (2.12) |
| *Dysthymia* | 6 (0.63) | 11 (1.15) | 21 (2.19) | 19 (1.98) | 38 (3.97) | 47 (4.91) | 45 (4.70) | 35 (3.65) | 40 (4.18) | 37 (3.86) | 39 (4.07) | 32 (3.34) | 30 (3.13) | 31 (3.24) | 29 (3.03) | 25 (2.61) | 23 (2.40) |
| Any anxiety disorder | 48 (1.08) | 87 (1.95) | 119 (2.67) | 197 (4.42) | 216 (4.85) | 245 (5.50) | 215 (4.83) | 240 (5.39) | 224 (5.03) | 176 (3.95) | 181 (4.06) | 158 (3.55) | 155 (3.48) | 114 (2.56) | 140 (3.14) | 106 (2.38) | 104 (2.34) |
| *Panic disorder/Agoraphobia* | 6 (0.41) | 19 (1.30) | 24 (1.65) | 51 (3.50) | 51 (3.50) | 51 (3.50) | 61 (4.19) | 62 (4.26) | 66 (4.53) | 58 (3.98) | 42 (2.88) | 48 (3.30) | 53 (3.64) | 28 (1.92) | 43 (2.95) | 38 (2.61) | 30 (2.06) |
| *Social phobia* | 31 (1.68) | 52 (2.81) | 71 (3.84) | 108 (5.84) | 128 (6.92) | 146 (7.89) | 116 (6.27) | 136 (7.35) | 104 (5.62) | 82 (4.43) | 93 (5.03) | 75 (4.05) | 66 (3.57) | 44 (2.38) | 69 (3.73) | 42 (2.27) | 44 (2.38) |
| *Specific phobias* | 12 (1.71) | 15 (2.13) | 19 (2.70) | 30 (4.27) | 36 (5.12) | 32 (4.55) | 27 (3.84) | 34 (4.84) | 38 (5.41) | 23 (3.27) | 28 (3.98) | 22 (3.13) | 21 (2.99) | 18 (2.56) | 20 (2.84) | 11 (1.56) | 21 (2.99) |
| *GAD* | 1 (0.18) | 4 (0.72) | 8 (1.45) | 15 (2.72) | 10 (1.81) | 18 (3.26) | 18 (3.26) | 22 (3.99) | 25 (4.53) | 26 (4.71) | 25 (4.53) | 15 (2.72) | 14 (2.54) | 15 (2.72) | 12 (2.17) | 13 (2.36) | 13 (2.36) |
| OCD | 3 (0.38) | 14 (1.77) | 38 (4.80) | 41 (5.18) | 37 (4.68) | 50 (6.32) | 43 (5.44) | 30 (3.79) | 44 (5.56) | 33 (4.17) | 37 (4.68) | 26 (3.29) | 41 (5.18) | 20 (2.53) | 26 (3.29) | 21 (2.65) | 30 (3.79) |
| PTSD | 16 (1.50) | 35 (3.28) | 35 (3.28) | 42 (3.94) | 50 (4.69) | 64 (6.00) | 58 (5.44) | 42 (3.94) | 39 (3.66) | 38 (3.56) | 40 (3.75) | 27 (2.53) | 36 (3.37) | 25 (2.34) | 29 (2.72) | 25 (2.34) | 27 (2.53) |
| Any somatoform disorder | 0 (0.00) | 0 (0.00) | 0 (0.00) | 0 (0.00) | 0 (0.00) | 0 (0.00) | 0 (0.00) | 0 (0.00) | 0 (0.00) | 0 (0.00) | 0 (0.00) | 0 (0.00) | 0 (0.00) | 0 (0.00) | 0 (0.00) | 0 (0.00) | 0 (0.00) |
| *Somatization disorder* | 3 (1.42) | 2 (0.95) | 6 (2.84) | 4 (1.90) | 6 (2.84) | 18 (8.53) | 4 (1.90) | 7 (3.32) | 12 (5.69) | 5 (2.37) | 9 (4.27) | 2 (0.95) | 5 (2.37) | 10 (4.74) | 7 (3.32) | 3 (1.42) | 2 (0.95) |
| *Pain disorder* | 4 (0.79) | 2 (0.39) | 7 (1.38) | 2 (0.39) | 4 (0.79) | 4 (0.79) | 7 (1.38) | 8 (1.57) | 6 (1.18) | 6 (1.18) | 9 (1.77) | 4 (0.79) | 8 (1.57) | 8 (1.57) | 10 (1.97) | 6 (1.18) | 4 (0.79) |
| Eating disorders | 12 (1.40) | 24 (2.80) | 36 (4.20) | 54 (6.30) | 55 (6.42) | 65 (7.58) | 49 (5.72) | 43 (5.02) | 33 (3.85) | 38 (4.43) | 27 (3.15) | 38 (4.43) | 31 (3.62) | 25 (2.92) | 21 (2.45) | 23 (2.68) | 18 (2.10) |
| Substance use disorders | 7 (0.92) | 9 (1.18) | 11 (1.44) | 25 (3.27) | 24 (3.14) | 35 (4.58) | 23 (3.01) | 36 (4.71) | 30 (3.93) | 26 (3.40) | 34 (4.45) | 30 (3.93) | 24 (3.14) | 30 (3.93) | 22 (2.88) | 18 (2.36) | 16 (2.09) |
| Psychotic disorders | 1 (0.30) | 1 (0.30) | 4 (1.19) | 11 (3.27) | 13 (3.87) | 8 (2.38) | 10 (2.98) | 7 (2.08) | 7 (2.08) | 12 (3.57) | 8 (2.38) | 11 (3.27) | 18 (5.36) | 15 (4.46) | 9 (2.68) | 10 (2.98) | 16 (4.76) |

Note: Frequencies are reported as absolute numbers; percentages in parentheses refer to row-wise proportions within mental disorder groups.

**Table S3 (continued). Detailed age distribution among the sample.**

|  | 35 | 36 | 37 | 38 | 39 | 40 | 41 | 42 | 43 | 44 | 45 | 46 | 47 | 48 | 49 |
| --- | --- | --- | --- | --- | --- | --- | --- | --- | --- | --- | --- | --- | --- | --- | --- |
| Any mental disorder | 303 (2.22) | 269 (1.97) | 252 (1.85) | 247 (1.81) | 238 (1.75) | 245 (1.80) | 223 (1.64) | 195 (1.43) | 158 (1.16) | 188 (1.38) | 169 (1.24) | 166 (1.22) | 175 (1.28) | 141 (1.03) | 175 (1.28) |
| Any mood disorder | 178 (2.16) | 156 (1.89) | 144 (1.75) | 155 (1.88) | 142 (1.72) | 147 (1.78) | 130 (1.58) | 127 (1.54) | 91 (1.10) | 110 (1.33) | 104 (1.26) | 96 (1.16) | 109 (1.32) | 78 (0.95) | 110 (1.33) |
| *Major Depressive Disorder* | 162 (2.17) | 139 (1.86) | 133 (1.78) | 142 (1.90) | 130 (1.74) | 139 (1.86) | 116 (1.55) | 108 (1.45) | 80 (1.07) | 96 (1.29) | 93 (1.25) | 87 (1.17) | 102 (1.37) | 74 (0.99) | 95 (1.27) |
| *Dysthymia* | 23 (2.40) | 18 (1.88) | 11 (1.15) | 19 (1.98) | 16 (1.67) | 11 (1.15) | 17 (1.77) | 16 (1.67) | 14 (1.46) | 15 (1.57) | 16 (1.67) | 14 (1.46) | 9 (0.94) | 9 (0.94) | 16 (1.67) |
| Any anxiety disorder | 100 (2.25) | 70 (1.57) | 78 (1.75) | 73 (1.64) | 69 (1.55) | 78 (1.75) | 67 (1.50) | 68 (1.53) | 39 (0.88) | 64 (1.44) | 43 (0.97) | 46 (1.03) | 55 (1.24) | 37 (0.83) | 42 (0.94) |
| *Panic disorder/Agoraphobia* | 37 (2.54) | 29 (1.99) | 26 (1.79) | 28 (1.92) | 26 (1.79) | 31 (2.13) | 28 (1.92) | 30 (2.06) | 20 (1.37) | 28 (1.92) | 19 (1.30) | 22 (1.51) | 19 (1.30) | 17 (1.17) | 10 (0.69) |
| *Social phobia* | 42 (2.27) | 24 (1.30) | 27 (1.46) | 27 (1.46) | 19 (1.03) | 19 (1.03) | 23 (1.24) | 23 (1.24) | 11 (0.59) | 14 (0.76) | 10 (0.54) | 10 (0.54) | 20 (1.08) | 19 (1.03) | 7 (0.38) |
| *Specific phobias* | 14 (1.99) | 9 (1.28) | 11 (1.56) | 9 (1.28) | 11 (1.56) | 14 (1.99) | 17 (2.42) | 5 (0.71) | 7 (1.00) | 11 (1.56) | 5 (0.71) | 6 (0.85) | 15 (2.13) | 4 (0.57) | 16 (2.28) |
| *GAD* | 14 (2.54) | 7 (1.27) | 14 (2.54) | 13 (2.36) | 9 (1.63) | 17 (3.08) | 8 (1.45) | 9 (1.63) | 8 (1.45) | 13 (2.36) | 7 (1.27) | 8 (1.45) | 5 (0.91) | 4 (0.72) | 11 (1.99) |
| OCD | 21 (2.65) | 25 (3.16) | 8 (1.01) | 16 (2.02) | 15 (1.90) | 23 (2.91) | 9 (1.14) | 15 (1.90) | 9 (1.14) | 8 (1.01) | 6 (0.76) | 11 (1.39) | 5 (0.63) | 4 (0.51) | 7 (0.88) |
| PTSD | 20 (1.87) | 22 (2.06) | 21 (1.97) | 25 (2.34) | 20 (1.87) | 24 (2.25) | 19 (1.78) | 10 (0.94) | 15 (1.41) | 13 (1.22) | 10 (0.94) | 15 (1.41) | 13 (1.22) | 8 (0.75) | 9 (0.84) |
| Any somatoform disorder | 0 (0.00) | 0 (0.00) | 0 (0.00) | 0 (0.00) | 0 (0.00) | 0 (0.00) | 0 (0.00) | 0 (0.00) | 0 (0.00) | 0 (0.00) | 0 (0.00) | 0 (0.00) | 0 (0.00) | 0 (0.00) | 0 (0.00) |
| *Somatization disorder* | 2 (0.95) | 3 (1.42) | 3 (1.42) | 4 (1.90) | 2 (0.95) | 1 (0.47) | 3 (1.42) | 1 (0.47) | 5 (2.37) | 5 (2.37) | 3 (1.42) | 1 (0.47) | 2 (0.95) | 2 (0.95) | 5 (2.37) |
| *Pain disorder* | 7 (1.38) | 3 (0.59) | 9 (1.77) | 10 (1.97) | 12 (2.36) | 15 (2.95) | 4 (0.79) | 10 (1.97) | 5 (0.98) | 10 (1.97) | 9 (1.77) | 7 (1.38) | 5 (0.98) | 10 (1.97) | 9 (1.77) |
| Eating disorders | 20 (2.33) | 18 (2.10) | 13 (1.52) | 13 (1.52) | 16 (1.87) | 8 (0.93) | 13 (1.52) | 9 (1.05) | 9 (1.05) | 9 (1.05) | 7 (0.82) | 7 (0.82) | 10 (1.17) | 8 (0.93) | 8 (0.93) |
| Substance use disorders | 20 (2.62) | 24 (3.14) | 16 (2.09) | 14 (1.83) | 22 (2.88) | 21 (2.75) | 12 (1.57) | 11 (1.44) | 11 (1.44) | 8 (1.05) | 12 (1.57) | 16 (2.09) | 9 (1.18) | 10 (1.31) | 11 (1.44) |
| Psychotic disorders | 13 (3.87) | 10 (2.98) | 9 (2.68) | 8 (2.38) | 7 (2.08) | 8 (2.38) | 14 (4.17) | 7 (2.08) | 9 (2.68) | 4 (1.19) | 3 (0.89) | 5 (1.49) | 11 (3.27) | 7 (2.08) | 6 (1.79) |

Note: Frequencies are reported as absolute numbers; percentages in parentheses refer to row-wise proportions within mental disorder groups.

**Table S3 (continued). Detailed age distribution among the sample.**

|  | 50 | 51 | 52 | 53 | 54 | 55 | 56 | 57 | 58 | 59 | 60 | 61 | 62 | 63 | 64 |
| --- | --- | --- | --- | --- | --- | --- | --- | --- | --- | --- | --- | --- | --- | --- | --- |
| Any mental disorder | 195 (1.43) | 180 (1.32) | 182 (1.33) | 230 (1.69) | 183 (1.34) | 209 (1.53) | 200 (1.47) | 165 (1.21) | 167 (1.22) | 164 (1.20) | 165 (1.21) | 121 (0.89) | 120 (0.88) | 99 (0.73) | 73 (0.54) |
| Any mood disorder | 129 (1.56) | 133 (1.61) | 126 (1.53) | 156 (1.89) | 115 (1.39) | 149 (1.81) | 144 (1.75) | 125 (1.52) | 112 (1.36) | 119 (1.44) | 112 (1.36) | 81 (0.98) | 76 (0.92) | 64 (0.78) | 45 (0.55) |
| *Major Depressive Disorder* | 113 (1.51) | 119 (1.60) | 114 (1.53) | 141 (1.89) | 103 (1.38) | 134 (1.80) | 127 (1.70) | 116 (1.55) | 102 (1.37) | 107 (1.43) | 104 (1.39) | 70 (0.94) | 69 (0.92) | 56 (0.75) | 40 (0.54) |
| *Dysthymia* | 18 (1.88) | 15 (1.57) | 15 (1.57) | 16 (1.67) | 10 (1.04) | 17 (1.77) | 22 (2.30) | 15 (1.57) | 14 (1.46) | 16 (1.67) | 11 (1.15) | 10 (1.04) | 7 (0.73) | 11 (1.15) | 6 (0.63) |
| Any anxiety disorder | 56 (1.26) | 50 (1.12) | 53 (1.19) | 49 (1.10) | 53 (1.19) | 63 (1.41) | 47 (1.06) | 47 (1.06) | 50 (1.12) | 42 (0.94) | 43 (0.97) | 28 (0.63) | 29 (0.65) | 30 (0.67) | 24 (0.54) |
| *Panic disorder/Agoraphobia* | 26 (1.79) | 23 (1.58) | 29 (1.99) | 23 (1.58) | 15 (1.03) | 34 (2.34) | 19 (1.30) | 23 (1.58) | 23 (1.58) | 17 (1.17) | 22 (1.51) | 12 (0.82) | 11 (0.76) | 13 (0.89) | 9 (0.62) |
| *Social phobia* | 12 (0.65) | 10 (0.54) | 10 (0.54) | 9 (0.49) | 11 (0.59) | 15 (0.81) | 9 (0.49) | 8 (0.43) | 8 (0.43) | 10 (0.54) | 10 (0.54) | 3 (0.16) | 6 (0.32) | 6 (0.32) | 1 (0.05) |
| *Specific phobias* | 6 (0.85) | 11 (1.56) | 6 (0.85) | 12 (1.71) | 12 (1.71) | 11 (1.56) | 12 (1.71) | 7 (1.00) | 11 (1.56) | 5 (0.71) | 4 (0.57) | 6 (0.85) | 5 (0.71) | 5 (0.71) | 4 (0.57) |
| *GAD* | 11 (1.99) | 6 (1.09) | 11 (1.99) | 7 (1.27) | 14 (2.54) | 12 (2.17) | 8 (1.45) | 8 (1.45) | 7 (1.27) | 12 (2.17) | 4 (0.72) | 3 (0.54) | 7 (1.27) | 8 (1.45) | 7 (1.27) |
| OCD | 4 (0.51) | 8 (1.01) | 7 (0.88) | 7 (0.88) | 4 (0.51) | 4 (0.51) | 4 (0.51) | 5 (0.63) | 4 (0.51) | 1 (0.13) | 4 (0.51) | 2 (0.25) | 3 (0.38) | 4 (0.51) | 1 (0.13) |
| PTSD | 12 (1.12) | 22 (2.06) | 8 (0.75) | 16 (1.50) | 11 (1.03) | 18 (1.69) | 16 (1.50) | 9 (0.84) | 12 (1.12) | 16 (1.50) | 18 (1.69) | 7 (0.66) | 5 (0.47) | 5 (0.47) | 5 (0.47) |
| Any somatoform disorder | 33 (8.01) | 30 (7.28) | 19 (4.61) | 35 (8.50) | 22 (5.34) | 29 (7.04) | 27 (6.55) | 25 (6.07) | 27 (6.55) | 24 (5.83) | 22 (5.34) | 16 (3.88) | 17 (4.13) | 13 (3.16) | 8 (1.94) |
| *Somatization disorder* | 3 (1.42) | 3 (1.42) | 3 (1.42) | 4 (1.90) | 5 (2.37) | 7 (3.32) | 4 (1.90) | 3 (1.42) | 4 (1.90) | 3 (1.42) | 4 (1.90) | 4 (1.90) | 4 (1.90) | 3 (1.42) | 2 (0.95) |
| *Pain disorder* | 24 (4.72) | 23 (4.53) | 13 (2.56) | 27 (5.31) | 15 (2.95) | 16 (3.15) | 21 (4.13) | 20 (3.94) | 18 (3.54) | 18 (3.54) | 14 (2.76) | 10 (1.97) | 11 (2.17) | 8 (1.57) | 3 (0.59) |
| Eating disorders | 14 (1.63) | 4 (0.47) | 7 (0.82) | 11 (1.28) | 8 (0.93) | 10 (1.17) | 8 (0.93) | 5 (0.58) | 6 (0.70) | 5 (0.58) | 6 (0.70) | 3 (0.35) | 2 (0.23) | 0 (0.00) | 0 (0.00) |
| Substance use disorders | 7 (0.92) | 9 (1.18) | 9 (1.18) | 13 (1.70) | 14 (1.83) | 20 (2.62) | 7 (0.92) | 7 (0.92) | 9 (1.18) | 10 (1.31) | 10 (1.31) | 4 (0.52) | 5 (0.65) | 6 (0.79) | 1 (0.13) |
| Psychotic disorders | 4 (1.19) | 3 (0.89) | 4 (1.19) | 5 (1.49) | 7 (2.08) | 5 (1.49) | 0 (0.00) | 3 (0.89) | 4 (1.19) | 3 (0.89) | 3 (0.89) | 3 (0.89) | 4 (1.19) | 1 (0.30) | 0 (0.00) |

Note: Frequencies are reported as absolute numbers; percentages in parentheses refer to row-wise proportions within mental disorder groups.

**Table S3 (continued). Detailed age distribution among the sample.**

|  | 65 | 66 | 67 | 68 | 69 | 70 | 71 | 72 | 73 | 74 | 75 | 76 | 77 | 78 | 79 |
| --- | --- | --- | --- | --- | --- | --- | --- | --- | --- | --- | --- | --- | --- | --- | --- |
| Any mental disorder | 53 (0.39) | 48 (0.35) | 52 (0.38) | 33 (0.24) | 46 (0.34) | 27 (0.20) | 19 (0.14) | 16 (0.12) | 16 (0.12) | 14 (0.10) | 15 (0.11) | 14 (0.10) | 15 (0.11) | 9 (0.07) | 10 (0.07) |
| Any mood disorder | 34 (0.41) | 30 (0.36) | 27 (0.33) | 18 (0.22) | 21 (0.25) | 13 (0.16) | 9 (0.11) | 7 (0.08) | 12 (0.15) | 8 (0.10) | 7 (0.08) | 9 (0.11) | 4 (0.05) | 3 (0.04) | 5 (0.06) |
| *Major Depressive Disorder* | 31 (0.42) | 26 (0.35) | 26 (0.35) | 16 (0.21) | 19 (0.25) | 12 (0.16) | 9 (0.12) | 5 (0.07) | 11 (0.15) | 8 (0.11) | 7 (0.09) | 9 (0.12) | 3 (0.04) | 2 (0.03) | 5 (0.07) |
| *Dysthymia* | 2 (0.21) | 8 (0.84) | 2 (0.21) | 3 (0.31) | 1 (0.10) | 2 (0.21) | 0 (0.00) | 2 (0.21) | 2 (0.21) | 0 (0.00) | 0 (0.00) | 0 (0.00) | 0 (0.00) | 1 (0.10) | 0 (0.00) |
| Any anxiety disorder | 16 (0.36) | 15 (0.34) | 19 (0.43) | 10 (0.22) | 19 (0.43) | 11 (0.25) | 3 (0.07) | 4 (0.09) | 5 (0.11) | 4 (0.09) | 3 (0.07) | 3 (0.07) | 4 (0.09) | 3 (0.07) | 3 (0.07) |
| *Panic disorder/Agoraphobia* | 8 (0.55) | 8 (0.55) | 6 (0.41) | 5 (0.34) | 7 (0.48) | 4 (0.27) | 2 (0.14) | 1 (0.07) | 3 (0.21) | 3 (0.21) | 1 (0.07) | 2 (0.14) | 3 (0.21) | 1 (0.07) | 1 (0.07) |
| *Social phobia* | 4 (0.22) | 3 (0.16) | 1 (0.05) | 0 (0.00) | 3 (0.16) | 2 (0.11) | 0 (0.00) | 2 (0.11) | 1 (0.05) | 0 (0.00) | 0 (0.00) | 1 (0.05) | 0 (0.00) | 1 (0.05) | 0 (0.00) |
| *Specific phobias* | 3 (0.43) | 1 (0.14) | 6 (0.85) | 3 (0.43) | 6 (0.85) | 2 (0.28) | 0 (0.00) | 1 (0.14) | 0 (0.00) | 0 (0.00) | 0 (0.00) | 0 (0.00) | 0 (0.00) | 0 (0.00) | 0 (0.00) |
| *GAD* | 2 (0.36) | 3 (0.54) | 4 (0.72) | 2 (0.36) | 5 (0.91) | 3 (0.54) | 0 (0.00) | 1 (0.18) | 0 (0.00) | 0 (0.00) | 1 (0.18) | 0 (0.00) | 0 (0.00) | 1 (0.18) | 2 (0.36) |
| OCD | 2 (0.25) | 0 (0.00) | 3 (0.38) | 2 (0.25) | 2 (0.25) | 1 (0.13) | 0 (0.00) | 0 (0.00) | 1 (0.13) | 0 (0.00) | 0 (0.00) | 1 (0.13) | 0 (0.00) | 0 (0.00) | 0 (0.00) |
| PTSD | 5 (0.47) | 3 (0.28) | 1 (0.09) | 0 (0.00) | 2 (0.19) | 2 (0.19) | 0 (0.00) | 0 (0.00) | 0 (0.00) | 0 (0.00) | 1 (0.09) | 0 (0.00) | 1 (0.09) | 0 (0.00) | 0 (0.00) |
| Any somatoform disorder | 5 (1.21) | 6 (1.46) | 7 (1.70) | 4 (0.97) | 4 (0.97) | 3 (0.73) | 5 (1.21) | 4 (0.97) | 1 (0.24) | 9 (2.18) | 2 (0.49) | 5 (1.21) | 2 (0.49) | 2 (0.49) | 0 (0.00) |
| *Somatization disorder* | 3 (1.42) | 1 (0.47) | 2 (0.95) | 0 (0.00) | 0 (0.00) | 0 (0.00) | 1 (0.47) | 0 (0.00) | 0 (0.00) | 0 (0.00) | 0 (0.00) | 0 (0.00) | 1 (0.47) | 0 (0.00) | 0 (0.00) |
| *Pain disorder* | 1 (0.20) | 4 (0.79) | 3 (0.59) | 2 (0.39) | 2 (0.39) | 2 (0.39) | 3 (0.59) | 4 (0.79) | 0 (0.00) | 9 (1.77) | 1 (0.20) | 5 (0.98) | 0 (0.00) | 2 (0.39) | 0 (0.00) |
| Eating disorders | 3 (0.35) | 2 (0.23) | 0 (0.00) | 0 (0.00) | 0 (0.00) | 0 (0.00) | 0 (0.00) | 1 (0.12) | 2 (0.23) | 0 (0.00) | 0 (0.00) | 0 (0.00) | 0 (0.00) | 0 (0.00) | 0 (0.00) |
| Substance use disorders | 6 (0.79) | 1 (0.13) | 1 (0.13) | 0 (0.00) | 1 (0.13) | 2 (0.26) | 1 (0.13) | 1 (0.13) | 2 (0.26) | 0 (0.00) | 0 (0.00) | 0 (0.00) | 1 (0.13) | 0 (0.00) | 0 (0.00) |
| Psychotic disorders | 0 (0.00) | 1 (0.30) | 2 (0.60) | 0 (0.00) | 0 (0.00) | 0 (0.00) | 0 (0.00) | 1 (0.30) | 0 (0.00) | 0 (0.00) | 1 (0.30) | 0 (0.00) | 0 (0.00) | 0 (0.00) | 0 (0.00) |

Note: Frequencies are reported as absolute numbers; percentages in parentheses refer to row-wise proportions within mental disorder groups.

**Table S3 (continued). Detailed age distribution among the sample.**

|  | 80 | 81 | 82 | 83 | 84 | 85 | 86 | 87 | 88 | 89 | 90 | 91 | 92 | 93 | 94 | 95 | 96 |
| --- | --- | --- | --- | --- | --- | --- | --- | --- | --- | --- | --- | --- | --- | --- | --- | --- | --- |
| Any mental disorder | 11 (0.08) | 4 (0.03) | 4 (0.03) | 2 (0.01) | 3 (0.02) | 1 (0.01) | 1 (0.01) | 0 (0.00) | 0 (0.00) | 1 (0.01) | 0 (0.00) | 0 (0.00) | 1 (0.01) | 0 (0.00) | 0 (0.00) | 0 (0.00) | 2 (0.01) |
| Any mood disorder | 4 (0.05) | 1 (0.01) | 2 (0.02) | 1 (0.01) | 2 (0.02) | 0 (0.00) | 0 (0.00) | 0 (0.00) | 0 (0.00) | 0 (0.00) | 0 (0.00) | 0 (0.00) | 0 (0.00) | 0 (0.00) | 0 (0.00) | 0 (0.00) | 0 (0.00) |
| *Major Depressive Disorder* | 4 (0.05) | 1 (0.01) | 2 (0.03) | 1 (0.01) | 2 (0.03) | 0 (0.00) | 0 (0.00) | 0 (0.00) | 0 (0.00) | 0 (0.00) | 0 (0.00) | 0 (0.00) | 0 (0.00) | 0 (0.00) | 0 (0.00) | 0 (0.00) | 0 (0.00) |
| *Dysthymia* | 0 (0.00) | 0 (0.00) | 0 (0.00) | 0 (0.00) | 0 (0.00) | 0 (0.00) | 0 (0.00) | 0 (0.00) | 0 (0.00) | 0 (0.00) | 0 (0.00) | 0 (0.00) | 0 (0.00) | 0 (0.00) | 0 (0.00) | 0 (0.00) | 0 (0.00) |
| Any anxiety disorder | 5 (0.11) | 2 (0.04) | 1 (0.02) | 1 (0.02) | 2 (0.04) | 0 (0.00) | 1 (0.02) | 0 (0.00) | 0 (0.00) | 0 (0.00) | 0 (0.00) | 0 (0.00) | 1 (0.02) | 0 (0.00) | 0 (0.00) | 0 (0.00) | 0 (0.00) |
| *Panic disorder/Agoraphobia* | 0 (0.00) | 0 (0.00) | 1 (0.07) | 0 (0.00) | 0 (0.00) | 0 (0.00) | 0 (0.00) | 0 (0.00) | 0 (0.00) | 0 (0.00) | 0 (0.00) | 0 (0.00) | 0 (0.00) | 0 (0.00) | 0 (0.00) | 0 (0.00) | 0 (0.00) |
| *Social phobia* | 0 (0.00) | 0 (0.00) | 0 (0.00) | 1 (0.05) | 1 (0.05) | 0 (0.00) | 0 (0.00) | 0 (0.00) | 0 (0.00) | 0 (0.00) | 0 (0.00) | 0 (0.00) | 0 (0.00) | 0 (0.00) | 0 (0.00) | 0 (0.00) | 0 (0.00) |
| *Specific phobias* | 2 (0.28) | 1 (0.14) | 0 (0.00) | 0 (0.00) | 0 (0.00) | 0 (0.00) | 0 (0.00) | 0 (0.00) | 0 (0.00) | 0 (0.00) | 0 (0.00) | 0 (0.00) | 0 (0.00) | 0 (0.00) | 0 (0.00) | 0 (0.00) | 0 (0.00) |
| *GAD* | 1 (0.18) | 0 (0.00) | 1 (0.18) | 0 (0.00) | 0 (0.00) | 0 (0.00) | 0 (0.00) | 0 (0.00) | 0 (0.00) | 0 (0.00) | 0 (0.00) | 0 (0.00) | 0 (0.00) | 0 (0.00) | 0 (0.00) | 0 (0.00) | 0 (0.00) |
| OCD | 1 (0.13) | 0 (0.00) | 0 (0.00) | 0 (0.00) | 0 (0.00) | 0 (0.00) | 0 (0.00) | 0 (0.00) | 0 (0.00) | 0 (0.00) | 0 (0.00) | 0 (0.00) | 0 (0.00) | 0 (0.00) | 0 (0.00) | 0 (0.00) | 0 (0.00) |
| PTSD | 0 (0.00) | 0 (0.00) | 0 (0.00) | 0 (0.00) | 0 (0.00) | 0 (0.00) | 0 (0.00) | 0 (0.00) | 0 (0.00) | 0 (0.00) | 0 (0.00) | 0 (0.00) | 0 (0.00) | 0 (0.00) | 0 (0.00) | 0 (0.00) | 0 (0.00) |
| Any somatoform disorder | 2 (0.49) | 0 (0.00) | 1 (0.24) | 1 (0.24) | 1 (0.24) | 1 (0.24) | 0 (0.00) | 0 (0.00) | 0 (0.00) | 0 (0.00) | 0 (0.00) | 0 (0.00) | 0 (0.00) | 0 (0.00) | 0 (0.00) | 0 (0.00) | 0 (0.00) |
| *Somatization disorder* | 0 (0.00) | 0 (0.00) | 0 (0.00) | 0 (0.00) | 0 (0.00) | 0 (0.00) | 0 (0.00) | 0 (0.00) | 0 (0.00) | 0 (0.00) | 0 (0.00) | 0 (0.00) | 0 (0.00) | 0 (0.00) | 0 (0.00) | 0 (0.00) | 0 (0.00) |
| *Pain disorder* | 1 (0.20) | 0 (0.00) | 1 (0.20) | 1 (0.20) | 1 (0.20) | 1 (0.20) | 0 (0.00) | 0 (0.00) | 0 (0.00) | 0 (0.00) | 0 (0.00) | 0 (0.00) | 0 (0.00) | 0 (0.00) | 0 (0.00) | 0 (0.00) | 0 (0.00) |
| Eating disorders | 0 (0.00) | 0 (0.00) | 0 (0.00) | 0 (0.00) | 0 (0.00) | 0 (0.00) | 0 (0.00) | 0 (0.00) | 0 (0.00) | 0 (0.00) | 0 (0.00) | 0 (0.00) | 0 (0.00) | 0 (0.00) | 0 (0.00) | 0 (0.00) | 0 (0.00) |
| Substance use disorders | 0 (0.00) | 0 (0.00) | 0 (0.00) | 0 (0.00) | 0 (0.00) | 0 (0.00) | 0 (0.00) | 0 (0.00) | 0 (0.00) | 0 (0.00) | 0 (0.00) | 0 (0.00) | 0 (0.00) | 0 (0.00) | 0 (0.00) | 0 (0.00) | 0 (0.00) |
| Psychotic disorders | 0 (0.00) | 0 (0.00) | 0 (0.00) | 0 (0.00) | 0 (0.00) | 0 (0.00) | 0 (0.00) | 0 (0.00) | 0 (0.00) | 0 (0.00) | 0 (0.00) | 0 (0.00) | 0 (0.00) | 0 (0.00) | 0 (0.00) | 0 (0.00) | 0 (0.00) |

Note: Frequencies are reported as absolute numbers; percentages in parentheses refer to row-wise proportions within mental disorder groups.

**Table S4. Global and disorder-specific results from comparisons between expected and observed treatment rates of older adults separated by patient gender.**

|  | Female | | | | | | | | Male | | | | | | | |
| --- | --- | --- | --- | --- | --- | --- | --- | --- | --- | --- | --- | --- | --- | --- | --- | --- |
| Disorder | Population proportions (%) | | | Observed N (%) | | | Representation Quotient [95%-CI] | | Population proportions (%) | | | Observed N (%) | | | Representation Quotient [95%-CI] | |
|  | Working-Age | Young-old | Old-old | Working-Age | Young-old | Old-old | Young-old | Old-old | Working-Age | Young-old | Old-old | Working-Age | Young-old | Old-old | Young-old | Old-old |
| Any mental disorder | 0.78 | 0.10 | 0.12 | 8,447 (96.7) | 224 (2.6) | 67 (0.8) | 0.26 [0.22; 0.29]*** | 0.07 [0.05; 0.08]*** | 0.84 | 0.08 | 0.07 | 4,741 (97.4) | 100 (2.1) | 26 (0.5) | 0.25 [0.20; 0.30]*** | 0.07 [0.05; 0.11]*** |
| Any mood disorder | 0.80 | 0.09 | 0.11 | 5,093 (97.2) | 121 (2.3) | 27 (0.5) | 0.25 [0.21; 0.30]*** | 0.05 [0.03; 0.07]*** | 0.89 | 0.06 | 0.05 | 2,915 (97.7) | 58 (1.9) | 11 (0.4) | 0.32 [0.25; 0.42]*** | 0.07 [0.04; 0.13]*** |
| *Major Depressive Disorder* | 0.80 | 0.09 | 0.11 | 4,639 (97.2) | 109 (2.3) | 25 (0.5) | 0.25 [0.21; 0.30]*** | 0.05 [0.03; 0.07]*** | 0.86 | 0.07 | 0.07 | 2,604 (97.6) | 54 (2.0) | 11 (0.4) | 0.28 [0.21; 0.37]*** | 0.06 [0.03; 0.12]*** |
| *Dysthymia* | 0.68 | 0.15 | 0.17 | 528 (96.2) | 20 (3.6) | 1 (0.2) | 0.25 [0.16; 0.39]*** | 0.01 [0.00; 0.07]*** | 0.84 | 0.08 | 0.07 | 406 (99.5) | 2 (0.5) | 0 (0.0) | 0.06 [0.01; 0.24]*** | 0.00 [0.00; 0.16]*** |
| Any anxiety disorder | 0.79 | 0.10 | 0.11 | 2,784 (96.9) | 69 (2.4) | 21 (0.7) | 0.25 [0.20; 0.32]*** | 0.07 [0.04; 0.10]*** | 0.83 | 0.09 | 0.08 | 1,527 (97.1) | 37 (2.4) | 8 (0.5) | 0.26 [0.18; 0.36]*** | 0.06 [0.03; 0.13]*** |
| *Panic disorder/Agoraphobia* | 0.75 | 0.12 | 0.13 | 923 (96.4) | 29 (3.0) | 5 (0.5) | 0.26 [0.18; 0.38]*** | 0.04 [0.01; 0.10]*** | 0.79 | 0.11 | 0.10 | 476 (95.6) | 18 (3.6) | 4 (0.8) | 0.33 [0.20; 0.53]*** | 0.08 [0.03; 0.22]*** |
| *Social phobia* | 0.94 | 0.03 | 0.03 | 1,094 (99.0) | 9 (0.8) | 2 (0.2) | 0.29 [0.14; 0.57]*** | 0.06 [0.01; 0.22]*** | 0.91 | 0.05 | 0.04 | 734 (98.8) | 7 (0.9) | 2 (0.3) | 0.19 [0.08; 0.41]*** | 0.06 [0.01; 0.24]*** |
| *Specific phobias* | 0.79 | 0.10 | 0.12 | 492 (96.1) | 17 (3.3) | 3 (0.6) | 0.33 [0.20; 0.54]*** | 0.05 [0.01; 0.16]*** | 0.76 | 0.13 | 0.11 | 184 (97.4) | 5 (2.6) | 0 (0.0) | 0.21 [0.08; 0.51]*** | 0.00 [0.00; 0.22]*** |
| *GAD* | 0.79 | 0.10 | 0.11 | 379 (95.0) | 14 (3.5) | 6 (1.5) | 0.36 [0.21; 0.62]*** | 0.13 [0.05; 0.30]*** | 0.94 | 0.03 | 0.03 | 144 (96.0) | 6 (4.0) | 0 (0.0) | 1.17 [0.48; 2.61] | 0.00 [0.00; 1.02]* |
| OCD | 0.85 | 0.07 | 0.08 | 469 (98.1) | 7 (1.5) | 2 (0.4) | 0.20 [0.09; 0.44]*** | 0.05 [0.01; 0.20]*** | 0.98 | 0.01 | 0.01 | 307 (98.7) | 4 (1.3) | 0 (0.0) | 1.60 [0.52; 4.35] | 0.00 [0.00; 2.11] |
| PTSD | 0.76 | 0.11 | 0.13 | 815 (98.7) | 9 (1.1) | 2 (0.2) | 0.10 [0.05; 0.19]*** | 0.02 [0.00; 0.08]*** | 0.84 | 0.09 | 0.08 | 233 (98.3) | 4 (1.7) | 0 (0.0) | 0.20 [0.06; 0.53]*** | 0.00 [0.00; 0.26]*** |
| Any somatoform disorder | 0.82 | 0.08 | 0.09 | 668 (93.2) | 38 (5.3) | 11 (1.5) | 0.65 [0.47; 0.90]** | 0.16 [0.09; 0.30]*** | 0.87 | 0.07 | 0.06 | 340 (95.5) | 10 (2.8) | 6 (1.7) | 0.40 [0.20; 0.75]** | 0.27 [0.11; 0.60]*** |
| *Somatization disorder* | 0.67 | 0.15 | 0.18 | 134 (97.1) | 4 (2.9) | 0 (0.0) | 0.19 [0.06; 0.50]*** | 0.00 [0.00; 0.19]*** | 0.83 | 0.09 | 0.08 | 68 (94.4) | 3 (4.2) | 1 (1.4) | 0.46 [0.12; 1.37] | 0.17 [0.01; 1.04] |
| *Pain disorder* | 0.85 | 0.07 | 0.08 | 336 (90.1) | 28 (7.5) | 9 (2.4) | 1.06 [0.73; 1.53] | 0.29 [0.14; 0.57]*** | 0.89 | 0.06 | 0.05 | 129 (95.6) | 2 (1.5) | 4 (3.0) | 0.25 [0.04; 0.96] | 0.55 [0.18; 1.45] |
| Eating disorders | 0.93 | 0.03 | 0.04 | 754 (99.2) | 6 (0.8) | 0 (0.0) | 0.26 [0.10; 0.59]*** | 0.00 [0.00; 0.18]*** | 0.77 | 0.12 | 0.11 | 95 (97.9) | 2 (2.1) | 0 (0.0) | 0.17 [0.03; 0.65]** | 0.00 [0.00; 0.43]*** |
| Substance use disorders | 0.78 | 0.10 | 0.12 | 317 (98.4) | 5 (1.6) | 0 (0.0) | 0.15 [0.06; 0.37]*** | 0.00 [0.00; 0.12]*** | 0.93 | 0.04 | 0.03 | 431 (97.5) | 10 (2.3) | 1 (0.2) | 0.59 [0.30; 1.10] | 0.00 [0.00; 0.42]*** |
| Psychotic disorders | 0.84 | 0.07 | 0.09 | 153 (100.0) | 0 (0.0) | 0 (0.0) | 0.00 [0.00; 0.42]*** | 0.00 [0.00; 0.36]*** | 0.89 | 0.06 | 0.05 | 176 (97.2) | 4 (2.2) | 1 (0.6) | 0.37 [0.12; 0.99] | 0.10 [0.01; 0.65]** |

*: p < 0.05, **: p < 0.01, ***: p < 0.001, p-values are from Bonferroni-corrected post-hoc binomial tests for age group specific deviations of observed proportions from reference population proportions. Representation quotients indicate the ratio between the proportion of an age group in the observed sample and their expected proportion in the reference population of German adults with a mental disorder.

**Table S5. Global and disorder-specific results from representation quotient analysis while separately comparing them to early (18-34 years), middle (35-49 years) and late working-age adults (50-64 years).**

| Disorder | Reference population proportions (%) | | | | | Observed N (%) | | | | | Representation Quotient [95%-CI] | | | | | |
| --- | --- | --- | --- | --- | --- | --- | --- | --- | --- | --- | --- | --- | --- | --- | --- | --- |
|  | Early working-age | Middle working-age | Late working-age | Young-old | Old-old | Early working-age | Middle working-age | Late working-age | Young-old | Old-old | Young-old vs. | | | Old-old | | |
|  |  |  |  |  |  |  |  |  |  |  | Early working-age | Middle working-age | Late working-age | Early working-age | Middle working-age | Late working-age |
| Any mental disorder | 0.31 | 0.23 | 0.27 | 0.09 | 0.10 | 7,621 (55.9) | 3,144 (23.1) | 2,453 (18.0) | 324 (2.4) | 93 (0.7) | 0.22 [0.20; 0.24]*** | 0.41 [0.37; 0.45]*** | 0.55 [0.50; 0.61]*** | 0.06 [0.05; 0.07]*** | 0.11 [0.09; 0.14]*** | 0.15 [0.12; 0.19]*** |
| Any mood disorder | 0.38 | 0.25 | 0.20 | 0.08 | 0.08 | 4,576 (54.2) | 1,921 (22.7) | 1,725 (20.4) | 185 (2.2) | 38 (0.4) | 0.26 [0.22; 0.30]*** | 0.43 [0.37; 0.50]*** | 0.43 [0.37; 0.49]*** | 0.05 [0.04; 0.07]*** | 0.09 [0.06; 0.12]*** | 0.09 [0.06; 0.12]*** |
| *Major Depressive Disorder* | 0.36 | 0.24 | 0.22 | 0.09 | 0.09 | 4,151 (54.3) | 1,737 (22.7) | 1,552 (20.3) | 168 (2.2) | 36 (0.5) | 0.24 [0.20; 0.28]*** | 0.42 [0.36; 0.48]*** | 0.43 [0.37; 0.50]*** | 0.05 [0.04; 0.07]*** | 0.09 [0.06; 0.12]*** | 0.09 [0.07; 0.13]*** |
| *Dysthymia* | 0.30 | 0.23 | 0.21 | 0.12 | 0.13 | 522 (53.3) | 227 (23.2) | 207 (21.1) | 22 (2.2) | 1 (0.1) | 0.18 [0.12; 0.28]*** | 0.34 [0.22; 0.52]*** | 0.37 [0.24; 0.55]*** | 0.01 [0.00; 0.05]*** | 0.02 [0.00; 0.10]*** | 0.02 [0.00; 0.10]*** |
| Any anxiety disorder | 0.29 | 0.24 | 0.28 | 0.10 | 0.10 | 2,798 (61.2) | 956 (20.9) | 679 (14.9) | 108 (2.4) | 30 (0.7) | 0.19 [0.15; 0.23]*** | 0.45 [0.38; 0.54]*** | 0.66 [0.55: 0.78]*** | 0.05 [0.03; 0.07]*** | 0.12 [0.08; 0.17]*** | 0.17 [0.12; 0.25]*** |
| *Panic disorder/Agoraphobia* | 0.25 | 0.23 | 0.28 | 0.11 | 0.12 | 749 (50.2) | 382 (25.6) | 303 (20.3) | 48 (3.2) | 10 (0.7) | 0.25 [0.19; 0.34]*** | 0.45 [0.34; 0.59]*** | 0.60 [0.45: 0.78]*** | 0.05 [0.02; 0.09]*** | 0.08 [0.04; 0.16]*** | 0.11 [0.05; 0.21]*** |
| *Social phobia* | 0.43 | 0.27 | 0.23 | 0.04 | 0.04 | 1,446 (76.0) | 304 (16.0) | 132 (6.9) | 16 (0.8) | 4 (0.2) | 0.16 [0.09; 0.26]*** | 0.49 [0.29; 0.80]** | 0.94 [0.56; 1.49] | 0.04 [0.01; 0.10]*** | 0.12 [0.04; 0.32]*** | 0.23 [0.07; 0.60]*** |
| *Specific phobias* | 0.29 | 0.21 | 0.29 | 0.11 | 0.11 | 416 (57.7) | 158 (21.9) | 121 (16.8) | 23 (3.2) | 3 (0.4) | 0.24 [0.16; 0.37]*** | 0.49 [0.32; 0.73]** | 0.74 [0.49; 1.09] | 0.03 [0.01; 0.10]*** | 0.06 [0.02; 0.20]*** | 0.10 [0.03; 0.30]*** |
| *GAD* | 0.36 | 0.20 | 0.28 | 0.08 | 0.08 | 261 (46.1) | 150 (26.5) | 129 (22.8) | 20 (3.5) | 6 (1.1) | 0.48 [0.30; 0.74]*** | 0.54 [0.35; 0.82]** | 0.76 [0.49; 1.15] | 0.14 [0.06; 0.31]*** | 0.16 [0.06; 0.35]*** | 0.22 [0.09; 0.49]*** |
| OCD | 0.50 | 0.24 | 0.18 | 0.04 | 0.04 | 545 (67.7) | 183 (22.7) | 64 (8.0) | 11 (1.4) | 2 (0.2) | 0.28 [0.15; 0.52]*** | 0.43 [0.23; 0.78]** | 0.92 [0.49; 1.57] | 0.05 [0.01; 0.20]*** | 0.08 [0.01; 0.30]*** | 0.16 [0.03; 0.61]*** |
| PTSD | 0.40 | 0.25 | 0.12 | 0.11 | 0.11 | 653 (59.1) | 252 (22.8) | 184 (16.7) | 13 (1.2) | 3 (0.3) | 0.12 [0.07; 0.21]*** | 0.22 [0.12; 0.38]*** | 0.21 [0.12; 0.37]*** | 0.02 [0.00; 0.07]*** | 0.03 [0.01; 0.13]*** | 0.03 [0.01; 0.13]*** |
| Any somatoform disorder | 0.30 | 0.25 | 0.29 | 0.08 | 0.08 | 411 (37.0) | 271 (24.4) | 357 (32.2) | 51 (4.6) | 20 (1.8) | 0.59 [0.45; 0.78]*** | 0.76 [0.57; 0.99] | 0.66 [0.50; 0.86]** | 0.20 [0.12; 0.33]*** | 0.26 [0.16; 0.41]*** | 0.22 [0.14; 0.36]*** |
| *Somatization disorder* | 0.27 | 0.17 | 0.30 | 0.13 | 0.13 | 108 (50.0) | 44 (20.4) | 56 (25.9) | 7 (3.2) | 1 (0.5) | 0.25 [0.11; 0.53]*** | 0.47 [0.21; 0.91]* | 0.48 [0.22; 0.96] | 0.04 [0.00; 0.22]*** | 0.06 [0.00; 0.39]*** | 0.07 [0.00; 0.41]*** |
| *Pain disorder* | 0.31 | 0.28 | 0.27 | 0.07 | 0.07 | 102 (19.4) | 128 (24.3) | 248 (47.1) | 33 (6.3) | 16 (3.0) | 1.40 [0.99; 1.92] | 1.10 [0.77; 1.51] | 0.63 [0.44; 0.89]* | 0.59 [0.33; 0.99] | 0.46 [0.26; 0.78]** | 0.26 [0.15; 0.46]*** |
| Eating disorders | 0.57 | 0.12 | 0.20 | 0.05 | 0.06 | 599 (68.8) | 171 (19.6) | 93 (10.7) | 8 (0.9) | 0 (0.0) | 0.17 [0.08; 0.34]*** | 0.19 [0.09; 0.38]*** | 0.47 [0.22; 0.92]* | 0.00 [0.00; 0.10]*** | 0.00 [0.00; 0.11]*** | 0.00 [0.00; 0.26]*** |
| Substance use disorders | 0.37 | 0.24 | 0.27 | 0.06 | 0.06 | 408 (52.5) | 219 (28.2) | 134 (17.2) | 15 (1.9) | 1 (0.1) | 0.30 [0.17; 0.49]*** | 0.39 [0.23; 0.65]*** | 0.68 [0.40; 1.11] | 0.02 [0.00; 0.12]*** | 0.03 [0.00; 0.16]*** | 0.04 [0.00; 0.28]*** |
| Psychotic disorders | 0.40 | 0.20 | 0.27 | 0.07 | 0.07 | 163 (47.7) | 122 (35.7) | 52 (15.2) | 4 (1.2) | 1 (0.3) | 0.19 [0.06; 0.52]*** | 0.16 [0.05; 0.42]*** | 0.45 [0.15; 1.14] | 0.05 [0.00; 0.29]*** | 0.04 [0.00; 0.24]*** | 0.11 [0.01; 0.66]*** |

*: p < 0.05, **: p < 0.01, ***: p < 0.001, p-values are from Bonferroni-corrected post-hoc binomial tests for age group specific deviations of observed proportions from reference population proportions. Representation quotients indicate the ratio between the proportion of an age group in the observed sample and their expected proportion in the reference population of German adults with a mental disorder.

**Table S6. Global and disorder-specific results from representation quotient analysis while assuming equal prevalences across age groups.**

| Disorder | Reference population proportions (%) | | | Observed N (%) | | | Representation Quotient  [95%-CI] | |
| --- | --- | --- | --- | --- | --- | --- | --- | --- |
|  | Working-Age | Young-old | Old-old | Working-Age | Young-old | Old-old | Young-old | Old-old |
| Any mental disorder | 0.74 | 0.13 | 0.13 | 13,218 (96.9) | 324 (2.4) | 93 (0.7) | 0.18 [0.16; 0.20]*** | 0.05 [0.04; 0.06]*** |
| Any mood disorder | 0.74 | 0.13 | 0.13 | 8,027  (97.4) | 179  (2.2) | 38  (0.5) | 0.17 [0.14; 0.19]*** | 0.03 [0.02; 0.05]*** |
| *Major Depressive Disorder* | 0.74 | 0.13 | 0.13 | 7,261  (97.3) | 163  (2.2) | 36  (0.5) | 0.17 [0.14; 0.20]*** | 0.04 [0.03; 0.05]*** |
| *Dysthymia* | 0.74 | 0.13 | 0.13 | 935  (97.6) | 22  (2.3) | 1  (0.1) | 0.18 [0.11; 0.27]*** | 0.01 [0.00; 0.05]*** |
| Any anxiety disorder | 0.74 | 0.13 | 0.13 | 4,318  (97.0) | 106  (2.4) | 29  (0.7) | 0.18 [0.15; 0.22]*** | 0.05 [0.03; 0.07]*** |
| *Panic disorder/Agoraphobia* | 0.74 | 0.13 | 0.13 | 1,400  (96.2) | 47  (3.2) | 9  (0.6) | 0.25 [0.19; 0.33]*** | 0.05 [0.02; 0.09]*** |
| *Social phobia* | 0.74 | 0.13 | 0.13 | 1,830  (98.9) | 16  (0.9) | 4  (0.2) | 0.07 [0.04; 0.11]*** | 0.02 [0.01; 0.04]*** |
| *Specific phobias* | 0.74 | 0.13 | 0.13 | 678  (96.4) | 22  (3.1) | 3  (0.4) | 0.24 [0.16; 0.37]*** | 0.03 [0.01; 0.10]*** |
| *GAD* | 0.74 | 0.13 | 0.13 | 526  (95.3) | 20  (3.6) | 6  (1.1) | 0.28 [0.18; 0.37]*** | 0.08 [0.03; 0.18]*** |
| OCD | 0.74 | 0.13 | 0.13 | 778  (98.4) | 11  (1.4) | 2  (0.3) | 0.11 [0.06; 0.20]*** | 0.02 [0.00; 0.08]*** |
| PTSD | 0.74 | 0.13 | 0.13 | 1,052  (98.6) | 13  (1.2) | 2  (0.2) | 0.09 [0.05; 0.16]*** | 0.01 [0.00; 0.06]*** |
| Any somatoform disorder | 0.74 | 0.13 | 0.13 | 1,009  (93.9) | 48  (4.5) | 17  (1.6) | 0.34 [0.26; 0.46]*** | 0.12 [0.07; 0.19]*** |
| *Somatization disorder* | 0.74 | 0.13 | 0.13 | 203 (96.2) | 7  (3.3) | 1  (0.5) | 0.26 [0.11; 0.54]*** | 0.04 [0.00; 0.22]*** |
| *Pain disorder* | 0.74 | 0.13 | 0.13 | 465 (91.5) | 30  (5.9) | 13 (2.6) | 0.46 [0.32; 0.65]*** | 0.19 [0.11; 0.33]*** |
| Eating disorders | 0.74 | 0.13 | 0.13 | 849  (99.1) | 8  (0.9) | 0  (0.0) | 0.07 [0.03; 0.15]*** | 0.00 [0.00; 0.04]*** |
| Substance use disorders | 0.74 | 0.13 | 0.13 | 748  (97.9) | 15  (2.0) | 1  (0.1) | 0.15 [0.09; 0.25]*** | 0.01 [0.00; 0.06]*** |
| Psychotic disorders | 0.74 | 0.13 | 0.13 | 331  (98.5) | 4  (1.2) | 1  (0.3) | 0.09 [0.03; 0.25]*** | 0.02 [0.00; 0.14]*** |

*: p < 0.05, **: p < 0.01, ***: p < 0.001, p-values are from Bonferroni-corrected post-hoc binomial tests for age group specific deviations of observed proportions from reference population proportions. Representation quotients indicate the ratio between the proportion of an age group in the observed sample and their expected proportion in the reference population of German adults with a mental disorder.

**Table S7. Global and disorder-specific results from representation quotient analysis while assuming a prevalence ratio of 0.5 between young-old and old-old adults.**

| Disorder | Reference population proportions (%) | | | Observed N (%) | | | Representation Quotient  [95%-CI] | |
| --- | --- | --- | --- | --- | --- | --- | --- | --- |
|  | Working-Age | Young-old | Old-old | Working-Age | Young-old | Old-old | Young-old | Old-old |
| Any mental disorder | 0.85 | 0.10 | 0.05 | 13,218 (96.9) | 324 (2.4) | 93 (0.7) | 0.24 [0.22; 0.27]*** | 0.13 [0.11; 0.16]*** |
| Any mood disorder | 0.87 | 0.09 | 0.04 | 8,027  (97.4) | 179  (2.2) | 38  (0.5) | 0.26 [0.22; 0.30]*** | 0.10 [0.07; 0.14]*** |
| *Major Depressive Disorder* | 0.86 | 0.09 | 0.05 | 7,261  (97.3) | 163  (2.2) | 36  (0.5) | 0.24 [0.21; 0.28]*** | 0.10 [0.07; 0.14]*** |
| *Dysthymia* | 0.80 | 0.13 | 0.07 | 935  (97.6) | 22  (2.3) | 1  (0.1) | 0.17 [0.11; 0.26]*** | 0.02 [0.00; 0.10]*** |
| Any anxiety disorder | 0.85 | 0.10 | 0.05 | 4,318  (97.0) | 106  (2.4) | 29  (0.7) | 0.24 [0.20; 0.29]*** | 0.12 [0.09; 0.18]*** |
| *Panic disorder/Agoraphobia* | 0.82 | 0.12 | 0.06 | 1,400  (96.2) | 47  (3.2) | 9  (0.6) | 0.27 [0.20; 0.35]*** | 0.10 [0.05; 0.19]*** |
| *Social phobia* | 0.95 | 0.04 | 0.02 | 1,830  (98.9) | 16  (0.9) | 4  (0.2) | 0.24 [0.14; 0.40]*** | 0.12 [0.04; 0.32]*** |
| *Specific phobias* | 0.83 | 0.11 | 0.06 | 678  (96.4) | 22  (3.1) | 3  (0.4) | 0.28 [0.18; 0.43]*** | 0.07 [0.02; 0.23]*** |
| *GAD* | 0.88 | 0.08 | 0.04 | 526  (95.3) | 20  (3.6) | 6  (1.1) | 0.46 [0.29; 0.71]*** | 0.26 [0.11; 0.60]*** |
| OCD | 0.94 | 0.04 | 0.02 | 778  (98.4) | 11  (1.4) | 2  (0.3) | 0.33 [0.17; 0.60]*** | 0.11 [0.02; 0.46]*** |
| PTSD | 0.83 | 0.11 | 0.06 | 1,052  (98.6) | 13  (1.2) | 2  (0.2) | 0.11 [0.06; 0.19]*** | 0.03 [0.00; 0.13]*** |
| Any somatoform disorder | 0.87 | 0.08 | 0.04 | 1,009  (93.9) | 48  (4.5) | 17  (1.6) | 0.53 [0.40; 0.71]*** | 0.36 [0.22; 0.59]*** |
| *Somatization disorder* | 0.79 | 0.14 | 0.07 | 203 (96.2) | 7  (3.3) | 1  (0.5) | 0.24 [0.11; 0.51]*** | 0.07 [0.00; 0.42]*** |
| *Pain disorder* | 0.89 | 0.07 | 0.04 | 465 (91.5) | 30  (5.9) | 13 (2.6) | 0.84 [0.58; 1.19] | 0.70 [0.39; 1.22] |
| Eating disorders | 0.92 | 0.06 | 0.03 | 849  (99.1) | 8  (0.9) | 0  (0.0) | 0.17 [0.08; 0.34]*** | 0.00 [0.00; 0.19]*** |
| Substance use disorders | 0.91 | 0.06 | 0.03 | 748  (97.9) | 15  (2.0) | 1  (0.1) | 0.32 [0.19; 0.54]*** | 0.04 [0.00; 0.27]*** |
| Psychotic disorders | 0.89 | 0.07 | 0.04 | 331  (98.5) | 4  (1.2) | 1  (0.3) | 0.17 [0.06; 0.47]*** | 0.08 [0.00; 0.53]*** |

*: p < 0.05, **: p < 0.01, ***: p < 0.001, p-values are from Bonferroni-corrected post-hoc binomial tests for age group specific deviations of observed proportions from reference population proportions. Representation quotients indicate the ratio between the proportion of an age group in the observed sample and their expected proportion in the reference population of German adults with a mental disorder.

**Table S8. Global and disorder-specific results from representation quotient analysis while adjusting for age-specific rates of long-term care needs.**

| Disorder | Reference population proportions (%) | | | Observed N (%) | | | Representation Quotient  [95%-CI] | |
| --- | --- | --- | --- | --- | --- | --- | --- | --- |
|  | Working-Age | Young-old | Old-old | Working-Age | Young-old | Old-old | Young-old | Old-old |
| Any mental disorder | 0.84 | 0.09 | 0.07 | 13,218 (96.9) | 324 (2.4) | 93 (0.7) | 0.26 [0.23; 0.29]*** | 0.10 [0.08; 0.12]*** |
| Any mood disorder | 0.86 | 0.08 | 0.06 | 8,027  (97.4) | 179  (2.2) | 38  (0.5) | 0.27 [0.24; 0.32]*** | 0.08 [0.06; 0.11]*** |
| *Major Depressive Disorder* | 0.85 | 0.08 | 0.06 | 7,261  (97.3) | 163  (2.2) | 36  (0.5) | 0.26 [0.22; 0.30]*** | 0.08 [0.05; 0.11]*** |
| *Dysthymia* | 0.78 | 0.12 | 0.09 | 935  (97.6) | 22  (2.3) | 1  (0.1) | 0.19 [0.12; 0.28]*** | 0.01 [0.00; 0.07]*** |
| Any anxiety disorder | 0.84 | 0.09 | 0.07 | 4,318  (97.0) | 106  (2.4) | 29  (0.7) | 0.25 [0.21; 0.31]*** | 0.09 [0.06; 0.13]*** |
| *Panic disorder/Agoraphobia* | 0.80 | 0.11 | 0.09 | 1,400  (96.2) | 47  (3.2) | 9  (0.6) | 0.29 [0.21; 0.38]*** | 0.07 [0.04; 0.14]*** |
| *Social phobia* | 0.94 | 0.03 | 0.03 | 1,830  (98.9) | 16  (0.9) | 4  (0.2) | 0.26 [0.15; 0.42]*** | 0.09 [0.03; 0.23]*** |
| *Specific phobias* | 0.82 | 0.10 | 0.08 | 678  (96.4) | 22  (3.1) | 3  (0.4) | 0.30 [0.19; 0.46]*** | 0.05 [0.01; 0.17]*** |
| *GAD* | 0.87 | 0.07 | 0.06 | 526  (95.3) | 20  (3.6) | 6  (1.1) | 0.49 [0.31; 0.76]*** | 0.19 [0.08; 0.44]*** |
| OCD | 0.93 | 0.04 | 0.03 | 778  (98.4) | 11  (1.4) | 2  (0.3) | 0.35 [0.18; 0.64]*** | 0.08 [0.01; 0.34]*** |
| PTSD | 0.82 | 0.10 | 0.08 | 1,052  (98.6) | 13  (1.2) | 2  (0.2) | 0.12 [0.06; 0.20]*** | 0.02 [0.00; 0.10]*** |
| Any somatoform disorder | 0.86 | 0.08 | 0.06 | 1,009  (93.9) | 48  (4.5) | 17  (1.6) | 0.57 [0.43; 0.76]*** | 0.27 [0.16; 0.44]*** |
| *Somatization disorder* | 0.78 | 0.13 | 0.10 | 203 (96.2) | 7  (3.3) | 1  (0.5) | 0.26 [0.11; 0.55]*** | 0.05 [0.00; 0.31]*** |
| *Pain disorder* | 0.88 | 0.07 | 0.05 | 465 (91.5) | 30  (5.9) | 13 (2.6) | 0.89 [0.62; 1.27] | 0.51 [0.29; 0.89]* |
| Eating disorders | 0.91 | 0.05 | 0.04 | 849  (99.1) | 8  (0.9) | 0  (0.0) | 0.18 [0.08; 0.37]*** | 0.00 [0.00; 0.14]*** |
| Substance use disorders | 0.90 | 0.06 | 0.04 | 748  (97.9) | 15  (2.0) | 1  (0.1) | 0.34 [0.20; 0.58]*** | 0.03 [0.00; 0.20]*** |
| Psychotic disorders | 0.89 | 0.06 | 0.05 | 331  (98.5) | 4  (1.2) | 1  (0.3) | 0.18 [0.06; 0.50]*** | 0.06 [0.00; 0.39]*** |

*: p < 0.05, **: p < 0.01, ***: p < 0.001, p-values are from Bonferroni-corrected post-hoc binomial tests for age group specific deviations of observed proportions from reference population proportions. Representation quotients indicate the ratio between the proportion of an age group in the observed sample and their expected proportion in the reference population of German adults with a mental disorder.
